# Nuclear-specific reductive carboxylation of alpha-ketoglutarate fuels histone acetylation to induce chromatin accessibility and gene activation

**DOI:** 10.1038/s41467-026-74786-3

**Published:** 2026-07-01

**Authors:** Abhisha Sawant Dessai, Nadya A. Elhalawany, Tao Dai, Justine J. Jacobi, Christian Prechtl, Alphonse N. Dimeck, Sierra R. Morton, Mark D. Long, Prashant K. Singh, Nagireddy Putluri, Mariana Lopes, Song Liu, Dominic J. Smiraglia, Katerina V. Gurova, Peder J. Lund, Leah A. Gates, Sung Yun Jung, Subhamoy Dasgupta

**Affiliations:** 1 Department of Cell Stress Biology, Roswell Park Comprehensive Cancer Center, Buffalo, NY USA; 2 Department of Biostatistics and Bioinformatics, Roswell Park Comprehensive Cancer Center, Buffalo, NY USA; 3 Department of Cancer Genetics & Genomics, Roswell Park Comprehensive Cancer Center, Buffalo, NY USA; 4 Department of Molecular and Cellular Biology, Baylor College of Medicine, Houston, TX USA; 5 Department of Nutrition, School of Medicine, Case Western Reserve University, Cleveland, OH USA; 6 Department of Biochemistry, School of Medicine, Case Western Reserve University, Cleveland, OH USA; 7 Department of Biochemistry and Molecular Pharmacology, Baylor College of Medicine, Houston, TX USA; 8 Present Address: Penn Epigenetics Institute, Perelman School of Medicine, University of Pennsylvania, Philadelphia, PA USA; 9 Present Address: Children’s Medical Center Research Institute, University of Texas Southwestern Medical Center, Dallas, TX USA

**Keywords:** Cancer metabolism, Epigenetics

## Abstract

Mitochondria remain at the core of cell metabolism, whereas the nucleus integrates cellular and environmental signals to activate genes. However, the mechanisms that directly link cellular metabolism to gene regulation are not well understood. Here we show, a metabolic pathway in the nucleus controls acetylation of histones by nuclear localization of mitochondrial enzymes aconitase (ACO2) and isocitrate dehydrogenase (IDH2). Metabolic tracing studies show that IDH2 and ACO2 catalyze reductive carboxylation of α-ketoglutarate to rapidly synthesize citrate to increase nuclear acetyl-CoA pool. Genetic and proteomic analyses reveal nuclear IDH2 and ACO2 form a complex with KAT2A/GCN5 for acetylation of histones to increase chromatin accessibility and activation of proliferative genes. Robust nuclear expressions of ACO2 and IDH2 drive aggressive tumors indicating the tumorigenic potential of IDH2-ACO2-KAT2A axis. Altogether, our work reveals a paradigm coupling a nuclear metabolic pathway with histone acetylation to control of gene expression that accentuates hyperproliferative phenotype in tumors.

## Abstract (web summary)

The mechanistic links between cellular metabolism and epigenetic regulation in cancer are not well understood. Here the authors find that nuclear localization of two mitochondrial enzymes, IDH2 and ACO2, increases the local abundance of acetyl-CoA to fuel histone acetylation, contributing to activation of proliferative genes in tumor cells.

## Introduction

One of the most critical steps facilitating the initiation of transcription is chromatin decondensation, achieved by the acetylation of histone proteins at gene regulatory regions. Acetylation of the lysine (K) residues in the N-terminal tail of histones reduces their affinity for DNA, thereby loosening the chromatin and providing greater accessibility to the transcription factors^1^. Histone acetyltransferases (HATs) catalyze the transfer of acetyl groups to the histone tails utilizing acetyl-CoA as a substrate^2^. One of the major sources of acetyl-CoA is citrate, which is produced in the mitochondria via the Kreb’s cycle, also known as the tricarboxylic acid cycle (TCA)^3,4^. Acetyl-CoA is membrane impermeable, and due to its unstable thioester bond and volatile nature, its synthesis must be sub-cellular compartment specific, proximal to where it is required^5^. In the nucleus, ATP-citrate lyase (ACLY) catalyzes the conversion of citrate to acetyl-CoA^6^, however its activity is dependent on the availability of citrate. Moreover, condensed chromatin is highly enriched in DNA, proteins, and RNA, causing molecular crowding, which is thought to affect diffusion^7^. This suggests pathways supporting the production of citrate and acetyl-CoA should be closely associated with chromatin to couple metabolism with histone acetylation. Indeed, previous work provided evidence for the presence of mitochondrial enzymes in the nucleus^8,9^, yet it remains elusive whether functional metabolic pathways exist within the nucleus. Moreover, there exists a distinct gap in the field, whether nuclear enzymes synchronize metabolic reactions to provide substrates for HATs on demand at specific gene promoters through their spatio-temporal localization.

In this work, we set out to investigate the mitochondrial enzymes present in the nucleus that control histone acetylation marks, focusing special attention on the role of TCA cycle enzymes. We find that mitochondrial TCA cycle enzymes citrate synthase (CS), aconitase 2 (ACO2), and isocitrate dehydrogenase 2 (IDH2) are present in the nucleus and bind to the chromatin. Metabolic isotope tracing experiments in intact nuclei reveal that IDH2 and ACO2 generate citrate by reductive carboxylation of alpha-ketoglutarate (α-KG), independent of mitochondrial contribution. Further, deep profiling of histone modifications identify H3Ac marks contain acetyl-carbon originating from the α-KG, supporting the notion that local synthesis of acetyl groups in the nucleus are necessary for histone acetylation. By immunoprecipitation and proteomics profiling, we demonstrate that ACO2 and IDH2 but not CS associate with histone H3 and form a complex with KAT2A (Lysine acetyltransferase 2 A, also known as GCN5L2), a HAT. Although the impact of this nuclear metabolic pathway appears to increase global histone acetylation levels and chromatin accessibility, analysis of high-throughput sequencing data identify specific enrichment of pioneering factor FOXA1 on promoters of proliferative genes. Furthermore, manipulating IDH2 and ACO2 levels specifically in the nucleus alter histone acetyl marks independent of mitochondrial contribution, that robustly induce hyperproliferative phenotype in multiple mouse models of cancer. Collectively, our results underscore the importance of reductive carboxylation of α-KG by IDH2 and ACO2 as an indispensable nuclear metabolic pathway that controls transcription of essential genes to sustain cellular proliferation and growth.

## Results

### Mitochondrial TCA cycle enzymes regulating citrate synthesis are found in the nucleus

**Fig. 1:**
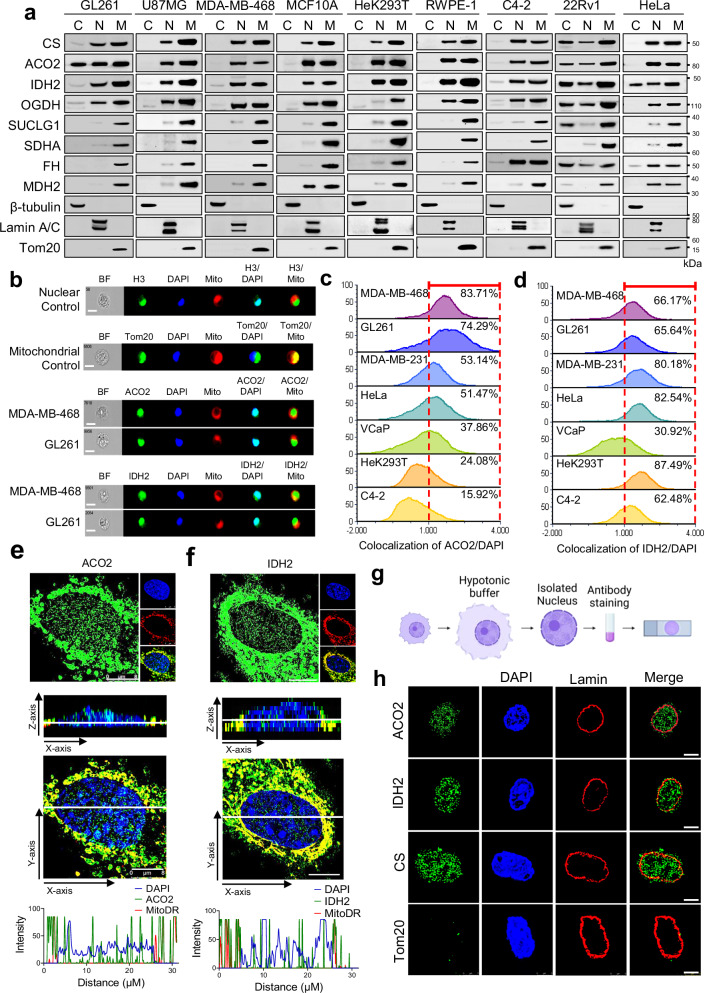
Analysis of sub-cellular distribution of TCA cycle enzymes identified aconitase 2 (ACO2), isocitrate dehydrogenase 2 (IDH2) and citrate synthase (CS) enriched in the nucleus. **a** Mouse and human cell lines were fractionated to isolate sub-cellular proteins localized within cytoplasm (C), nucleus (N), and mitochondria (M). Immunoblotting was performed for mitochondrial Tricarboxylic acid (TCA) cycle enzymes: CS, ACO2, IDH2, Oxoglutarate dehydrogenase (OGDH), Succinyl-CoA-ligase (SUCLG1), Succinate dehydrogenase complex flavoprotein subunit A (SDHA), Fumarate hydratase (FH), and Malate dehydrogenase 2 (MDH2). Cytosolic, nuclear, and mitochondrial markers β-tubulin, Lamin A/C, and Tom20, respectively used for purity. (representative of at least 3 independent experiments for each line). **b** Imaging flow cytometry at single-cell resolution was performed by staining cells with nuclear dye- DAPI (blue) and mitochondrial stain- MitoTracker (deep red). Controls were co-stained with histone H3 or Tom20 (green) in GL261 cells. Immunostaining (green) of ACO2 and IDH2 were performed in GL261 and MDA-MB-468 cell lines. Scale bar 10 µm. (*n* = 10,000 cells, representative of two independent experiments per cell line). **c**,** d** Imaging flow cytometry data shown in (**b**) were quantified to measure the percentage of cells with nuclear localized ACO2 and IDH2 in various cell lines. Similarity feature was used to calculate the colocalization score of (**c**) ACO2 or (**d**) IDH2 merged with DAPI. Cells with colocalization scores ≥1 were considered as positive cells for nuclear-localized ACO2 or IDH2, and the percentage of positive cells were shown on the right. **e**, **f** Confocal microscopy followed by Z-stack analysis of GL261 cells (*n* = 3) co-stained with (**E**) ACO2 (green) or (**f**) IDH2 (green) with DAPI (blue), and MitoDR (red), scanned at 0.2 μm increments along the Z-axis. Mid-plane immunofluorescence intensity at the XY-axis was shown at the bottom. Scale bar 8 µm. (representative fields from 3 independent experiments). **g** Schematic depicting the workflow for rapid isolation of intact nuclei was created in BioRender. https://biorender.com/skjnpbn. **h** Representative images of isolated intact nuclei from GL261 (representative fields from 3 independent experiments) co-stained with ACO2, IDH2, CS, or Tom20 (Green) along with DAPI (Blue) and Lamin A/C (Red). Scale bar 3 µm. Source data are provided as a Source Data file.

Mitochondrial TCA cycle metabolites such as citrate, α-KG, succinyl-CoA, succinate, and fumarate are used as essential cofactors by epigenetic enzymes for chromatin remodeling^10–13^. We conducted an unbiased analysis of TCA cycle enzymes to assess if they may be present in the nucleus and bound to the chromatin. For this, we performed biochemical fractionation of multiple immortalized cell lines, both tumorigenic and non-tumorigenic, from human and mouse origin. Using differential centrifugation, we isolated sub-cellular fractions of cytosol, nucleus, and mitochondria and confirmed the purity of the fractions by analyzing compartment-specific expression of β-tubulin, Lamin A/C, and Tom20 in cytosolic, nuclear, and mitochondrial fractions, respectively (Fig. 1a and Supplementary Fig. S1a). Immunoblotting of the cellular fractions revealed a set of mitochondrial TCA cycle enzymes, compromised of citrate synthase (CS), aconitase 2 (ACO2), isocitrate dehydrogenase 2 (IDH2), and 2-oxoglutarate dehydrogenase (OGDH) that are consistently present in the nuclear fractions across all the cell lines examined (Fig. 1a and Supplementary Fig. S1a). Importantly, three of these enzymes, CS, ACO2, and IDH2, are known to directly modulate mitochondrial citrate levels^4^. In contrast, other TCA cycle enzymes like succinyl CoA ligase (SUCLG1), succinate dehydrogenase complex (SDHA), fumarase (FH), and malate dehydrogenase 2 (MDH2) were predominantly localized only in the mitochondria, with very little present in the nucleus. Next, we quantified the percentage of cells that exhibit nuclear localized ACO2 and IDH2 enzymes within a homogenous population of cells. For this, we performed multispectral immunostaining followed by imaging flow cytometry at a single-cell resolution, allowing the distribution of TCA cycle enzymes within different sub-cellular compartments to be determined. Cells were stained with ACO2 or IDH2 antibodies and counterstained with DAPI (nuclear) and MitoTracker (mitochondrial) labeling dyes, followed by quantitative imaging of 10,000 individual cells for each cell line (Fig. 1b and Supplementary Fig. S1b). A colocalization score higher or equal to (≥) 1 was used as a cut-off for strong nuclear localization^14^, based on the staining intensity range set by positive control histone H3 (nuclear) and negative control Tom20 (mitochondrial) (Supplementary Fig. S1c, d). Our findings indicate significant heterogeneity in the nuclear localization of ACO2 or IDH2 enzymes within a given cell line, ranging from ~ 84% to ~ 15% (Fig. 1c, d). Among the cell lines investigated, MDA-MB-468 (human triple negative breast cancer cell line) and GL261 (murine glioma cell line) depicted strong nuclear ACO2 and IDH2 staining (Fig. 1c, d), hence, we selected these two lines for additional analyses. Further, we performed high-resolution confocal microscopy with z-stack imaging that confirmed localization of CS, ACO2, and IDH2 in the nucleus (Fig. 1e, f and Supplementary Fig. S2a–d). Quantification of fluorescence pixel intensities at a selected XY plane obtained from the ‘z-stack’ showed CS, ACO2, and IDH2 signals (green peaks) overlapping with DAPI (blue), whereas the MitoTracker signals (red) were excluded from the nucleus. Our findings vividly demonstrate nuclear localization of these three TCA cycle enzymes (Fig. 1e, f and Supplementary Fig. S2e–h). Finally, we aimed to investigate the nuclear levels of CS, ACO2, and IDH2 in a setting devoid of all other cellular organelles. For this, we established a protocol to rapidly isolate intact nuclei while eliminating mitochondria, cytoplasm and other membrane bound organelles (Fig. 1g). The integrity of the isolated nucleus was verified by Lamin A/C immunostaining, confirming intact nuclear membrane, and the purity was authenticated by negative staining for Tom20, validating the absence of mitochondria (Fig. 1h and Supplementary Fig. S2i, j). Confocal microscopy of the isolated nuclei showed intense immunostaining signals for CS, ACO2, and IDH2 within the nuclear compartment. The staining pattern of the enzymes showed distinct ‘puncta-like or biomolecular condensates’ within the nucleus, which could be indicative of enzyme association with transcriptional hotspots or active transcription sites. In summary, a number of rigorous and unbiased approaches demonstrate reproducible localization of TCA cycle enzymes CS, ACO2, and IDH2 within the nucleus.

### Nuclear enzymes form a metabolic complex on the chromatin associated with histone proteins

**Fig. 2:**
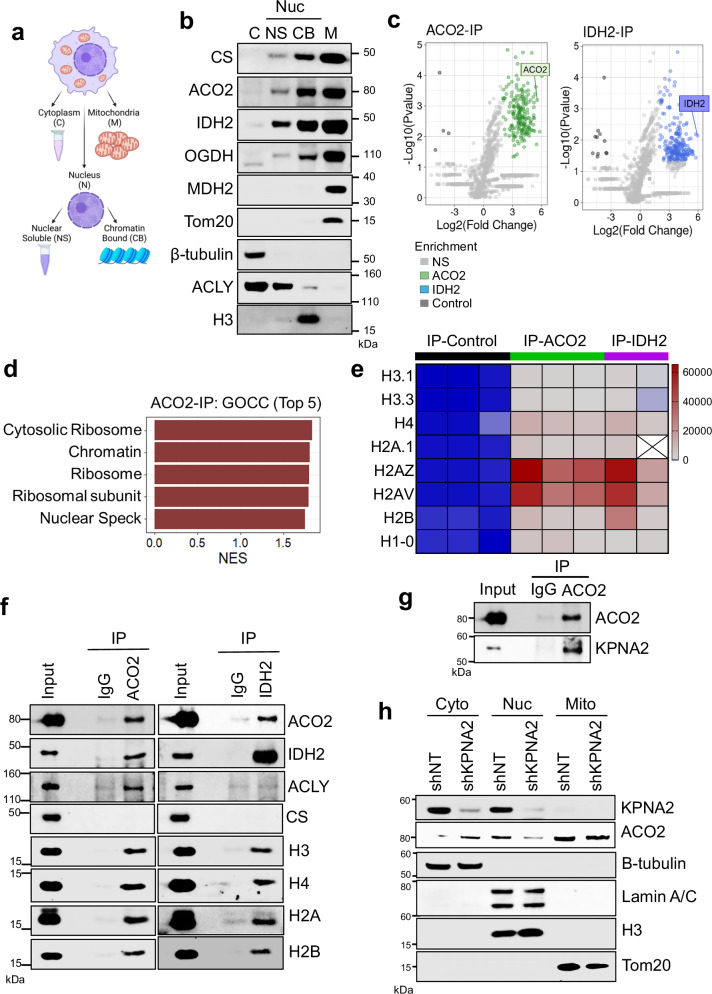
In the nucleus ACO2 and IDH2 enzymes are associated with chromatin and form a complex with histone proteins. **a** Schematics showing the experimental plan to isolate nuclear soluble (NS) and chromatin-bound (CB) fractions from the nucleus, along with cytosolic (C) and mitochondrial (M) proteins was created in BioRender. https://biorender.com/afwgk3c. **b** MDA-MB-468 cells were fractionated followed by immunoblot analysis of mitochondrial TCA cycle enzymes and ACLY. β-tubulin, histone H3, and Tom20 were used as cytosolic, chromatin-bound, and mitochondrial markers, respectively to confirm the purity of the fractions. (representative of 3 independent experiments). **c** ACO2 and IDH2 were immunoprecipitated (IP) from GL261 nuclear lysates, followed by mass-spectrometry (MS)-based proteomics (pre-clear control). The volcano plot shows significantly enriched proteins. (*n* = 3 independent samples per group) Two-tailed Student’s *t* test (Excel T.TEST, tails = 2, type = 3) was used *p* < 0.05 were highlighted. **d** Significantly enriched interactors from nuclear ACO2 pulldown (Log2 fold change > 2 and -log10(*p*-value) > 1.5 for immunoprecipitated ACO2 compared to pre-clear control) were analyzed by Gene Ontology Cellular Component (GOCC), and the top 5 pathways are shown (FDR *q* < 0.05). **e** Heatmap showing IP-MS data from (**c**), depicting the relative enrichment of histone subunits with nuclear ACO2 and IDH2 immunoprecipitation compared to control (pre-clear) samples from GL261 cells. Log2 fold change > 2 along with -log10(*p*-value) > 1.5. **f** ACO2, IDH2 and control (IgG) antibodies were used to perform immunoprecipitation from nuclear lysates of MDA-MB-468, followed by immunoblotting for metabolic enzymes and histone subunits. Input represents 1% of the total protein. (representative of three independent experiments). **g** ACO2-IP samples from (**f**) used for importin-alpha subunit 1 (KPNA2) immunoblotting. ACO2 IP/IB blot same as (**f**). IgG control. (representative of three independent experiments). **h** GL261 cells expressing non-targeting shRNAs (shNT) or shRNAs targeting KPNA2 (shKPNA2) were fractionated to isolate cytosolic (Cyto), nuclear (Nuc), and mitochondrial (Mito) proteins, and immunoblotting performed to measure KPNA2, ACO2, and IDH2 in the sub-cellular fractions. β-tubulin, Lamin A/C, and Tom20 were used as cytosolic, nuclear, and mitochondrial marker proteins. (representative of two independent experiments). Source data are provided as a Source Data file.

Next, we investigated the spatial distribution of the metabolic enzymes and their potential interactors in the nucleus to gain functional insights and mechanisms regulating nuclear entry. For this, nuclear extracts were further separated into nuclear soluble (NS) and chromatin bound (CB) fractions, using a nuclear membrane lysis buffer to extract nuclear soluble proteins, followed by a high-salt lysis buffer to extract chromatin-bound proteins (Fig. 2a). Our data indicated that CS, ACO2, and IDH2 are localized in both these nuclear fractions, but were enriched in the chromatin bound fraction (Fig. 2b and Supplementary Fig. S3a, b). In contrast, TCA cycle enzyme MDH2 was completely excluded from the nucleus, highlighting the specificity of CS, ACO2 and IDH2 chromatin association. We then immunoprecipitated (IP) endogenous ACO2 and IDH2 from the nuclear fraction, followed by a Liquid Chromatography-Mass Spectrometry (LC-MS) based proteomics analysis to identify potential interactors (Fig. 2c and Supplementary Fig. S3c). Gene Ontology analysis of the interactors indicated chromatin proteins were significantly enriched (FDR *q*-value = 0.045) (Fig. 2d). We found peptide abundance of several histone subunits including H3.1, H3.3, H4, H2AZ, H2AV, and H2B that were higher in both ACO2 and IDH2 immuno-precipitates compared to the controls (Fig. 2e). To validate the IP-MS data, we pulled down endogenous ACO2 and IDH2 from the nuclear lysates followed by immunoblotting with histone antibodies. Our data confirmed that nuclear ACO2 and IDH2 were associated with histone H3, H4, H2A, and H2B in both GL261 and MDA-MB-468 lines (Fig. 2f and Supplementary Fig. S3d, e). Moreover, we observed ACO2 and IDH2 interact with each other, but neither interacted with CS. Interestingly, ACO2 was identified to interact with ACLY (Fig. 2f), an enzyme known to generate acetyl-CoA in the nucleus^6^. These findings imply that nuclear ACO2 interacts with IDH2 and ACLY, but not CS, to form a metabolic complex on the chromatin associated with histone proteins. In addition, our IP-MS data identified strong enrichment of karyopherin subunit alpha 2 (KPNA2), also known as importin subunit alpha-1 (importin-α), with nuclear ACO2 (Supplementary Fig. S3f). KPNA2 binds to the nuclear localization sequence (NLS) of target proteins and traffics them into the nucleus^15^. Using the cNLS mapper algorithm, we found a bipartite NLS (score 4.2) at the N-terminus of ACO2 (Supplementary Fig. S3g, h). Immunoprecipitation of ACO2 from nuclear lysates followed by immunoblotting with KPNA2 antibody confirmed ACO2 binds to KPNA2 (Fig. 2g, Supplementary Fig. S3i). To investigate if KPNA2 facilitates nuclear localization of ACO2, we performed shRNA-mediated acute genetic silencing of KPNA2 followed by expression analysis of ACO2 in the cellular fractionations. We found a robust decrease in nuclear ACO2 levels with a concomitant increase of ACO2 in the cytosol upon KPNA2 depletion, whereas mitochondrial ACO2 remained unaffected (Fig. 2h). To identify possible upstream signals that could trigger the nuclear translocation process, we investigated several stress factors, among which serum starvation showed a marginal increase in ACO2 and IDH2 nuclear levels (Supplementary Fig. S3j). These findings suggest ACO2 translocation to the nucleus is dependent on importin-α, whereas IDH2 relies on an independent mechanism, and cellular stress signals could potentially instigate their nuclear import (Supplementary Fig. S3k).

### Chromatin-bound metabolic complex associates with KAT2A to coordinate histone acetylation

**Fig. 3:**
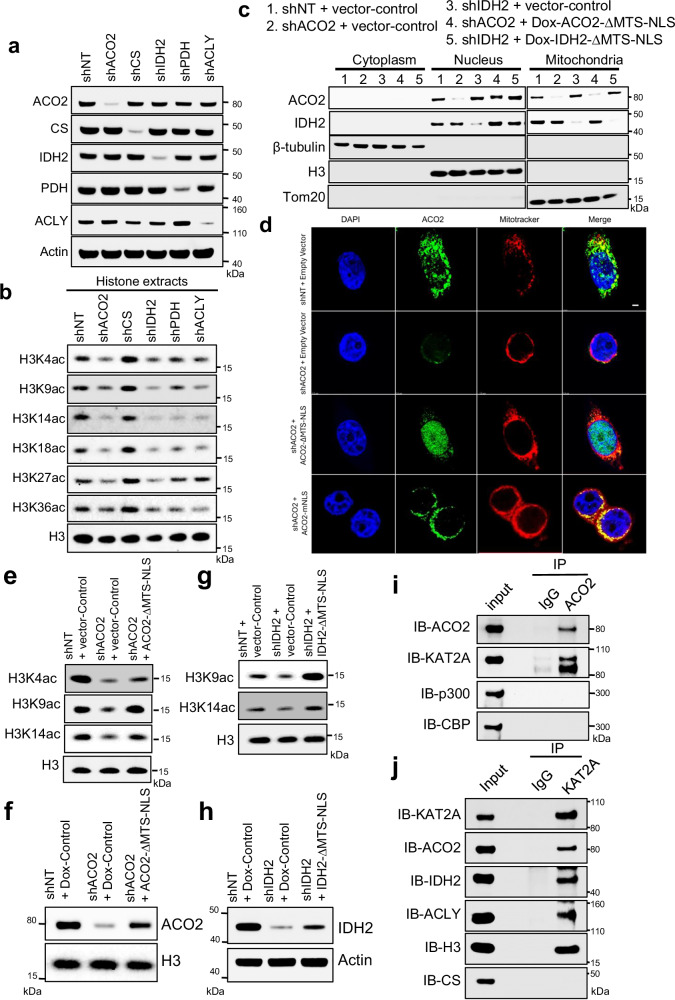
Metabolic enzymes in the nucleus control acetylation of histones by associating with KAT2A. **a** Immunoblots showing knockdown of shACO2, shCS, shIDH2, shPDH, and shACLY compared to control shNT in MDA-MB-468 cells and actin was used as a loading control. **b** Acid extracted histones from MDA-MB-468 cells with stable expression of shNT (control), shRNAs targeting ACO2 (shACO2), CS (shCS), IDH2 (shIDH2), PDH (shPDH), and ACLY (shACLY) were used for immunoblotting to measure H3 acetylation marks. Total histone H3 was used as a loading control. **c** Engineered doxycycline-inducible constructs (ACO2-∆MTS-NLS, and IDH2-∆MTS-NLS) expressing nuclear localization (NLS) tagged ACO2 or IDH2 with a deleted mitochondrial transition sequence (∆MTS) were transduced in ACO2 (shACO2) and IDH2 (shIDH2) depleted MDA-MB-468 cells, respectively and an empty vector (doxycycline-inducible) was used as a vector-control. Cellular fractions used for ACO2 and IDH2 immunoblotting compared to controls. β-tubulin, Lamin A/C, and Tom20 were used to check fraction purity. Doxycycline, 1 µg/mL. **d** MDA-MB-468 cells expressing shNT (control), shACO2, shACO2 cells with ACO2-∆MTS-NLS construct, or shACO2 cells with mutated-ACO2-nuclear localization signal (ACO2-mNLS) was used for immunofluorescence confocal microscopy to determine the cellular localization of ACO2 (green) counterstained with nucleus (DAPI, blue), and mitochondria (mitotracker, red). Scale bar 10 µm. (representative fields from 3 independent experiments). **e**–**h** Acid extracted histones isolated from MDA-MB-468 cells with stable expression of shACO2 or transduced with ACO2-∆MTS-NLS (**e**–**f**)**;** and shIDH2 or transduced with IDH2-∆MTS-NLS (**g**,** h**) were used for immunoblotting with H3K4ac, H3K9ac and H3K14ac, compared to controls (shNT + vector-control). Total histone H3 was used as a loading control. Doxycycline, 1 µg/mL. **i** Nuclear lysates from MDA-MB-468 cells were used to immunoprecipitate (IP) endogenous ACO2, followed by immunoblotting analysis with histone acetyl transferase (HAT) enzymes: KAT2A, p300, and CBP. Input represents 0.5% of the total protein used for immunoprecipitation. **j** Endogenous KAT2A was immunoprecipitated (IP) from MDA-MB-468 nuclear lysates, followed by immunoblotting analysis with ACO2, IDH2, ACLY, CS, and H3, compared to control (IgG). Input represents 0.5% of the total protein. (**a**–**j**) are representative data from 3 independent experiments for each line. Source data are provided as a Source Data file.

Metabolic reactions catalyzed by CS, ACO2, and IDH2 modulate mitochondrial citrate levels, which is then exported to the cytosol and converted to acetyl-CoA by ACLY^4^. Since our findings indicated CS, ACO2 and IDH2 are bound to chromatin, we investigated whether they control histone acetylation levels. We performed genetic depletion of the enzymes and generated stable knockdown lines (Fig. 3a and Supplementary Fig.S4a). Recent studies have shown ACLY and PDH enzymes can modulate histone acetylation marks^6,16^, so we included them as positive controls. Immunoblot analysis of the acid-extracted histones from these stable lines revealed that ACO2 and IDH2 ablation significantly decreased several histone H3 acetylation marks, among which H3K9ac, H3K14ac, and H3K18ac showed consistent reduction in most of the lines investigated (Fig. 3b, Supplementary Fig. S4–d). Surprisingly, depletion of CS increased some of the H3 acetylation marks, suggesting nuclear CS enzyme may reduce histone acetylation. CS was not part of the nuclear metabolic complex and failed to interact with histone proteins, so we specifically focused on investigating whether alterations in the histone acetylation levels were mediated by nuclear ACO2 and IDH2. For this, we generated doxycycline inducible mutant constructs of ACO2 and IDH2 to express the enzymes preferentially in the nucleus. We deleted the endogenous mitochondrial transition sequence (∆MTS) to block their canonical mitochondrial localization, followed by addition of a strong nuclear localization sequence (NLS) on the N-terminus (Supplementary Fig. S4e). We generated stable cell lines expressing ACO2-∆MTS-NLS (Supplementary Fig. S4f, g) or IDH2-∆MTS-NLS in ACO2 or IDH2 depleted cells, respectively. Immunoblot analysis of cellular fractions confirmed nuclear specific rescue of ACO2 and IDH2 by the mutant constructs upon doxycycline induction, but not in the mitochondria (Fig. 3c). In addition, we mutated the Arg (R) and Lys (K) residues to Ala (A) within the ‘predicted bipartite NLS-domain’ identified in ACO2 (Supplementary Fig. S3g, h) and re-expressed the mutated-NLS-ACO2 (mNLS-ACO2) in ACO2-depleted stable cells (Supplementary Fig. S4h). Cellular fractionation and confocal microscopy revealed that mNLS-ACO2 failed to localize to the nucleus, whereas the mitochondrial translocation was not compromised (Fig. 3d and Supplementary Fig. S4i). These domain-specific mutations confirmed that the nuclear-specific localization of ACO2 is tightly controlled by the endogenous bipartite NLS. Immunoblot analysis of the acid-extracted histone revealed nuclear-specific re-expression of ACO2 or IDH2 was sufficient to rescue H3K9ac and H3K14ac marks that were lost in ACO2 or IDH2 depleted cells (Fig. 3e–h). These findings indicate that nuclear ACO2 and IDH2 may fuel histone acetylation, supported by the inability of mNLS-ACO2 to restore the H3Ac marks (Supplementary Fig. S4j), independent of mitochondrial functions. Next, we investigated any potential HAT enzymes that may preferentially work in concert with ACO2 and IDH2 to acetylate histones. Among the known HATs, our immunoprecipitation data identified nuclear ACO2 has strong interaction with KAT2A but not with p300 or CBP proteins (Fig. 3i and Supplementary Fig. S5a). Reciprocal immunoprecipitation of endogenous KAT2A also demonstrated interaction with all three metabolic enzymes ACO2, IDH2 and ACLY in the nucleus, but not with CS (Fig. 3j and Supplementary Fig. S5b). Collectively, these results suggest that the chromatin bound nuclear metabolic complex of ACO2, IDH2 and ACLY may specifically be associated with KAT2A to coordinate acetylation of histone H3 tails (Supplementary Fig. S5c).

### ACO2 and IDH2 catalyze reductive carboxylation of α-KG in the nucleus to fuel histone acetylation

**Fig. 4:**
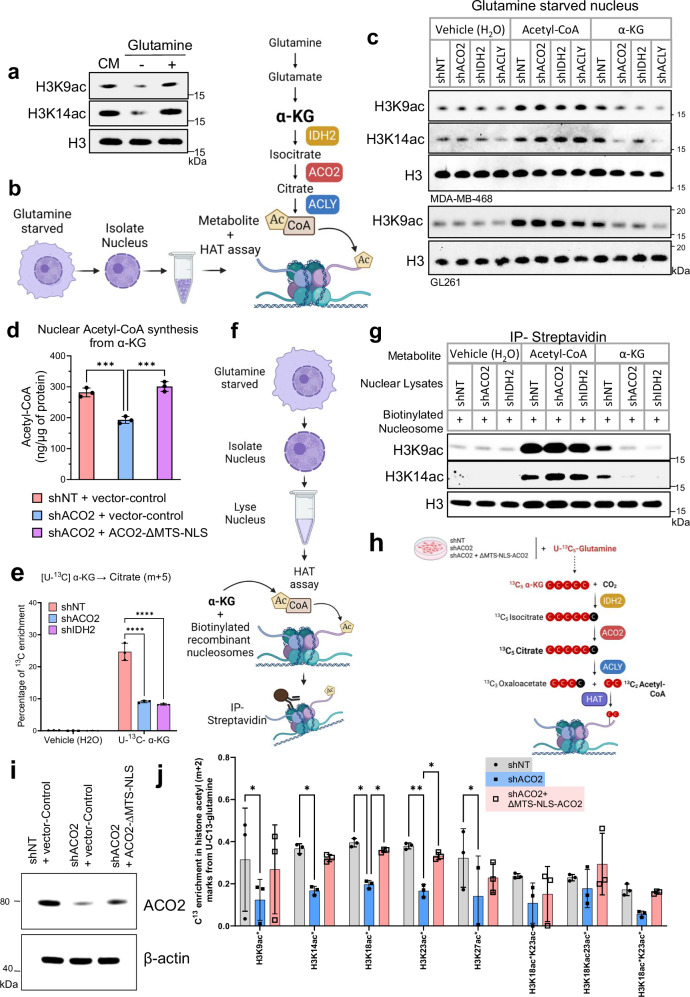
ACO2 and IDH2 mediated reductive carboxylation of alpha-ketoglutarate (αKG) synthesize citrate in the nucleus that generates nuclear acetyl-CoA pool for histone acetylation. **a** Immunoblot showing H3K9ac and H3K14ac in GL261 cells cultured in complete medium (CM), glutamine starved (-glutamine) or replenished ( + glutamine). Histone H3 was used as a loading control (representative of 3 independent experiments). **b** Schematic showing in vitro histone acetyl transferase (HAT) assay, created in BioRender. https://biorender.com/apkbyg2. **c** Immunoblot analysis of H3K9ac and H3K14ac marks following in vitro HAT assay (**b**) in MDA-MB-468 and GL261, histone H3 was a loading control. (representative of 3 independent experiments). **d** Concentration of acetyl-CoA in the nucleus of MDA-MB-468 shNT + vector-control, shACO2 + vector-control, and shACO2 + ACO2-∆MTS-NLS following HAT assay with α-KG (2 mM), normalized to total nuclear protein. Doxycycline, 1 µg/mL. (*n* = 3 independent samples per group). One-way ANOVA with Tukey’s multiple comparisons test comparing shACO2 + vector-control with: shNT + vector-control *** *P* = 0.0005; and shACO2 + ACO2-∆MTS-NLS *** *P *= 0.0002. Error bars represent mean ± SD. **e** Relative enrichment of C^13^ isotopes in citrate (m + 5) after incubation of isolated nuclei from MDA-MB-468 with U-^13^C_5_-α-KG (2 mM) in HAT assay. Two-way ANOVA with Tukey’s multiple comparisons test comparing shNT with: shACO2 **** *P* < 0.0001; and shIDH2 **** *P* < 0.0001. Error bars are presented as mean ± SD. (*n* = 3 independent samples per group). **f** Schematic showing HAT assay using recombinant nucleosomes, created in BioRender. https://biorender.com/olllyzl. **g** Immunoblot showing H3K9ac and H3K14ac levels in MDA-MB-468 cells expressing shNT (control), shACO2, and shIDH2, histone H3 was a loading control. (representative of two independent experiments). **h** Schematics showing metabolite tracing to detect ^13^C-isotopes on histone marks, created in BioRender. https://biorender.com/lz7xg0x. **i** Immunoblot showing ACO2 levels in shNT, shACO2, and shACO2 with ACO2-∆MTS-NLS expression in MDA-MB-468. **j** Fractional enrichment of acetyl C^13^ (m + 2) on H3Ac marks in MDA-MB-468 from U-^13^C_5_-Glutamine. Error bars are presented as mean ± SD. Two-way ANOVA with Fisher’s multiple comparisons test comparing shNT with shACO2: H3K9ac, *P *= 0.0189; H3K14ac, *P =* 0.0145; H3K18ac, *P* = 0.0152; H3K23ac, *P* = 0.0093; H3K27ac, *P* = 0.0441; and shACO2 v shACO2 + ACO2-∆MTS-NLS: H3K18ac, *P *= 0.0469; H3K23ac, *P *= 0.0405. (*n* = 3 independent samples per group). Source data are provided as a Source Data file.

Reversible reactions of IDH2 and ACO2 catalyze reductive carboxylation of glutamine-derived α-KG to generate citrate, which is then converted to acetyl-CoA by ACLY^17–19^. Thus, we interrogated whether chromatin bound nuclear metabolic complex of IDH2, ACO2, and ACLY utilize glutamine derived α-KG to generate acetyl groups for histone acetylation. Starving the cells without glutamine reduced the levels of H3K9ac and H3K14ac, and replenishing glutamine in the culture medium efficiently restored the histone acetylation marks (Fig. 4a). To determine whether glutamine derived α-KG is required for acetylation of histones in the nucleus, we used rapidly isolated intact nuclei, that are devoid of cytosolic and mitochondrial proteins, for a cell-free in-vitro HAT assay reliant on the endogenous HAT-enzymes present in the isolated nuclei. Isolated intact nuclei from glutamine-starved cells, were incubated with intermediate metabolites of glutamine catabolism pathway, to test whether these metabolites could rescue histone acetylation marks in an in-vitro HAT assay (Fig. 4b). Supplementing acetyl-CoA, citrate, or α-KG efficiently restored H3K9ac and H3K14ac levels compared to vehicle control (H_2_O), suggesting these metabolites could be utilized to generate donor acetyl groups in the nucleus (Supplementary Fig. S6a). We isolated intact nuclei from glutamine starved cells and treated them with vehicle control (H2O), citrate, α-KG, and glutamine and identified the addition of α-KG showed increased levels of acetyl-CoA in the nucleus compared to vehicle control (H_2_O), citrate and glutamine (Supplementary Fig. S6b). Next, we performed HAT assay utilizing isolated nuclei from ACO2, IDH2, or ACLY depleted, and control (shNT) cells cultured in glutamine-starved conditions (Supplementary Fig. S6c). The addition of acetyl-CoA increased H3K9ac and H3K14ac levels in all the lines irrespective of genetic inhibition of the enzymes, indicating HAT enzymes were functionally active in our assay conditions and only acetyl-CoA was limited in the isolated nucleus (Fig. 4c). However, the addition of α-KG failed to restore histone acetylation levels in IDH2, ACO2, or ACLY depleted nucleus, whereas the control (shNT) nuclei demonstrated profound histone acetylation (Fig. 4c). At the end of HAT assay metabolite measurements confirmed that acetyl-CoA levels were significantly elevated in the isolated nucleus supplemented with α-KG compared to vehicle (Supplementary Fig. S6d), however, loss of ACO2 significantly reduced nuclear acetyl-CoA levels, which were efficiently restored by nuclear specific expression of ACO2-∆MTS-NLS construct (Fig. 4d). These data imply that the nuclear localized IDH2, ACO2, and ACLY are obligatory for α-KG dependent acetyl-CoA synthesis required for acetylation of histones.

Next, we performed metabolic tracing experiments to confirm IDH2 and ACO2 catalyze the reversible reactions converting α-KG into citrate required for acetyl CoA generation in the nucleus. We incubated the isolated intact nucleus from IDH2 or ACO2- depleted, and control (shNT) cells with uniformly labeled ^13^C α-KG (U-^13^C α-KG) under HAT-assay conditions. LC-MS analysis of the metabolites from the isolated nucleus revealed strong incorporation of ^13^C from αKG into citrate (m + 5, *m* represents nominal mass) isotopomers in the control (shNT) cells, whereas loss of IDH2 or ACO2 significantly reduced the percentage of ^13^C incorporation in citrate m + 5 levels (Fig. 4e and Supplementary Fig. S6e–i). Synthesis of citrate (6 carbons) from αKG (5 carbons) requires the addition of an unlabeled carbon by the reductive carboxylation process, generating citrate (m + 5) catalyzed by the reversible enzymes IDH2 and ACO2^17,18,20^. Hence, our metabolic tracing experiments confirmed that in the nucleus, IDH2 and ACO2 produce citrate by reductive carboxylation of αKG, which is then used by ACLY to produce acetyl-CoA for histone acetylation. To further confirm our observations, we performed in-vitro HAT assay with nuclear lysates from control (shNT), ACO2 depleted or IDH2 depleted cells that were incubated with unlabeled biotinylated recombinant mononucleosomes along with α-KG (Fig. 4f). Immunoprecipitation of the biotinylated nucleosomes after the HAT assay followed by immunoblotting, revealed robust acetylation of H3K9 and H3K14 residues, only when control nuclear lysates were incubated in presence of α-KG, but the effect was completely abrogated in case of ACO2 or IDH2 depleted nuclear lysates (Fig. 4g and Supplementary Fig. S6j, k). These findings confirmed that nuclear ACO2 and IDH2 mediated metabolic functions are essential for histone acetylation. Finally, we performed metabolic tracing experiments to confirm that α-KG derived from glutamine metabolism are major carbon source used by nuclear ACO2 and IDH2 to drive histone acetylation marks (Fig. 4h). For this, we incubated glutamine starved shNT, shACO2 and shACO2 + ACO2-∆MTS-NLS cells (Fig. 4i) with U-^13^C-glutamine followed by unbiased proteomics profiling of histone modifications to measure enrichment of acetyl (m + 2) marks. Our data revealed that approximately 30–40% of H3Ac marks were enriched with ^13^C from glutamine, and loss of ACO2 significantly reduced H3K9Ac, H3K14Ac, H3K18ac, H3K23ac and H3K27ac marks. Moreover, restoring the levels of ACO2 in the nucleus using dox-inducible expression of ACO2-∆MTS-NLS rescued most of the H3Ac marks that were significantly decreased due to loss of ACO2, confirming that nuclear metabolic functions of ACO2, but not mitochondrial, is essential to drive histone acetylation (Fig. 4j). Together, these data support the notion that nuclear metabolic enzymes IDH2 and ACO2 dependent reductive carboxylation of α-KG is a major carbon source for acetyl-CoA production essential for acetylation of histones.

### Nuclear metabolic complex enhances chromatin accessibility to induce transcription of specific genes

**Fig. 5:**
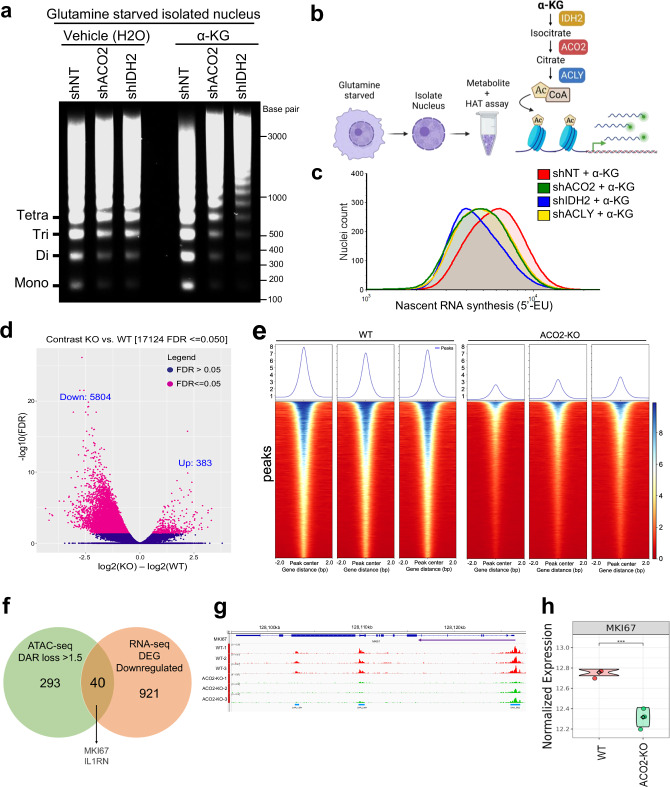
Utilization of α-KG by IDH2 and ACO2 in the nucleus increases chromatin accessibility to induce nascent RNA synthesis. **a** Relative levels of mono-, di-, tri- and tetra- nucleosome fragments following MNase digestion assay using intact nuclei isolated from glutamine-starved GL261 cells incubated with vehicle control (H2O) or α-KG (2 mM) in HAT assay (representative of 3 independent experiments). **b** Schematic showing HAT assay coupled to nascent-RNA synthesis and detection was created in BioRender. https://biorender.com/cyux48c. **c** Intensity plot showing nascent-RNA synthesis rates in nuclei isolated from GL261 shNT, shACO2, shIDH2, and shACLY treated with α-KG (2 mM) and 5-Ethynyl Uridine (EU) as described in (**b**). (*n* = 25,000 individual nuclei per sample per group, representative of 2 independent experiments). **d** Volcano plot showing the differential accessible regions (DAR) (FDR ≤ 0.05) that were open (up) or closed (down) in ACO2-KO compared to WT (control) ATAC-seq (GSE301090), C4-2 cells. (*n* = 3 independent samples per group). **e** Tornado plot depicting the DARs obtained from ATAC-seq (GSE301090) centered at peak regions that were lost (number of peaks lost =  16,626) due to ACO2 depletion (ACO2-KO) compared to WT (control) C4-2 cells. **f** Venn diagram showing the common target genes (40) identified by integrating ATAC-seq (GSE301090) (*n* = 3 independent samples per group) and RNA-seq (GSE301089) (WT: *n* = 2, ACO2-KO: *n* = 3 independent samples) data obtained from C4-2 WT and ACO2-KO samples. A total of 333 genes (367 regions) were lost in ACO2-KO compared to WT (loss > 1.5 within 5 kb of promoter), whereas RNA-seq identified a total of 961 DEG, using p.adj < 0.05 in ACO2-KO compared to WT. **g** Integrative Genomics Viewer (IGV) based visualization of ATAC-seq (GSE301090) peaks in C4-2 WT (red) vs ACO2-KO (green) at MKI67 genomic loci. **h** Normalized expression levels of MKI67 gene in C4-2 WT vs ACO2-KO from RNAseq (GSE301089) analysis. ***p < 0.001 using Student’s *t* test, two-tailed. Source data are provided as a Source Data file.

To investigate the impact of IDH2 and ACO2 mediated acetylation of histone H3 marks on the chromatin state, we performed an MNase digestion assay to assess the chromatin accessibility. The data indicated that the levels of mono-, di- and tri-nucleosome DNA fragments were significantly reduced upon IDH2 and ACO2 depletion implying a more condensed chromatin state, compared to control (shNT) (Supplementary Fig. S7a, b). Interestingly, α-KG supplementation significantly increased the shorter nucleosome DNA fragments, whereas loss of ACO2 or IDH2 abrogated the effect (Fig. 5a and Supplementary Fig. S7c). α-KG is also known to be required for histone and DNA demethylation levels^21^, however, loss of IDH2 or ACO2 had minimal effects on histone methylation marks (Supplementary Fig. S7d). This observation suggests that reduced chromatin accessibility in IDH2 or ACO2-depleted nuclei is caused by their inability to utilize α-KG for histone acetylation. Next, to determine the effects of altered chromatin accessibility on gene transcription, we quantified the rate of nascent RNA synthesis in IDH2 and ACO2-depleted nuclei compared to the control (shNT). Isolated nuclei from glutamine-starved cells were treated with α-KG, along with NTPs and 5’-Ethynyl Uridine (EU) to label nascent RNA. After the completion of *vitro* assay, the nuclei were fixed and analyzed by flow cytometry to quantify the 5’EU incorporation in the newly synthesized RNA (Fig. 5b). We found supplementation of α-KG in nuclei isolated from shNT (control) cells, had higher production of 5’EU labeled nascent RNA compared to nuclei with depleted levels of IDH2, ACO2, or ACLY (Fig. 5c and Supplementary Fig. 7e). Based on these findings, we conclude that utilization of α-KG by nuclear IDH2 and ACO2 to acetylate histone H3 tails is essential for chromatin relaxation and induction of gene transcription.

To determine the impact of nuclear localized metabolic enzymes on chromatin accessibility and gene transcription, we performed an assay for transposase-accessible chromatin using sequencing (ATAC-seq) and integrated the data with transcriptome profiles obtained from RNAseq. Depletion of ACO2 (ACO2-KO) (Supplementary Fig. S7f) resulted in an altered chromatin profile compared to control (WT) (Supplementary Fig. S7g) and analysis of the differentially accessible regions (DAR) revealed closing of 5804 regions with only 383 regions being newly opened (Fig. 5d). These findings indicate that loss of ACO2 caused an overall closed chromatin state due to a significant loss of chromatin accessible regions (Fig. 5e). RNAseq analysis also showed an altered transcriptomic profile upon ACO2 depletion compared to WT (Supplementary Fig. S7h–j). Gene set enrichment analysis (GSEA) of the differentially expressed genes (DEG) and significantly closed DARs due to ACO2 ablation showed alteration in metabolic signatures essential for cellular responses to starvation (Supplementary Fig. S8a, b). Integrating ATAC-seq DARs and RNA-seq DEGs, we identified 40 overlapping affected genes, among which were MKI67 (cell cycle and proliferation marker) and IL1RN (Interleukin 1 Receptor Antagonist) that were both found to be significantly repressed upon ACO2 depletion (Fig. 5f–h and Supplementary Fig. S8c–e). Moreover, ACO2-∆MTS-NLS re-expression rescued several chromatin accessible regions that were lost due to ACO2-depletion, confirming the importance of nuclear ACO2 functions (Supplementary Fig. S8f). Since KAT2A was the only HAT enzyme found to be associated with nuclear ACO2, we integrated the ACO2-associated ATAC-seq DARs with KAT2A ChIP-seq data^22^. GSEA analysis of the top 200 overlapping targets identified enrichment for Fisher DREAM targets, which is a master coordinator of cell cycle-dependent genes (Supplementary Fig. S8g)^23^. Overall, these findings imply that nuclear metabolic enzymes mediate acetylation of histone H3 by KAT2A, and this histone H3 acetylation increases chromatin accessibility, resulting to drive expression of genes involved in cell survival and adaptation.

### Chromatin accessibility created by nuclear metabolic enzymes enrich FOXA1 to induce proliferative phenotypes

**Fig. 6:**
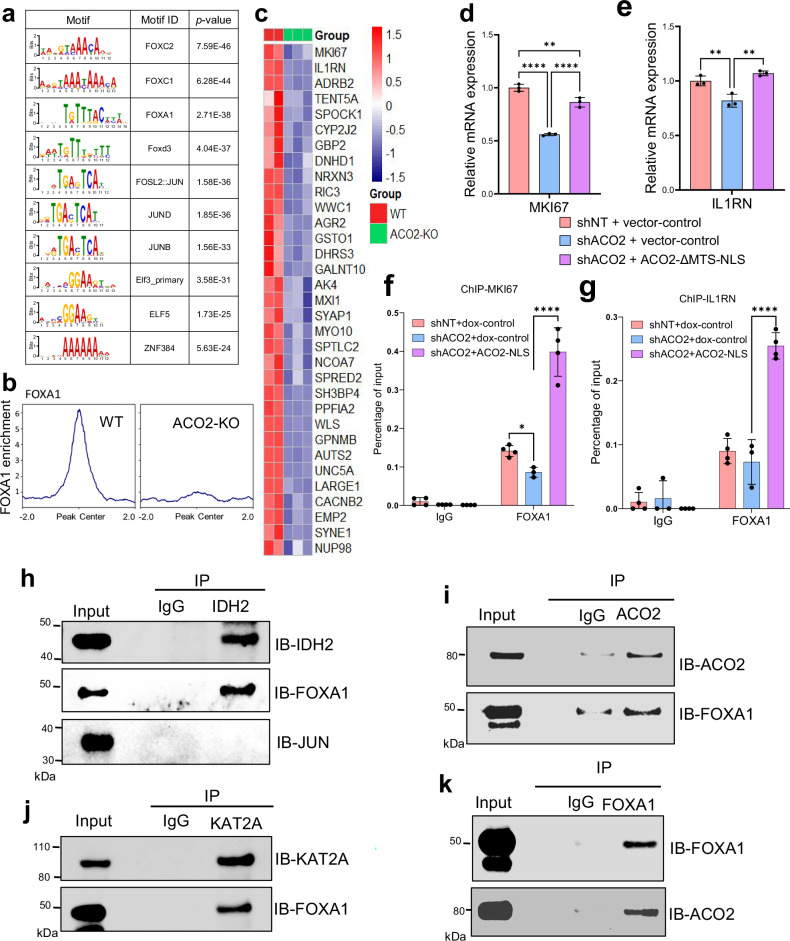
Increased chromatin accessibility by nuclear metabolic enzymes preferentially recruits FOXA1 to induce proliferative phenotype. **a** Motif enrichment analysis showing transcription factors that were significantly altered, due to the loss of DARs in ACO2-KO compared to WT (control) C4-2 cells, found through ATAC sequencing (GSE301090).* n* = 3 independent samples per group. **b** Overall ATAC-seq (GSE301090) peak intensity at FOXA1 motif sites, centered at peak regions, in ACO2-WT and ACO2-KO cells. (WT: *n* = 2, ACO2-KO: *n* = 3 independent samples). **c** Heatmap depicting normalized expression levels of ATAC-seq (GSE301090) and RNA-seq (GSE301089) overlapping target gene signature with FOXA1 motif site. This is defined as ATAC-seq DAR loss > 1.5 within 5 kb of promoter, along with decreased mRNA expression levels using p.adj < 0.05 in ACO2-KO compared to WT, that also contain FOXA1 motif site. *n* = 2 (WT), *n *= 3 (ACO2-KO). **d**,** e** Relative mRNA expression of MKI67 (**d**) and IL1RN (**e**) in MDA-MB-468 cells (*n* = 3 independent samples). Doxycycline, 1 µg/mL. Error bars are presented as mean ± SD. Two-way ANOVA with Tukey’s multiple comparisons test was used to compare shNT group with shACO2: MKI67, ****P < 0.0001; IL1RN, *P* = 0.0062; and between shACO2 and shACO2 + ACO2-∆MTS-NLS groups: MKI67, ****P < 0.0001; IL1RN, *P* = 0.0012. *n* = 3 independent samples per group. **f**,** g** Percentage of input of FOXA1 occupancy at MKI67 (**f**) and IL1RN (**g**) proximal promoter regions normalized to IgG (control) in MDA-MB-468 cells. Doxycycline, 1 µg/mL, (*n* = 4, independent samples). Error bars are presented as mean ± SD. Two-way ANOVA with Tukey’s multiple comparisons test was used to compare FOXA1 occupancy in the shNT group with shACO2: MKI67, *P* = 0.0456; IL1RN, *P* = 0.5546; and between shACO2 and shACO2 + ACO2-∆MTS-NLS groups: MKI67, ****P < 0.0001; IL1RN, ****P < 0.0001. *n* = 3 independent samples per group. **h**–**k** Nuclear lysates isolated from MDA-MB-468 were used for immunoprecipitation (IP) of (**h**), IDH2 followed by immunoblotting for FOXA1 and c-JUN; (**i**) ACO2, followed by immunoblotting for FOXA1; (**j**) KAT2A, followed by immunoblotting for FOXA1; (**k**) FOXA1, followed by immunoblotting for ACO2. Isotype-controlled IgG was used as a negative control, and 0.5% input used for the IP. Representative of 2 independent experiments. Source data are provided as a Source Data file.

Although the impact of the nuclear metabolic pathway appears to increase global histone acetylation levels and chromatin accessibility, motif enrichment analysis of the DARs identified from ATAC sequencing revealed significant enrichment of the motif bound by the pioneering factor FOXA1 (Fig. 6a, b). Using GIGGLE, we queried specific DARs that were altered by ACO2 depletion against the CistromeDB database, which is an experimentally derived database for > 10,000 ChIP-seq datasets across > 1100 transcription factors^24^. From GIGGLE analysis, we observed FOXA1 to be the most enriched transcription factor, overlapping with the DARs that get lost upon ACO2 depletion (Supplementary Fig. S9a, b). Interestingly, 35 out of the 40 overlapping target genes identified by integrating ATAC-seq and RNA-seq datasets, including MKI67 and IL1RN, had a FOXA1 binding site (Fig. 6c). ACO2 depletion significantly decreased MKI67 and IL1RN expression compared to control (shNT), whereas nuclear-specific rescue of ACO2 by ACO2-∆MTS-NLS construct was sufficient to restore endogenous MKI67 and IL1RN levels (Fig. 6d, e). Next, we analyzed FOXA1 occupancy on the proximal promoters of MKI67 and IL1RN under conditions of loss and gain-in-nuclear ACO2 expression. ChIP-qPCR confirmed significantly increased enrichment of FOXA1 on MKI67 and IL1RN gene promoters in nuclear-specific ACO2 expressing cells, compared to ACO2-depleted or control (shNT) cells (Fig. 6f, g). To determine whether FOXA1 is associated with the chromatin regulatory complex containing IDH2-ACO2-KAT2A, we performed immunoprecipitation (IP) followed by immunoblotting (IB). Our data revealed that IDH2 (Fig. 6h), ACO2 (Fig. 6i), and KAT2A (Fig. 6j) are bound to FOXA1 (Fig. 6k), but not with another pioneering factor, cJUN, observed in our analysis (Fig. 6a). Although further analysis of genome-wide co-occupancy of these chromatin regulators and metabolic enzymes are needed, our findings provide evidence that pioneering factor FOXA1 could associate with metabolic enzymes ACO2 and IDH2 to mediate histone acetylation and gene activation.

### Nuclear metabolic enzymes ACO2 and IDH2 drive aggressive proliferative tumors in vivo

**Fig. 7:**
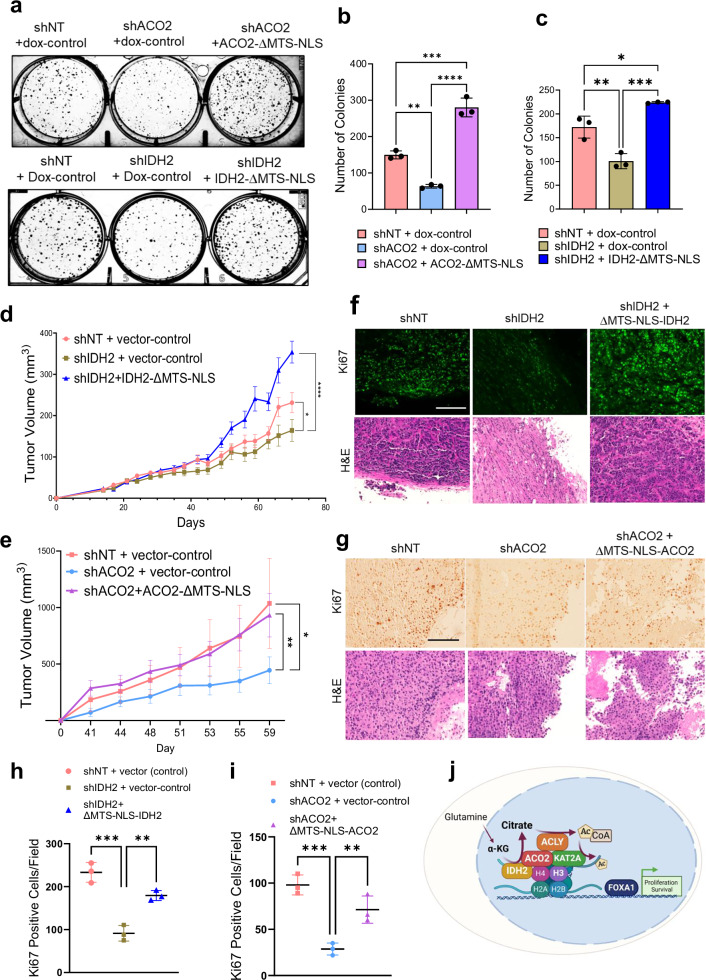
Nuclear ACO2 and IDH2 drive aggressive tumor progression in mouse models of cancer. **a** Colony assay in MDA-MB-468 cells showing effects of ACO2 and IDH2 nuclear rescue with ∆MTS-NLS constructs. Doxycycline 1 µg/mL. Representative of 3 independent experiments. **b**,** c** Quantification of the number of colonies obtained in Fig. 7a, (**b**) rescue with ACO2-∆MTS-NLS in shACO2 cells; (**c**) rescue with IDH2-∆MTS-NLS in shIDH2 cells; Doxycycline 1 µg/mL. *n* = 3, independent samples per group. Error bars are presented as mean ± SD. One-way ANOVA with Tukey’s multiple comparisons test to compare shNT with: shACO2, *P* = 0.0016; shACO2 + ACO2-∆MTS-NLS: *P* = 0.0002; shIDH2: *P* = 0.004; shIDH2 + IDH2-∆MTS-NLS: *P* = 0.0187; shACO2 with shACO2 + ACO2-∆MTS-NLS: **** *P* ≤ 0.0001; and shIDH2 with shIDH2 + IDH2-∆MTS-NLS: *P* = 0.0002. **d** Orthotopic breast tumor growth rate of MDA-MB-468 cells expressing shNT+vector-control (*n* = 10), shIDH2+vector-control (*n* = 9), or shIDH2 + IDH2-∆MTS-NLS *(n* = *9)*. Doxycycline (2000mg/kg) was added to the chow. Non-parametric Friedman test (two-tailed) with Dunn’s multiple comparisons test was performed to compare shNT with: shIDH2, *P* = 0.0138; and shIDH2 with shIDH2 + IDH2-∆MTS-NLS, **** *P* < 0.0001. Error bars are presented as mean ± SEM. **e** Subcutaneous tumor growth rate of C4-2 cells expressing shNT+vector-control, shACO2 + vector-control, and shACO2 + ACO2-∆MTS-NLS (*n* = 9 mice/group). Doxycycline (2000mg/kg) was added to the chow. Non-parametric Friedman test (two-tailed) with Dunn’s multiple comparisons to compare shNT with: shACO2, *P* = 0.0489; and shACO2 with shACO2 + ACO2-∆MTS-NLS, *P* = 0.0054. Error bars are presented as mean ± SEM. **f**,** g** H&E and Ki67 immunohistochemistry performed with tumor tissues (representative of 3 independent samples per group) isolated from panel Fig. 7d, e. Scale bar 150 µm. **h**,** i** Quantification of Ki67 positive cells per microscopy field from Fig.7f, g. (*n* = 3 independent samples per group) One-way ANOVA with Tukey’s multiple comparisons test to compare shNT with shIDH2: *P* = 0002; shIDH2 with shIDH2 + IDH2-∆MTS-NLS: *P* = 0.0026; shNT with shACO2, *P* = 0.0007; and shACO2 with shACO2 + ACO2-∆MTS-NLS, *P* = 0.0082. Error bars are presented as mean ± SD. **j** Proposed model of nuclear metabolic complex (NMC) consisting of ACO2, IDH2 and ACLY associated with histones and catalyze reductive carboxylation of α-KG to generate citrate for nuclear acetyl-CoA. NMC binds to KAT2A to acetylate histones favoring FOXA1-dependent gene expression and tumor growth. Created in BioRender.https://biorender.com/6k1yof7. Source data are provided as a Source Data file.

Since MKI67 is a cellular proliferation marker, we next assessed the impact of nuclear localized metabolic enzymes on cell proliferation and survival. Interestingly, nuclear-specific rescue of IDH2 or ACO2 significantly increased colony formation and proliferative capacity of MDA-MB-468 cells (Fig. 7a–c and Supplementary Fig. S9c). To study the impact of this nuclear localized metabolic pathway on in vivo tumor growth and proliferation, we stably expressed exogenous ACO2-∆MTS-NLS in ACO2-depleted C4-2 cells. Engineered cells were subcutaneous implanted into male NSG mice to assess tumorigenicity. Similarly, IDH2-depleted MDA-MB-468 cells stably expressing IDH2-∆MTS-NLS or control vector were implanted orthotopically into the mammary fat pad of female NSG mice. Multiple models were used to enhance scientific rigor and assess sex as a biological variable. In both mouse models of cancer, we found nuclear IDH2, or ACO2 expression (Supplementary Fig. S9d, e) rapidly accelerated tumor progression compared to the IDH2 (Fig. 7d and Supplementary Fig. S9f) or ACO2 depleted tumors (Fig. 7e and Supplementary Fig. S9g). Specifically, breast tumors with nuclear IDH2 expression were significantly greater in tumor volume than the shNT (control), implying a hyperproliferative phenotype, corroborated with nuclear localized metabolic enzymes. An immunohistochemical analysis of the tumors confirmed increased MKI67 (Ki67) staining in tumors with nuclear IDH2 and ACO2 (Fig. 7f–i). Collectively, these results highlight the importance of nuclear-specific metabolic pathways consisting of IDH2 and ACO2 that epigenetically regulate chromatin accessibility to induce a proliferative phenotype by recruiting pioneering factor FOXA1 (Fig. 7j).

## Discussion

Here, we discover a nuclear-localized metabolic pathway responsible for reductive carboxylation of α-KG to provide citrate and acetyl-CoA to support histone acetylation and chromatin accessibility. We demonstrate this nuclear metabolism is mediated by IDH2 and ACO2, which are present within the nucleus and bound to chromatin through their association with histone proteins. In the nucleus, IDH2 and ACO2 along with ACLY, form a complex on chromatin, which we term the nuclear metabolic complex (NMC) (Fig. 7j). Nuclear localization of ACO2 was found to be mediated by importin-α (KPNA2), which may bind to the bipartite NLS on the N-terminus of ACO2 to translocate the protein through the nuclear pore complex. Mechanisms regulating the nuclear entry of IDH2 yet to be characterized, although there are some putative NLS domains which could regulate its nuclear entry. The nuclear functions of ACO2 and IDH2 could be context-dependent, potentially reflecting cellular adaptations to specific metabolic and environmental stress conditions reflecting the cellular state. This could be induced by several upstream signals, including growth factors, hormones, nutrient signaling, hypoxia, and pH, which could potentially trigger nuclear localization of these enzymes. High-resolution confocal images revealed that nuclear CS, ACO2, and IDH2 appear as biomolecular condensates, although detailed studies are required to confirm the association of these enzymes with previously characterized nuclear bodies. Metabolic flux analysis in isolated nuclei confirmed IDH2 and ACO2 catalyze reductive carboxylation of α-KG to synthesize citrate, which serves as a carbon donor for histone acetylation. ACLY has been shown to generate nuclear acetyl-CoA from citrate^6^, and our findings have now identified α-KG as the carbon donor for citrate synthesis in the nucleus. Genetic depletion of these three metabolic enzymes significantly reduced several histone acetylation marks, indicating synthesis of acetyl-CoA within the nucleus is rate-limiting for epigenetic modifications. CS was also found to be present in the nucleus but was not part of the NMC, and its depletion increased several histone H3 acetylation marks. Metabolic homeostasis is often maintained by negative feedback, and it is possible CS may convert unused acetyl-CoA in the nucleus back to citrate, thereby protecting against uncontrolled histone acetylation by HATs. A recent report^25^ supports our observations indicating CS could influence histone acetylation levels, however specific mechanisms and metabolic reactions controlled by CS in the nucleus needs further investigation. Our biochemical screen also identified OGDH, an enzyme that generates succinyl-CoA from α-KG in the nucleus. A recent study has implicated OGDH controlling histone succinylation to promote tumor cell proliferation and development^26^. Collectively, our findings establish IDH2, ACO2, and ACLY form a functional nuclear metabolic pathway that regulates α-KG dependent histone acetylation.

Coordination of molecular events in the nucleus is critical for chromatin decondensation at transcriptionally active regions^27^. Our study highlighted the spatio-temporal association between nuclear metabolic enzymes (IDH2, ACO2 and ACLY) and KAT2A, promoting histone acetylation, that facilitated binding of pioneering factor FOXA1 (Fig. 7h). Pioneering factors are known to bind to genome prior to gene activation and facilitates assembly of transcription factors and coactivators^28^. Hence, we propose that nuclear metabolic pathway-dependent gene activation is likely induced by FOXA1, at least in part. Prominent histone acetylation marks actively regulated by the IDH2-ACO2 axis, such as H3K9ac and H3K14ac, are known to co-occur on many active promoters or enhancers, whereas H3K27ac is a well-recognized marker for active enhancers^29,30^.

We found proliferative gene MKI67 significantly induced by IDH2-ACO2 dependent recruitment of FOXA1. Nuclear-specific rescue of IDH2 and ACO2 were sufficient to induce tumor growth and proliferation independent of their mitochondrial activities, indicating the tumorigenic potential of the nuclear metabolic pathway. It is likely in absence of these enzymes in the mitochondria; cells reprogram their metabolic pathways to sustain bioenergetic needs. Our findings also align with a recent study demonstrating TCA cycle enzymes mediate global epigenetic changes during zygotic genome activation (ZGA)^9^. Our study underscores the importance of IDH2 and ACO2 along with ACLY forming an active metabolic complex on the chromatin, that utilizes α-KG to generate acetyl groups for KAT2A dependent epigenetic rewiring inducing proliferation and growth (Fig. 7h). Based on these observations, it is plausible that nuclear metabolic functions mediating epigenetic reprogramming may influence cellular decisions driving tumor growth, proliferation and differentiation, and cancer-specific vulnerabilities could be exploited for clinical benefits.

## Methods

### Cell culture

All research complies with the Institutional guidelines and ethical regulations (IBC: 160602). Glioblastoma cell lines GL261 and U87MG; breast cancer cell lines MDA-MB-231 and MDA-MB-468; human embryonic kidney cell line HEK293T; cervical cancer cell line HELA; and prostate cancer cell line VCaP were all cultured in DMEM (Gibco, Catalog# 11965092) supplemented with 10% fetal bovine serum (HI-FBS, Gibco, Catalog# 16140071), and 1% Antibiotic-Antimycotic (Gibco, Catalog# 15240062). Prostate cancer cell lines C4-2 and 22Rv1 were cultured in RPMI-1640 supplemented with 10% fetal bovine serum and 1% Antibiotic-Antimycotic. Normal prostate epithelial line RWPE-1 was cultured in Keratinocyte SFM supplement (Gibco, Catalog# 17005042) with Bovine Pituitary Extract (BPE), human recombinant epidermal growth factor (rEGF), and 1% Antibiotic-Antimycotic. Normal breast epithelial cell line MCF10A was cultured in DMEM/F12 (Invitrogen, Catalog# 11330-032) supplemented with 5% Horse Serum (Invitrogen, Catalog #16050-122), EGF (20 ng/mL), Hydrocortisone (0.5 mg/mL), Cholera Toxin (100 ng/mL), Insulin (10 µg/mL) and 1% Antibiotic-Antimycotic. All cell lines were obtained from ATCC except C4-2, which were a gift from Dr. Weigel, Baylor and GL261, which were a gift from Dr. Michael Ciesielski, Roswell Park. All cell lines were maintained at 37 °C with 5% CO2 and were periodically tested for mycoplasma using MycoAlert Mycoplasma Detection Kit (Lonza, Catalog# LT07–318).

### Sub-cellular fractionation

*Dignam* et al. protocol was used for cell fractionation with minor modifications to optimize nuclear and mitochondrial purity^31^. Cells were grown on a 15 cm^2^ plate until 80–90% confluency. Media was aspirated off the plates, followed by a cold PBS wash and 3 mL of hypotonic buffer (10 mM Tris-HCl, pH 8, 1-5 mM KCl, 1.5 mM MgCl_2_, 1 mM DTT, phosphatase inhibitor, and EDTA-free protease inhibitor) was added and incubated on ice for 15 minutes. Cells were scraped off using a cell lifter, which resulted in cell membrane rupture and nuclei release. To pellet the nuclei, the suspension was centrifuged at 700 x g for 5 minutes at 4 °C. The supernatant (cytosol, C) was centrifuged again at 11,000 x *g* for 15 min at 4 °C to isolate mitochondria (M). The nuclei pellet was washed once with 1 mL hypotonic buffer and was centrifuged at 700 x *g* for 5 min at 4 °C. To lyse the whole nucleus (N), isolated nuclei were resuspended in 500 µL of nuclear extraction buffer (20 mM HEPES, pH 7.9, 25% glycerol, 0.42 M NaCl, 0.2 mM EDTA, 1.5 mM MgCl_2_, 1 mM DTT, phosphatase inhibitor and protease inhibitor) and lysed on ice for 1 hr, with brief vortexing every 10 minutes. The mitochondrial pellet was lysed with 100 µL of RIPA buffer in the presence of phosphatase inhibitor and protease inhibitor on ice for 15 min.

Nuclear soluble (NS) and chromatin-bound (CB) proteins were separated from isolated nuclei based on the following protocol^32^. Nuclei were first resuspended in 500 µL of Buffer B (3 mM EDTA, 0.2 mM EGTA, 1 mM DTT, with protease and phosphatase inhibitors) and incubated on ice for 15 minutes, followed by centrifugation at 1700 x *g* for 5 min at 4 °C. The supernatant was saved as a nuclear soluble (NS) fraction. The pellet was washed once with Buffer B and centrifuged again at 1700 x *g* for 5 min at 4 °C. To isolate the chromatin-bound (CB) proteins, the pellet was resuspended in 500 µL of nuclear extraction buffer along with 200U of micrococcal nuclease (MNase) enzyme, 1 mM CaCl_2_ and phosphatase inhibitors and incubated on ice for 10 minutes, followed by incubation at room temperature for 5 min to digest the chromatin. Whole nucleus (N), chromatin-bound (CB), and mitochondrial (M) fractions were briefly sonicated on ice at 10% amplitude (1 second on, 0.1 second off) for a total of 6 seconds, followed by centrifugation at a 16,000 x *g* for 10 min at 4 °C. Supernatants were used as the final fraction.

### Antibodies

The primary antibodies used in this study, along with the source and catalog number, are listed in Supplementary Data 1.

### Immunofluorescence staining and confocal microscopy

Cells were seeded on a coverslip in a 24-well tissue culture plate for 48 hours. For mitochondrial staining, MitoTracker Deep Red (Thermo, Catalog# M22426) dissolved in DMSO was mixed in DMEM (no HI-FBS) and added to the cells for 10 minutes at 37 °C. The medium was aspirated off. Cells were fixed with 10% buffered formalin for 30 min at room temperature, followed by permeabilization with PBST (PBS with 0.25% Triton X-100) for 30 min at room temperature. Cells were blocked with PBSTA (PBS with 0.25% Triton X-100 and 3% BSA) for 1 h at room temperature. Cells were incubated with primary antibodies in PBSTA overnight at 4 °C. Cells were washed 3 times with PBS for 5 min each. Later, cells were incubated with fluorophore-conjugated secondary antibody in PBSTA for 1 h at room temperature in the dark. Stained cells were washed 3 times with PBS for 5 min each. Coverslips were later mounted on a slide with an Anti-fade Mounting Medium with DAPI (Vector Laboratories, Cat. No. H-1800). Coverslips were imaged using a Leica TCS SP8 confocal microscope under 100x oil immersion objective with white light (470nm-670nm) and 405 nm diode excitation lasers and detected using PMT and hybrid detectors (high-sensitivity photocathode and APD). For z-stack scanning, images were taken at 0.2 µm increments from the top to the bottom of a cell. Images were analyzed and deconvoluted using integrated Leica LAS X and Huygens deconvolution software system. Fluorescence intensities of a Z-stack image cutting through the middle of a nucleus at a selected X-Y plane were measured using ImageJ software and plotted using GraphPad Prism 9 software.

### Imaging flow cytometry

After cell culture, 1-2 × 10^6^ cells were collected and first stained with MitoTracker Deep Red (Thermo, Catalog# M22426) in DMEM or RPMI medium for 10 min at 37 °C. Cells were spun down at 500 x *g* for 5 min at 4 °C and washed with PBS. Next, cells were fixed and permeabilized using Foxp3 Fixation/Permeabilization working solution (eBiosciences, 00-5523-00) at room temperature for 45 minutes. Cells were then spun down at 500 x *g* for 5 min at 4 °C and washed with permeabilization buffer. Afterwards, cells were incubated with primary antibodies (ACO2, IDH2, Tom20, and Histone H3) in a permeabilization buffer at room temperature for 45 minutes. Cells were spun down at 500 x *g* for 5 min at 4 °C and washed with permeabilization buffer. Cells were then incubated with secondary antibodies conjugated to a fluorophore in a permeabilization buffer for 30 minutes at room temperature (protected from light). Cells were spun down at 500 x *g* for 5 minutes at 4 °C and washed with permeabilization buffer. Cells were spun down and resuspended in a 50 µl flow buffer with DAPI. Images were acquired using the INSPIRE™ software on the ImagestreamX Mark II imaging flow cytometer at 40 × magnification, with lasers 405 nm, 488 nm, 640 nm, and side scatter (785 nm). Single color controls for each of the specific markers were acquired for compensation. Nuclear localization of metabolic enzymes was analyzed using IDEAS™ (v6.2) analysis software using the nuclear localization wizard, which calculates the mean similarity of a DAPI and protein of interest (ACO2, IDH2, Tom20, and Histone H3) using Pearson’s Correlation Coefficient. A mean similarity greater than or equal to 1 (R1 ≥ 1) was considered positive for nuclear localization^14^.

### Isolation of intact nuclei and immunofluorescence staining

The protocol for the isolation of intact nuclei for confocal microscopy was adapted from the Sardo et al. group^33,34^. Briefly, cells were trypsinized, washed with ice-cold PBS, and a pellet of 10 million cells was spun down, snap-frozen with liquid N_2_, and stored at − 80 °C until the nuclei isolation. Hypotonic buffer (10 mM Tris-HCl, pH 8, 1-5 mM KCl, 1.5 mM MgCl_2_, 1 mM DTT, phosphatase inhibitor, and EDTA-free protease inhibitor) was added to the cell pellet and incubated on ice for 5 min. The nuclei pellet spun down at 700 x *g* for 5 min at 4 °C. The nuclei pellet was incubated again in the hypotonic buffer for 5 min on ice and spun down at 700 x *g* for 5 min at 4 °C. The purity of the isolated nuclei was confirmed under microscopy using a Trypan Blue solution (Thermo, Catalog# 15250061). The nuclei pellet was later washed with 1 mL of nuclei PURE storage buffer (Sigma, Catalog # S9183) in the presence of phosphatase inhibitor and protease inhibitor. The nuclei pellet was resuspended in 100 μL nuclei PURE storage buffer. For blocking, 1 mL of 5% HI-FBS in PBS was added to the sample and incubated on ice for 1 hour. The nuclei pellet was spun down at 700 x *g* for 5 min at 4 °C. Primary antibodies (ACO2, IDH2, CS, Tom20, Lamin A/C) in 5% HI-FBS in PBS were added to the nuclei pellet and stained at 4 °C overnight. The nuclei pellet was washed 3 times using 500 μL of 5% HI-FBS in PBS. Secondary antibodies conjugated to a fluorophore in 5% HI-FBS in PBS were added to the samples. The samples were incubated at 4 °C for 1 h and at room temperature for 10 min in the dark. The nuclei pellet was washed 3 times using 500 μL of 5% FBS in PBS. After the last wash, the nuclei were resuspended in 10 μl mounting media with DAPI (Vector Lab, Catalog# H-1800-10) and mounted on a slide. Stained nuclei were imaged using a Leica TCS SP8 Confocal Microscope under a 63X oil immersion objective, illuminated with 405, 488, and 638 nm diode laser lines, and detected using a hybrid spectral detector. Images were analyzed and deconvoluted using Leica LAS X software.

### Generation of nuclear specific ACO2 and IDH2 constructs

ACO2 gene (NM_001098) and IDH2 gene (NM_002168.4) under doxycycline-inducible promoter with blasticidin antibiotic selection marker (Catalog # TLO2034_Inducible_Lenti-TRE3G-ACO2-P2A-tRFP-PGK-Tet3G-bsd, Catalog # TLO2051_Inducible Lenti-TRE3G-IDH2-op-P2A-mCherry-PGK-Tet3G-Blasticidin) were purchased from Transomic technologies. Using the Q5 Site-directed mutagenesis kit (NEB, Catalog # E0554S), ACO2 and IDH2 mitochondrial transition sequence (MTS) were deleted, and simultaneously the nuclear localization sequence (NLS) of SV40 (PKKKRKV) was inserted into ACO2 (ACO2-∆MTS-NLS) and IDH2 (IDH2-∆MTS-NLS). The mutated NLS ACO2 (ACO2-mNLS) was generated by cloning the mutated fragment ACO2_mutated-NLS_fragment-CR456365.1: GGGTGTGCGGCAGTACCATGTGGCCTCAGTCCTGTGCCAAGCGGCCGCGGTGGCGATGAGCCACTTTGAGCCCAACGAGTACATCCATTATGACCTGCTAGAGGCGAACATTAACATTGTTGCGAAACGACTGAACCGGCCGCTGACACTCTCGGAGAAGATTG into the ACO2 construct using HIFI DNA assembly kit (NEB, Catalog # E5520S). Modified ACO2 and IDH2 plasmid were transformed into NEB 5-alpha Competent E. coli and individual clones were selected for plasmid amplification and isolation. Plasmid sequences were confirmed using Sanger sequencing. ACO2-∆MTS-NLS and ACO2-mNLS lentivirus were used to transduce MDA-MB-468_shACO2 or C4-2_shACO2 cells (ACO2 3’UTR targeting shRNA clone # TRCN0000114531), whereas IDH2-∆MTS-NLS lentivirus was used to transduce MDA-MB-468_shIDH2 (IDH2 3’UTR targeting shRNA clone # TRCN0000428288). Stably transduced cells were selected under 5 µg/mL blasticidin for 3 weeks. As a vector control, an empty vector purchased from Transomic technologies under a doxycycline inducible promoter was transduced in shNT, shACO2, and shIDH2 cell lines, which were stably selected using 400 µg/mL Neomycin for 3 weeks. Cells were kept under 1 µg/mL doxycycline for 2 passages to induce the expression of the construct.

ACO2_MTS: PYSLLVTRLQKALGVRQYHVASVL (1-28aa) was deleted.

IDH2_MTS: MAGYLRVVRSLCRASGSRPAWAPAALTAPTSQEQPRRHY (1-39aa) was deleted.

2X-SV40 NLS: PKKKRKVPKKKRKV was inserted for both plasmids.

Site-directed mutagenesis primers for -∆MTS-NLS.

ACO2 primers:

Forward: CCGAAAAAAAAACGCAAAGTGTGCCAACGGGCCAAGGTG

Reverse: CACTTTGCGTTTTTTTTTCGGCGGCGCCATGGTGGCGGCCTC

IDH2 primers:

Forward: CCGAAAAAAAAACGCAAAGTGGCAGATAAGAGAATAAAAGTAGCCAAACCAG

Reverse: CACTTTGCGTTTTTTTTTCGGCATGGTGGCGGCCTCGAG

### Lentivirus production

Hek293T cells were seeded in a 10 cm2 tissue culture plate. At 75% confluency, cells were transfected with pMD2.G, 20 μg, (Addgene, Cat. No. 12259) and psPAX2, 10 μg, (Addgene, Cat. No. 12260) along with the plasmid of interest, 25 μg, using lipofectamin 2000 (Invitrogen, Cat # 11668500). The media containing lentivirus particles were harvested after 48–72 h followed by incubation with PEG-it Virus Precipitation Solution (System Biosciences) overnight at 4 °C. Lentivirus particles were precipitated at 1500 x *g* at 4 °C for 30 min, which were later resuspended in cold PBS and store at − 80 °C.

### shRNA or sgRNA mediated knockdown

Cells were seeded in a desired tissue culture. The next day, a lentivirus carrying a specific shRNA construct was added to cells in the presence of polybrene (10 µg/mL). After 4 days, cells were either directly used for certain experiments or were grown in the presence of specific antibiotics to generate stable cell lines. Puromycin 1 µg/mL, Blasticidine 0.5 µg/mL for MDA-MB-468 and 1 µg/mL for GL261, Neomycin 400 µg/mL for MDA-MB-468, 800 µg/mL for GL261. The details of each shRNA are provided in Supplementary Table 1. Three different clones of all-in-one lenti-U6-ACO2 gRNA-Cas9-puro vectors were obtained from Baylor CBASS core, and C4–2 cells were transduced with the packaged lentiviruses and selected under 1 μg/mL puromycin, as reported^20^.

### Whole-cell protein lysis

Whole cells were lysed using NP-40 lysis buffer (Thermo, Catalog # FNN021) with protease and phosphatase inhibitors at 4 °C for 30 min, with 15 s vortexing every 5 minutes. Samples were sonicated at 10% amplitude (1 s on, 0.1 s off) for a total of 6 seconds. To remove any debris, the samples were centrifuged for 16000 x *g* for 15 min at 4 °C. The supernatant was transferred into a new tube. Protein concentrations were estimated using a Micro BCA protein assay kit (Thermo, Catalog # 23235).

### Immunoblotting

Immunoblotting was performed as described previously^35,36^. Briefly, protein lysates were denatured using LDS sample buffer (Thermo, Catalog# 1882363) and reducing agent (Thermo, Catalog# NP0009). The samples were heated at 95 °C for 5 min. The protein samples were separated on gradient NuPAGE 4-12% Bis-Tris gels (Thermo, Catalog# NP0323), followed by a semi-dry transfer of proteins onto nitrocellulose membrane (Thermo, Catalog# IB23001) using iBlot 2 Dry Blotting System (Invitrogen). The membranes were blocked with 5% milk in 1X TBST for 1 h on a shaker at room temperature. The membranes were incubated with primary antibodies in 5% Bovine Serum Albumin (BSA) or 5% milk in 1X TBST overnight on the shaker at 4 °C. The next day, the membranes were incubated at room temperature for 30 minutes on a shaker. Primary antibodies were removed, and the membranes were washed 3 times with 1X TBST for 5 min. The membranes were later incubated with HRP-conjugated rabbit or mouse secondary antibodies, diluted in 5% milk in 1X TBST (1:2500), for 2 h at room temperature. Membranes were washed 3 times with 1X TBST for 10 min at room temperature. The membranes were developed using ECL substrate (Thermo, Catalog# 32132) and acquired using the ChemiDoc Imaging system (Bio-Rad). Densitometry analysis was performed using either Image Lab (Bio-Rad) or ImageJ software. Band intensities were normalized to the membrane background. Fold changes were calculated by normalizing the band intensities to the loading control proteins, either beta-actin or Histone H3. Immunoblotting of sub-cellular fractions were derived from the same experiment but were run on different gels for identical molecular weight protein and were processed in parallel. Histone extracts were also derived from same experiments but run on different gels for H3Ac, H4Ac, and H3Me marks due to similar molecular weights and were processed in parallel. For immunoprecipitation followed by immunoblotting, samples were derived from the same experiment but were run on different gels for identical molecular weight proteins and were processed in parallel. Detailed information for each figure included in the reporting summary.

### Coomassie Blue staining of protein gels

Lysed proteins were first separated on NuPAGE 4-12% Bis-Tris gels (Thermo, Catalog# NP0323). Gels were stained with Coomassie Brilliant blue solution (Sigma, Catalog # B6529) for 10 min at room temperature on the shaker. Gels were washed with 10% acetic acid at room temperature on the shaker until clear bands were visible. Gels were imaged using the ChemiDoc Imaging system (Bio-Rad).

### Immunoprecipitation (IP)

Nuclei were isolated from the cells as previously described^37^. Isolated nuclei were lysed with nuclear extraction buffer (20 mM HEPES, pH 7.9, 25% glycerol, 0.42 M NaCl, 0.2 mM EDTA, 1.5 mM MgCl_2_, 1 mM DTT, phosphatase inhibitor and protease inhibitor) in the presence of 200U MNase enzyme, 1 mM CaCl_2_ and protease and phosphate inhibitors, for 1 hour on ice, with vortexing every 10 min. Samples were sonicated at 10% amplitude (1 s on, 0.1 s off) for a total of 6 seconds, followed by centrifugation at 16,000 x *g* for 10 min at 4 °C. The supernatant was collected, and the salt concentration in the nuclear lysates was reduced to 0.21 M NaCl by adding an equal volume of nuclear extraction buffer without any NaCl. This supernatant was used as a final nuclear fraction.

For IP-proteomics, 3 mg of nuclear lysates were pre-cleared with protein G beads (ThermoFisher, Catalog# 88848) on a rotator for 1 h at 4 °C. Pre-cleared beads were washed 2 times with NP40 lysis buffer and 2 times with PBS. Pre-cleared supernatant was incubated with ACO2 (Cell Signaling, Catalog # 6571S, 6 µL) or IDH2 (Thermo, Catalog# MAS-17271, 6 µL) antibodies and incubated on a rotator at room temperature for 1 hour. Protein G beads were added to each sample and incubated for another 30 min at room temperature. ACO2 and IDH2 bound beads were separated and washed 2 times with NP40 lysis buffer and 2 times with PBS. Precleared and proteins bound to beads were subjected to LC-mass spectrometry-based proteomics analysis. Protein samples were digested on beads using Trypsin/Lys-C enzymes. The resulting digested peptides were then enriched and desalted using an in-house STAGE tip column^38^ packed with 2 mg of C18 beads (3 µm, Dr. Maisch GmbH, Germany) and subsequently vacuum dried. The resuspended peptides were subjected to nanoLC-MS/MS using a Thermo Scientific nanoLC-1000 system coupled to an Orbitrap Fusion mass spectrometer with an ESI source. Peptides were initially loaded onto an in-house Reprosil-Pur Basic C18 trap column (1.9 µm, Dr. Maisch GmbH, Germany) (2 cm length, 100 µm i.d.) before separation on a 5 cm analytical column (150 µm i.d.). Separation was achieved using a 75 min gradient of 0–35% acetonitrile/0.1% formic acid at a flow rate of 800 nl/minutes. Data acquisition was performed in data-dependent analysis mode (DDA) to enable unbiased peptide detection. Spectra obtained were searched against the Human RefSeq database (release 2020), including target-decoy searches, using Proteome Discoverer 2.1 interface (PD 2.1, Thermo Fisher) with the Mascot algorithm (Mascot 2.4, Matrix Science). The peptides identified from the Mascot result file were validated with 5% false discovery rate (FDR). For relative quantification, the data was then grouped into gene products and assigned homology and identification quality groups using an in-house developed algorithm. Each gene product amount was estimated using a label-free intensity-based absolute quantification (iBAQ) approach.

For IP-immunoblotting, 1 mg of nuclear lysates were precleared with protein G beads for 1 hour at 4 °C. Pre-cleared supernatant was later transferred into a new tube and incubated with ACO2, IDH2, KAT2A, rabbit IgG, or mouse IgG antibodies along with protein G beads on a rotator overnight at 4 °C. Protein-bound beads were separated and washed twice with nuclear extraction buffer, twice with PBS, and once with H2O. 90 µL of 2X LDS sample buffer (Thermo, Catalog# 1882363) and 10 µL reducing agent (Thermo, Catalog# NP0009) was added to beads and heated at 95 °C for 5 min to elute and denatures the proteins. The samples were later used for immunoblotting analysis.

### Histone extraction

For histone acetylation comparison between shRNA and sgRNA knockdown cell lines, cells were grown until 80-90% confluency. Fresh medium was added for 1 h before cell pellet collection. For glutamine studies, cells were grown until 80% confluency, followed by overnight glutamine starvation using DMEM without glutamine (10% dialyzed FBS, 1% antibiotic). For glutamine rescue, the medium was later changed with DMEM with glutamine (4 mM) (10% dialyzed FBS, 1% antibiotic) for 6 hours.

Histones were extracted using the following protocol^39^. Cell pellets (5 × 10^6^ cells) were resuspended in 1 mL of hypotonic buffer (10 mM Tris, pH 8.0, 1 mM KCl, 1.5 mM MgCl_2_, 1 mM DTT, 10 mM sodium butyrate with protease and phosphatase inhibitors) and incubated at 4 °C on a rotator for 30 minutes. The nuclei pellet was isolated by centrifuging at 10,000 x *g* for 10 minutes at 4 °C. The nuclei pellet was resuspended in 400 μL of 0.4 N H2SO4 and incubated overnight on a rotator at 4 °C. The supernatant was collected by centrifuging for 16,000 x *g* for 10 minutes at 4 °C. 132 μl of 100% TCA was added dropwise to the supernatant and incubated on ice for 2 h. Histones were pelleted down by centrifuging the samples at 16,000 x *g* for 15 min at 4 °C. The pellet was washed with 1 mL of ice-cold acetone. The samples were allowed to dry at room temperature, which was later resuspended in 100 μL of water.

### In-vitro HAT assay using intact nuclei

Cells were grown on 10 cm^2^ plates until 80% confluency, followed by an overnight (16 h) glutamine starvation (DMEM without glutamine with dialyzed FBS and antibiotics). To isolate nuclei, the media was first aspirated off, followed by a cold PBS wash. 2 mL of hypotonic buffer (10 mM Tris-HCl, pH 8, 5 mM KCl, 1.5 mM MgCl_2_, 1 mM DTT, phosphatase inhibitor, and EDTA-free-protease inhibitor) was added onto the plate and incubated on ice for 15 minutes. Using a cell lifter, cells were scraped off to rupture the cell membrane and release nuclei. The suspension was centrifuged at 700 x *g* for 5 min at 4 °C to pellet the nuclei. Nuclei were washed once with the hypotonic buffer.

For HAT assay with intact nuclei, isolated nuclei were counted and resuspended at 2 × 10^6^ nuclei /100uL in HAT assay buffer (50 mM Tris, pH 8.0, 50 mM NaCl, 0.1 mM EDTA, 50 µg/mL BSA, 0.01% triton X-100, 1 mM DTT, 10 mM Sodium butyrate, 0.075 mM spermine, 0.25 mM spermidine, protease, and phosphatase inhibitor)^40^. HAT activity relied on endogenous HAT enzymes present in the isolated nuclei.

For HAT assay with nuclear lysates, isolated nuclei were first lysed with nuclear extraction buffer (20 mM HEPES, pH 7.9, 25% glycerol, 0.42 M NaCl, 0.2 mM EDTA, 1.5 mM MgCl_2_, 1 mM DTT, phosphatase inhibitor and protease inhibitor) for 30 minutes on ice with brief vortexing every 5 min, followed by sonication (2 times, total 6 seconds, on 1 s, off 0.1 s, 10% amplitude). Lysates were spun down at max speed for 10 minutes at 4 °C. NaCl salt concentration in the supernatant was lowered down to 0.21 M using 0 M NaCl nuclear extraction buffer along with the addition of 50 µg/mL BSA, 0.01% triton X-100, 1 mM DTT, 10 mM sodium butyrate, protease, and phosphatase inhibitor. These nuclear lysates were later used for the HAT assay. HAT activity relied on endogenous HAT enzymes that were present in the nuclear lysates.

Isolated nuclei suspended in HAT assay buffer or nuclear lysates mentioned above were incubated at 30 °C for 10 min to pre-warm the sample. Later, 500 µM ATP (required for ACLY enzymatic activity) along with respective metabolites (Vehicle (H_2_O), 10 µM Acetyl-CoA, 2 mM citrate, 2 mM α-KG and 2 mM glutamate) were added to the nuclei and incubated at 30 °C for 2 h, unless specified, for HAT assay. After the assay, samples were used for immunoblotting analysis, MNase digestion assay, Nascent-RNA assay, metabolomics, or proteomics. For immunoblotting analysis, after the HAT assay, samples were sonicated at 10% amplitude (1 s on, 0.1 s off) for a total of 6 seconds. Loading dye and reducing agent were added to each sample and were heated for 5 min at 95 °C. The effect of the added metabolites on histone acetylation marks was analyzed through immunoblotting.

### MNase digestion assay

Nuclei were isolated from untreated, or glutamine-starved cells as mentioned before. Untreated nuclei were used directly for the MNase digestion assay. However, to determine the impact of metabolites on chromatin, an in-vitro HAT assay was performed as mentioned above before proceeding with the MNase digestion assay. After the assay, nuclei were spun down at 1000 x *g* for 5 min at 4 °C. Nuclei were resuspended at 2 × 10^6^ nuclei /100uL in MNase buffer (15 mM Tris HCl, pH 8, 15 mM NaCl, 60 mM KCl, 0.34 M Sucrose, 1% Beta-mercaptoethanol, 1 mM CaCl_2_, 0.15 mM spermine, 0.5 mM spermidine and phosphatase inhibitor) in the presence of MNase enzyme (Final conc: 0-0.5U/µL). For chromatin digestion, samples were incubated at 37 °C for 15 min. 5 µL of 0.5 M EDTA was added to each sample to stop the enzymatic digestion. Samples were deproteinized by adding 36 µL of H_2_O, 24 µL of 10% SDS, and 48 µL of 5 M NaCl. To extract the digested DNA, 200 µL of phenol/chloroform/isoamyl alcohol mix was added and briefly vortexed, followed by centrifugation at max speed for 10 minutes. The top layer was transferred into a new tube, and 10% (v/v) 3 M sodium acetate was added to it. 2.5 times (v/v) of 100% ethanol was added to each sample and was kept overnight at − 20 °C to precipitate the digested DNA. DNA was pelleted by centrifugation at max speed for 15 min. The pellet was washed with 500 μL of 75% ethanol. The DNA pellet was later air-dried and resuspended in H_2_O. DNA concentrations were measured using a nanodrop, and around 500 ng of DNA was separated on 1% agarose gel and analyzed.

### Nascent-RNA assay

Nascent RNA synthesis in isolated nuclei was analyzed using Click-iT™ RNA Alexa Fluor™ 488 Imaging Kit (ThermoFisher Scientific, Catalog# C10329) with some modifications. Nuclei were isolated from glutamine-starved cells as previously described. Isolated nuclei were resuspended in HAT assay buffer (2 × 10^6^ nuclei /100 µL) along with 500 µM of NTP mix (ThermoFisher Scientific, Catalog# 18109017), 200 U/mL SUPERase•In™ RNase Inhibitor (ThermoFisher Scientific, Catalog# AM2694). The samples were pre-warmed at 30 °C for 10 minutes, followed by the addition of 500 µM ATP, vehicle (H2O), or 2 mM α-KG and 3 mM of 5-ethynyl uridine (EU) to label nascent RNA. The assay was performed at 30 ^°^C for 2 h. Treated nuclei were spun down at 700 x *g* for 10 min at 4 °C. The nuclei were fixed using 200 µL of 10% formalin in PBS for 10 minutes at room temperature. Nuclei were spun down at 700 x *g* for 5 min at 4 °C, followed by PBS wash. To detect the EU-bound RNA, 200 µL of Click-iT reaction cocktail that includes Alexa Fluor-488-azide was added to the nuclei and incubated at room temperature for 30 min in the dark. Later, the samples were spun down at 700 x *g* for 5 min at 4 °C, followed by a wash with 300uL of Click‑iT reaction rinse buffer. Finally, nuclei were resuspended in the flow buffer along with DAPI. Samples were analyzed with a Flow cytometer, and the data was analyzed using FSC Express 7 software. Single nuclei were gated, and the intensity of 5’-EU labeled nascent RNA bound to Azide-488 was measured on the FITC channel.

### Isotope tracing experiments in isolated nuclei followed by metabolomics

In-vitro HAT assay using intact nuclei was performed as mentioned previously in the presence of vehicle (H_2_O) or 2 mM U-13C5 α-KG (Cambridge isotope, Catalog# CLM-2411-0.1) for 1 hour at 30 °C. After the assay, samples were snap-frozen and stored in liquid nitrogen until targeted metabolomics. ^13^C labeling of citrate (m + 5) was analyzed through targeted mass spectrometry, using ultra–high performance LC (UPLC) coupled to MS/MS (Agilent 1290 infinity series UPLC system and Agilent 6495 mass spectrometer; Agilent Technologies), as described previously^37^. Briefly, an X-bridge amide column (3.5 μm, 4.6 × 100 mm; Waters) was applied in electrospray ionization (ESI) positive mode as described earlier. Mobile phase A and B were 0.1% FA in water and ACN, respectively. Gradient flow was as follows: 0–3 min 85% B; 3–12 min 85%–30 % B, 12–15 min 30%–2 % B, and 16 min 95% B, followed by re-equilibration until the end of the gradient at 23 min to the initial starting condition of 85% B. Flow rate of the solvents used for the analysis was 0.3 mL/min. The injection volume was 5 μL^41^.

### Isotope tracing experiments followed by proteomics

MDA-MB-468 expressing shNT, shACO2, and shACO2 with inducible expression of ACO2-∆MTS-NLS were cultured in the presence of U-13C Glutamine (Cambridge isotope) for 2 h at 37 °C. Cells were washed and snap frozen. Histones were extracted from the nuclei of frozen cell pellets by acid extraction, as mentioned before^42,43^. Histone peptides were analyzed by EasyLC 1000 or Dionex UltiMate 3000 LC systems as previously reported, with minor modifications. In brief, the affinity-purified protein was digested on beads using Trypsin/Lys-C enzymes. The resulting digested peptides were then enriched and desalted using an in-house STAGE tip column^38^ packed with 2 mg of C18 beads (3 µm, Dr. Maisch GmbH, Germany) and subsequently vacuum dried. The resuspended peptides were subjected to nanoLC-MS/MS using a Thermo Scientific nanoLC-1000 system coupled to an Orbitrap Fusion mass spectrometer with an ESI source. Peptides were initially loaded onto an in-house Reprosil-Pur Basic C18 trap column (1.9 µm, Dr. Maisch GmbH, Germany) (2 cm length, 100 µm i.d.) before separation on a 5 cm analytical column (150 µm i.d.). Separation was achieved using a 75 min gradient of 0–35% acetonitrile/0.1% formic acid at a flow rate of 800 nl/minutes. Data acquisition was performed in data-dependent analysis mode (DDA) to enable unbiased peptide detection. Spectra obtained were searched against the Human RefSeq database (release 2020), including target-decoy searches, using Proteome Discoverer 2.1 interface (PD 2.1, Thermo Fisher) with the Mascot algorithm (Mascot 2.4, Matrix Science). Dynamic modifications such as acetylation on lysine, acetyl-^13^C2 (m + 2) on lysine, and oxidation of methionine were allowed. Precursor mass tolerance was set at 20 ppm, with a fragment mass tolerance of 0.5 Da, and a maximum of two missed cleavages were permitted. Assigned peptides were filtered with a 1% false discovery rate (FDR) using percolator validation based on q-value. Post-translational modification (PTM) sites were manually validated.

### Acetyl-CoA measurement

Acetyl-CoA synthesis in isolated nuclei was measured using an acetyl-CoA assay kit (Millipore Sigma, Catalog# MAK039-1KT) with some modifications. In-vitro HAT assay was performed as previously described in the presence of vehicle (H_2_O) or 2 mM α-KG at 30 °C for 0, 30, 60 and 120 min. Samples were sonicated at 10% amplitude (1 s on, 0.1 s off) for a total of 6 s. 10 µL of each sample was used for total nuclear protein estimation, and the rest was used for Acetyl-CoA measurement. 20 µL of 4 M ice-cold perchloric acid was added to each tube and kept on ice for 5 minutes, followed by centrifugation at 16000 x *g* for 10 min. The supernatant was transferred into a new tube, and 10 μL of 5 M KOH was added and kept on ice for 5 minutes to neutralize the sample. Samples were centrifuged at 16000 x *g* for 10 min, and the supernatant was used for acetyl-CoA measurement using the assay kit protocol.

### ATAC-seq

ATAC-seq samples and libraries were prepared following the Buenrostro et al. protocol with the following modifications^44^. Nuclei were isolated from 50,000 cells. The samples were tagmented by resuspending nuclei in 2.5 μL TDE1 transposase (Illumina; FC-121-1030), 25 μL TD 2x buffer (Illumina; FC-121-1030), 22.5 μL nuclease-free water, followed by incubation at 37 °C for 30 min. The tagmented DNA fragments were purified by using a Qiagen MinElute PCR Purification kit. The purified DNA fragments were amplified by PCR (10 cycles) using KAPA Hi Fi 2X ready-mix (Roche). The amplified products were purified with AmpureXP beads (Beckman Colter), followed by verification of the final library profiles by HSD1000 screentape on a Tapestation 4200 (Agilent). The libraries were quantitated using the KAPA Biosystems qPCR kit (Roche) and were pooled together in an equimolar fashion, following experimental design criteria. The resulting pool was then loaded into the appropriate NovaSeq Reagent cartridge and sequenced on a NovaSeq6000 following the manufacturer’s recommended protocol (Illumina Inc.).

### ATAC-seq analysis

ATAC-seq reads were aligned to the human genome (version GRCh38) with bowtie2. Picard was used to mark and remove the duplicated reads. Accessible peak regions for each replicate were called using MACS2, with a q-value cutoff of 0.05 in paired-end mode^45^. All the peaks in replicates were merged to identify consensus genomic region peaks. UCSC toolkit bigWigAverageOverBed was used to extract the counts of fragments for each replicate and to make a count table for the differential analysis. Integrative Genomics Viewer was used to explore chromatin accessibility. To identify differentially accessible regions (DARs), the count table containing fragment counts was loaded into DESeq2. The Wald test for the negative binominal general linear model coefficients was used to call the DARs, with FDR cutoff ≤ 0.05. Using the ChIPseeker package and the “annotatePeak” function, the genomic distribution of opening and closing DARs were determined. Motif enrichment analysis was performed using AME (analysis of motif enrichment) that utilizes the MEME suite. Gene set enrichment analysis (GSEA) was performed using the molecular signatures database (MSigDB) using *clusterProfiler* and *fgsea* packages in R. To find potential transcription factors associated with ACO2 cistromes, we utilized GIGGLE to query the CistromeDB database, which includes experimentally collected ChIP-seq data for 1111 transcription factors and 75 histone marks^46^.

### RNA extraction

Approximately, 1 × 10^6^ cells were lysed with QIAzol lysis reagent, followed by the addition of chloroform. Aqueous and organic phases were later separated by centrifugation. Aqueous phases were transferred to a new tube, followed by the addition of 100% ethanol to it, and mixed by pipetting. The sample was later transferred into an RNeasy Mini spin column for collecting total RNA. On-column DNAse digestion was performed to remove residual genomic DNA. High-quality RNA was later eluted in 60 µL of RNase-free water. Qubit Broad Range RNA kit (Thermofisher) was used for quantitative assessment of the purified total RNA. The concentration of the RNA was determined by Ribogreen fluorescent binding to the isolated RNA. Qualitative analysis of the RNA was performed using RNA Nanotape on the 4200 Tapestation (Agilent Technologies), where sizing of the RNA was determined, and a qualitative numerical score^47^ was assigned.

### RNA-seq

About 500 ng of total RNA was used to make sequencing libraries with the mRNA HyperPrep kit (Roche) as per the manufacturer’s instructions. First, Poly(A) RNA was captured using mRNA Capture Beads (KAPA BIOSYSTEMS). RNA was eluted, fragmented, and primed for cDNA synthesis. 1st strand of cDNA is later synthesized with random primers. Later, the RNA template gets removed, and a replacement strand is synthesized by incorporating dUTP in place of dTTP to generate ds cDNA. Pure Beads (Roche) were used to separate the ds cDNA from the second-strand reaction mix, resulting in blunt-ended cDNA. A single ‘A’ nucleotide was then added to the 3’ ends of the blunt fragments. Multiple indexing adapters, containing a single ‘T’ nucleotide on the 3’ end of the adapter, were ligated to the ends of the ds cDNA, preparing them for hybridization onto a flow cell. Adapter ligated libraries were amplified by PCR, purified using Pure Beads, and validated for appropriate size on a 4200 TapeStation D1000 Screentape (Agilent Technologies, Inc.). The DNA libraries were quantitated using KAPA Biosystems qPCR kit and were pooled together in an equimolar fashion, following experimental design criteria. Each pool was denatured and diluted to 350pM with 1% PhiX control library added. The resulting pool was then loaded into the appropriate NovaSeq Reagent cartridge, as determined by the number of sequencing cycles desired, and sequenced on a NovaSeq6000 following the manufacturer’s recommended protocol (Illumina Inc.).

### RNA-seq analysis

Using Salmon, quasi-alignment of raw reads to the human transcriptome (hg19) and transcript abundance estimation was performed. Using DESeq2, estimated transcript abundance was normalized, and differentially expressed genes (DEGs) were identified. Gene set enrichment analysis (GSEA) was performed using gene sets from the molecular signatures database (MSigDB) using *clusterProfiler* and *fgsea* packages in R.

### Chromatin immunoprecipitation

ChIP assay was performed as described in the ChIP-IT Express kit (Active Motif # 53008) protocol with some modifications. MDA-MB-468 cells were grown on a 15 cm^2^ plate until 80% confluency. Cells were given fresh medium 4 h prior to fixation using formaldehyde. After cross-linking, samples were sonicated using a water bath sonicator (Diagenode Bioruptor UCD-200) for a total 30 min (5 min cycle, 30 son 30 s off). ChIP was performed using 6 µL FOXA1 (Cell signaling # 53528S) and rabbit IgG as a control. qPCR was performed with eluted ChIP DNA using specific MKI67 and IL1RN-1 promoter primers.

MKI67 Forward: TTCAGAAGGTTCCTCCAAAGG

MKI67 Reverse: GGTAAGTGTGAGATGGGAGATG

IL1RN-1 Forward: CCTTGGTCACACTATCCACATT

IL1RN-1 Reverse: CTGAGGCTGACAATGGTTAAGA

### Cell proliferation assay

Cells were grown in the presence of 1 µg/mL doxycycline for 4 days. Cells were counted, and 1000 cells were seeded in a 96 well (*n* = 6) with 1 µg/mL doxycycline for the cell proliferation assay. Cell proliferation rate was measured every 2 days using an MTS-based cell proliferation kit (Promega, Catalog# G3581). Briefly, 100 µL new medium was added to each well along with 20 µL of CellTiter 96® Aqueous One Solution Reagent. Plates were incubated at 37 °C for 1 hr. Absorbance was measured at 490 nm using 96 well plate reader.

### Colony formation assay

For the ACO2 and IDH2 rescue experiment, cells were first cultured in the presence of 1 µg/mL doxycycline for 4 days. Cells were trypsinized and counted. Around 3000 cells were seeded in a 6 well (*n* = 3) for colony formation assay. Cells were allowed to proliferate for 14 days in the presence of 1 µg/mL doxycycline. The medium was aspirated off, and the colonies were fixed with 100% ice-cold methanol at room temperature for 30 minutes. The colonies were stained with 0.25% crystal violet in methanol at room temperature for 1 h. Wells were rinsed with water to get rid of the extra dye. The plates were air-dried at room temperature and imaged using the ChemiDoc Imaging system (Bio-Rad). Colonies were counted using ImageJ software.

### Animal studies

Animal experiments were carried out in accordance with protocol 1391 M approved by the Roswell Park Institutional Animal Care and Use Committee following NIG guidelines. The maximal tumor size and burden set by the ethics committee was a tumor diameter of 2 cm for subcutaneous xenografts, and for orthotopic xenografts, humane endpoint criteria were used, including total body weight loss of greater than 15–20%, impaired mobility, abdominal distention, and other indications of compromised animal welfare; no animals exceeded these limits. MDA-MB-468 cells stably expressing shNT + vector-control, shIDH2 + vector-control, or shIDH2 + IDH2-∆MTS-NLS were cultured in the presence of doxycycline (1 µg/mL) for one week prior to implantation in the mice. Similarly, C4-2 cells stably expressing shNT + vector-control, shACO2 + vector-control, or shACO2 + ACO2-∆MTS-NLS were cultured in the presence of doxycycline (1 µg/mL) for one week prior to implantation. NOD.Cg-Prkdc^scid^ Il2rg^tm1Wjl^/SzJ (NSG) (Roswell Park in-house) mice were put on doxycycline chow (2000mg/kg) (TD.09633 Rodent Diet) for 1 week prior to injection and kept on the doxycycline chow (2000mg/kg) until the end of the study. Two million cells mixed in 1:1 PBS: Matrigel were injected orthotopically into the number four mammary fat pad of 7-9 weeks old female NOD.Cg-Prkdc^scid^ Il2rg^tm1Wjl^/SzJ (NSG) mice for the MDA-MB-468 group, and for the C4-2 group, tumor cells were injected subcutaneously on the right flank of 7-9 weeks old male NSG mice. Using a vernier caliper, the tumor length (L) and width (W) were measured twice per week, and the tumor volume was calculated as (L x W x W)/2. At the end of the study, primary tumors were surgically removed, and their final weight was recorded.

### Putative nuclear localization signal (NLS) prediction

cNLS mapper software was used for predicting the putative nuclear localization signal (NLS) of metabolic enzymes^48–50^. The amino acid sequence of ACO2 (NM_001098), IDH2 (NM_002168), and CS (NM_004077) was analyzed using the cNLS mapper with a cut-off score ≥ 4. NLS prediction restricted to 60 amino acid on N- and C- terminal regions.

### Schematics

All the schematics were generated using Biorender Software.

### Statistical information

All data are represented as mean ± s.d. unless otherwise indicated, and statistical comparisons between different groups were performed using one-way or two-way ANOVA with appropriate multiple comparison corrections. For all statistical analyses, differences of *P*  ≤  0.05 were considered statistically significant. At least three different samples with similar results were reported. Independent samples in Figs. 1–6, 7a–c, 7f–i and Supplementary Figs. S1–8, S9a–c, refer to different wells/plates of cells cultured simultaneously or separately, and processed as part of the same experiment. GraphPad Prism software version 9.0 was used for data analysis.

### Reporting summary

Further information on research design is available in the Nature Portfolio Reporting Summary linked to this article.

## Supplementary information

Supplementary Information

Description of Additional Supplementary Files

Supplementary Data 1

Reporting Summary

Transparent Peer Review file

## Source data

Source Data

## Data Availability

The ATAC-seq data (C4-2 cells) generated in this study have been deposited in the Gene Expression Omnibus (GEO) database under accession code GSE301090. The RNA-seq data generated in this study have been deposited in the Gene Expression Omnibus (GEO) database under accession code GSE301089. The ATAC-seq data (MDA-MB-231 cells) generated in this study have been deposited in the Gene Expression Omnibus (GEO) database under accession code GSE301095. The Mass Spectrometry raw data (IP ACO2 and IDH2) generated in this study have been deposited in the massIVE repository under accession code MSV000099250[https://massive.ucsd.edu/ProteoSAFe/private-dataset.jsp?task=44dd358087cf4cd48f269ff4b81785a8]. The Mass Spectrometry raw data and the Xcel file with the quantitative analysis of 13 C labeling generated in this study have been deposited to the massIVE repository under accession code MSV000099224[https://massive.ucsd.edu/ProteoSAFe/private-dataset.jsp?task=bc2259d87bb543cd8dc524ce89bb835f]. The HeLa-S3 ChIP–seq datasets used in this study are available in the GEO database under accession codes IgG: GSM935339, and KAT2A: GSM935302. The remaining data are available within the Article, Supplementary Information or Source Data file Source data are provided in this paper.
