# Supplementary material for: Nuclear-specific reductive carboxylation of alpha-ketoglutarate fuels histone acetylation to induce chromatin accessibility and gene activation
Source: Nat Commun. 2026 Jul 1;17:8216. doi: 10.1038/s41467-026-74786-3 (PMC13462710; doi:10.1038/s41467-026-74786-3)

Supplementary Information:

Nuclear-specific reductive carboxylation of alpha-ketoglutarate fuels histone acetylation to induce chromatin accessibility and gene activation

Abhisha Sawant Dessai[1,8], Nadya A. Elhalawany[1], Tao Dai[1,9], Justine J. Jacobi[1], Christian Prechtl[1], Alphonse N. Dimeck[1], Sierra R. Morton[1], Mark D. Long[2], Prashant K. Singh[3], Nagireddy Putluri[4], Mariana Lopes[5], Song Liu[2], Dominic J. Smiraglia[1], Katerina V. Gurova[1], Peder J. Lund[5], Leah A. Gates[6], Sung Yun Jung[4,7], and Subhamoy Dasgupta[1*]

[1]Department of Cell Stress Biology, Roswell Park Comprehensive Cancer Center; Buffalo, NY, 14263, USA.

[2]Department of Biostatistics and Bioinformatics, Roswell Park Comprehensive Cancer Center; Buffalo, NY 14263, USA.

[3]Department of Cancer Genetics & Genomics, Roswell Park Comprehensive Cancer Center; Buffalo, NY 14263, USA.

[4]Department of Molecular and Cellular Biology, Baylor College of Medicine, Houston, TX 77030, USA.

[5]Department of Nutrition, School of Medicine, Case Western Reserve University, Cleveland, OH, USA

[6]Department of Biochemistry, School of Medicine, Case Western Reserve University, Cleveland, OH, USA

[7]Department of Biochemistry and Molecular Pharmacology, Baylor College of Medicine, Houston, TX 77030, USA.

[8]Present Address: Penn Epigenetics Institute, Perelman School of Medicine, University of Pennsylvania, Philadelphia, PA 19104, USA

[9]Present Address: Children's Medical Center Research Institute, University of Texas Southwestern Medical Center, Dallas, TX 75390, USA.

*Corresponding author. Email: Subhamoy.Dasgupta@RoswellPark.org

Supplementary Figures 1-9

Supplementary Table 1

Supplementary Fig. S1

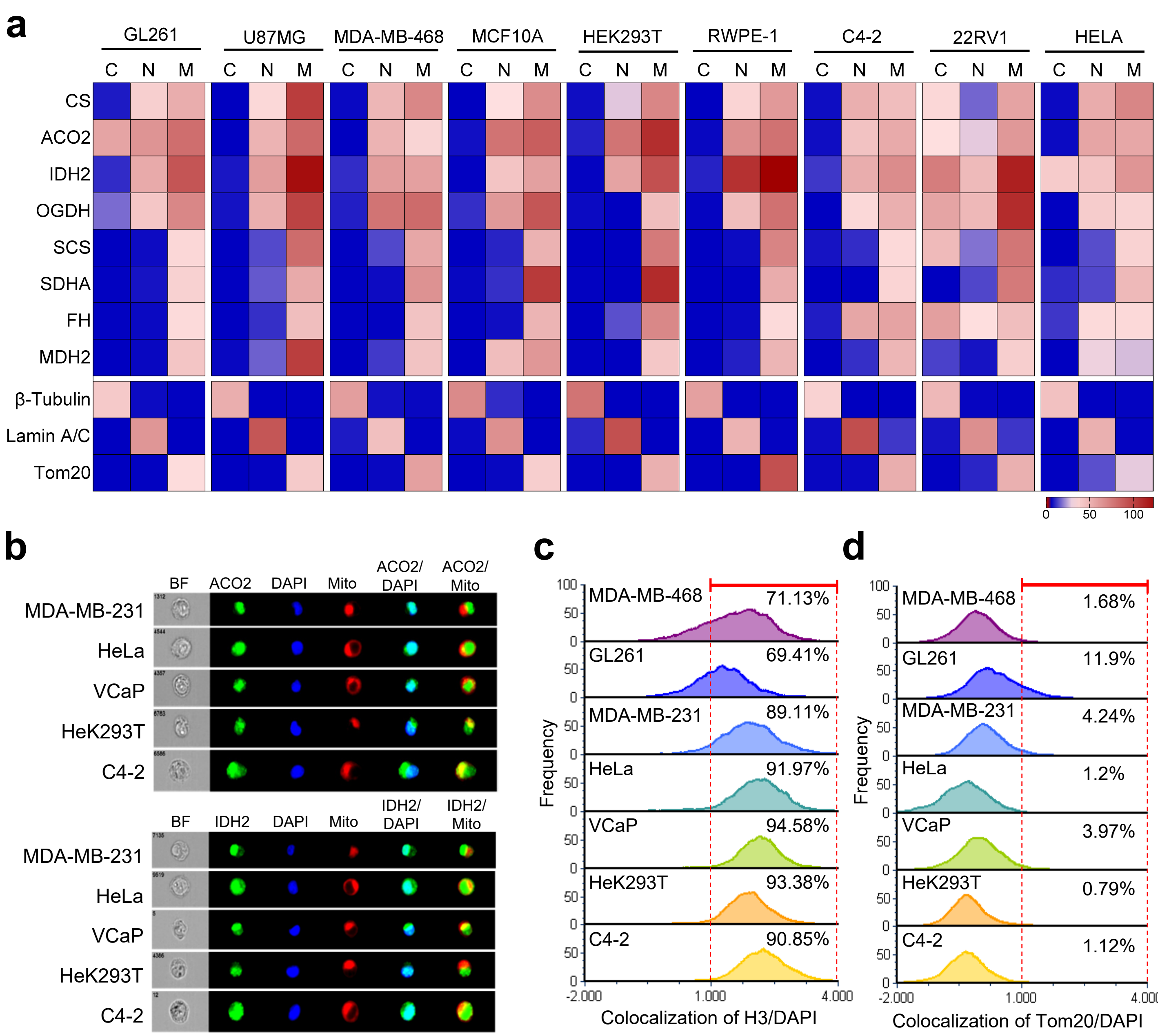

Supplementary Fig. S1: Quantification of the sub-cellular distribution of mitochondrial TCA cycle enzymes.

(**a**) Various mouse and human cell lines were fractionated to separate cytosolic (C), nuclear (N), and mitochondrial (M) proteins, followed by immunoblot analysis of mitochondrial TCA cycle enzymes. β-tubulin, Lamin A/C, and Tom20 were used as cytosolic, nuclear, and mitochondrial markers, respectively. Immunoblots shown in Fig. 1A, were used to quantify the intensities of individual protein bands normalized to fraction-resident proteins β-tubulin, Lamin A/C, and Tom20 to generate a representative heatmap using GraphPad Prism. representative of 3 independent experiments used for quantification.

(**b**) Image-stream (imaging flow cytometry) based immunofluorescence analysis of cells stained with DAPI (Blue), MitoTracker (Deep Red), and ACO2 or IDH2 (Green). Representative images for ACO2 and IDH2 staining in MDA-MB-231, HeLa, VCap, HeK293T and C4-2 are included in the panel. (n = 10,000 cells, for each cell line)

(**c-d**) The Colocalization index of Tom20 (C) or histone H3 (D) with DAPI, at a single-cell resolution, was analyzed using the Amnis IDEAS™ (v6.2) software, as negative and positive controls for Figs. 1A to D. Similarity feature was used to calculate the colocalization score for Tom20 or Histone H3 merging with DAPI. Cells with colocalization scores ≥ 1 were gated as positive cells for nuclear-localized Histone H3 or Tom20. The percentage of cells with colocalization scores ≥ 1 is shown on the right. (n = 10,000 cells imaged for each cell line, representative of two independent experiments)

Source data are provided as a Source Data file.

Supplementary Fig. S2

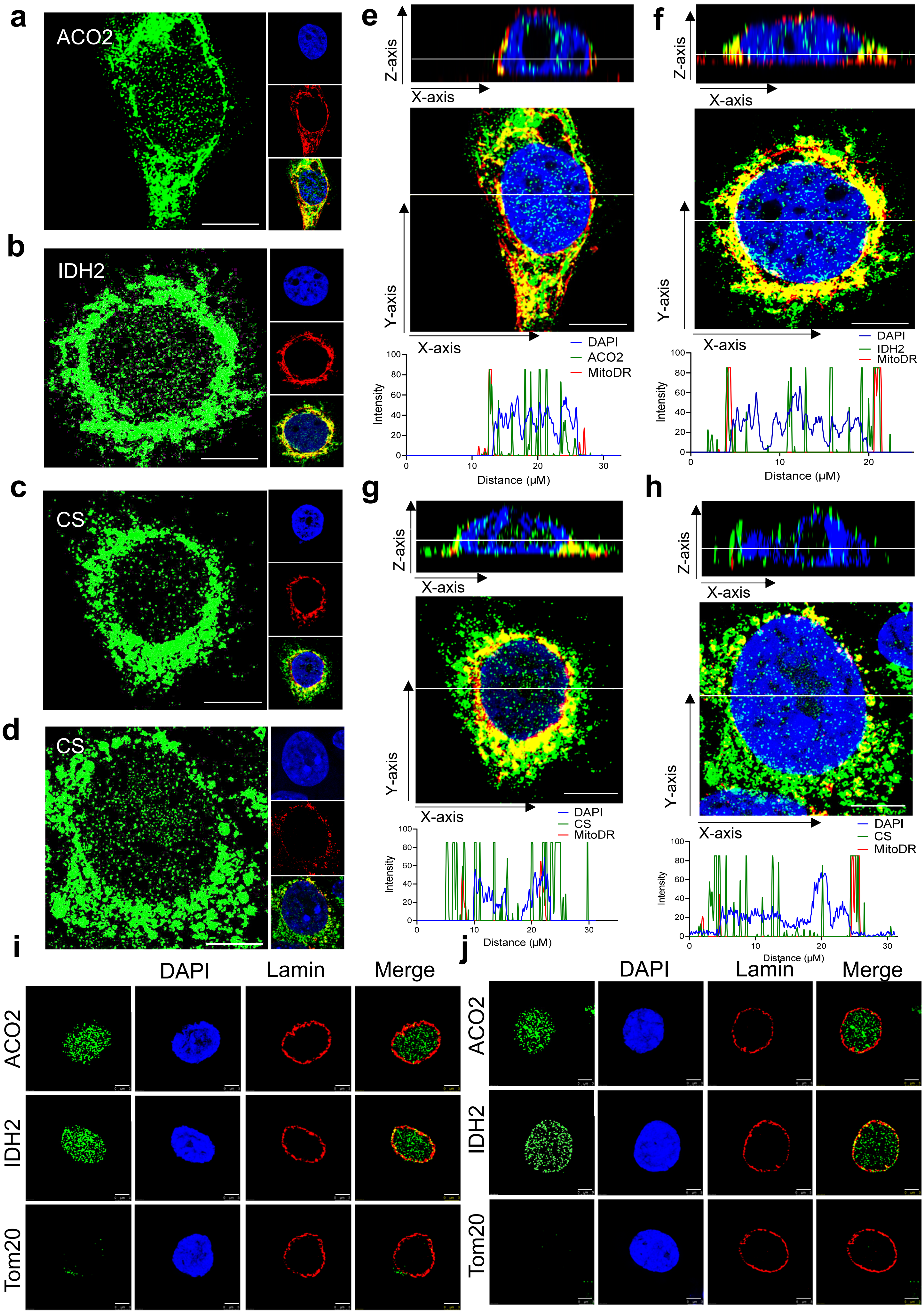

Supplementary Fig. S2: High resolution confocal microscopy revealed nuclear metabolic enzymes appear as distinct spots or speckles.

(**a-d**) Representative images obtained from confocal microscopy of immunofluorescence staining with antibodies (green): ACO2, IDH2, or CS in MDA-MB-468 and GL261 cells, co-stained with DAPI (Blue) and MitoTracker (MitoDR- Red). Scale bar 8μm. Representative fields from 3 independent experiments.

(**e-h**) Z-stack analysis of the confocal images stained for ACO2 (E), IDH2 (F), or CS (G-H), co-stained with DAPI (Blue) and MitoDR (Red) in MDA-MB-468 (E-G) and GL261 (H) cells. Cells were scanned at 0.2 μm increments along the z-axis using a confocal microscope. ImageJ software was used to analyze immunofluorescence intensity at the mid-plane XY-axis of the cells (bottom graph). Scale bar 8μm. Representative fields from 3 independent experiments.

(**i-j**) Representative images obtained from confocal microscopy of isolated intact nuclei from MDA-MB-468 (I) and HeLa (J) cells immunostained with ACO2, IDH2, or Tom20 (Green) antibodies, along with nuclear marker (DAPI - blue) and nuclear membrane marker (Lamin A/C – red). Scale bar 3μm. Representative fields from 3 independent experiments.

Source data are provided as a Source Data file.

Supplementary Fig. S3

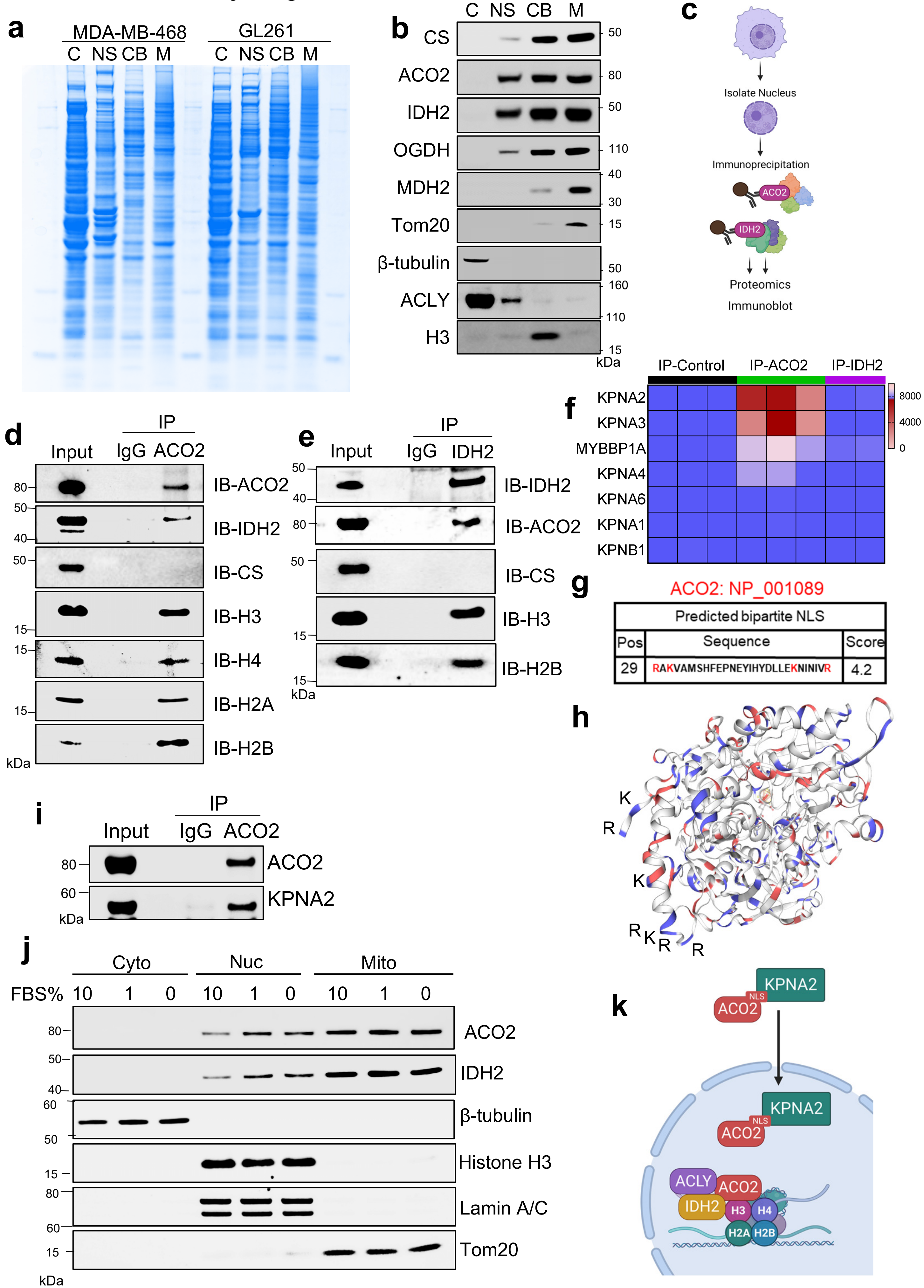

Supplementary Fig. S3: Nuclear translocation of ACO2 is mediated by importin-α, where it associates with chromatin and forms a nuclear metabolic complex with IDH2.

(**a**) Coomassie blue-stained gel showing the total protein loaded for each sub-cellular fractions isolated from MDA-MB-468 and GL261 cells analyzed in Figs. 2B and Fig. S3B. Cytosolic (C), nuclear soluble (NS), chromatin-bound (CB), and mitochondrial (M) fractions. Representative of 3 independent experiments.

(b) GL261 cells were fractionated followed by immunoblot analysis for TCA cycle enzymes. β-tubulin, Histone H3, and Tom20 were used as cytosolic, chromatin-bound, and mitochondrial markers, respectively. Representative of 3 independent experiments.

(**c**) Schematic depicting immunoprecipitation of ACO2 or IDH2 followed by LC-MS based proteomics or immunoblot analysis was created in BioRender. https://biorender.com/dtp0huv

(**d-e**) Immunoprecipitation of ACO2 **(d)** or IDH2 **(e)** from nuclear lysates of GL261, followed by immunoblotting with IDH2, ACO2, CS, and histone subunits H2A, H2B, H3, and H4 antibodies. Input represents 0.5% of the total protein and IgG was used as an isotype control. Representative of 3 independent experiments.

(**f**) Heatmap of IP-MS data showing relative enrichment of nuclear import complex proteins compared to control (pre-clear) samples from GL261 cells. n = 2-3 independent samples per group.

(**g**) cNLS mapper was used to predict nuclear localization signal (NLS) on ACO2 protein. NLS score cut-off ≥ 4 was used with NLS prediction restricted to 60 amino acid on N- and C- terminal regions.

(**h**) ACO2 protein structure highlighting charged amino acids (blue – positively charged, red – negatively charged). Positively charged amino acids within the predicted NLS sequence are labelled; K: lysine, R: arginine.

(**i**) Immunoprecipitation of ACO2 from nuclear lysates isolated from GL261 cells followed by immunoblotting for importin-alpha subunit 1 (KPNA2). IgG was used as an isotype control. Representative of 3 independent experiments.

(j) Immunoblot showing the relative levels of ACO2 and IDH2 in cytoplasm, nucleus, and mitochondria upon serum (FBS) starvation (0%) or limited FBS (1%) compared to completed serum (10% FBS) for 24hrs. β-tubulin, Lamin A/C, and Tom20 were used as marker proteins. Representative of 2 independent experiments.

(**k**) Schematic showing KPNA2 dependent nuclear translocation of ACO2 was created in BioRender. https://biorender.com/ejshnog

Source data are provided as a Source Data file.

Supplementary Fig. S4

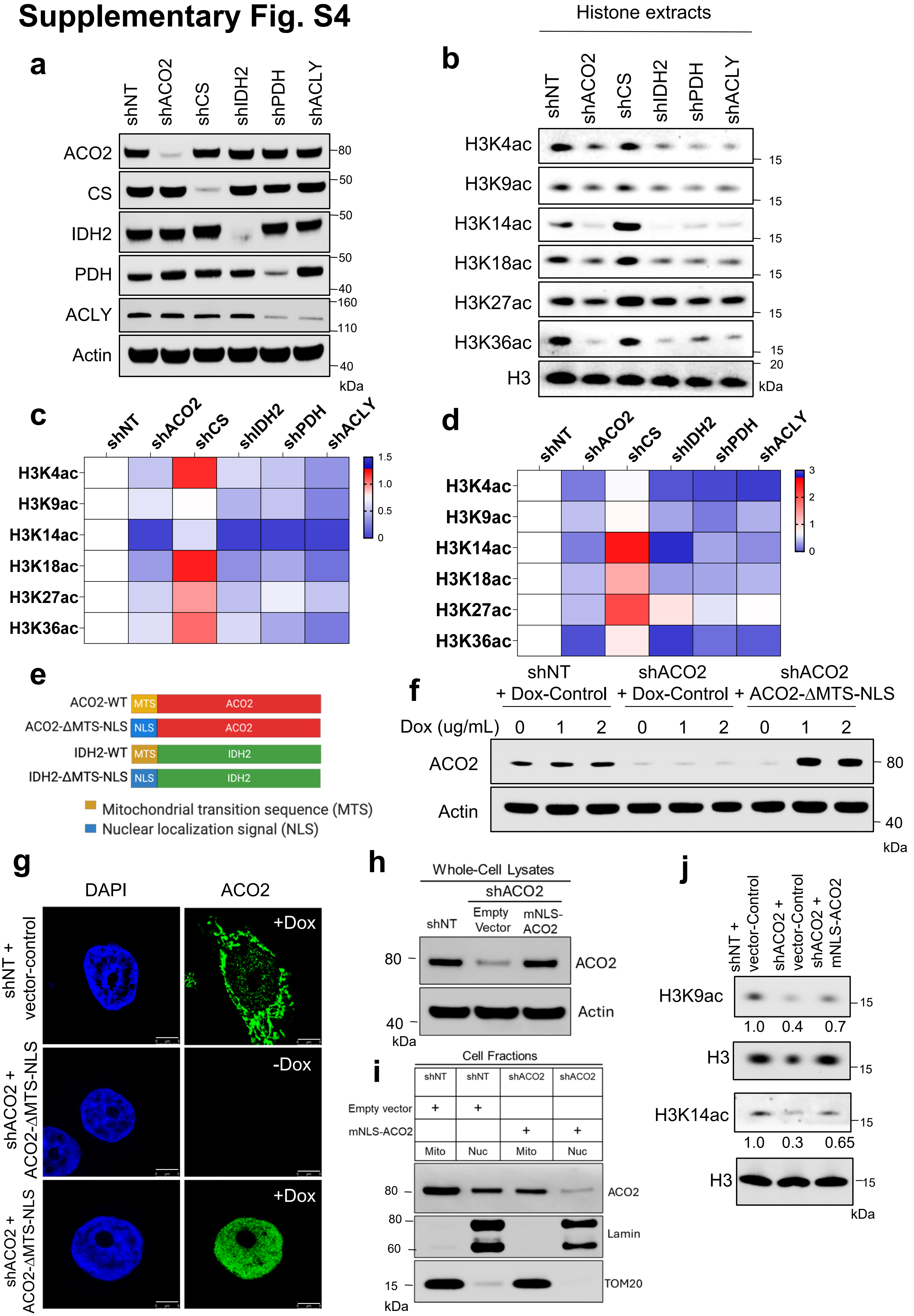

Supplementary Fig. S4: Nuclear localized metabolic enzymes control histone H3 acetylation.

(**a**) Immunoblots showing knockdown efficiency of shACO2, shCS, shIDH2, shPDH, and shACLY compared to control shNT in GL261 cells. Actin was loading control. Representative of 3 independent experiments.

(**b**) Immunoblot analysis of acid extracted histones from GL261 shNT control, shACO2, shCS, shIDH2, shPDH, and shACLY knockdown cell lines. Histone H3 was loading control. Representative of 3 independent experiments.

(**c-d**) Heatmap showing quantification of the band intensities of histone acetylation marks that were normalized to total histone H3 levels for GL261 **(C)** and MDA-MB-468 **(D)** cell lines. Representative of 3 independent experiments.

(**e**) Schematic showing mitochondrial transition sequence (MTS) of ACO2 and IDH2 was replaced with nuclear localization signal (NLS) to generate ∆MTS-NLS mutants was created in BioRender. https://biorender.com/0o6xuxx

(**f**) Immunoblot analysis showing doxycycline (dox) inducible expression of ACO2-∆MTS-NLS mutant in shACO2-expressing MDA-MB-468 cells. Actin was loading control. Representative of 2 independent experiments.

(**g**) Immunofluorescence microscopy was performed after 4 days of +/- doxycycline (1µg/mL) treatment ($n=3$ biological replicates). Representative fields from 3 independent experiments.

(h) Bipartite NLS on ACO2 as shown in Supplementary Fig. S3g, containing lysine (K) and arginine (R) residues were mutated to alanine (A) to create mutated-ACO2-NLS (mNLS) and the immunoblot showing levels of ACO2. Actin was loading control. Representative of 3 independent experiments.

(i) Immunoblot showing levels of ACO2 (wild type) and ACO2-mNLS in mitochondria and nucleus. Lamin A/C and Tom20 were used as marker proteins. Representative of 3 independent experiments.

(j) Immunoblots showing levels of H3K9Ac, H3K14ac, compared to total H3 in MDA-MB-468 cells expressing shNT (control), shACO2, or shACO2+ACO2-mNLS as shown in **(h)** Representative of 3 independent experiments.

Source data are provided as a Source Data file.

Supplementary Fig. S5

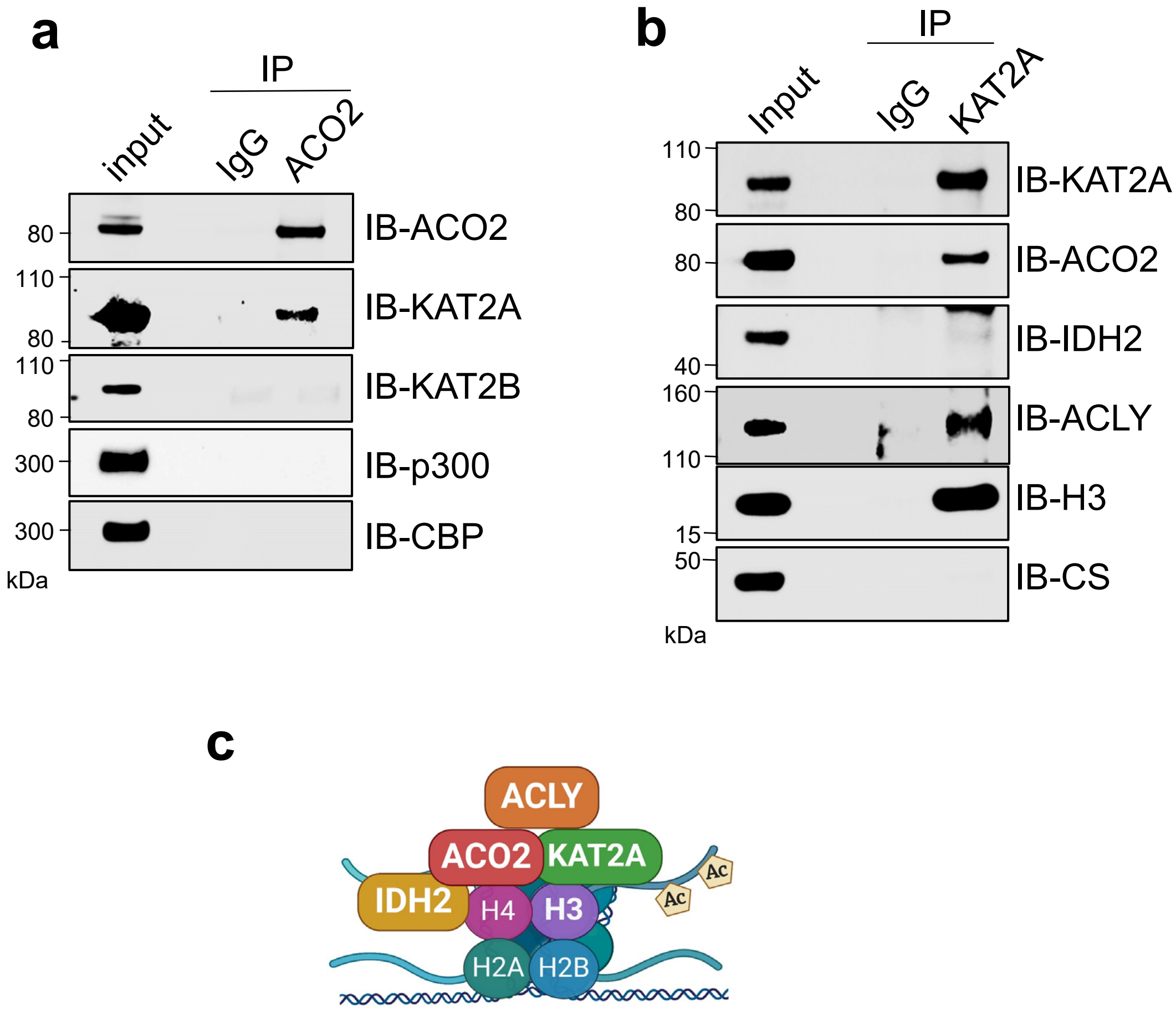

Supplementary Fig. S5: Metabolic enzymes ACO2, IDH2, and ACLY form a nuclear metabolic complex with KAT2A on the chromatin.

(**a**) Immunoprecipitation (IP) of endogenous ACO2 followed by immunoblotting with KAT2A, p300, and CBP antibodies. Input represents 0.5% of total protein. Representative of 3 independent experiments.

(**b**) IP of KAT2A from GL261 nuclear lysates followed by immunoblotting with ACO2, IDH2, ACLY, CS, and H3, antibodies compared to control (IgG). Input represents 0.5% of the total protein. Representative of 3 independent experiments.

(c) Schematic summarizing the working hypothesis indicating ACO2, IDH2, and ACLY form nuclear metabolic complex (NMC) on chromatin was created in BioRender. https://biorender.com/6mfvaxj

Source data are provided as a Source Data file.

Supplementary Fig. S6

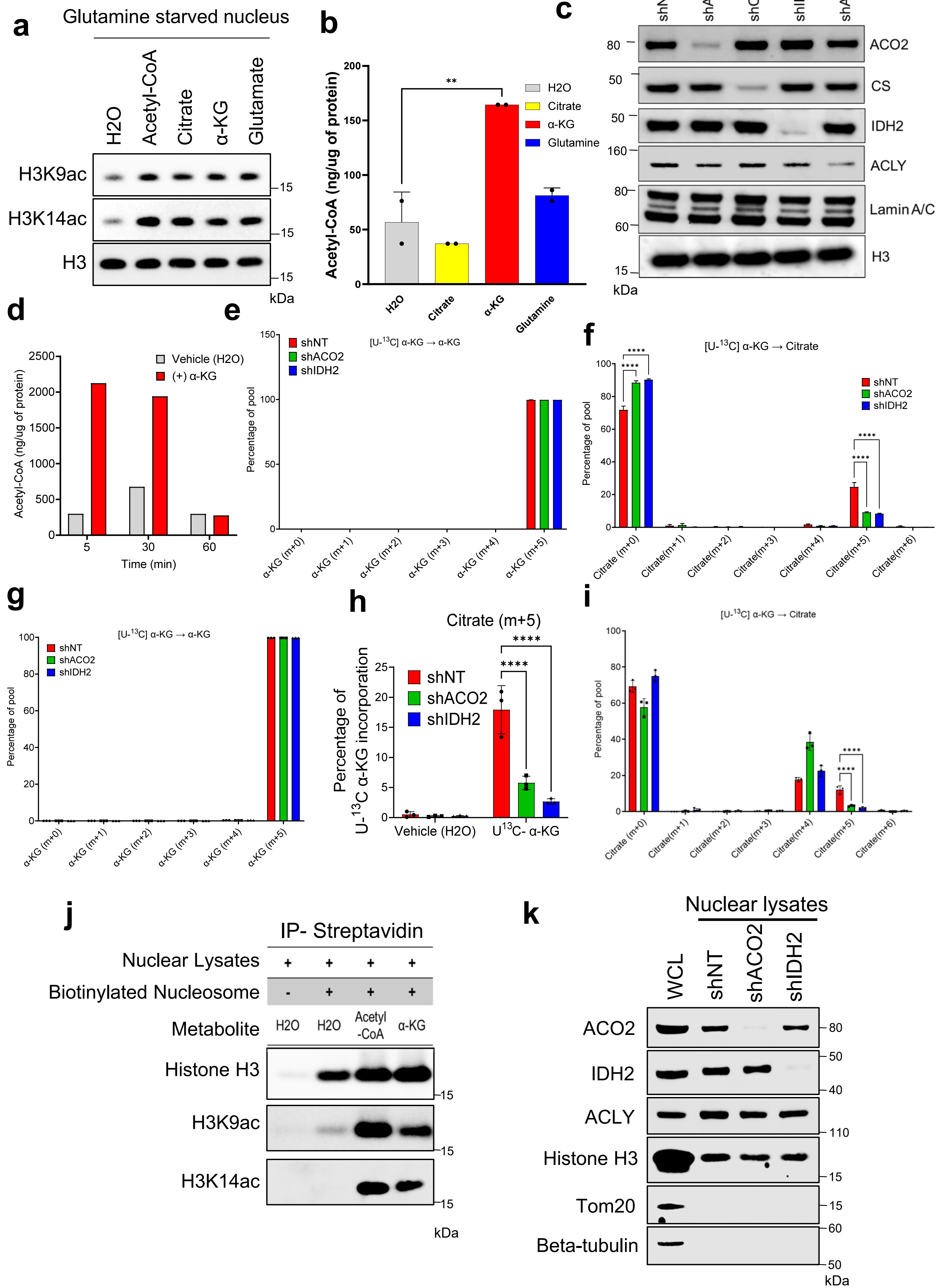

Supplementary Fig. S6: Isotope tracing studies revealed IDH2 and ACO2 utilize alpha-ketoglutarate (αKG) to generate citrate in the nucleus.

(a) Immunoblots showing levels of H3K9ac and H3K14ac in isolated intact nuclei from glutamine-starved GL261 cells incubated with control (H_2O), acetyl-CoA (5μM), citrate (2mM), and α-KG (2mM). Total histone H3 was loading control. Representative of 2 independent experiments.

(b) Concentration of acetyl-CoA normalized to total nuclear protein in isolated nuclei from glutamine-starved GL261 cells treated with H2O (control), citrate (2mM), alpha-ketoglutarate (2mM) and glutamine (2mM). *n*=2 independent samples per group. **P < 0.001, *P* values were calculated by ordinary one-way ANOVA with Dunnett's multiple comparisons test.

(c) Immunoblots showing levels of ACO2, CS, IDH2 and ACLY used in Fig. 4c. Lamin A/C and histone H3 are used as a loading control. Representative of 3 independent experiments.

(d) Concentration of acetyl-CoA normalized to total nuclear protein in isolated nuclei from glutamine-starved GL261 cells treated with H2O (control) or alpha-ketoglutarate (2mM). *n=3* technical replicates per group.

(e-g) Mass isotope distribution of α-KG **(e, g)** and citrate **(f)** in isolated nuclei from MDA-MB-468 **(e, f)** and GL261 **(g)** cells expressing shNT, shACO2, or shIDH2 treated with U-$^{13}C_5$-α-KG (2mM). *n=3 independent samples* per group. m is nominal mass. *n=3* biological replicates, **** P ≤ 0.0001, P-values were calculated by two-way ANOVA with Tukey's multiple comparisons test. Error bars are presented as mean ± SD.

(**h-i**) Isolated nuclei from glutamine-starved GL261 cells expressing shNT, shACO2, and shIDH2 were incubated with U-$^{13}C_5$-α-KG (2mM) followed by LC-MS analysis to quantify the relative enrichment of C^{13} isotopes in citrate (m+5) **(h)** and mass isotope distribution of citrate **(i)**. *n=3* independent samples per group, **** P < 0.0001, P-values were calculated by two-way ANOVA with Tukey's multiple comparisons test. Error bars are presented as mean ± SD.

(**j**) Immunoblots showing levels of H3K9ac and H3K14ac following *in vitro* HAT with biotinylated nucleosomes and nuclear lysates from MDA-MB-468. Total histone H3 was loading control. Representative of 2 independent experiments.

(**k**) Immunoblot showing levels of ACO2, IDH2 and ACLY compared to shNT (control) and whole cell lysates (WCL). Total histone H3 was loading control. Representative of 2 independent experiments.

Source data are provided as a Source Data file.

Supplementary Fig. S7

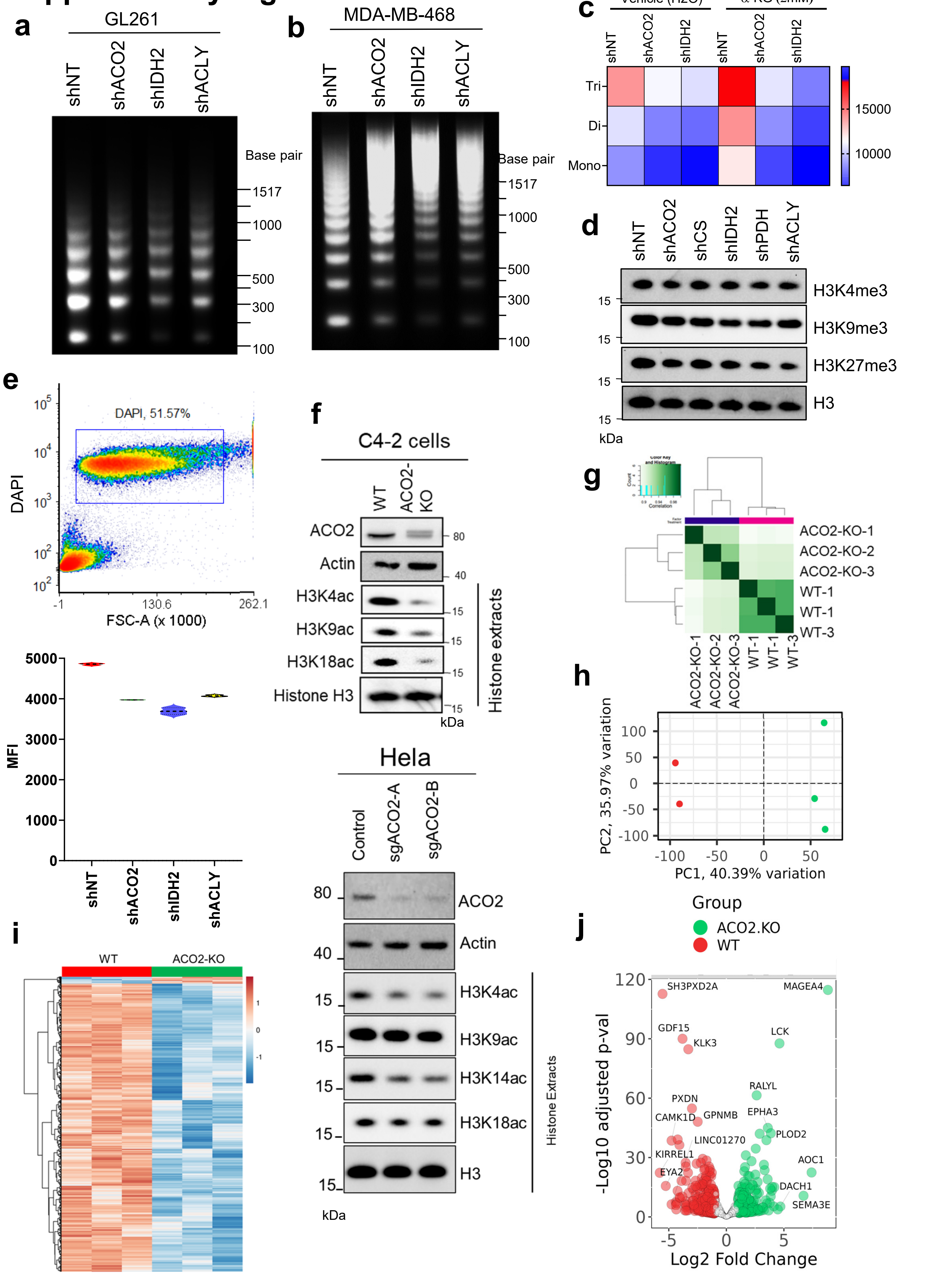

Supplementary Fig. S7: Loss of ACO2 decreases chromatin accessibility with altered transcriptomic profile.

(**a-b**) MNase digestion assay was performed using nuclei isolated from GL261 (a) or MDA-MB-468 (b) cells expressing shNT, shACO2, shIDH2, and shACLY, and the digested DNA was separated by agarose gel to detect the relative levels of mono-, di-, tri-, and tetra- nucleosome fragments as marked on the gel. Representative of 3 independent experiments.

(**c**) Heatmap showing the quantification of mono-, di-, tri- and tetra-nucleosome fragments levels shown in Fig. 5a. (*n=2,* independent samples per group)

(**d**) Immunoblot analysis to measure relative levels of H3K4me3, H3K9me3, and H3K27me3 in histone extracts isolated from GL261 cells expressing shNT, shACO2, shCS, shIDH2, shPDH, and shACLY. Histone H3 was used as a loading control. (Representative of 2 independent experiments)

(e) Graph showing the percentage of DAPI-positive nuclei used for measuring the nascent-RNA synthesis by 5-ethynyl uridine (EU) incorporation in GL261 cells. (n=25,000 nuclei per sample). Graph showing mean fluorescence intensity (MFI) of nascent RNA quantified in shNT, shACO2, and shIDH2 expressing cells treated with α-KG (2mM) (Representative of 2 independent experiments.).

(**f**) Acid extracted histones from C4-2 (top) and Hela (bottom) cells with ACO2 knockout (CRISPR-Cas9/sgRNA) were used for immunoblotting analysis to measure the relative levels of histone H3 acetylation marks compared to control (ACO2-WT). Total histone H3 was used as a loading control. (Representative of 2 independent experiments)

(g) Cluster heatmap showing the correlation between WT vs ACO2-KO samples used for ATAC sequencing (GSE301090), *n=3,* independent samples per group.

(**h**) Principal component analysis (PCA) analysis of RNAseq (GSE301089) data obtained from WT (n=2) and ACO2-KO (n=3), independent samples per group.

(**i**) Heatmap showing the enrichment of differentially accessible regions (DAR) (FDR≤0.05) obtained from ATAC sequencing (GSE301090) of WT and KO samples. *n=3,* independent samples per group

(**j**) Volcano plot showing differentially enriched genes (DEG) obtained from RNAseq (GSE301089) analysis of ACO2-KO compared to WT samples. WT (n=2) and ACO2-KO (n=3), independent samples per group.

Source data are provided as a Source Data file.

Supplementary Fig. S8

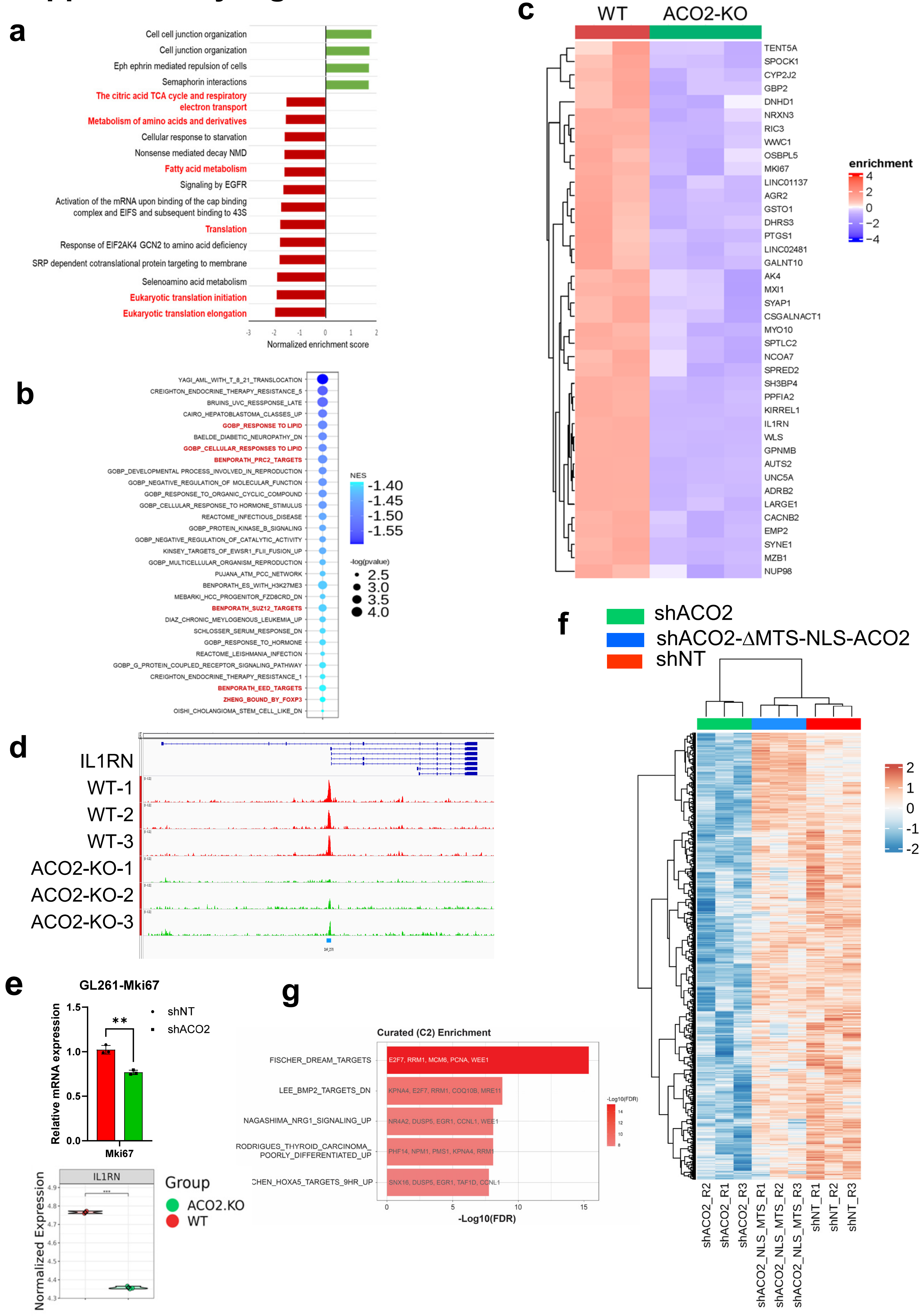

Supplementary Fig. S8: Pathway analysis identified essential target genes required for proliferation and growth.

(**a**) Gene set enrichment analysis (GSEA) using REACTOME was performed on significant DEGs between WT vs ACO2-KO cells. The plot shows the normalized enrichment score (NES) of top significantly enriched REACTOME pathways in C4-2-WT (Red) and C4-2-ACO2-KO (Green), (p-value < 0.01, gene set size > 25).

(**b**) Gene set enrichment analysis (GSEA) of ATAC-seq DARs (GSE301090) showing curated enriched pathways in ACO2-KO compared to WT cells. *n=3,* independent samples per group

(**c**) Heatmap showing normalized mRNA expression of 40 overlapping targets between ATAC-seq (GSE301090) and RNA-seq (GSE301089) obtained by analyzing WT vs ACO2-KO samples. ATAC-seq targets with loss > 1.5 within 5kb of promoter as well as significantly reduced mRNA expression levels, using p.adj < 0.05 in ACO2-KO vs WT.

(**d**) Integrative Genomics Viewer (IGV) based visualization of IL1RN ATAC-seq (GSE301090) peaks in WT (red) and ACO2-KO (green) samples.

(**e**) Relative mRNA expression of Mki67 in GL-261 cells expressing shNT and shACO2 (n=3, independent samples per group). Error bars are presented as mean ± SD. ** p ≤ 0.01 calculated by two-way ANOVA with Tukey's multiple comparisons test. RNA-seq based normalized gene expression levels of IL1RN in WT. ***p < 0.001.

(f) ATAC-seq (GSE301095) was performed using MDA-MB-468 cells expressing shNT, shACO2, shACO2 + ACO2-ΔMTS-NLS (*n=3,* independent samples per group). Heatmap showing functional role of nuclear ACO2 on chromatin accessibility. Chromatin regions (n = 1687; accessibility loss > 1.5FC) that were lost due to ACO2 depletion (shACO2) compared to control (shNT) were restored by ACO2-ΔMTS-NLS expression.

(**g**) GSEA (curated C2 gene sets) analysis of significant overlapping targets between the KAT2A ChIP-seq dataset (GEO accession numbers: IgG: GSM935339, KAT2A: GSM935302) and significant ATAC-seq DARs lost in ACO2-KO compared to WT (loss > 1.5 within 5kb of promoter). A list of the top 5 significant GSEA pathways highlighting top targets within the pathway is shown.

Source data are provided as a Source Data file.

Supplementary Fig. S9

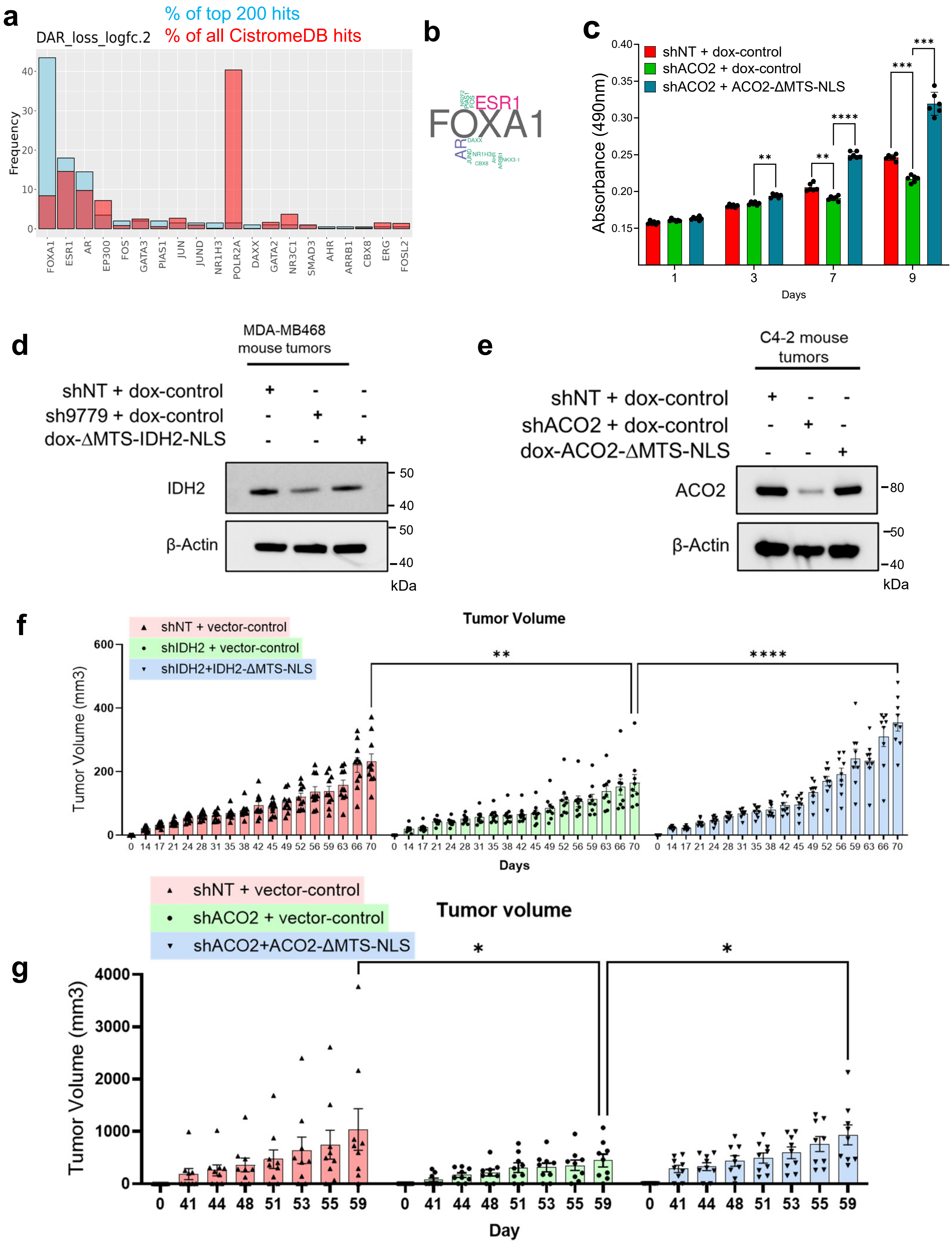

Supplementary Fig. S9: Nuclear specific rescue of IDH2 and ACO2 drives hyper proliferative phenotype in multiple mouse models of cancer.

(a) Bar graph highlighting the frequencies of top transcription factors observed through GIGGLE analysis after overlapping the complete CistromeDB dataset with significant ATAC-seq (GSE301090) DARs lost in ACO2-KO compared to WT (loss > 1.5 within 5kb of promoter). (*n=3,* independent samples per group)

(b) Word cloud depicting the frequencies of the top transcription factors observed in the top 200 hits overlapping between the complete CistromeDB dataset with significant ATAC-seq DARs lost in ACO2-KO compared to WT.

(**c**) Graphical representation of cellular proliferation rate of MDA-MB-468 cells expressing, shNT + vector-control, shACO2 + vector-control, and shACO2 + ACO2-ΔMTS-NLS (n=6, *over two* independent experiments) on days 1, 3, 7, and 9. Cells were kept under doxycycline 1µg/mL for ACO2 rescue. Error bars are presented as mean ± SD. ****P ≤ 0.0001, ***P ≤ 0.001, ** P ≤ 0.01. P-values were calculated by two-way ANOVA with Tukey's multiple comparisons test.

(d) Immunoblotting showing the expression of IDH2 in mouse tumors generated by implanting MDA-MB468 cells with shNT, shIDH2 and shIDH2+ IDH2-ΔMTS-NLS (n=3, independent samples per group were pooled). Actin was used as a loading control.

(e) Immunoblotting showing the expression of ACO2 in mouse tumors generated by implanting C4-2 cells with shNT, shACO2 and shACO2+ ACO2-ΔMTS-NLS (n=3, independent samples per group were pooled). Actin was used as a loading control.

(f) Tumor growth was measured each week after implanting MDA-MB468 cells with shNT (*n = 10)*, shIDH2 (*n = 9)* and shIDH2+ IDH2-ΔMTS-NLS *(n = 9)* and tumor volume calculated in mm^3. ** P = 0.0011, **** P < 0.0001 were calculated by ordinary 2-way ANOVA with Tukey's multiple comparisons test. Error bars are presented as mean ± SEM.

(g) Tumor growth was measured each week after implanting C4-2 cells with shNT, shIDH2 and shIDH2+ IDH2-ΔMTS-NLS (n=9 mice per group) and tumor volume calculated in mm^3. *P < 0.05, P values were calculated by ordinary 2-way ANOVA with Tukey's multiple comparisons test. Error bars are presented as mean ± SEM.

Source data are provided as a Source Data file.

Table S1: List of shRNA

Gene	Mature Antisense	Clone-ID	Region	Species	Plasmid Backbone
ACLY	AAGATTTGGCTTCTTCGAG	V2LHS_94212	CDS	Human	pGIPZ
ACO2	AGATTGGCAATGTCTTCCC	V3LHS_340894	CDS	Human	pGIPZ
CS	TATGTCTTTCAAATTCGTG	V2LHS_172874	CDS	Human	pGIPZ
IDH2	AAGTCTGTGGCCTTGTACT	V2LHS_133275	CDS	Human	pGIPZ
PDHA1	TATAAAGTCAGGCAGACCT	V2LHS_11335	CDS	Human	pGIPZ
ACLY	GAAGGCAAGATCCTCATCATT	TRCN0000055216	CDS	Mouse	pLKO.1-puro
ACO2	CCCATGCAACTGTATTTATTT	TRCN0000114531	3UTR	Mouse	pLKO.1-puro
CS	GCACCCAACATTTGAGTTATT	TRCN0000075858	3UTR	Mouse	pLKO.1-puro
IDH2	AGCTCCAGTGGGTACTCTGTA	TRCN0000428288	3UTR	Mouse	pLKO.1-puro
IDH2	CCTCTCTGGAGGCCTTTCTAG	TRCN0000229779	3UTR	Human	pLKO.1-puro
KPNA2	GTGTTCTCATAGATTTGTCTT	TRCN0000093514	3UTR	Mouse	pLKO.1-puro
KPNA2	TCATGTAGCTGAGACATAAAT	TRCN0000293910	3UTR	Human	pLKO.1-puro
PDHA1	TCAGATCTTTGAAGCTTACAA	TRCN0000325912	CDS	Mouse	pLKO.1-puro

Supplementary Table 1: List of shRNAs

Gene	Mature Antisense	Clone-ID	Region	Species	Plasmid Backbone
ACLY	AAGATTTGGCTTCTTCGAG	V2LHS_94212	CDS	Human	pGIPZ
ACO2	AGATTGGCAATGTCTTCCC	V3LHS_340894	CDS	Human	pGIPZ
CS	TATGTCTTTCAAATTCGTG	V2LHS_172874	CDS	Human	pGIPZ
IDH2	AAGTCTGTGGCCTTGTACT	V2LHS_133275	CDS	Human	pGIPZ
PDHA1	TATAAAGTCAGGCAGACCT	V2LHS_11335	CDS	Human	pGIPZ
ACLY	GAAGGCAAGATCCTCATCATT	TRCN0000055216	CDS	Mouse	pLKO.1-puro
ACO2	CCCATGCAACTGTATTTATTT	TRCN0000114531	3UTR	Mouse	pLKO.1-puro
CS	GCACCCAACATTTGAGTTATT	TRCN0000075858	3UTR	Mouse	pLKO.1-puro
IDH2	AGCTCCAGTGGGTACTCTGTA	TRCN0000428288	3UTR	Mouse	pLKO.1-puro
IDH2	CCTCTCTGGAGGCCTTTCTAG	TRCN0000229779	3UTR	Human	pLKO.1-puro
KPNA2	GTGTTCTCATAGATTTGTCTT	TRCN0000093514	3UTR	Mouse	pLKO.1-puro
KPNA2	TCATGTAGCTGAGACATAAAT	TRCN0000293910	3UTR	Human	pLKO.1-puro
PDHA1	TCAGATCTTTGAAGCTTACAA	TRCN0000325912	CDS	Mouse	pLKO.1-puro

Supplementary Fig. S3b

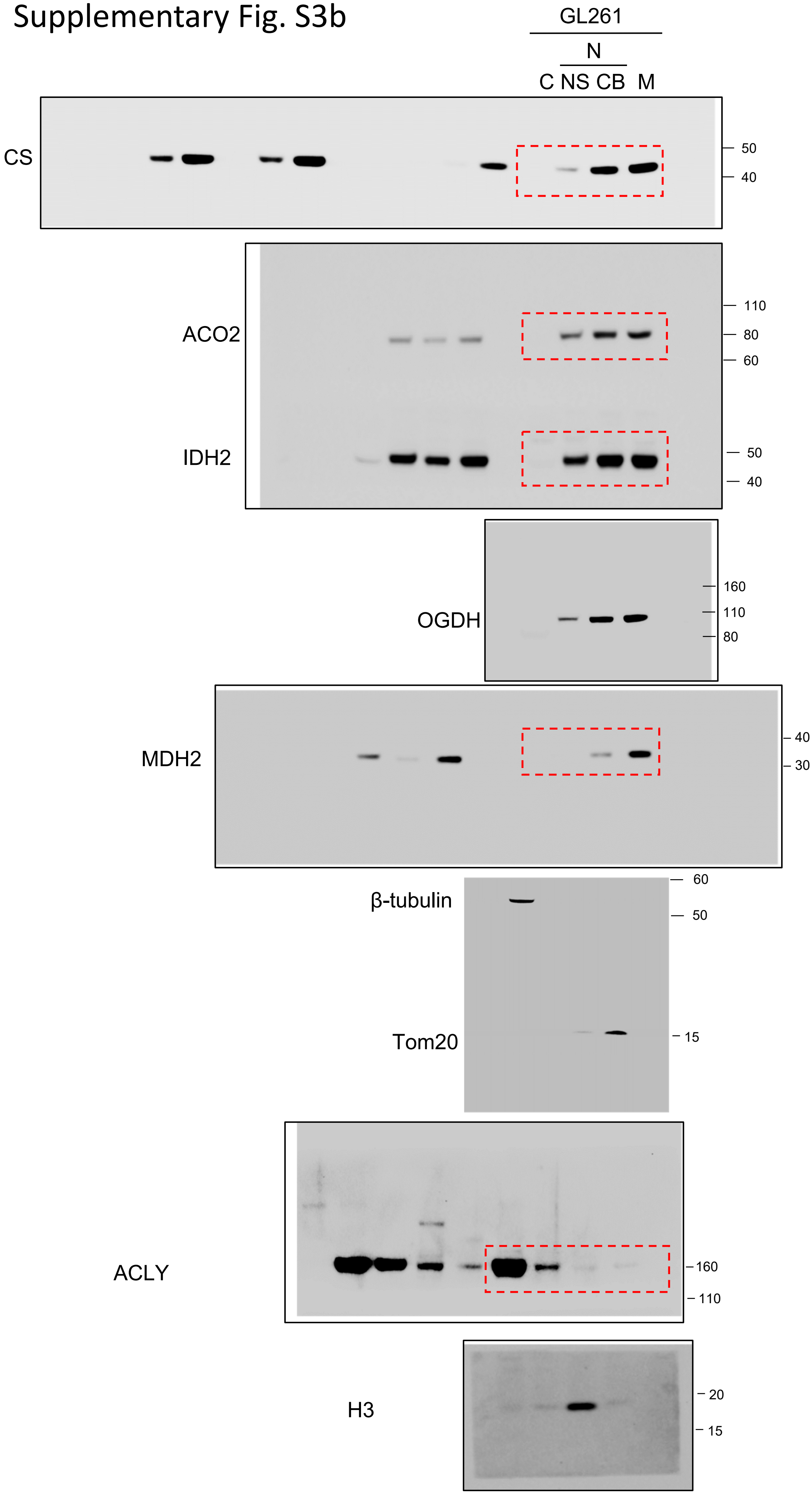

Supplementary Fig. S3d

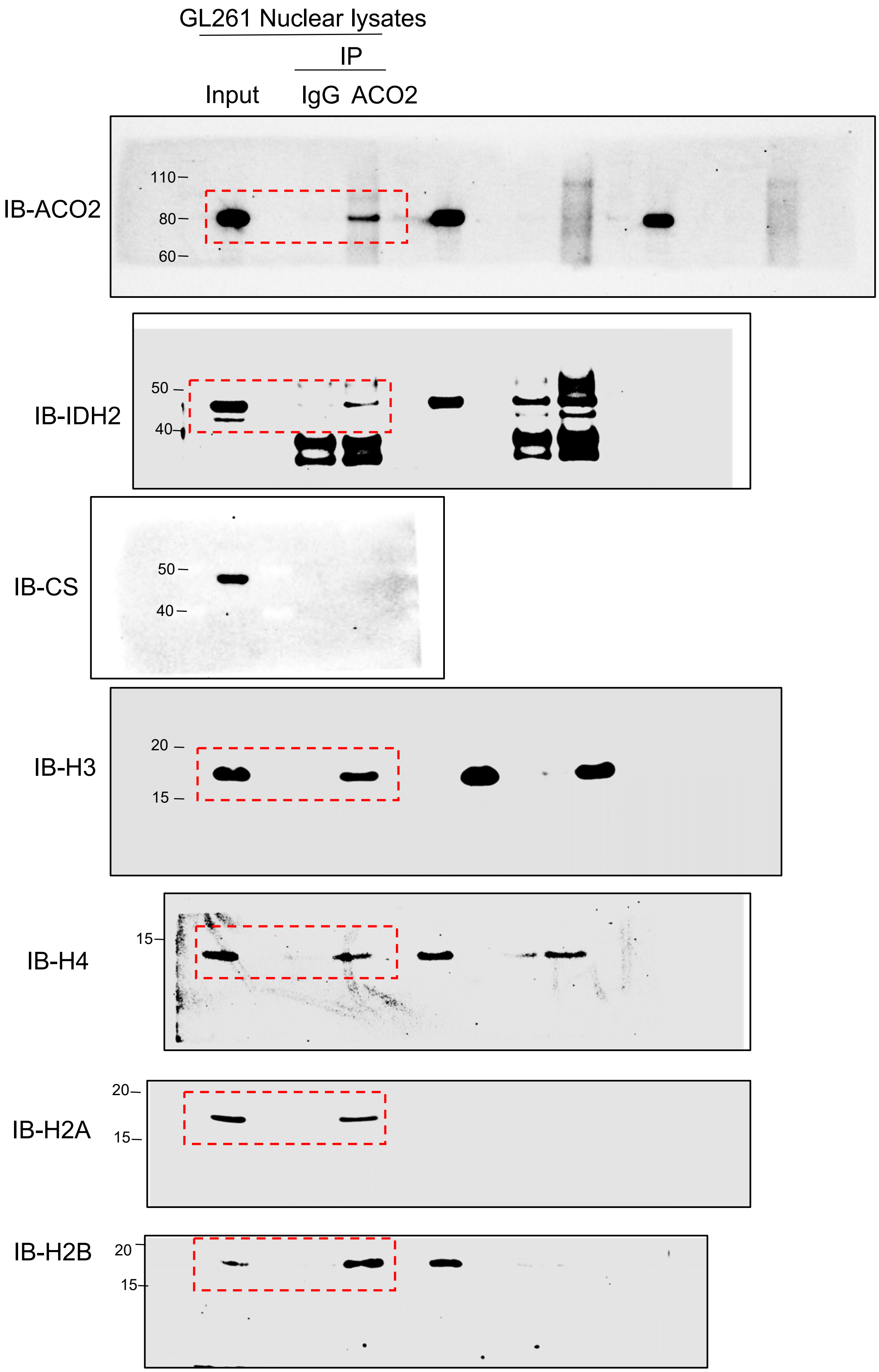

Supplementary Fig. S3e

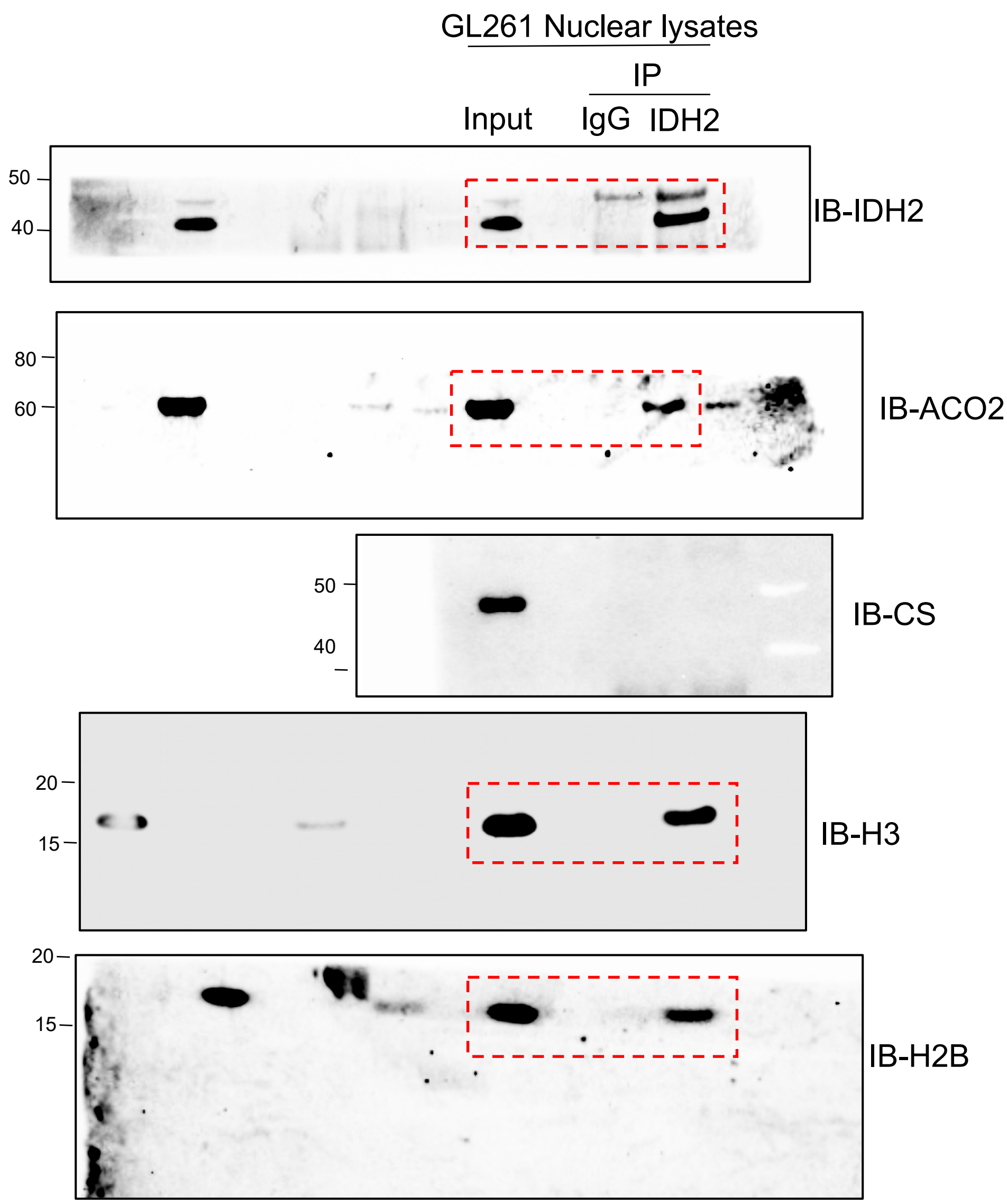

Supplementary Fig. S3i

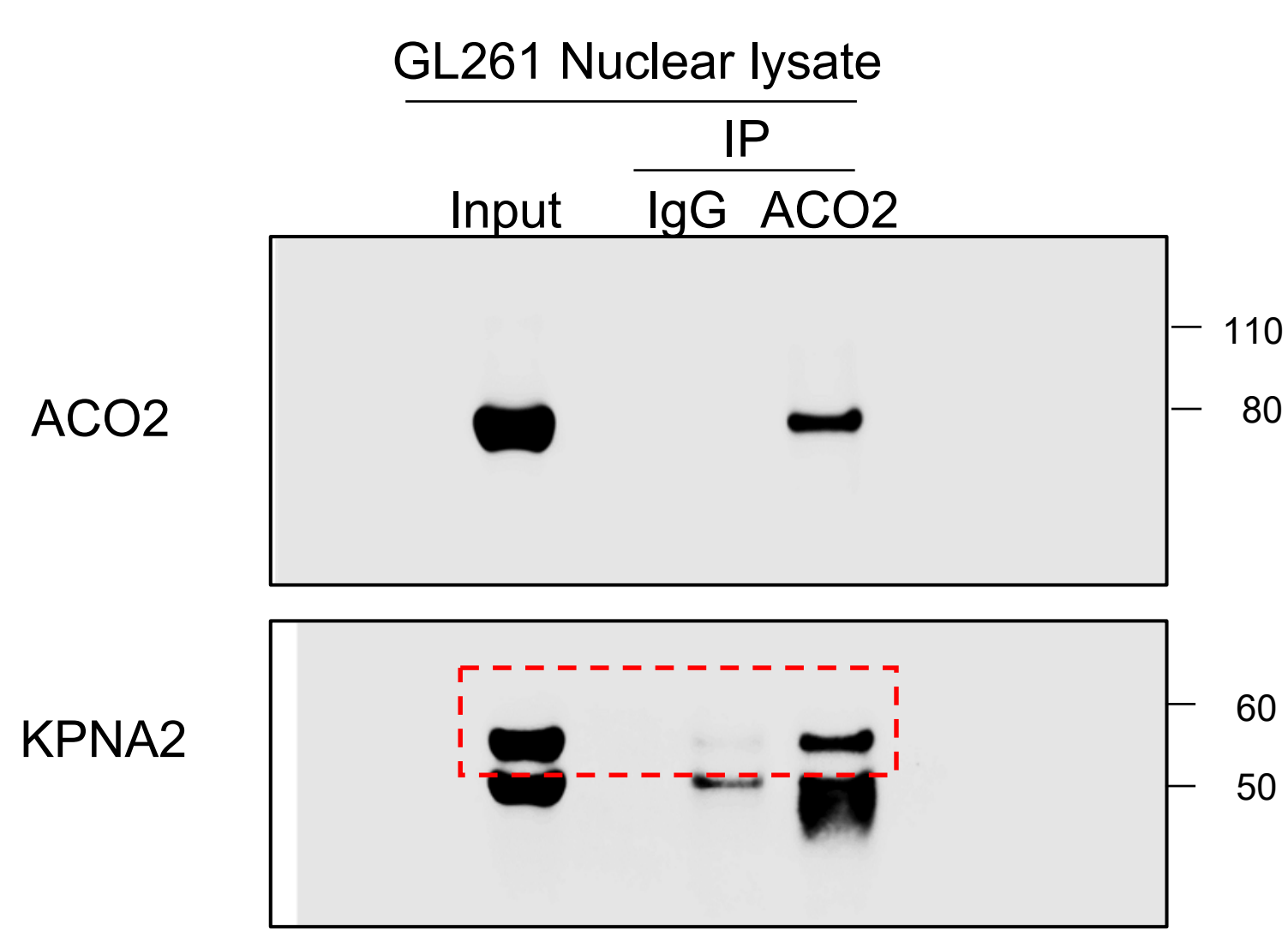

Supplementary Fig. S3j

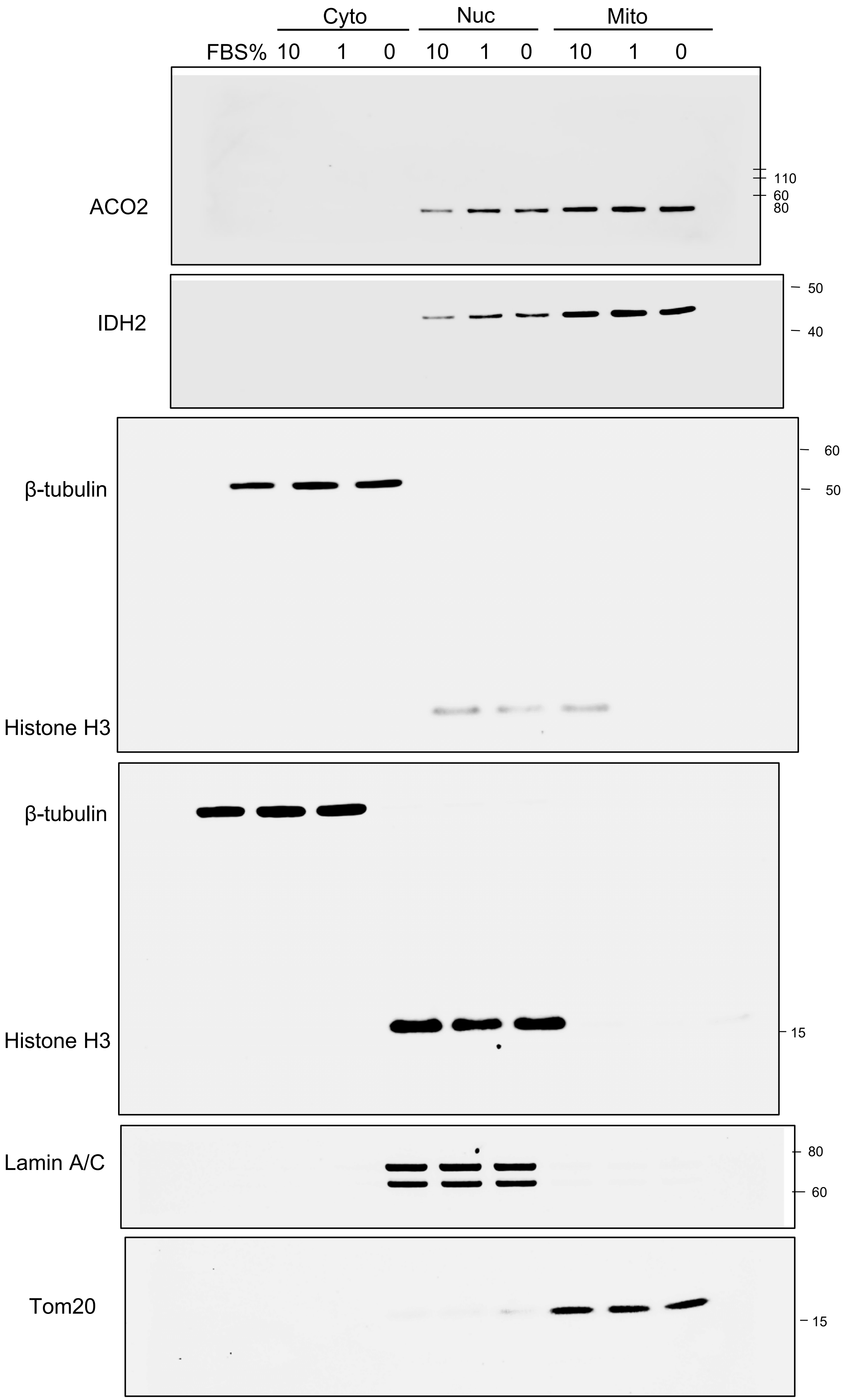

Supplementary Fig. S4a

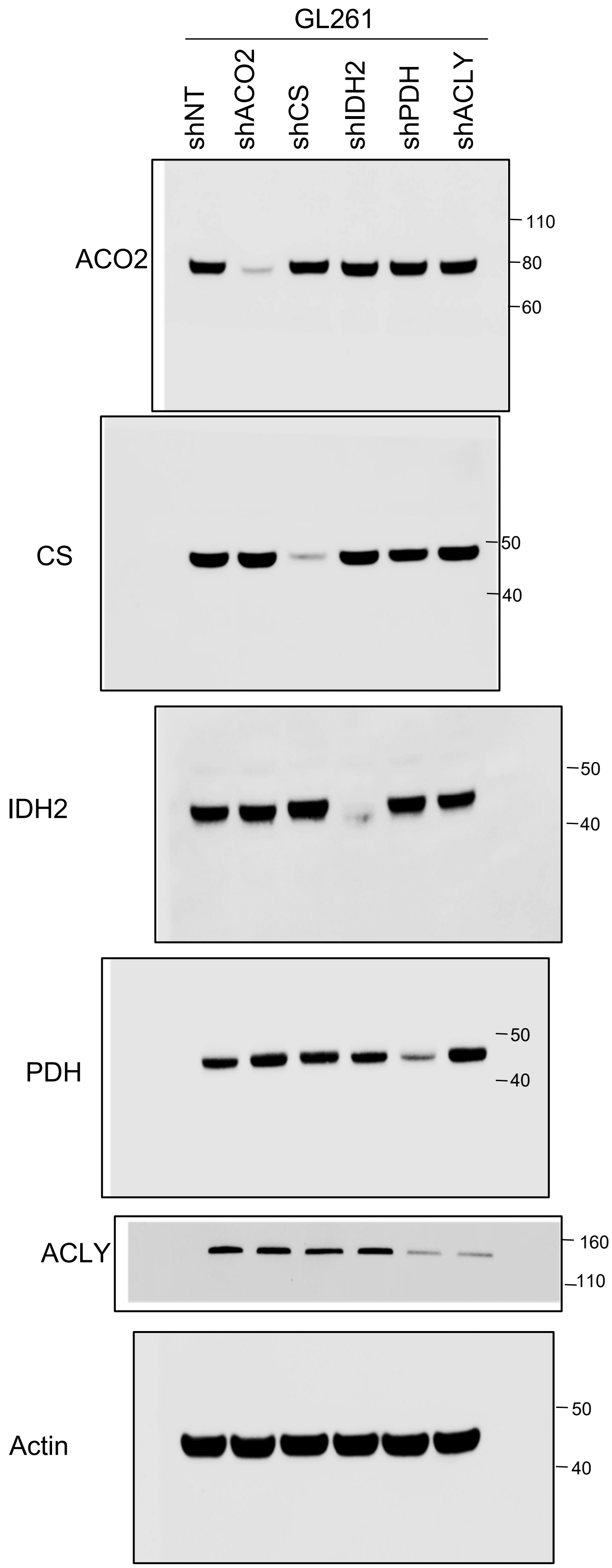

Supplementary Fig. S4b

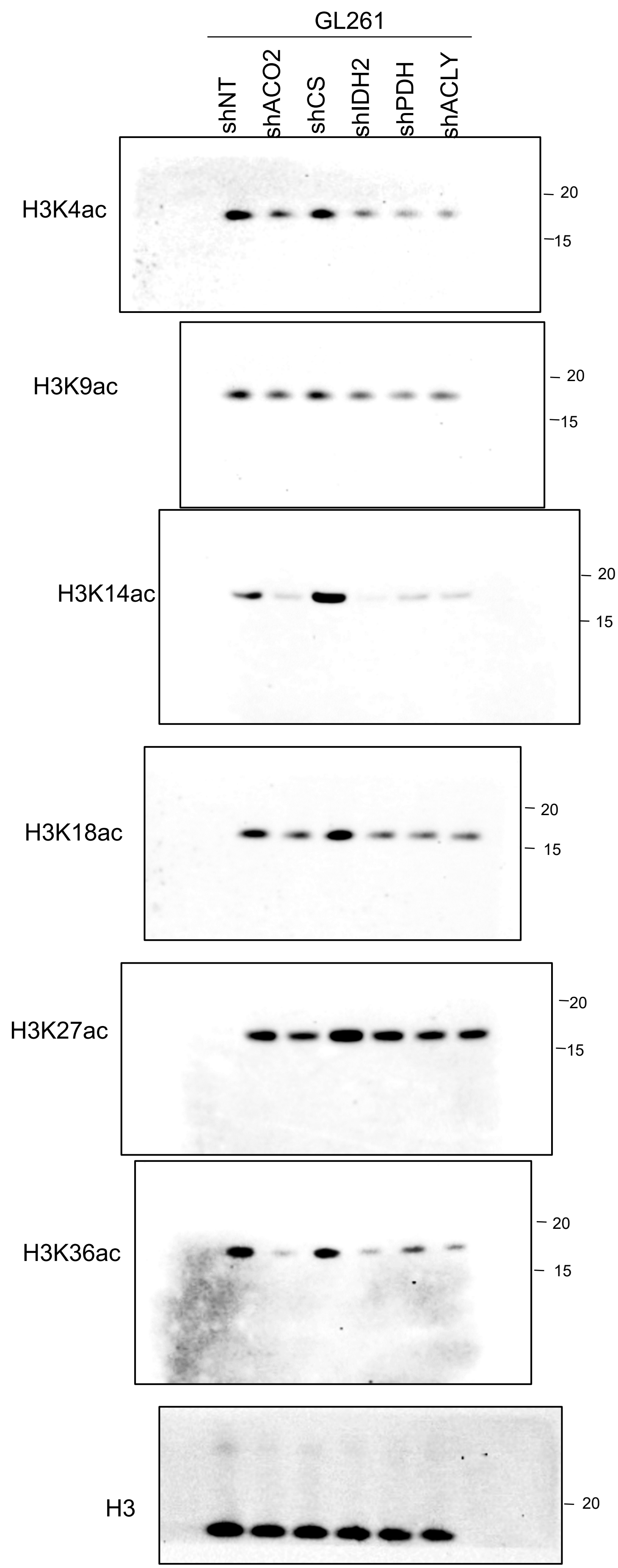

Supplementary Fig. S4f

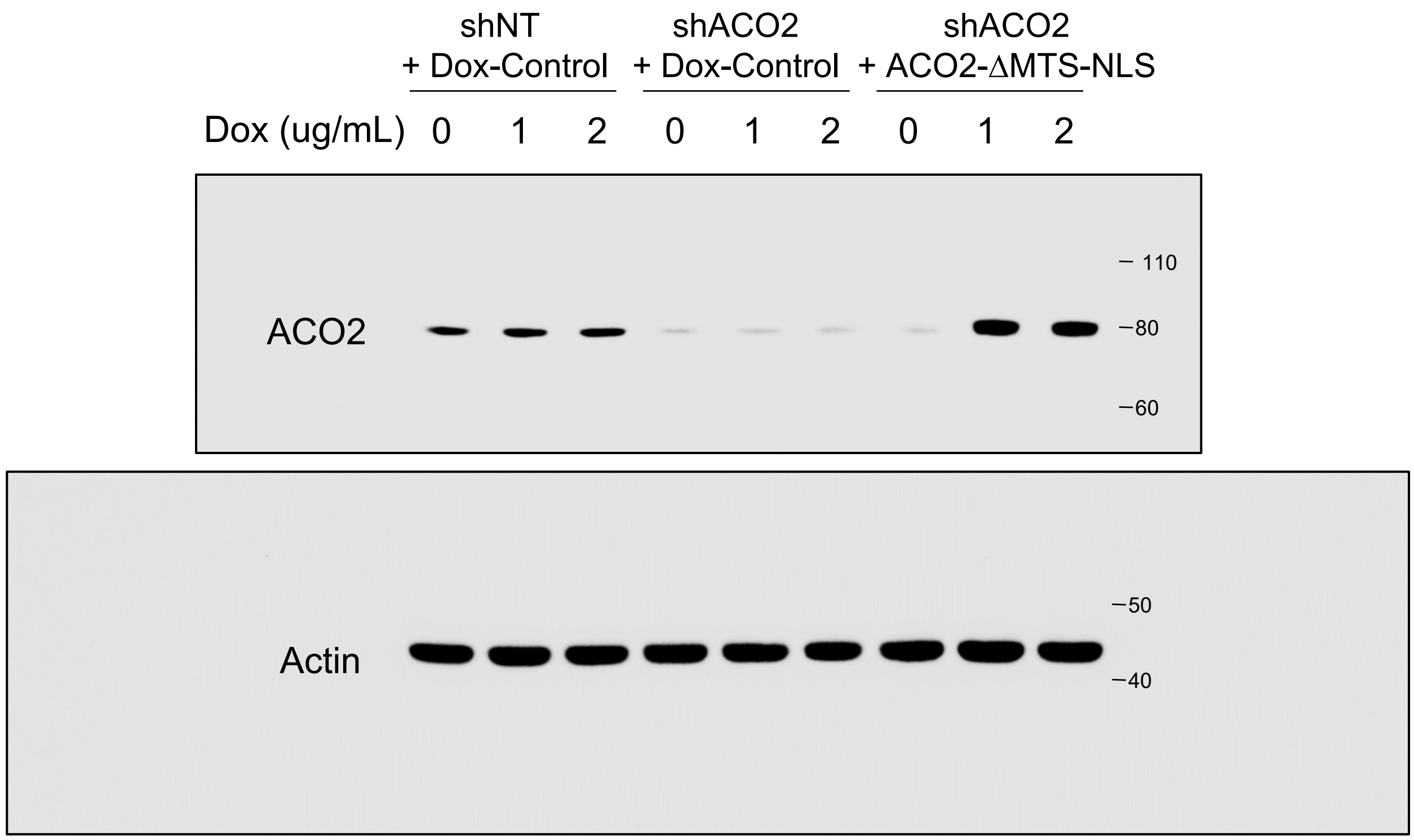

Supplementary Fig. S4h

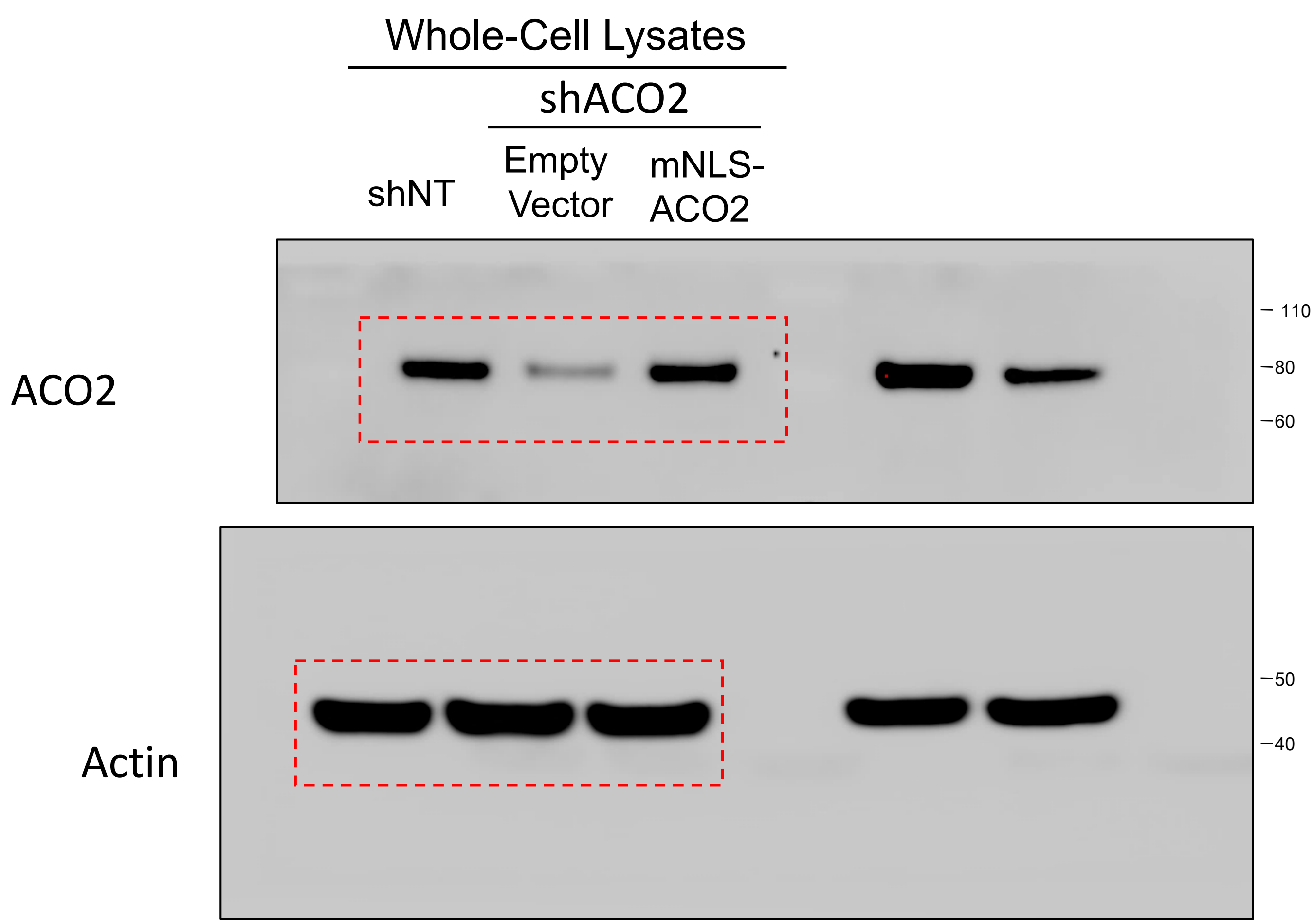

Supplementary Fig. S4i

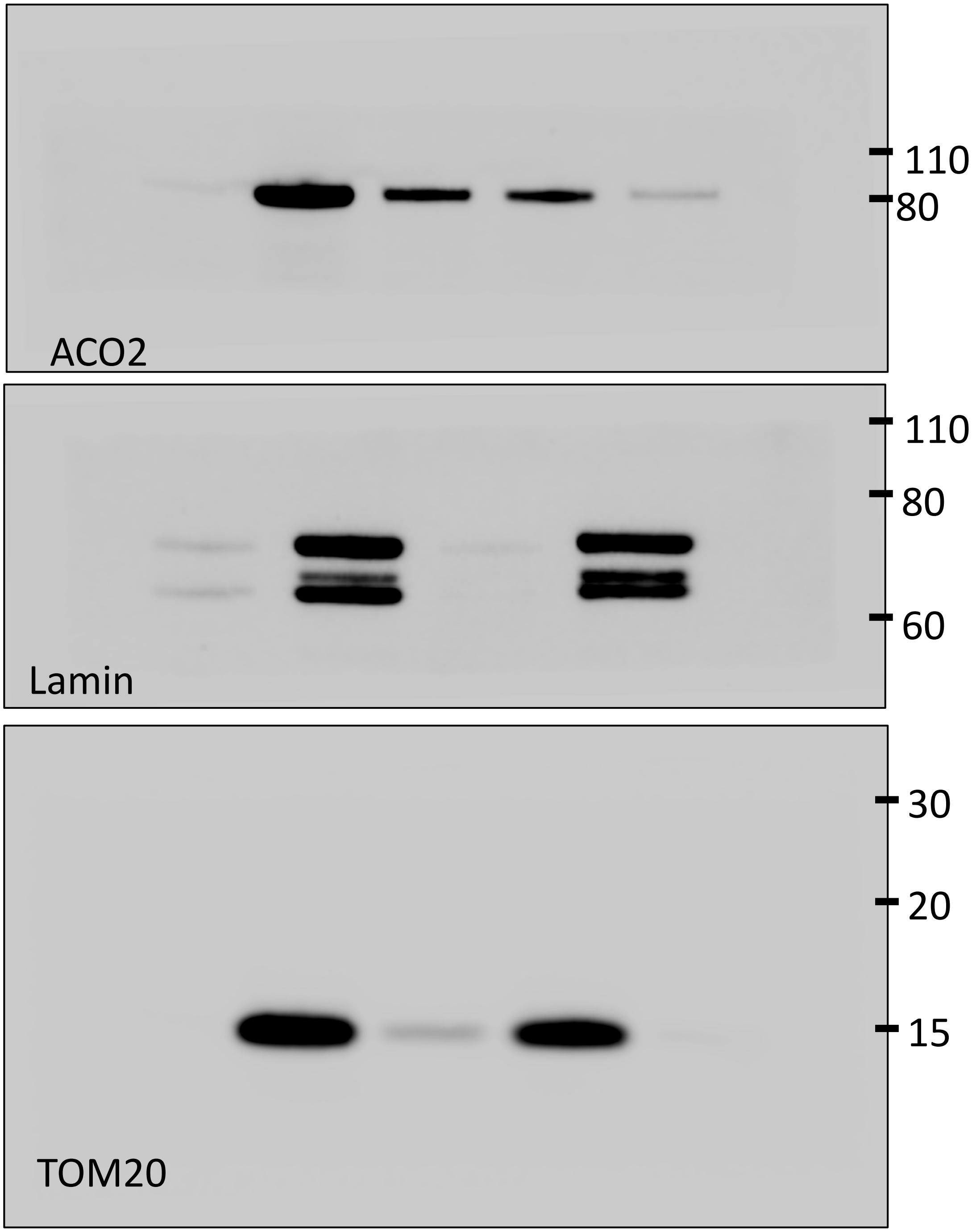

Supplementary Fig. S4j

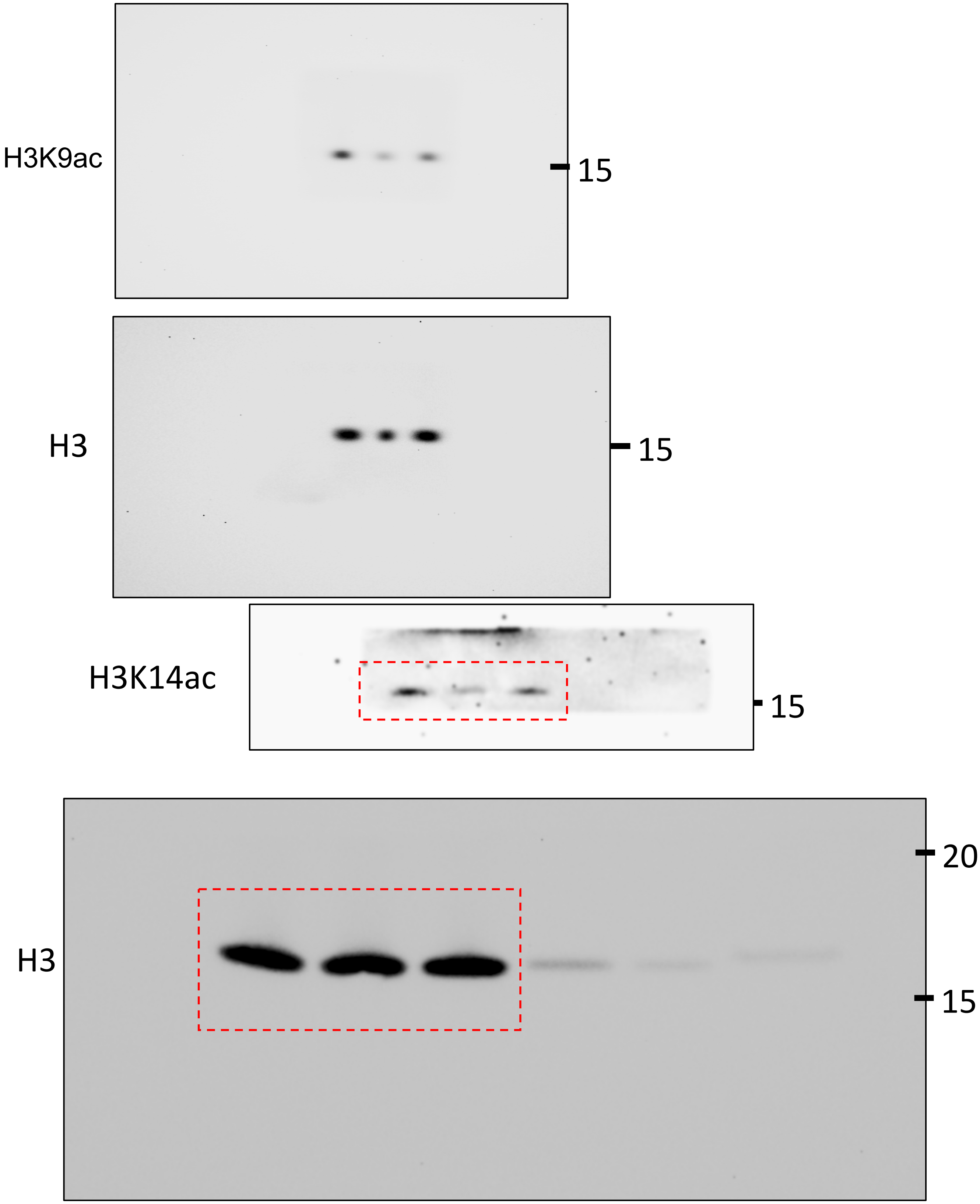

Supplementary Fig. S5a

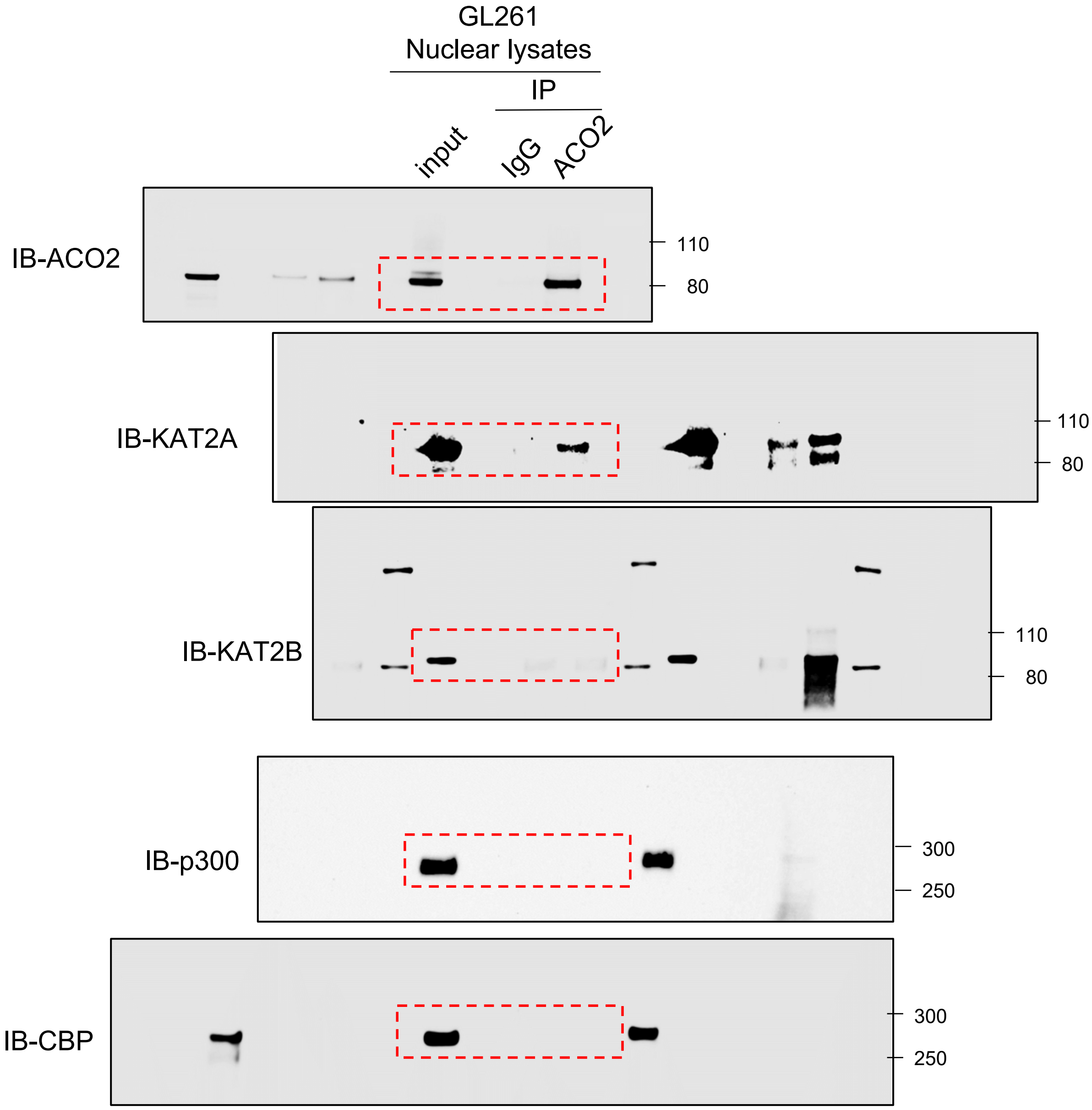

Supplementary Fig. S5b

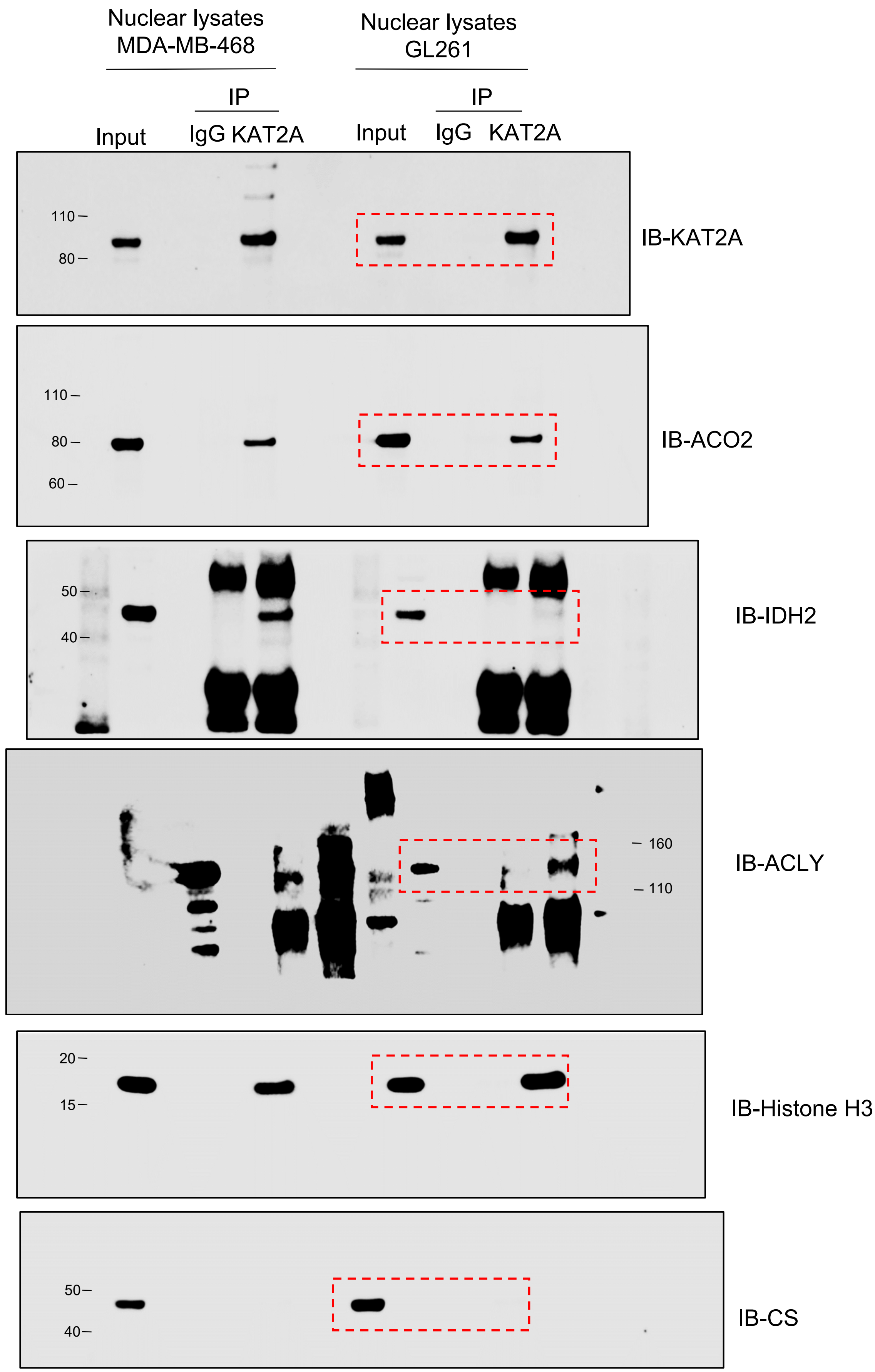

Supplementary Fig. S6a

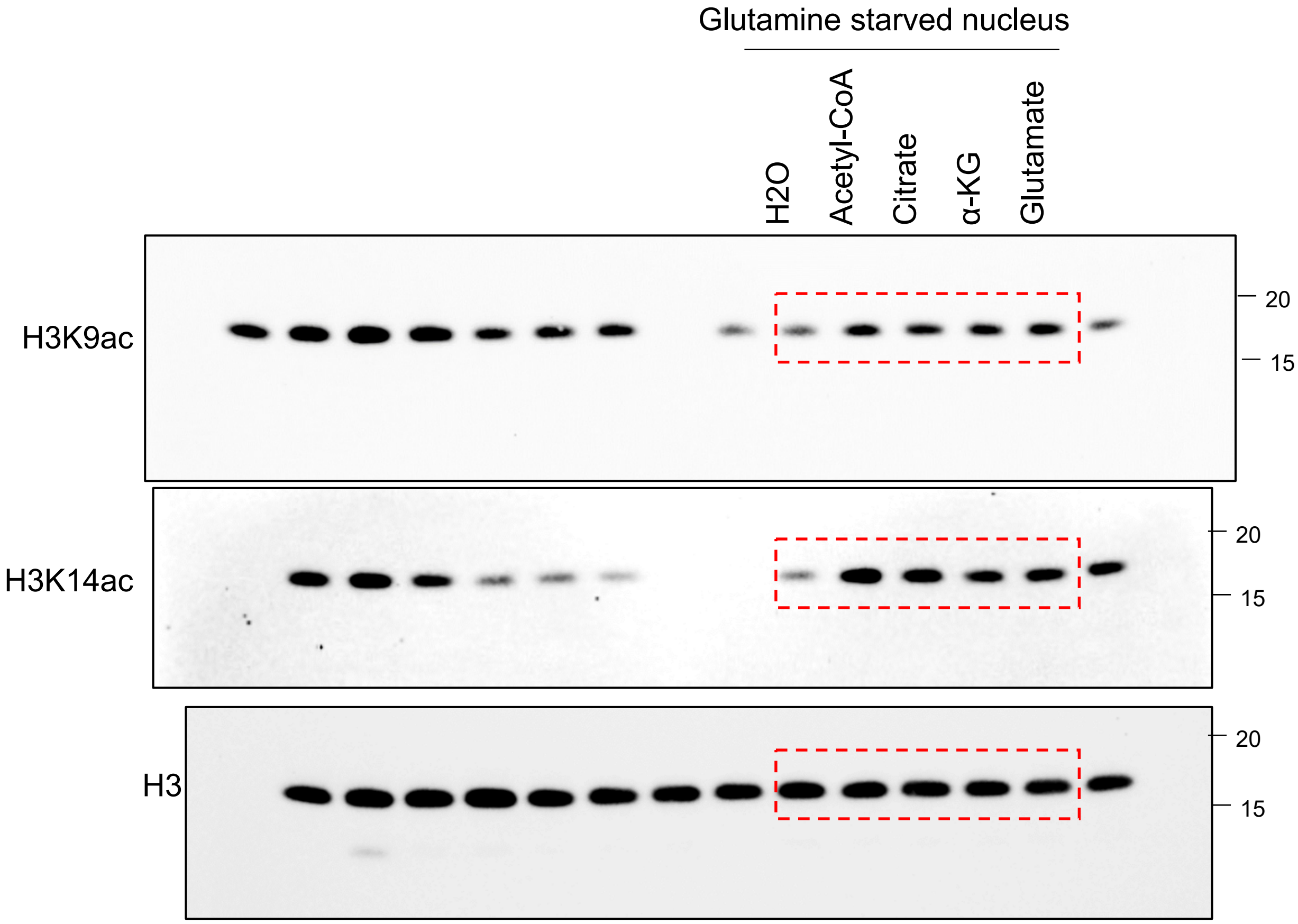

Supplementary Fig. S6c

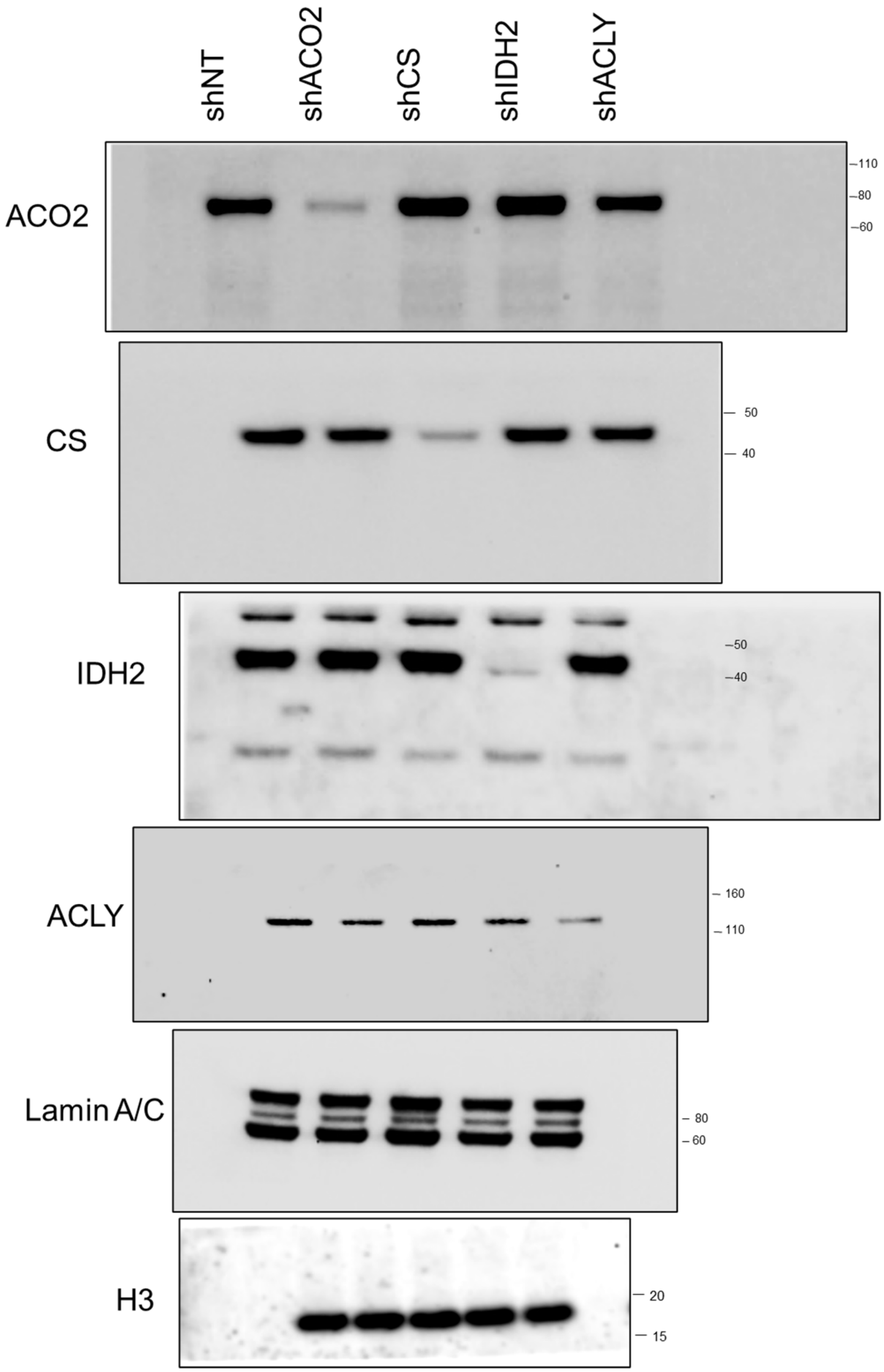

Supplementary Fig. S6j

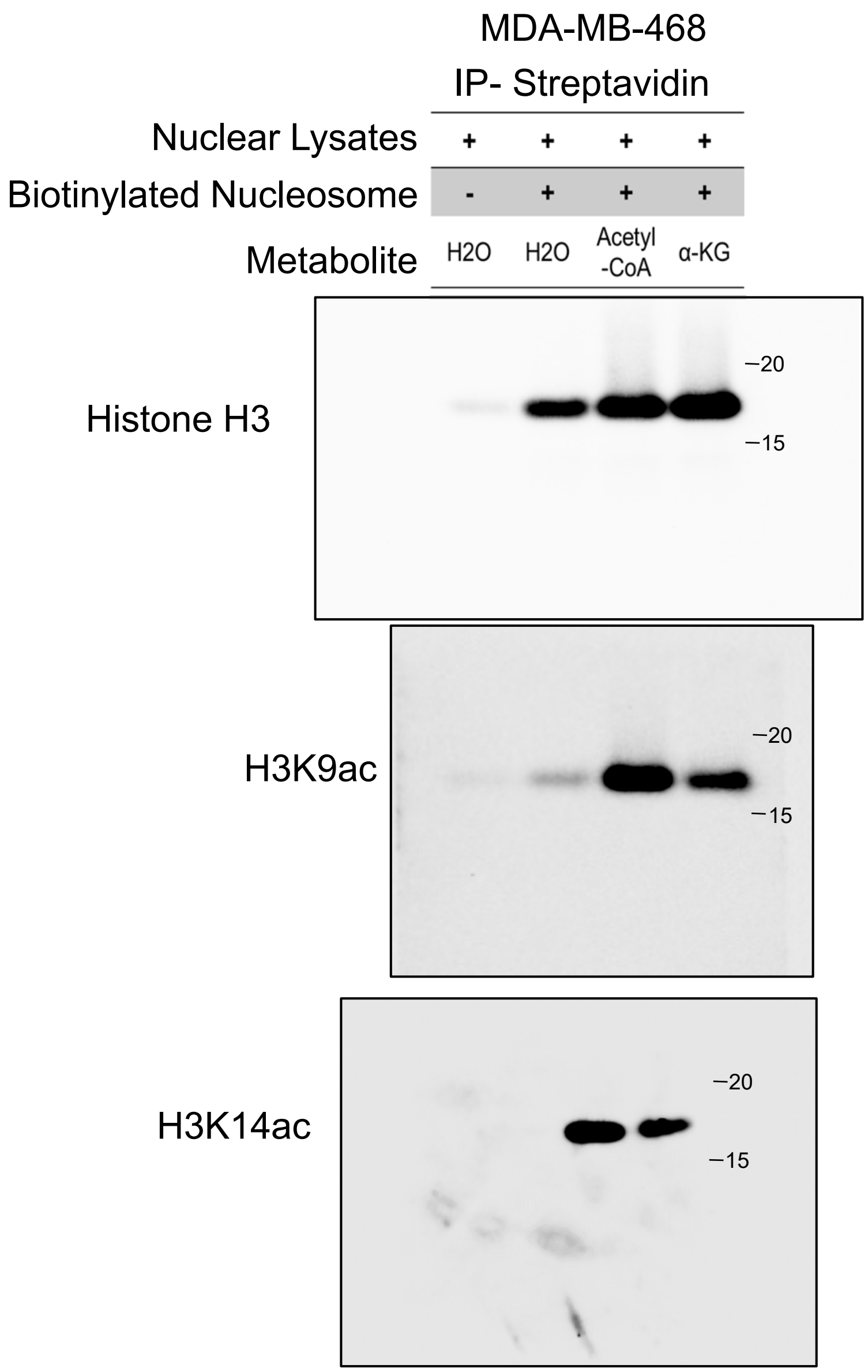

Supplementary Fig. S6k

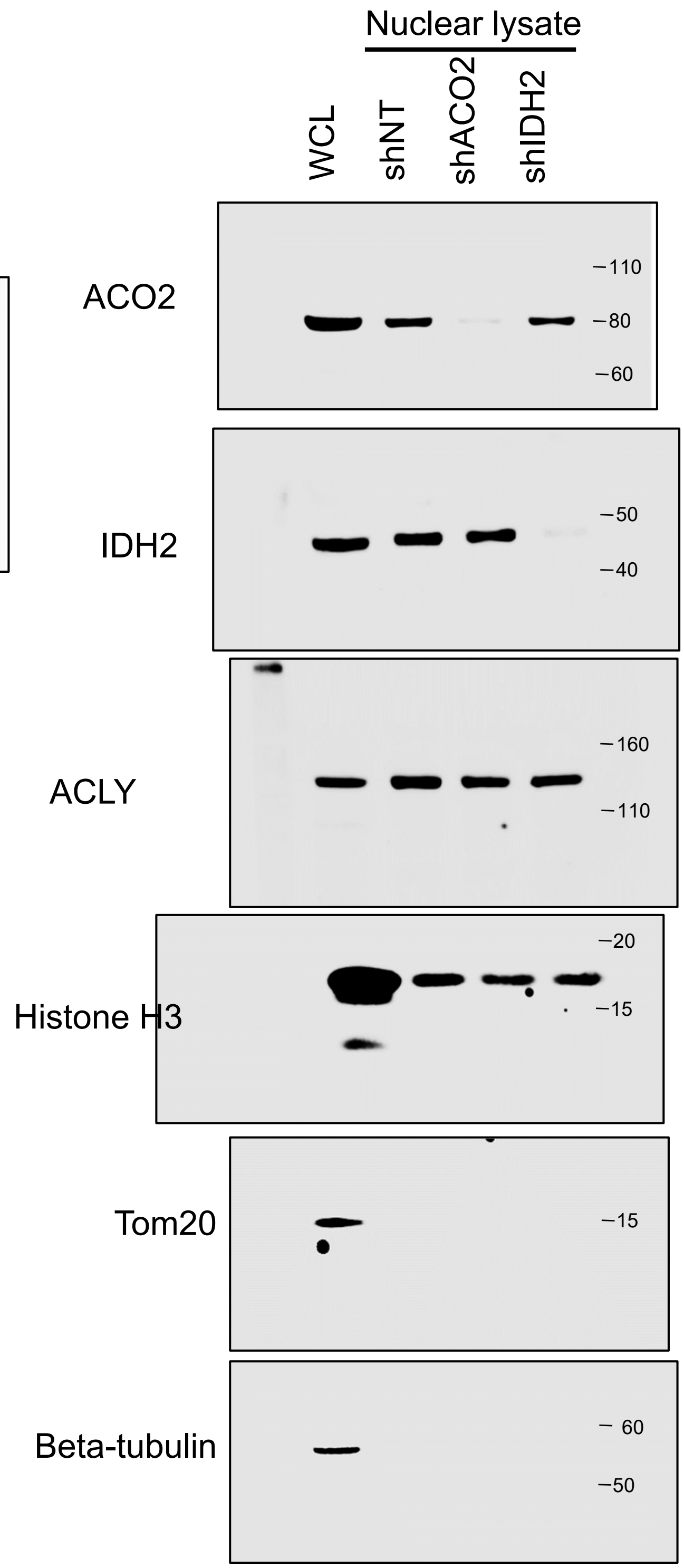

Supplementary Fig. S7d

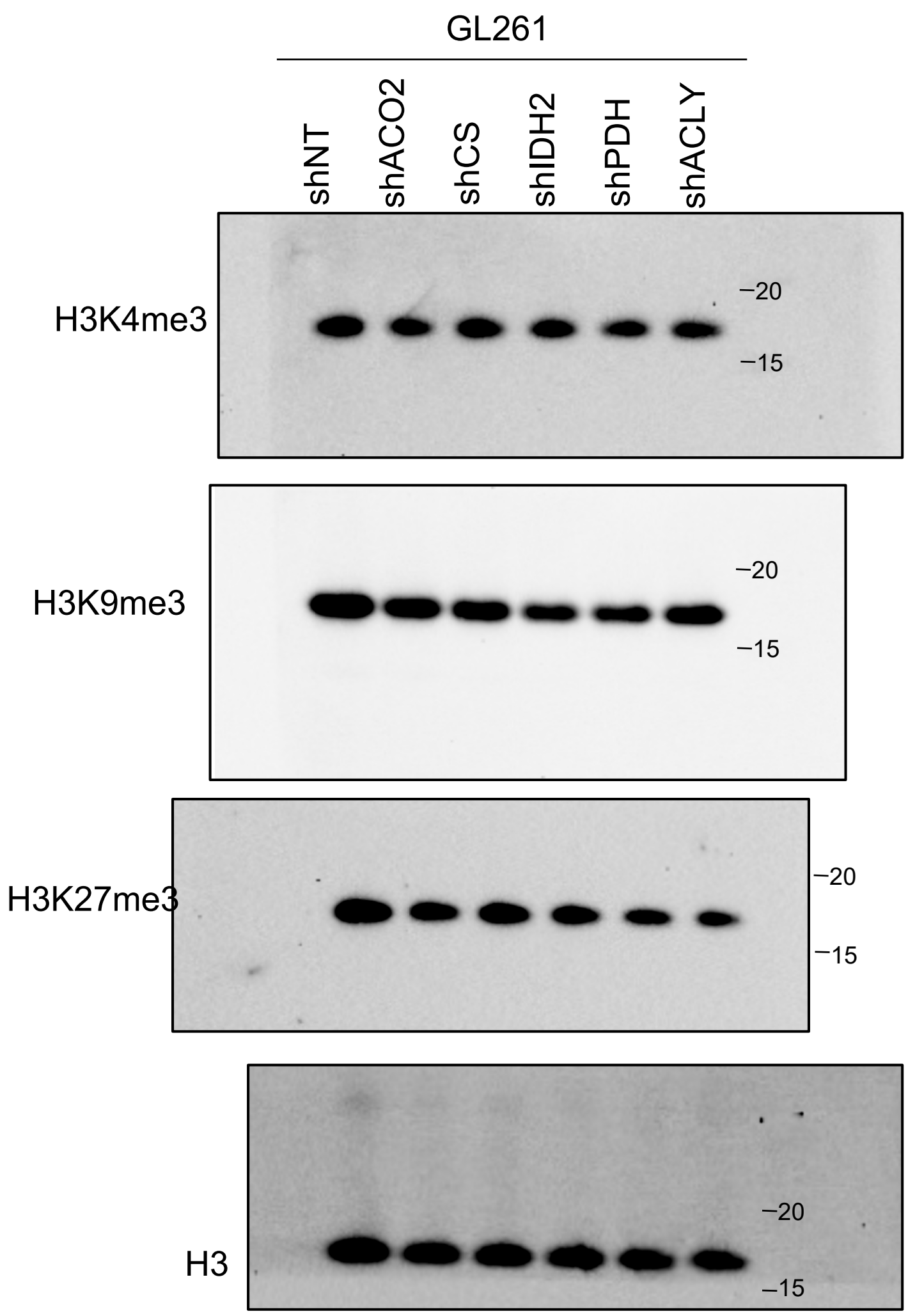

Supplementary Fig. S7f

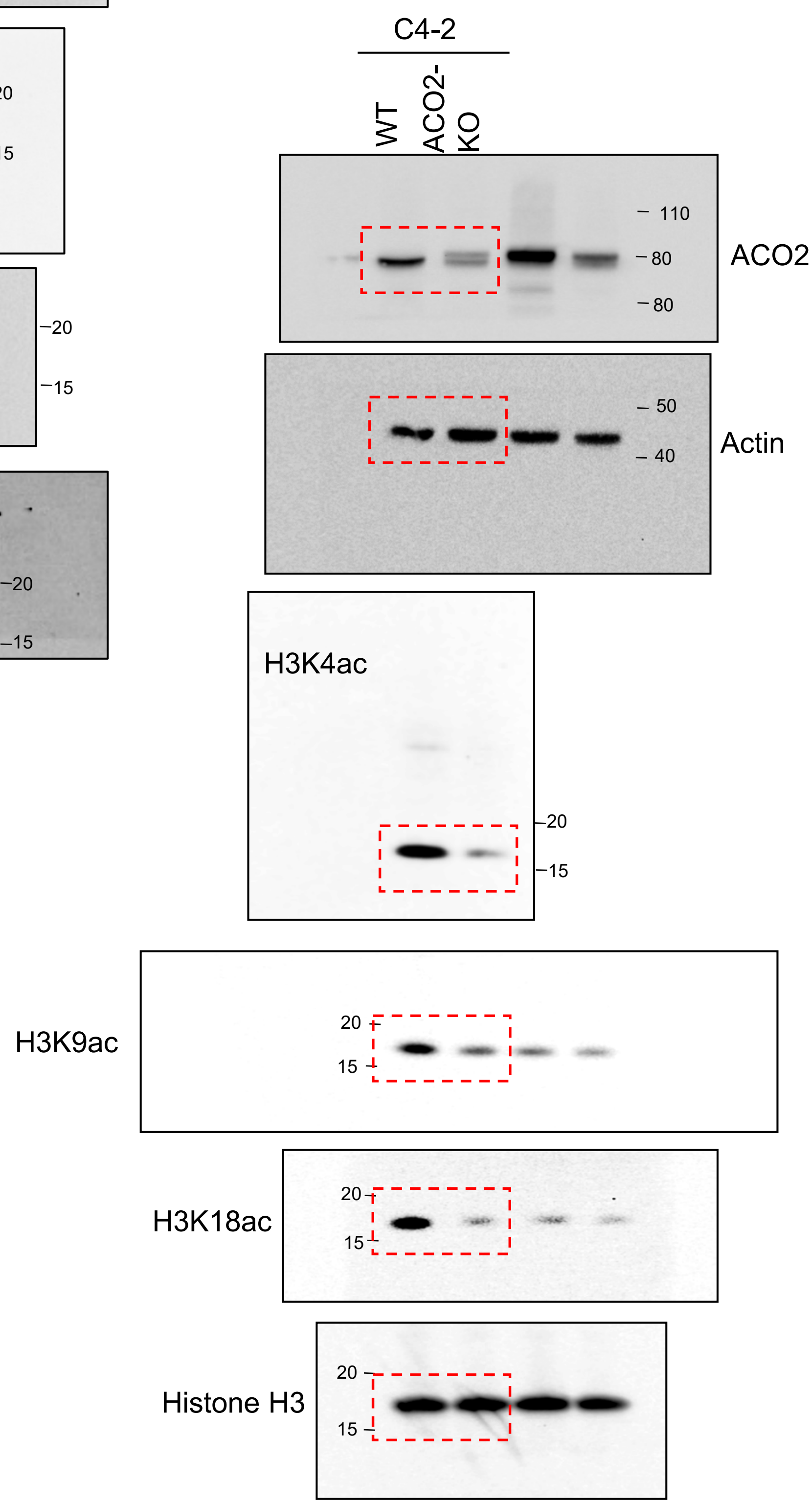

Supplementary Fig. S7f

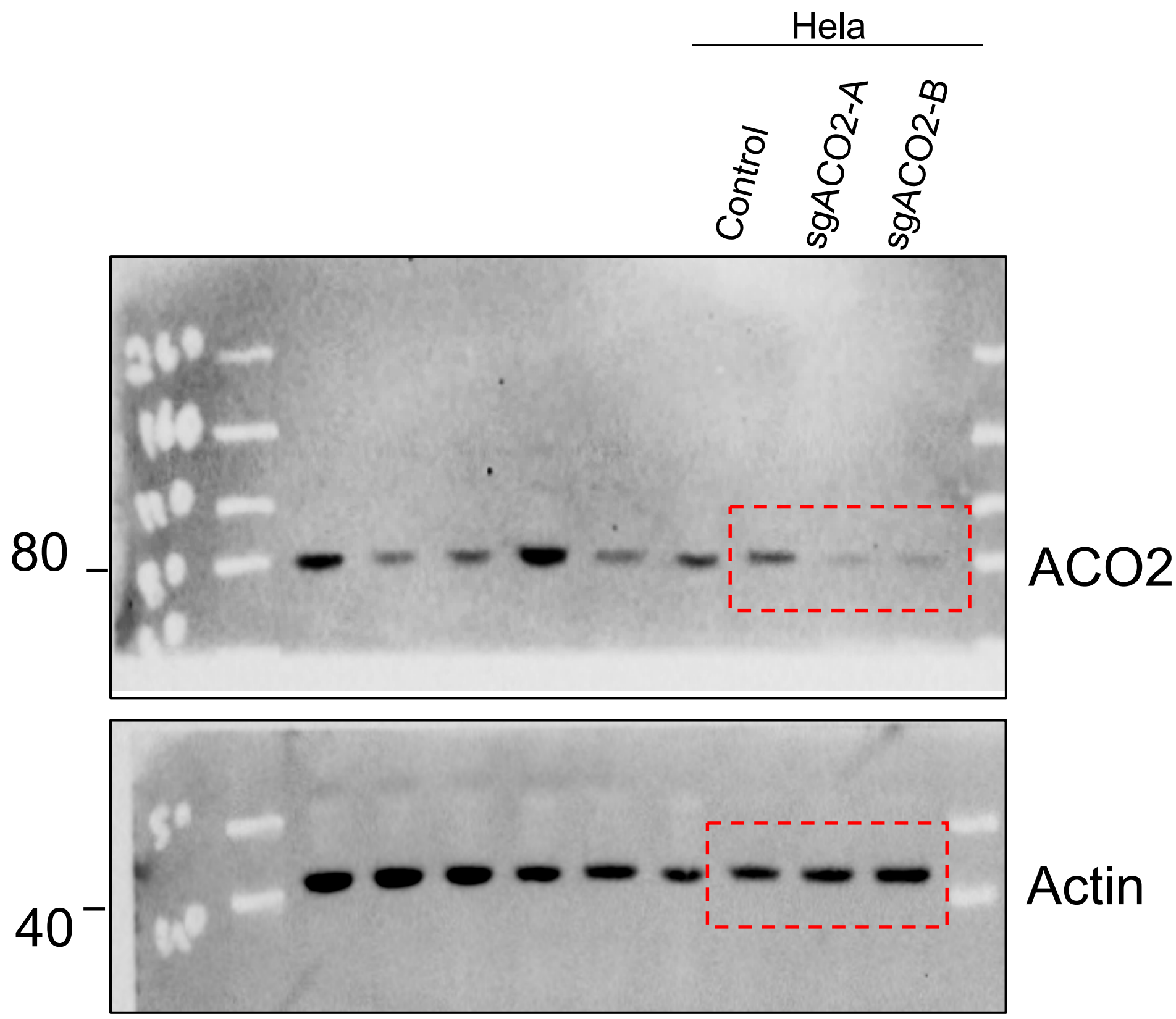

Supplementary Fig. S7f

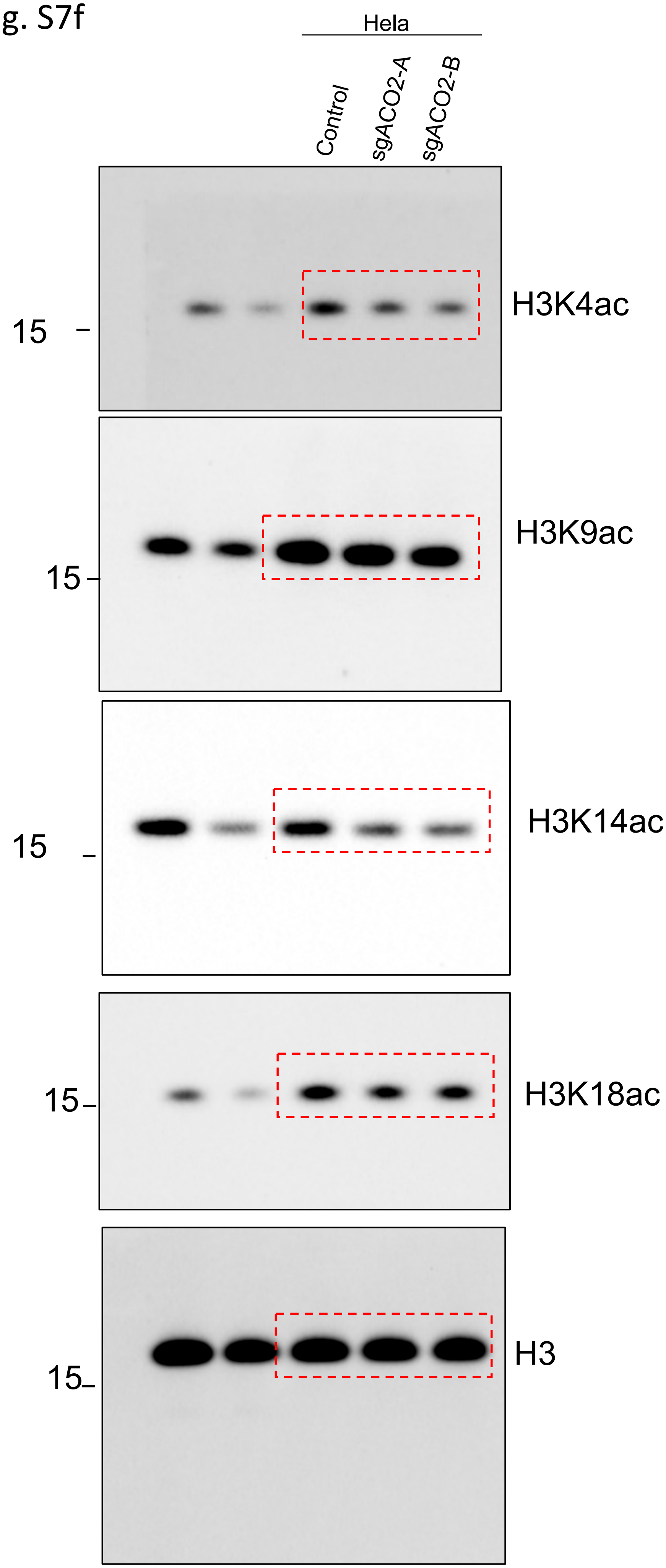

Supplementary Fig. S9d

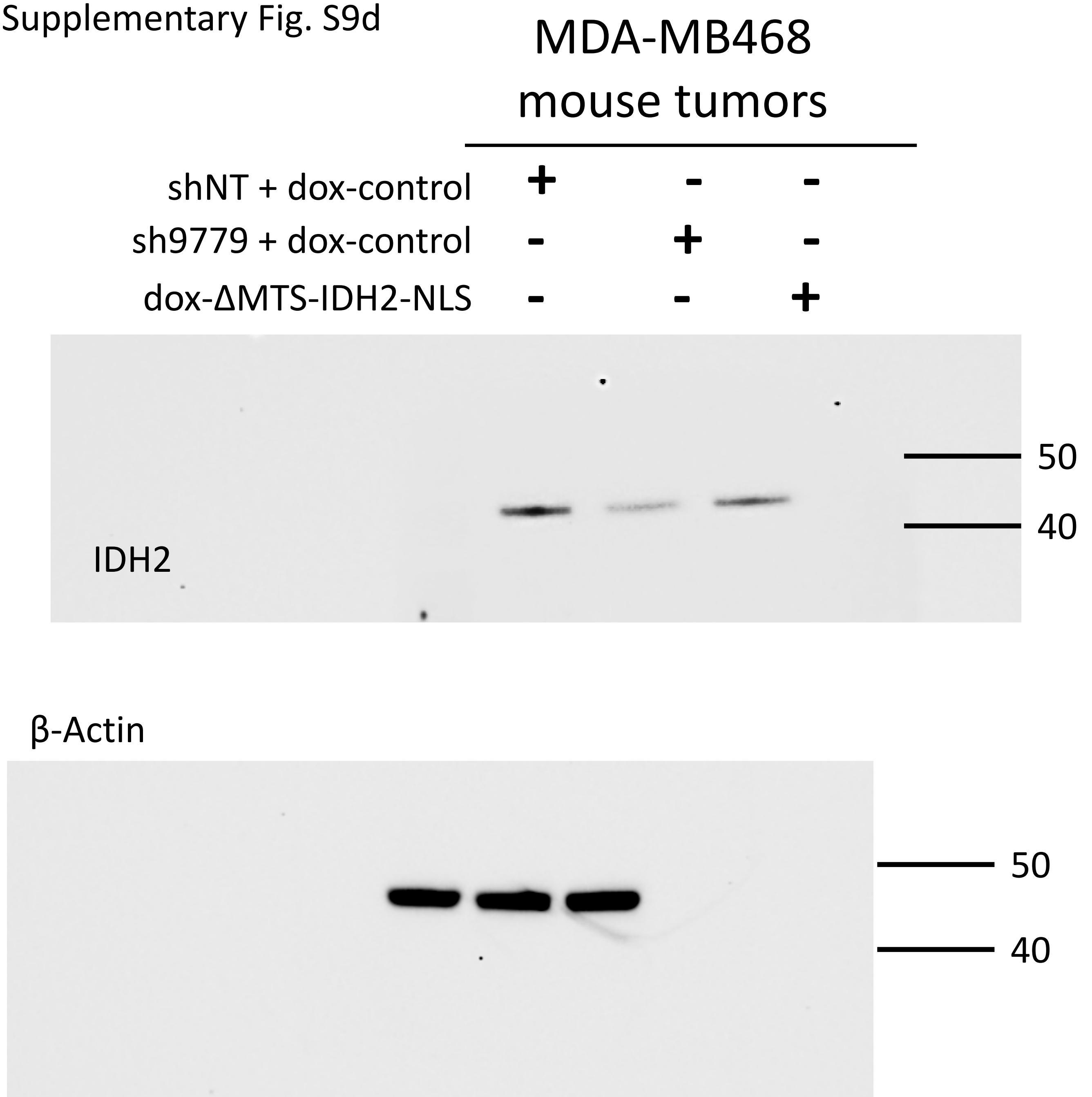

Supplementary Fig. S9e

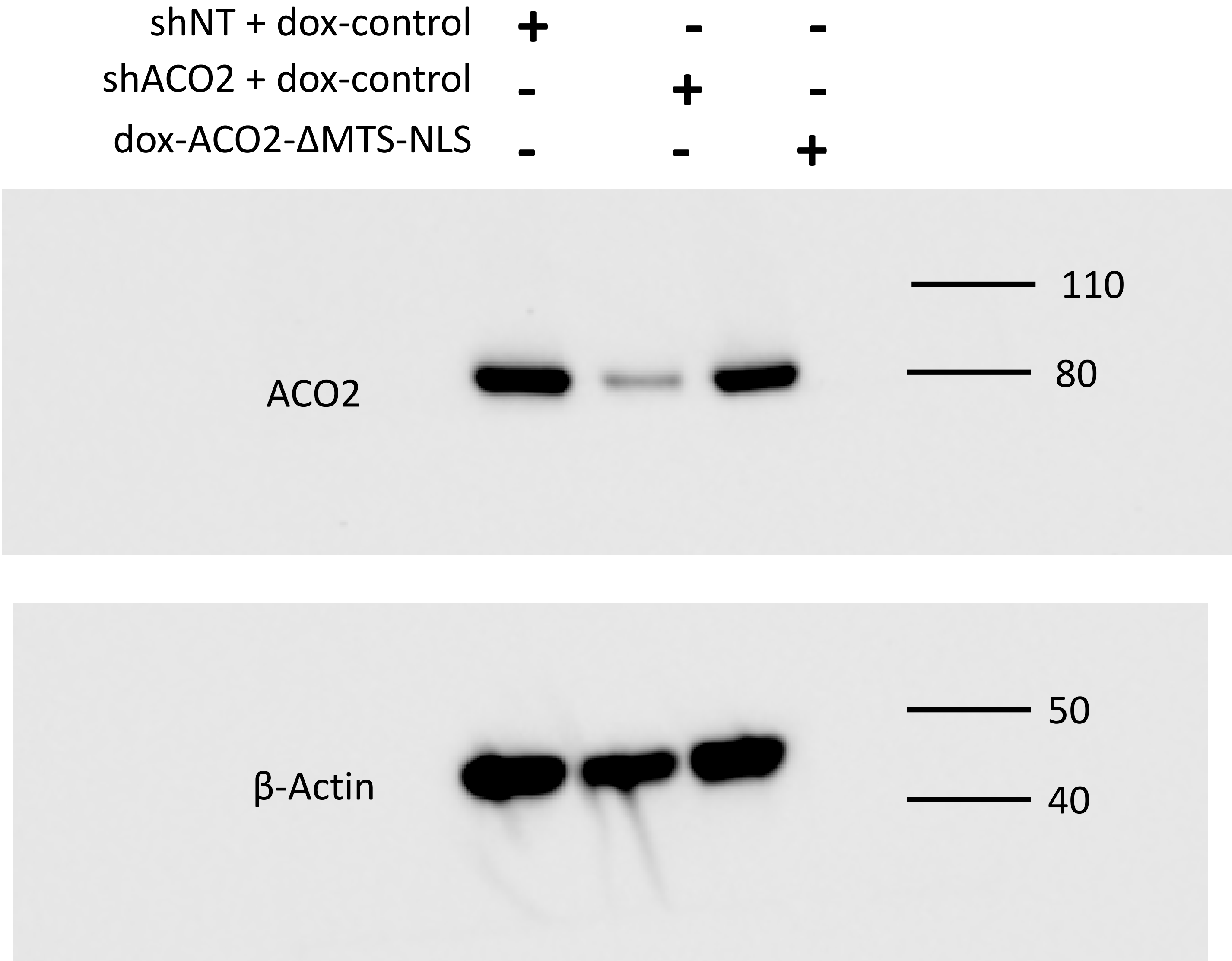

Supplement: Supplementary file 1 [file 41467_2026_74786_MOESM1_ESM.pdf]
