# Supplementary material for: Nuclear-specific reductive carboxylation of alpha-ketoglutarate fuels histone acetylation to induce chromatin accessibility and gene activation
Source: Nat Commun. 2026 Jul 1;17:8216. doi: 10.1038/s41467-026-74786-3 (PMC13462710; doi:10.1038/s41467-026-74786-3)
Supplement: Supplementary file 2 [file 41467_2026_74786_MOESM2_ESM.pdf]

Description of Additional Supplementary Files

Supplementary Data 1. List of Antibodies
