# Supplementary material for: Nuclear-specific reductive carboxylation of alpha-ketoglutarate fuels histone acetylation to induce chromatin accessibility and gene activation
Source: Nat Commun. 2026 Jul 1;17:8216. doi: 10.1038/s41467-026-74786-3 (PMC13462710; doi:10.1038/s41467-026-74786-3)
Supplement: Supplementary file 3 [file 41467_2026_74786_MOESM4_ESM.pdf]

nature portfolio

Corresponding author(s): Subhamoy Dasgupta, PhD

Last updated by author(s): Apr 3, 2026

Reporting Summary

Nature Portfolio wishes to improve the reproducibility of the work that we publish. This form provides structure for consistency and transparency in reporting. For further information on Nature Portfolio policies, see our Editorial Policies and the Editorial Policy Checklist.

Statistics

For all statistical analyses, confirm that the following items are present in the figure legend, table legend, main text, or Methods section.

n/a	Confirmed	
☐	☒	The exact sample size (*n*) for each experimental group/condition, given as a discrete number and unit of measurement
☐	☒	A statement on whether measurements were taken from distinct samples or whether the same sample was measured repeatedly
☐	☒	The statistical test(s) used AND whether they are one- or two-sided<br>*Only common tests should be described solely by name; describe more complex techniques in the Methods section.*
☐	☒	A description of all covariates tested
☐	☒	A description of any assumptions or corrections, such as tests of normality and adjustment for multiple comparisons
☐	☒	A full description of the statistical parameters including central tendency (e.g. means) or other basic estimates (e.g. regression coefficient) AND variation (e.g. standard deviation) or associated estimates of uncertainty (e.g. confidence intervals)
☐	☒	For null hypothesis testing, the test statistic (e.g. *F*, *t*, *r*) with confidence intervals, effect sizes, degrees of freedom and *P* value noted<br>*Give P values as exact values whenever suitable.*
☒	☐	For Bayesian analysis, information on the choice of priors and Markov chain Monte Carlo settings
☒	☐	For hierarchical and complex designs, identification of the appropriate level for tests and full reporting of outcomes
☐	☒	Estimates of effect sizes (e.g. Cohen's *d*, Pearson's *r*), indicating how they were calculated

Our web collection on statistics for biologists contains articles on many of the points above.

Software and code

Policy information about availability of computer code

Data collection	Leica TCS SP8 Confocal Microscope<br>ImagestreamX Mark II imaging flow cytometer<br>ChemiDoc Imaging system (Bio-Rad)<br>Thermo Scientific nanoLC-1000 system coupled to an Orbitrap Fusion mass spectrometer with an ESI source<br>Illumina NovaSeq6000<br>QuantStudio 6 Flex Real-Time PCR
Data analysis	GraphPad Prism software version 11.0.0<br>IDEAS™ (v6.2)<br>Leica LAS X and Huygens deconvolution v3.7.4.23463<br>ImageJ v1.54p<br>Image Lab (v6.0.1, Bio-Rad)<br>FSC Express v7.28.0035<br>Proteome Discoverer 2.1 interface (PD 2.1, Thermo Fisher) with the Mascot algorithm (Mascot 2.4, Matrix Science)

For manuscripts utilizing custom algorithms or software that are central to the research but not yet described in published literature, software must be made available to editors and reviewers. We strongly encourage code deposition in a community repository (e.g. GitHub). See the Nature Portfolio guidelines for submitting code & software for further information.

Data

Policy information about availability of data

All manuscripts must include a data availability statement. This statement should provide the following information, where applicable:

- Accession codes, unique identifiers, or web links for publicly available datasets
- A description of any restrictions on data availability
- For clinical datasets or third party data, please ensure that the statement adheres to our policy

The ATAC-seq data (C4-2 cells) generated in this study have been deposited in the Gene Expression Omnibus (GEO) database under accession code GSE301090 [https://www.ncbi.nlm.nih.gov/geo/query/acc.cgi?acc=GSE301090]. The RNA-seq data generated in this study have been deposited in the Gene Expression Omnibus (GEO) database under accession code GSE301089 [https://www.ncbi.nlm.nih.gov/geo/query/acc.cgi?acc=GSE301089]. The ATAC-seq data (MDA-MB-231 cells) generated in this study have been deposited in the Gene Expression Omnibus (GEO) database under accession code GSE301095 [https://www.ncbi.nlm.nih.gov/geo/query/acc.cgi?acc=GSE301095]. The Mass Spectrometry raw data (IP ACO2 and IDH2) generated in this study have been deposited in the massIVE repository under accession code MSV000099250[https://massive.ucsd.edu/ProteoSAFe/private-dataset.jsp?task=44dd358087cf4cd48f269ff4b81785a8]. The Mass Spectrometry raw data and the Xcel file with the quantitative analysis of 13C labeling generated in this study have been deposited to the massIVE repository under accession code MSV000099224[https://massive.ucsd.edu/ProteoSAFe/private-dataset.jsp?task=bc2259d87bb543cd8dc524ce89bb835f]. The HeLa-S3 ChIP–seq datasets used in this study are available in the GEO database under accession codes IgG: GSM935339[https://www.ncbi.nlm.nih.gov/geo/query/acc.cgi?acc=GSM935339], and KAT2A: GSM935302 [https://www.ncbi.nlm.nih.gov/geo/query/acc.cgi?acc=GSM935302]

Research involving human participants, their data, or biological material

Policy information about studies with human participants or human data. See also policy information about sex, gender (identity/presentation), and sexual orientation and race, ethnicity and racism.

Reporting on sex and gender	C4-2 prostate cancer cell line was implanted in male NSG mice for xenograft study.<br>MDA-MB-468 triple negative breast cancer cell line was implanted in mammary fat pad of female NSG mice.
Reporting on race, ethnicity, or other socially relevant groupings	n/a
Population characteristics	n/a
Recruitment	n/a
Ethics oversight	n/a

Note that full information on the approval of the study protocol must also be provided in the manuscript.

Field-specific reporting

Please select the one below that is the best fit for your research. If you are not sure, read the appropriate sections before making your selection.

[x] Life sciences [ ] Behavioural & social sciences [ ] Ecological, evolutionary & environmental sciences

For a reference copy of the document with all sections, see nature.com/documents/nr-reporting-summary-flat.pdf

Life sciences study design

All studies must disclose on these points even when the disclosure is negative.

Sample size	Statistical methods were not used to predetermine sample size (n). Number of sample was determined based on experimental approach, availability, feasibility required to obtain definitive result
Data exclusions	None
Replication	Experiments reported are replicated at least three times (biological replicates) with similar observations.
Randomization	Majority of the experiments were performed following genetic manipulation of cells using shRNA/siRNA procedures or transfection of DNA plasmids. Similarly, animal experiments were performed with genetically manipulated cell lines. This design does not need randomization and recording the origin of samples are critical.
Blinding	The researchers were not blinded during data collection/ or analysis.

Reporting for specific materials, systems and methods

We require information from authors about some types of materials, experimental systems and methods used in many studies. Here, indicate whether each material, system or method listed is relevant to your study. If you are not sure if a list item applies to your research, read the appropriate section before selecting a response.

Materials & experimental systems

n/a	Involved in the study	
☐	☒	Antibodies
☐	☒	Eukaryotic cell lines
☐	☐	Palaeontology and archaeology
☐	☒	Animals and other organisms
☐	☐	Clinical data
☐	☐	Dual use research of concern
☐	☐	Plants

Methods

n/a	Involved in the study	
☒	☐	ChIP-seq
☐	☒	Flow cytometry
☒	☐	MRI-based neuroimaging

Antibodies

Antibodies used

Cell Signaling, Acetyl-Histone H3 (Lys14) (D4B9) Rabbit mAb, 7627S - 1:2000
Cell Signaling, Acetyl-Histone H3 (Lys9) (C5B11) Rabbit mAb, 9649S - 1:2000
Cell Signaling, Acetyl-Histone H3 (Lys36) (D9T5Q) Rabbit mAb, 27683S - 1:2000
Cell Signaling, Acetyl-Histone H3 (Lys27) (D5E4) XP® Rabbit mAb, 8173S - 1:500
Cell Signaling, Histone H4 (L64C1) Mouse mAb, 2935S - 1:2000
Cell Signaling, Histone H3 (D1H2) XP® Rabbit mAb, 4499S- 1:1000
Cell Signaling, Histone H2B (D2H6) Rabbit mAb, 12364S - 1:500
Cell Signaling, Histone H2A (L88A6) Mouse mAb, 3636S - 1:1000
Cell Signaling, Tri-Methyl-Histone H3 (Lys9) (D4W1U) Rabbit mAb, 13969S - 1:1500
Cell Signaling, Tri-Methyl-Histone H3 (Lys27) (C36B11) Rabbit mAb, 9733S - 1:1000
Cell Signaling, Tri-Methyl-Histone H3 (Lys4) (C42D8) Rabbit mAb , 9751S - 1:8000
Cell Signaling, ACO2 (D6D9) XP® Rabbit mAb , 6571S - 1:1000
Cell Signaling, SDHA (D6J9M) XP® Rabbit mAb, 11998S - 1:1000
Cell Signaling, Citrate Synthase (D7V8B) Rabbit mAb , 14309S - 1:1000
Cell Signaling, MDH2 (D8Q5S) Rabbit mAb, 11908S - 1:1000
Cell Signaling, Fumarase (D9C5) Rabbit mAb , 4567S - 1:1000
Cell Signaling, Succinyl-CoA Synthetase Antibody, 5557S - 1:1000
Cell Signaling, Lamin A/C Antibody, 2032S - 1:1000
Cell Signaling, Lamin A/C (4C11) Mouse mAb, 4777S - 1:2000
Cell Signaling, β-Tubulin (9F3) Rabbit mAb, 2128S - 1:1000
Cell Signaling, Tom20 (D8T4N) Rabbit mAb, 42406S - 1:5000
Cell Signaling, ATP-Citrate Lyase (D1X6P) Rabbit mAb, 13390S - 1:1000
Cell Signaling, KPNA2 Antibody, 14372S - 1:1000
Cell Signaling, Goat Anti-Mouse IgG, Light-Chain Specific Antibody (HRP Conjugate), 91196S - 1:1000
Cell Signaling, Mouse Anti-rabbit IgG (Conformation Specific) (L27A9) mAb (HRP Conjugate), 5127S - 1:2000
Promega, Anti-Rabbit IgG (H + L), HRP conjugate, W4011 - 1:2000
Promega, Anti-Mouse IgG (H + L), HRP conjugate, W4021 - 1:2000
Invitrogen, Aconitase 2 Monoclonal Antibody (7G4) (Mouse), MA1-029 - 1:1000, 6uL for IP
Invitrogen, IDH2 Monoclonal Antibody (GT673) (Mouse), MA5-17271 - 1:1000, 6uL for IP
Invitrogen, KPNA3 Polyclonal Antibody, PA5-117127 - 1:500
Invitrogen, Aconitase 2 Polyclonal Antibody (Goat), PA5-19269 - 3-5 μg/mL
Millipore Sigma, Normal Mouse IgG, 12-371 - 0.5ug for IP
Millipore Sigma, Normal Rabbit IgG, 12-370 - 0.5ug for IP
Millipore Sigma, Anti-acetyl-Histone H3 (Lys23) Antibody, 07-355- 1:90,000
Millipore Sigma, Anti-acetyl-Histone H3 (Lys36) Antibody, 07-540 - 1:1000
Millipore Sigma, Anti-acetyl-Histone H3 (Lys 4) Antibody, 07-539- 1:4000
Millipore Sigma, Anti-acetyl-Histone H3 (Lys14) Antibody, 07-353 - 1:2000
Proteintech, ACLY Polyclonal antibody, 15421-1-AP - 1:1000
Proteintech, IDH2 Polyclonal antibody, 15932-1-AP- 1:5000
Proteintech, OGDH Polyclonal antibody, 15212-1-AP- 1:1000
Abcam, Anti-Citrate synthetase antibody, ab96600 - 1:1000
Abcam, Anti-PDHA1 antibody [8D10E6], ab110334 - 1:1000
BD Biosciences, Purified Mouse Anti-Tom20, 612278 - 1:1000
Cell signaling, GCN5L2 (C26A10) Rabbit mAb #3305, 3305S - 1:1000
Cell signaling, PCAF (C14G9) Rabbit mAb #3378, 3378S - 1:1000
Cell signaling, FoxA1/HNF3α (E7E8W) Rabbit mAb #53528. 53528S - 1:2000, ChIP, 6uL
Cell signaling, c-Jun (60A8) Rabbit mAb #9165, 9165S - 1:1000
Cell signaling, p300 (D8Z4E) Rabbit mAb #86377, 86377S - 1:1000
Cell signaling, CBP (D6C5) Rabbit mAb #7389, 7389S - 1:1000
Invitrogen, Goat anti-Rabbit IgG (H+L) Cross-Adsorbed Secondary Antibody, Alexa Fluor™ 488, A11008 - 1:1000

Invitrogen, Goat anti-Mouse IgG (H+L) Cross-Adsorbed Secondary Antibody, Alexa Fluor™ 488, A11001 - 1:1000
Invitrogen, Goat anti-Rabbit IgG (H+L) Cross-Adsorbed Secondary Antibody, Alexa Fluor™ 594, A11012 - 1:1000
Invitrogen, Donkey anti-Mouse IgG (H+L) Highly Cross-Adsorbed Secondary Antibody, Alexa Fluor™ 594, A21203 - 1:1000
Invitrogen, Goat anti-Rabbit IgG (H+L) Highly Cross-Adsorbed Secondary Antibody, Alexa Fluor™ 647, A-21245 - 1:1000
Invitrogen, Goat anti-Mouse IgG (H+L) Cross-Adsorbed Secondary Antibody, Alexa Fluor™ 647, A-21235 - 1:1000

Validation

All the antibodies used in these paper were validated by the antibodies manufacturers against human or mouse species.

Eukaryotic cell lines

Policy information about cell lines and Sex and Gender in Research

Cell line source(s)

All the cell lines were obtained from ATCC except C4-2, which were a gift from Dr. Weigel, Baylor and GL261, which were a gift from Dr. Michael Ciesielski, Roswell Park.
U87MG: HTB-14
MDA-MB-231: HTB-26
MDA-MB-468: HTB-132
HEK293T: CRL-3216
HELA: CRM-CCL-2
VCaP: CRL-2876
22Rv1: CRL-2505
RWPE-1: CRL-3607
MCF10A: CRL-10317

Authentication

Cell lines were not authenticated.

Mycoplasma contamination

All cell lines were confirmed to be mycoplasma-free using MycoAlert Mycoplasma Detection Kit (Lonza, Catalog# LT07–318).

Commonly misidentified lines
(See ICLAC register)

n/a

Palaeontology and Archaeology

Specimen provenance

Provide provenance information for specimens and describe permits that were obtained for the work (including the name of the issuing authority, the date of issue, and any identifying information). Permits should encompass collection and, where applicable, export.

Specimen deposition

Indicate where the specimens have been deposited to permit free access by other researchers.

Dating methods

If new dates are provided, describe how they were obtained (e.g. collection, storage, sample pretreatment and measurement), where they were obtained (i.e. lab name), the calibration program and the protocol for quality assurance OR state that no new dates are provided.

☐ Tick this box to confirm that the raw and calibrated dates are available in the paper or in Supplementary Information.

Ethics oversight

Identify the organization(s) that approved or provided guidance on the study protocol, OR state that no ethical approval or guidance was required and explain why not.

Note that full information on the approval of the study protocol must also be provided in the manuscript.

Animals and other research organisms

Policy information about studies involving animals; ARRIVE guidelines recommended for reporting animal research, and Sex and Gender in Research

Laboratory animals

NOD.Cg-Prkdcscid Il2rgtm1Wjl/SzJ (NSG) female mice (Age 6-8 weeks, Roswell Park in-house)
NOD.Cg-Prkdcscid Il2rgtm1Wjl/SzJ (NSG) male mice (Age 6-8 weeks, Roswell Park in-house)

Wild animals

n/a

Reporting on sex

n/a

Field-collected samples

n/a

Ethics oversight

Animal experiments were carried out in accordance with protocol 1391M and 1365M, approved by the Roswell Park Institutional Animal Care and Use Committee.

Note that full information on the approval of the study protocol must also be provided in the manuscript.

Clinical data

Policy information about clinical studies

All manuscripts should comply with the ICMJE guidelines for publication of clinical research and a completed CONSORT checklist must be included with all submissions.

Clinical trial registration	n/a
Study protocol	n/a
Data collection	n/a
Outcomes	n/a

Dual use research of concern

Policy information about dual use research of concern

Hazards

Could the accidental, deliberate or reckless misuse of agents or technologies generated in the work, or the application of information presented in the manuscript, pose a threat to:

No	Yes	
☒	☐	Public health
☒	☐	National security
☒	☐	Crops and/or livestock
☒	☐	Ecosystems
☒	☐	Any other significant area

Experiments of concern

Does the work involve any of these experiments of concern:

No	Yes	
☒	☐	Demonstrate how to render a vaccine ineffective
☒	☐	Confer resistance to therapeutically useful antibiotics or antiviral agents
☒	☐	Enhance the virulence of a pathogen or render a nonpathogen virulent
☒	☐	Increase transmissibility of a pathogen
☒	☐	Alter the host range of a pathogen
☒	☐	Enable evasion of diagnostic/detection modalities
☒	☐	Enable the weaponization of a biological agent or toxin
☒	☐	Any other potentially harmful combination of experiments and agents

Plants

Seed stocks	n/a
Novel plant genotypes	n/a
Authentication	n/a

Flow Cytometry

Plots

Confirm that:

- [x] The axis labels state the marker and fluorochrome used (e.g. CD4-FITC).
- [x] The axis scales are clearly visible. Include numbers along axes only for bottom left plot of group (a 'group' is an analysis of identical markers).
- [x] All plots are contour plots with outliers or pseudocolor plots.
- [x] A numerical value for number of cells or percentage (with statistics) is provided.

Methodology

Sample preparation	Nuclei were isolated from human cell line. After in-vitro HAT assay, samples were fixed using formaldehyde and DAPI positive singlet nuclei used for measuring nascent RNA synthesis. No sequential gating strategy used.
Instrument	LSRFortessa
Software	FSC Express 7
Cell population abundance	At least 20000 nuclei were analyzed for fluorescent intensity in the histogram
Gating strategy	DAPI positive nuclei were first selected in DAPI channel, and single nuclei gated by FSC and SSC plots to exclude the clumps.

- [ ] Tick this box to confirm that a figure exemplifying the gating strategy is provided in the Supplementary Information.
