# Supplementary material for: Nuclear-specific reductive carboxylation of alpha-ketoglutarate fuels histone acetylation to induce chromatin accessibility and gene activation
Source: Nat Commun. 2026 Jul 1;17:8216. doi: 10.1038/s41467-026-74786-3 (PMC13462710; doi:10.1038/s41467-026-74786-3)
Supplement: Supplementary file 4 [file 41467_2026_74786_MOESM5_ESM.pdf]

nature portfolio

Peer Review File

Nuclear-specific reductive carboxylation of alpha-ketoglutarate fuels histone acetylation to induce chromatin accessibility and gene activation

Corresponding Author: Dr Subhamoy Dasgupta

This file contains all reviewer reports in order by version, followed by all author rebuttals in order by version.

Version 0:

Reviewer comments:

Reviewer #1

(Remarks to the Author)
Sawant Dessai et al report that “Nuclear-specific reductive carboxylation of alpha-ketoglutarate fuels histone acetylation to induce FOXA1-dependent gene activation”. The authors perform a comprehensive study of the presence of TCA cycle enzymes in the nucleus. Specifically, they describe that ACO2, IDH2 and ACLY localize at the chromatin forming a metabolic complex that includes HAT KAT2A. Moreover, they claim that reductive carboxylation of α-KG by ACO2 and IDH2 in the nucleus fuels histone acetylation, increasing chromatin accessibility, FOXA1 binding at specific proliferation related genes, and their subsequent transcription. While the authors robustly demonstrate the nuclear presence of ACO2 and IDH2 and their impact on local histone acetylation, the functional and biological outcome, regarding chromatin accessibility, altered transcriptional programs, and growth in vivo, are more superficially addressed. Furthermore, although the study is interesting and relevant for the field, I believe the authors should moderate their conclusions regarding functional outcome (last two figures) and regarding novelty, as the presence of metabolic enzymes in the nucleus affecting acetylation or chromatin regulation have been previously reported (Wellen et al, Science 2009; Sdelci et al Nat Gen 2019; Mocholi et al, Cell Rep 2023, among others).

In its current state, the manuscript raises some major issues:
- While the nuclear localization of ACO2 and IDH2 is clearly shown (although number of experiments is not stated), the multispectral immunostaining followed by imaging flow cytometry shows high variability among cell lines, potentially suggesting different levels of dependency on these enzymes for nuclear acetylation or potential regulation of nuclear localization depending on the cellular state. How do the authors explain/address the impact of this variability?
- All the demonstration of histone acetylation by the described metabolic complex in Fig.3 is mainly done in just one cell-line, the MDA-MB-468. Is this also validated in other cell-lines? Also, from 6 histone acetylation marks shown in panel 3b, only 2 are shown in panel 3e and the effect seems to be milder. The rest of histone acetylation marks should be shown to ensure consistency.
- Regarding the metabolic study several concerns arise:
  It is unclear, in the text or methods, whether the HAT assays performed analyze the function of endogenous HATs or imply the addition of recombinant exogenous HATs, please clarify.
  Nuclei isolation for metabolic studies is a milestone of nuclear metabolic studies due to the easy diffusion of metabolites through the nuclear envelope. How did the authors overcome this challenge?
  Experiment in Fig4c is lacking silencing controls showing the downregulation of the enzymes studied and in Figure 4d, there should be a vehicle or non-α-KG control to be able to state that there is an Acetyl-CoA increase upon α-KG addition. Moreover, are AcetylCoA levels also affected by shIDH2 and shACLY?
  In MDA-MB-468 there is an increase in unlabeled citrate (m+0) (Ext data Fig5c), how do the authors explain this?
  Ext data Fig5d, related to GL261 cells, is differently represented, as compared to Ext data Fig5c, same representation (with representation of all different amouns of carbons labelled) and axis labelling is suggested to maintain consistency and avoid confusion. Moreover, α-KG labelling control (m+5) should also be shown for GL261 cells as done in Ext.data Fig5b for MDA-MB-468 cells.
  Regarding in-vitro HAT assay with biotinylated recombinant nucleosomes, how does the mass-spec profile compare to non-labelled histone? Also, does this acetyl (m+2) signal at H3K27decrease in shACO2/shIDH2 cells?
- The chromatin accessibility, transcription and in vivo growth conclusions are overstated based on the quantifiable and

computational data shown:
Regarding the MNase assay, the authors try to quantify the levels o mono-, di-, tri- and tetra-nucleosomes based on the migration on an agarose gel (Ext.data 6c). However, how many experiments are quantified to ensure changes are not due to experimental variability (not stated)? And can the authors really discern whether changes are due to more/less condensed chromatin and not due to MNase digestion efficiency based on initial chromatin amount or chromatin amount loaded after MNase digestion?
In relation to the nascent RNA experiment, how biologically relevant is the difference shown? The authors should represent it as a dot-plot or violin-plot, showing different replicas, average values and deviation.
ATAC-seq analysis and integration are unclear. Why do authors suddenly change to C4-2 cellular model to study this part? From 5804 lost loci mentioned in Fig.5d why are there only 333 taken into consideration in Fig5f for the integration? What is the fold-change established in Fig.5d? If real DARs are 333 (also changes in tornado plots do not mention number of peaks and represent very small change according to scale) and from the integration only the expression of 40 genes is altered, they seem very small numbers for such a general regulatory mechanism described. In this line, GSEAs performed in ATAC-seq and RNA-seq datasets do not seem to show much accordance and the rationale for terms highlighted does not seem evident, authors should try to explain better or analyze more in depth. Also, expression changes shown for MKI67 and IL1RN represent tiny changes according to the scale to show such a high statistical significance (what statistical test has been performed?).
ChIP-QPCR data should be represented as percentage input.
In vivo experiments lack several controls: for instance ACO2, IDH2 and mutant construct expression level controls in tumors and tumor volume data for ACO2 are missing. The former is particularly important considering that doxycycline induced expression in MDA-MB-468 cells (Ext-data Fig4f), showed higher expression of mutant ACO2 as compared to endogenous (same data should also be shown for IDH2), which could explain clonogenic data differences and potentially IDH2 in vivo data, if that were the case. For statistical analysis of mice data, non-parametric analysis (Mann Whitney) is recommended, as normal distribution cannot be assumed. In sum, data shown demonstrates expression of both enzymes in the nucleus is required and sufficient to ensure proliferative capacity of the cells in vivo, but data is lacking to conclude tumorigenic potential as compared to shNT control cells.

Minor issues:
- The authors show that ACO2 may localize at the nucleus by KPNA2-driven translocation. However, no putative mechanism is posed to explain the presence of IDH2 in the nucleus. The authors should at least discuss potential mechanisms in the discussion.
- The IP-MS heatmap showing relative enrichment of histone subunits in Fig. 2h has the same color code in the max and min values of the scale, which makes its interpretation complicated.
- There is a mistake in Ext. data Fig6c, as it refers to quantification of Fig. 4a, but should be Fig. 5a.
- For in vivo experiments tumor growth rate should not be in mm3 (check axis label).

Reviewer #2

(Remarks to the Author)
Comments on Dessai et al.
In this manuscript, the authors propose a novel role for known enzymes of the tricarboxylic acid (TCA) cycle in generating acetyl-CoA at sites of FoxA transcription. Their claims are based on findings of the nuclear localization of citrate synthase (CS), aconitase 2 (ACO2), and isocitrate dehydrogenase 2 (IDH2), which are shown to associate with a known lysine acetyltransferase and all core histone molecules. To substantiate their findings, they demonstrate that these metabolic enzymes are active in isolated nuclei and can synthesize nuclear acetyl-CoA from 2OG, which can then be used for histone acetylation. A knockdown of ACO2 and IDH2 results in increased resistance to MNase digestion and decreased acetylation, suggesting higher chromatin condensation under these conditions. This is corroborated by the observation of many genomic regions with differential accessibility. Furthermore, they identify enriched FOXA1 binding sites within these regions. They also show that a nuclear-specific rescue of a global knockdown of ACO2 and IDH2 stimulates tumor growth. This manuscript describes a series of elegant experiments demonstrating a connection between metabolism and chromatin structure through the localization of TCA enzymes to the nucleus. However, some additional experiments are required to strengthen their conclusions.
Detailed Criticisms:
1. The authors use western blots for quantifying histone acetylation. Given their ability to perform mass spectrometry (MS)-based assays, as indicated in figure 4h, they should use MS-based quantification for all measurements, as it is more reliable.
2. While the authors provide convincing evidence that the nuclear localization of the enzymes can support acetyl-CoA synthesis in vitro, the claim that this occurs in a localized manner in the genome is less convincing. To address this, they should either elucidate the recruitment mechanism (though this may be out of scope) or provide evidence through methods such as interfering with potential interactors or mapping the recruitment of the complex and the acetylation sites affected.
3. A major criticism is the lack of function/rescue experiments using inactive enzyme mutants or inhibitors to unambiguously demonstrate that the observed effects are due to enzyme activity. Control experiments with enzymatically inactive enzymes or the use of inhibitors would address this.
4. A more physiological approach could involve manipulating nuclear NAD+/NADH levels, which should significantly impact

IDH2 activity.
5. It is important to quantify how much this pathway contributes to total histone acetylation, given that such a carbon fixation reaction is rare in organisms outside the plant kingdom. This should be measured and discussed in a revised manuscript.
6. Additional data on the effects of ACO2 or IDH2 knockdown on the cell cycle and general metabolism would help determine whether the observed effects are specific to in situ acetyl-CoA synthesis or are influenced by other factors.
Minor Criticisms:
1. The extended figure 5c shows ample nuclear citrate, which should fuel acetylation without the need for reductive carboxylation. Quantification of this pathway's contribution to total histone acetylation needs to be performed.
2. In Figure 6, it is unclear whether the specific effect on FoxA binding depends on the loss of nuclear ACO2 or IDH2 or if it is also observed with other methods of decreasing nuclear acetyl-CoA, such as ACLY inhibition. The authors suggest that increased acetylation improves FoxA, ESR1, and AR binding. What makes these sites special? Can FoxA co-purify with ACO2, IDH2, or KAT2A? If not, how are these enzymes targeted to specific genomic loci, and what happens if this interaction is blocked?

Reviewer #3

(Remarks to the Author)
The authors explore a ‘moonlighting’ mechanism that directs the local abundance of acetyl-CoA pools by nuclear localization of mitochondrial enzymes ACO2 and IDH2. It is proposed that in the nucleus these enzymes generate citrate which can then be used as a substrate for acetyl-CoA production. Additional data suggests that subsequent histone acetylation and FOXA1 recruitment drives transcriptional responses. Increased nuclear expression of ACO2 and IDH2 was also sufficent to drive tumorigenesis. This is an extensive and interesting study that is in general carried out well. However, there remain several points of attention concerning interpretation of data and the inclusion of experiments to support conclusions that are sometimes based too much on correlations.

General Points

Unfortunaltey there is not sufficient data to support that nuclear localization of metabolic enzymes is critical for epigentic and transcriptional respones. A core conclusion of the study. Most experiments to address this issues are performed by whole cell Sh-mediated knockdowns, followed by adding back nuclear metabolic enzymes. They are often performed in isolated nuclei. This does not demonstrate that nuclear metabolic enzymes are required (under homeostatic conditions) in intact cells. The authors have identified an NLS in ACO2 that they propose is necessary for nuclear translocation. This NLS should be mutated (deltaNLS-ACO2) and shown that there is no translocaton of ACO2 to the nucleus. Under these conditions the effect of deltaNLS-ACO2 on histone acetylation and transcription should be evaluated. This would strongly support the conclusions of the study.

In line with this, there are no meaurements of nuclear metabolites in intact cells. To demonstrate the importance of their findings the authors should show that loss of ACO2/IDH2/ACLY modulates nuclear citrate and acetyl-CoA levels in intat cells. To do this they can emply widely available metabolic biosensors for citrate and acetyl-CoA. By generating nuclear and cytoplasmic reporters, they can directly show the impact of modulating ACO/IDH2 levels on nuclear acetyl-CoA.

The model proposes that ACO2 generates citrate for ACLY that subsequently results in histone acetylation. However, the authors perform almost no experiments to demonstrate this critical role of ACLY. ACLY should be included in the analysis in Figure 1 as it is essential for production of acetyl-CoA, and central to the model proposed. Does driving ACLY to the nucleus, or preventing ACLY nuclear translocaion phenocopy the data presented for ACO2 and IDH2?

All figure legends should include number of independent experiments and statistics where appropriate. This is particulary relevant for Western Blot data, but also many other anayses. Quantification of multiple independent experiments should be performed. This is missing in many if not most experiments. For much of the work it appears that experiments were only performed once.

Proteomics and RNAseq data should be available in open access online repository. At the moment it is impossible to check results or quality while reviewing the manuscript.

Specific Points

Figure 5 and Extended Figure 6-7. It is unclear how these ATAC-seq experiments are performed or how they should be interpreted. Are these whole cell ACO2-KO, or nuclear-specific ACO2-KO. What does ‘KO’ actually mean in this context? From the text it appears to be ‘depletion’ which suggests ‘knock-down’ (KD), but not knockout. This experimental approach needs to be better described and interpreted on basis of either whole cell loss of ACO2 or nuclear-specific loss. Why not use the shACO2+ACO2-dMTS-NLS cell context? This would seem necessary to define a role for nuclear ACO2 in chromatin accessibility and transcription.

Figure 6. The FOXA1 data should be removed from the paper as it doesn’t strengthen the story. To include this data it is necessary to perform a series of additional experiments with FOXA1 KO in vitro and in vivo to demonstrate that it is indeed responsible for mediating the effects of nuclear ACO2. Without this, the data presented is correlative and is not required.

Figure 6d. A significant reduction if IL1RN might be the case but this is so minor that it is hard to understand if this would

have a functional effect. I would remove this rather unconvincing data from the paper. Are these the only two genes that show any significant decrease in mRNA expressoin under these conditions? What about the other 40 genes presented in Figure 6c?

Minor Points

Figure 1b. The authors describe considerable heterogeneity of ACO2 and IDH2 nuclear localization. Have they correlated this with cell division/proliferation? Or do they perhaps have an alternative explanation?

Page 7. The authors write 'ACO2 was identified to have a strong affinity towards ACLY'. There data does not support this statement, they show a non-quantitaive co-IP. The text should be modified to : 'can interact with ACLY'.

Figure 2i. The authors show that ACO2 and IDH2 interact. They further show that ACLY only inreracts with ACO2. How do they explain this? Are there independent ACO2-IDH2 and ACO2-ACLY complexes? In the model in Figure 3h they show that all three proteins are in one complex which doesn't correlate with the data.

The term 'moonlighting' is misleading. It suggests that these metabolic enzymes have an additional function. I would re-phrase this.

Version 1:

Reviewer comments:

Reviewer #1

(Remarks to the Author)
Sawant et al report that "Nuclear-specific reductive carboxylation of alpha-ketoglutarate fuels histone acetylation to induce FOXA1-dependent gene activation". The authors perform a comprehensive study of the presence of TCA cycle enzymes in the nucleus. Specifically, they describe that ACO2, IDH2 and ACLY localize at the chromatin forming a metabolic complex that includes HAT KAT2A.

I thank the authors for addressing most of my previous concerns and I believe the new data and added clarifications strengthen the work.

Some minor concerns remain from the arguments posed in the rebuttal:
- Metabolic analysis: The fact that metabolite measurement is performed in cells after HAT assay should also be mentioned in the manuscript text (apart from the methods section).
- MNase digestion: The added controls increase the quality of the data and strengthen the conclusion. The quantification should be represented as the mean of all replicas performed and added in the manuscript.
-Nascent RNA synthesis: Regarding the representation as a violin plot (which is missing the label of axis Y), unless the quantification is just of one experiment (it would not make sense to have stats there then), I do not understand the authors feeling that it does not represent correctly the result. According to the statistical test, it goes in line with their conclusion and I think, together with the original plot, makes the data more robust.
-ATAC-seq: I understand the rationale of the authors to change the cell line in this case. However, they should at least show the expression changes observed in MKI67 and IL1RN in the cell lines used throughout the paper the paper. Moreover, the information of altered peaks obtained in the ATAC-seq etc, should be mentioned in the ATAC-seq tornado plots (fig. 5e), together with numbers on the axes of the upper plots showing total binding.
In vivo data: The authors disregarded the fact that in vivo statistical analysis should be performed using non-parametric tests. The stats shown (would be better to represent exact p values) do not represent the tumor variability observed. Thus, I insist on repeating the analysis with non-parametric tests. In the case statistical significance is not reached due to the variability, it could also be represented as tumor growth rate, based on calculation of the slope.

Reviewer #2

(Remarks to the Author)
I am impressed by the number of additional data provided by the authors to address my concerns and the one's raised by the other reviewers. They now convincingly show that a substantial amount of acetyl groups on the histones is indeed derived from glutamine, suggesting that this might be an important source of nuclear acetyl-CoA. The NLS mutation further supports the hypothesis of the importance of a nuclear localisation of these enzymes (but not necessarily their activity). I am still not fully convinced that the synthesis happens in situ rather than generally increasing nuclear acetyl-CoA but it is certainly a valid hypothesis. The co-IP shows an interaction, but it does not necessarily prove that the synthesis happens only at these sites. The authors have tempered their statements along these lines.

Reviewer #3

(Remarks to the Author)
The authors have done an excellent job in extensively addressing the reviewers comments.

Version 2:

Reviewer comments:

Reviewer #1

(Remarks to the Author)
I thank the reviewers for addressing all the remaining minor concerns.
Just two notes on my side:
- The axis of the violin plot added in Extended Data Fig. 6e, should go from 0-5000.
- I believe lines 16-17 of page 83 are out of place.

Open Access This Peer Review File is licensed under a Creative Commons Attribution 4.0 International License, which permits use, sharing, adaptation, distribution and reproduction in any medium or format, as long as you give appropriate credit to the original author(s) and the source, provide a link to the Creative Commons license, and indicate if changes were made.
In cases where reviewers are anonymous, credit should be given to 'Anonymous Referee' and the source.
The images or other third party material in this Peer Review File are included in the article's Creative Commons license, unless indicated otherwise in a credit line to the material. If material is not included in the article's Creative Commons license and your intended use is not permitted by statutory regulation or exceeds the permitted use, you will need to obtain permission directly from the copyright holder.
To view a copy of this license, visit https://creativecommons.org/licenses/by/4.0/

Reviewer 1:

Sawant Dessai et al report that “Nuclear-specific reductive carboxylation of alpha-ketoglutarate fuels histone acetylation to induce FOXA1-dependent gene activation”. The authors perform a comprehensive study of the presence of TCA cycle enzymes in the nucleus. Specifically, they describe that ACO2, IDH2 and ACLY localize at the chromatin forming a metabolic complex that includes HAT KAT2A. Moreover, they claim that reductive carboxylation of α-KG by ACO2 and IDH2 in the nucleus fuels histone acetylation, increasing chromatin accessibility, FOXA1 binding at specific proliferation related genes, and their subsequent transcription. While the authors robustly demonstrate the nuclear presence of ACO2 and IDH2 and their impact on local histone acetylation, the functional and biological outcome, regarding chromatin accessibility, altered transcriptional programs, and growth in vivo, are more superficially addressed. Furthermore, although the study is interesting and relevant for the field, I believe the authors should moderate their conclusions regarding functional outcome (last two figures) and regarding novelty, as the presence of metabolic enzymes in the nucleus affecting acetylation or chromatin regulation have been previously reported (Wellen et al, Science 2009; Sdelci et al Nat Gen 2019; Mocholi et al, Cell Rep 2023, among others).

Reviewer #1 found our work robust and comprehensive in demonstrating that mitochondrial enzymes ACO2, IDH2 and ACLY were localized to the nucleus and control histone acetylation by forming a complex with KAT2A. Additionally, reviewer#1 found our study to be very interesting and relevant to the field and provided excellent suggestions to further strengthen our findings with respect to biological and functional outcomes and overall conclusions. To address Reviewer#1’s comments, we performed several additional experiments as suggested, and we are providing here the new data along with point-by-point response to the critics raised.

<u>Major Issues:</u>

While the nuclear localization of ACO2 and IDH2 is clearly shown (although number of experiments is not stated), the multispectral immunostaining followed by imaging flow cytometry shows high variability among cell lines, potentially suggesting different levels of dependency on these enzymes for nuclear acetylation or potential regulation of nuclear localization depending on the cellular state. How do the authors explain/address the impact of this variability?

This is a critical point raised by the Reviewer#1. We agree that our findings clearly demonstrate that there is heterogeneity in the sub-cellular localization of ACO2 and IDH2 both in normal and malignant lines.

- *Experiments in our laboratory are performed with the highest rigor and reproducibility. The <u>sub-cellular fractionation experiments</u> for each cell line had at least three biological replicates (n=3-5) and only reproducible immunoblots demonstrating the sub-cellular distribution of enzymes were presented. The <u>confocal microscopy and immunofluorescence staining</u> of whole cell or isolated nuclei were performed using n=3 biological replicates and images were captured from three different slides for each sample, and only representative images were included in the manuscript. In our <u>image-stream analysis</u> (imaging flow cytometry), we captured individual images at single-cell resolution for n=10,000 cells for each cell line, and the normalized data showing the distribution of the mitochondrial enzymes in nucleus or mitochondria were shown. <u>We have now updated the figure legends section with this information</u>.*
- *The observed variability in nuclear localization across cell lines could be attributed to the known biological diversity among cancer sub-types, which often exhibit distinct metabolic demands, regulatory mechanisms, and signaling pathways [1]. The heterogeneity within the homogenous population of cells indicates that the nuclear-entry of ACO2 and IDH2 could be intricately regulated, and their nuclear functions could be context-dependent, potentially reflecting adaptations to specific metabolic and environmental stress-conditions reflecting their cellular state. At this point, we are carefully characterizing several upstream signals including growth factors, hormones, nutrient signaling, hypoxia, and pH which could potentially trigger nuclear localization. We believe that this variability highlights the complexity of*

metabolic regulation in the nucleus and underscores the need for further investigation. <u>In the discussion section, we have included these perspectives.</u>

- *Further, to address this comment experimentally we have now been able to identify that serum starvation increases nuclear localization of ACO2 and IDH2. This <u>new data</u> has been added as (Extended data Fig. 3j) in the manuscript. Both ACO2 and IDH2 immunoblots clearly indicate that their nuclear localization increased due to serum starvation, whereas mitochondrial levels remain unchanged, suggesting cellular stress signals could instigate nuclear import.*

Manuscript Fig. Extended Data Fig. 3j

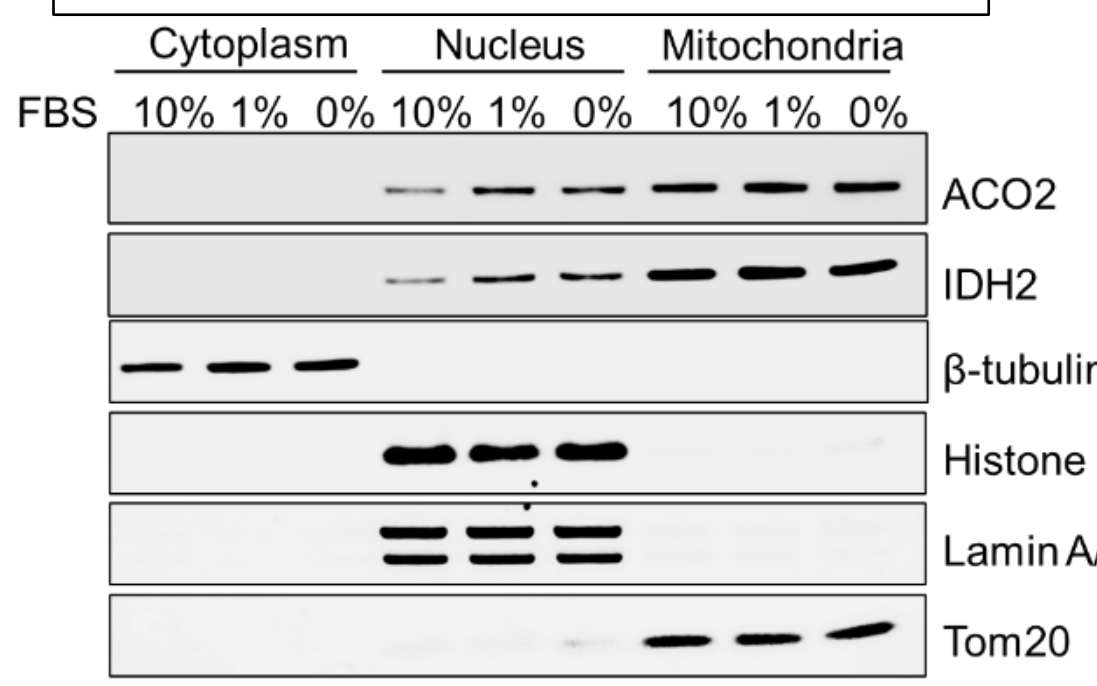

<u>Figure R1</u>: Serum starvation of MDA-MB-468 cells increase nuclear localization of the metabolic enzymes ACO2 and IDH2.

All the demonstration of histone acetylation by the described metabolic complex in Fig.3 is mainly done in just one cell-line, the MDA-MB-468. Is this also validated in other cell-lines? Also, from 6 histone acetylation marks shown in panel 3b, only 2 are shown in panel 3e and the effect seems to be milder. The rest of histone acetylation marks should be shown to ensure consistency.

Reviewer#1 raised an important point regarding the demonstration of histone acetylation changes by the metabolic complex in cell lines beyond MDA-MB-468.

- *In addition to MDA-MB-468 line, we performed stable knockdown of the metabolic enzymes in GL261 cell line followed by acid-extraction of histones to analyze histone acetyl marks. Both MDA-MB-468 and GL261 demonstrated increased levels of ACO2 and IDH2 enzymes in the nucleus by image-stream analysis (Fig. 1c). Data included in <u>Supplementary Fig. 4a-b</u> also demonstrated that stable knockdown of metabolic enzymes in GL261 altered histone acetyl marks. We quantitated the levels of histone acetyl marks compared to the control (shNT), and the heatmap demonstrated that in both MDA-MB-468 and GL261 the alterations in histone acetyl marks due to ablation of metabolic enzymes were comparable (<u>Extended Data Fig. 4c, d)</u>.*

- *Moreover, we have further validated the role of ACO2 modulating histone acetylation in two additional cell lines C4-2 (prostate cancer) and HeLa. CRISPR/Cas9-mediated depletion of ACO2 in C4-2 and HeLa cell lines (<u>Extended Data Fig. 6f)</u> robustly reduced the similar histone acetylation marks compared to controls.*

Manuscript Fig. Extended Data Fig. 4a-d

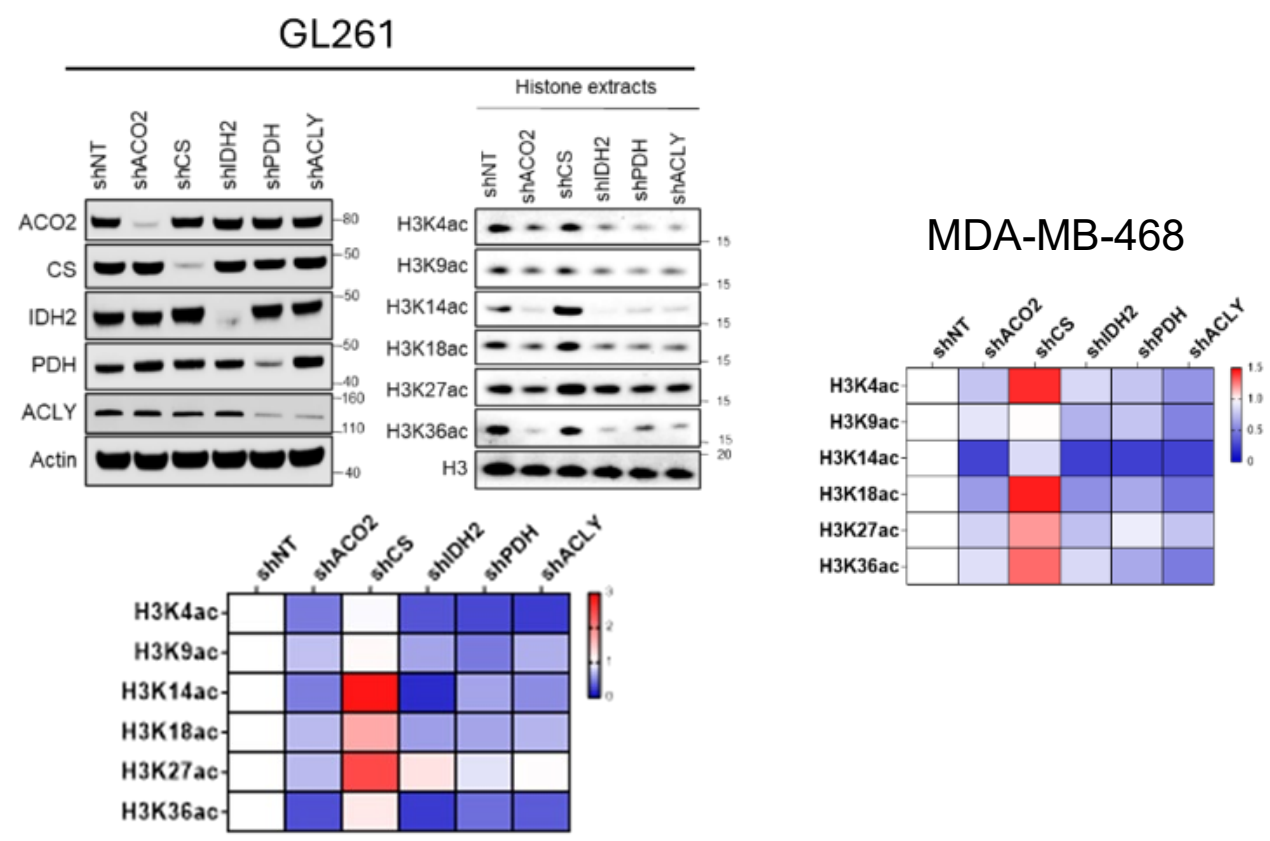

Manuscript Fig. Extended Data Fig. 6f

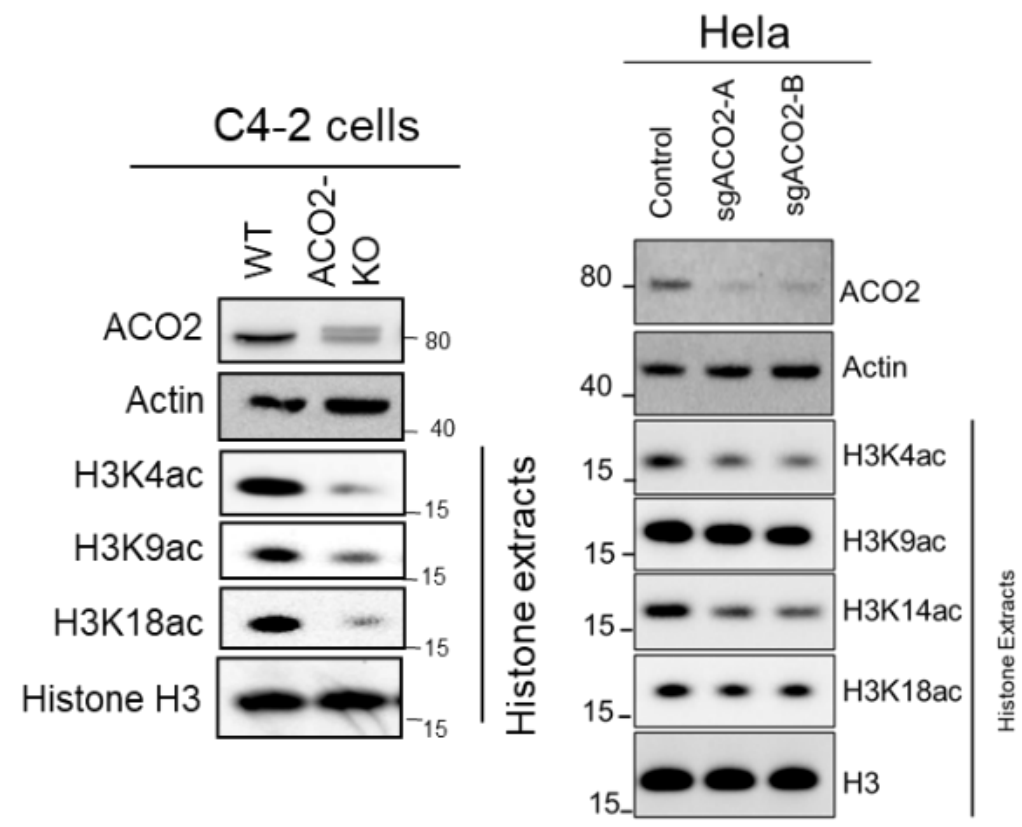

Figure R2: Left: Knockdown of different metabolic enzymes in GL261 cells followed by acid extraction of histones to measure H3Ac marks. Right: ACO2 was knocked out using CRISPR/Cas9 system in C4-2 and Hela cells followed by puromycin selection to generate a pooled population. Acid extracted histones from stable cell were analyzed to measure H3Ac marks. Total Histone H3 used as loading control.

Regarding the metabolic study several concerns arise:

♣ *It is unclear, in the text or methods, whether the HAT assays performed analyze the function of endogenous HATs or imply the addition of recombinant exogenous HATs, please clarify.*

We agree with the reviewer#1 this was not clearly mentioned in our methods and now clearly indicated. We performed HAT assays utilizing the function of endogenous HATs to analyze the impact of nuclear metabolic enzymes ACO2, IDH2 and ACLY.

- *In figure 4b-c, HAT assays were performed using intact nuclei isolated from control (shNT), ACO2, IDH2 and ACLY knockdown cells. In this assay, isolated nuclei from the above-mentioned cells were incubated with indicated metabolite in a HAT-assay buffer using endogenous HATs.*
- *In figure 4f-g and Supplementary fig.5j-k, HAT assays were performed using nuclear lysates isolated from control (shNT) or ACO2, IDH2 and ACLY knockdown cells followed by incubation with recombinant biotinylated nucleosomes along with indicated metabolites. Again, in this experiment we used the function of endogenous HATs.*
- *To support the enzymatic reactions, ATP was supplemented in the assay buffer, since ACLY requires ATP for its activity. Additionally, sodium butyrate was added to inhibit histone deacetylation, ensuring that the measured acetylation levels reflect HAT activity rather than a balance between acetylation and deacetylation processes. All these details have been added to the methods.*

♣ *Nuclei isolation for metabolic studies is a milestone of nuclear metabolic studies due to the easy diffusion of metabolites through the nuclear envelope. How did the authors overcome this challenge?*

We absolutely agree with the reviewer#1, that this has been a major hurdle in the field. Majority of the previous studies have isolated nucleus, mitochondria, cytosol and other organelles followed by LC/MS-based metabolic profiling to determine compartment specific metabolic processes. In this case, easy diffusion of metabolites through the nuclear pores during the isolation process created anomaly in determining nucleus-confined metabolites or metabolic processes.

In our studies, we used isolated intact nucleus from various genetically engineered lines to perform in-vitro HAT-assay in presence or absence of exogenously added metabolites to analyze impact on histone-acetyl marks. We primarily used histone marks as a readout to analyze the impact of upstream nuclear metabolic reactions. Our measurement of nuclear metabolites were detected at the conclusion of the in-vitro HAT assay. Hence, mixing nuclear metabolites with other organelles during the HAT-assay were minimized.

The isolation of intact functional nucleus from the cells was rigorously optimized. Specifically, nuclei were rapidly isolated by directly adding hypotonic buffers to the cell culture plate (as described in the methods). This approach reduced isolation time, avoided freeze-thaw cycles, and ensured uniform exposure of the hypotonic buffer to all cells, thereby improving the quality and integrity of the isolated nuclei. The integrity of the isolated nuclei was confirmed by imaging (Fig. R3), and their purity was validated by both immunoblotting (Figure 1a) and immunofluorescence microscopy (Figure 1h). We used the isolated intact nucleus to perform HAT-assay using the endogenous HAT-enzyme during which essential metabolites such as ATP, acetyl-CoA, and alpha-ketoglutarate were supplemented. For nascent RNA studies, nucleotides were also added.

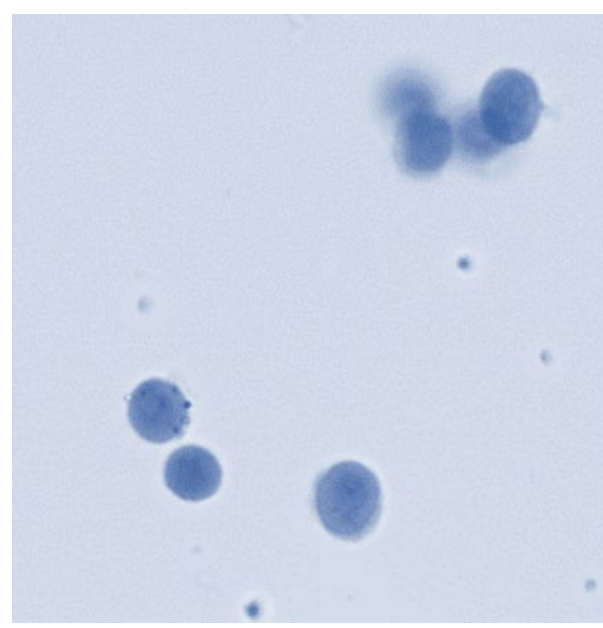

Fig R3. Upon treatment with hypotonic buffer, isolated nuclei from GL261 cells were stained with trypan blue.

♣ Experiment in Fig4c is lacking silencing controls showing the downregulation of the enzymes studied and in Figure 4d, there should be a vehicle or non-α-KG control to be able to state that there is an Acetyl-CoA increase upon α-KG addition. Moreover, are AcetylCoA levels also affected by shIDH2 and shACLY?

We apologize for not including immunoblots demonstrating stable knockdown of the enzymes compared to non-targeting control that were used in Figure. 4c.

- *Lentivirus mediated stable expression of plasmids targeting shNT (control), shACO2, shIDH2, and shACLY (also shCS) were established prior to the in-vitro HAT assay. The immunoblots showing efficacy of knockdown of the enzymes compared to controls are included here in R4, now added in Extended Data Fig. 5c.*

*We agree with reviewer #1 about the importance of vehicle or non-α-KG control in **Fig. 4d**. We performed several additional and parallel experiments along with Fig. 4d, which are now included in the manuscript.*

- *Isolated intact nuclei from glutamine starved cells were treated with vehicle control (H_2O), citrate (2mM), α-KG (2mM), and glutamine(2mM). Addition of α-KG- showed increased levels of acetyl-CoA in the nucleus compared to vehicle control (H_2O), citrate and glutamine. We also conducted a time-course experiment with α-KG, which showed a rapid increase in acetyl-CoA levels until 30 minutes, followed by a decline after 60 minutes, likely due to its utilization by HAT enzymes. Both new data are now added to the manuscript (Extended Data Fig. 5b and d).*

Manuscript Fig. Extended Data Fig. 5c

Manuscript Fig. Extended Data Fig. 5b, d

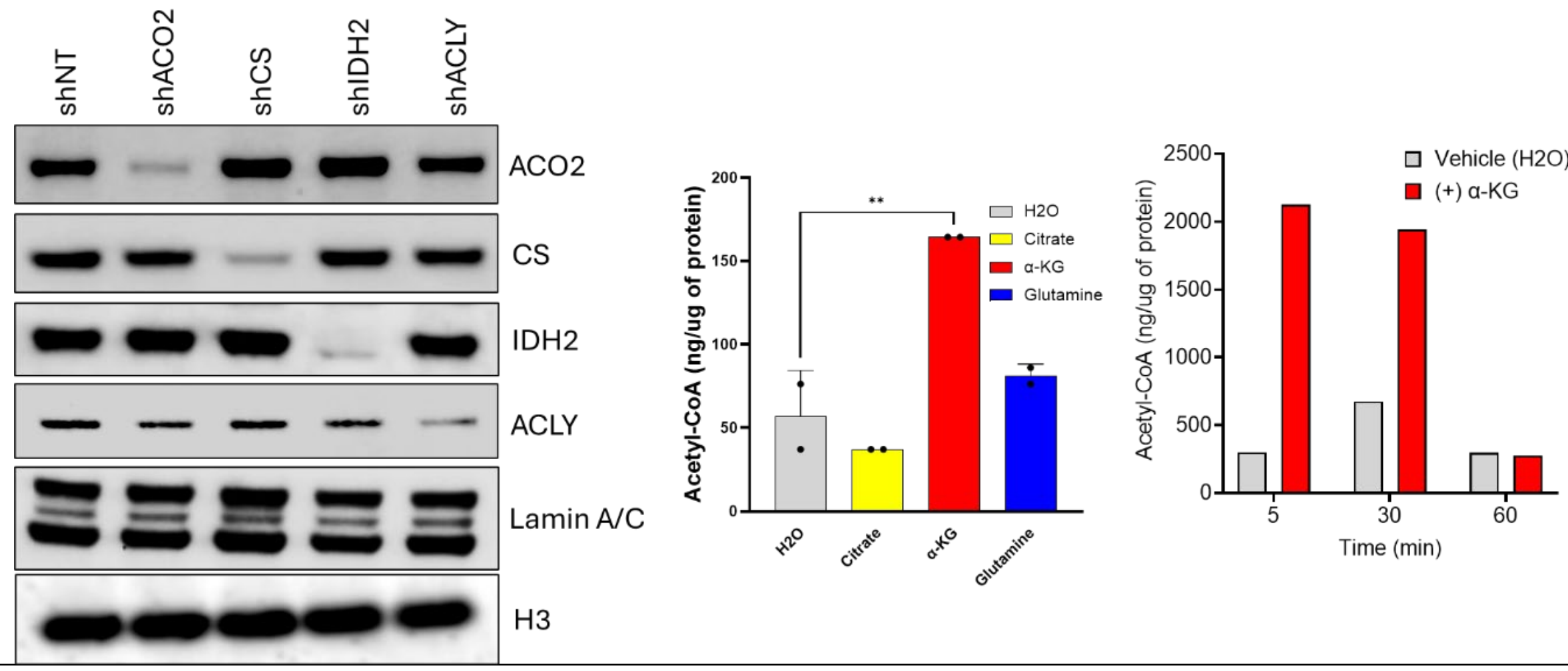

Fig R4. Left) Immunoblotting of nuclear lysates showing knockdown levels of ACO2, CS, IDH2 and ACLY used in Fig. 4c. Lamin A/C and histone H3 are used as a loading control. Middle) Isolated nuclei from glutamine starved GL261 cells were treated with H2O (vehicle control) citrate (2mM), alpha-ketoglutarate (2mM) and glutamine (2mM) for 1hr, followed by acetyl-CoA measurement (normalized to nuclear protein conc). Right) Isolated nuclei from glutamine starved GL261 cells were treated with vehicle control (H2O) or alpha-ketoglutarate (2mM) for 5-60min, followed by acetyl-CoA measurement (normalized to nuclear protein conc).

- *Given the well-documented role of ACLY in regulating nuclear acetyl-CoA [2-6] (as demonstrated by Dr. Wellen's group and others), we primarily focused on determining the nuclear function of ACO2 and IDH2. As shown in Fig. 4c, loss of ACLY also reduced H3Ac marks from citrate indicating the importance in generating acetyl CoA for the HATs.*
- *We also measured steady state levels of nuclear acetyl CoA in stable cells expressing shACO2 and shIDH2 compared to shNT (control). Interestingly, we did not observe a significant change in the steady state levels of acetyl CoA levels between these groups, although there is a trend of reduced acetyl-CoA (Fig. R5). However, we observed significant increase in acetyl CoA levels after addition of α-KG in the isolated nucleus, although the levels of free acetyl CoA levels decreased with time (Fig. R4-right panel). This could be attributed to the rapid utilization of acetyl-CoA by HAT enzymes or the inherent unstable nature of acetyl-CoA, which fails to capture the nascent acetyl CoA levels synthesized.*

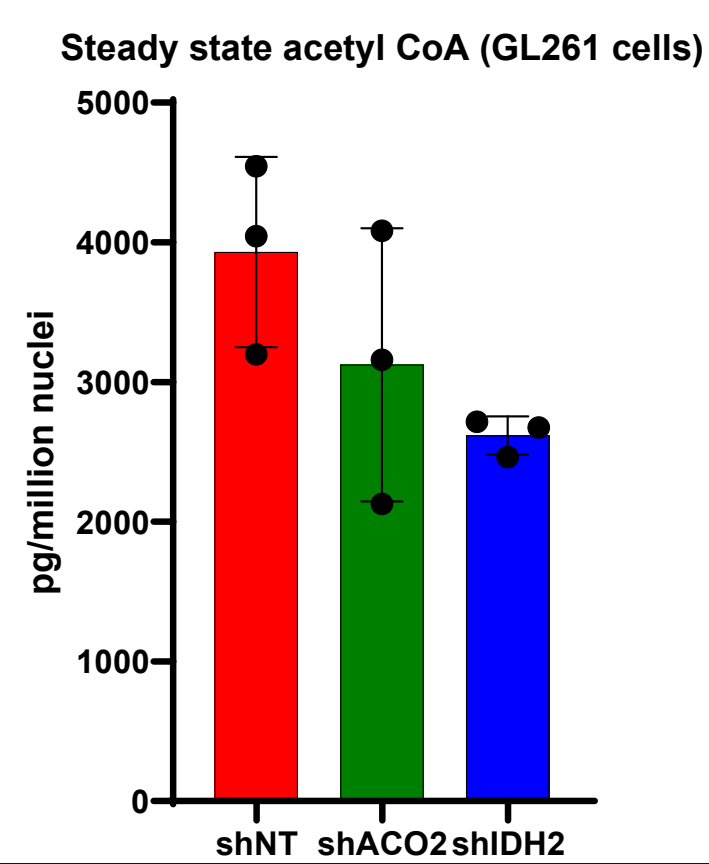

Fig R5. Acetyl-CoA measurement in isolated nucleus from GL261 cells stably expressing shNT, shACO2 and shIDH2.

♣ In MDA-MB-468 there is an increase in unlabeled citrate (m+0) (Ext data Fig5c), how do the authors explain this?

We thank the reviewer for this insightful question. In Ext data Fig. 5c (now Ext Data Fig. 5f), we calculated the percentage of C^{13}-tracers incorporated in citrate isotopomers [such as (m+1), (m+2), (m+3), (m+4), (m+5) and (m+6)] from universally labelled α-KG (U-13C5 α-KG) by measuring the total levels of naturally abundant citrate (C12) and citrate with C13 isotopes, using the formula: % of Incorporation = citrate isotopomer/(13C+12C) x 100. Importantly, shNT nuclei treated with α-KG exhibit significantly higher levels of citrate (m+5), whereas loss of ACO2 or IDH2 significantly reduced the C13 incorporation in citrate (m+5) (Figure. 4e). The remaining citrate isotopomers did not show sufficient enrichment of C13 isotopes from α-KG in the nucleus and no significant differences were observed between the groups. This clearly indicated the existence of a functional nuclear metabolic pathway comprised of ACO2 and IDH2 that converted α-KG into citrate via reductive carboxylation. The apparent increase seen in the percentage unlabeled citrate (m+0) in shACO2 and shIDH2 is due to reduced C13-incorporation into citrate isotopomers, resulting in increased fraction of citrate that were unlabeled. For the reviewers, we also plotted total citrate levels (C^{13} + C^{12}) below in Fig. R6.

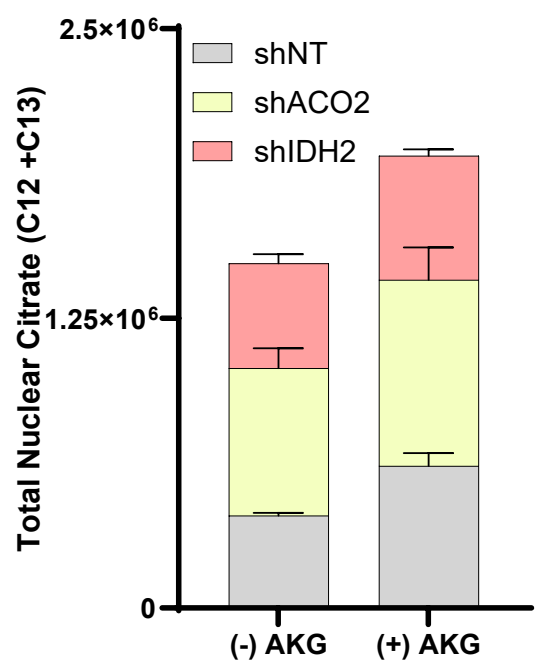

Fig R6. Graph representing total levels of Citrate (C12+C13) in isolated nuclei treated -/+ alpha-ketoglutarate

♣ Ext data Fig5d, related to GL261 cells, is differently represented, as compared to Ext data Fig5c, same representation (with representation of all different amouns of carbons labelled) and axis labelling is suggested to maintain consistency and avoid confusion. Moreover, α-KG labelling control (m+5) should also be shown for GL261 cells as done in Ext.data Fig5b for MDA-MB-468 cells.

As suggested, we have analyzed the citrate isotopomer distribution in GL261. The enrichment patterns of U-13C5 α-KG into citrate were slightly different. Both citrate (m+4) and citrate (m+5) isotopomers were found to enrich C13 carbons in GL261, however only the levels of citrate (m+5) isotopomers were significantly altered due to ACO2 or IDH2 knockdown confirming the reductive carboxylation based nuclear metabolic functions of ACO2 and IDH2. Specifically, we now show the distribution of all labeled citrate isotopomers and have ensured consistent axis labeling across both figures. Additionally, as suggested, we have included the α-KG labeling control (m+5) for GL261 cells in the revised figure, similar to what was shown for MDA-MB-468 cells in Extended Data Fig. 5b. This addition provides a more comprehensive view of the metabolic flux in GL261 cells and ensures consistency in the presentation of labeling data.

Manuscript Fig. Extended Data Fig. 5g

Manuscript Fig. Extended Data Fig. 5i

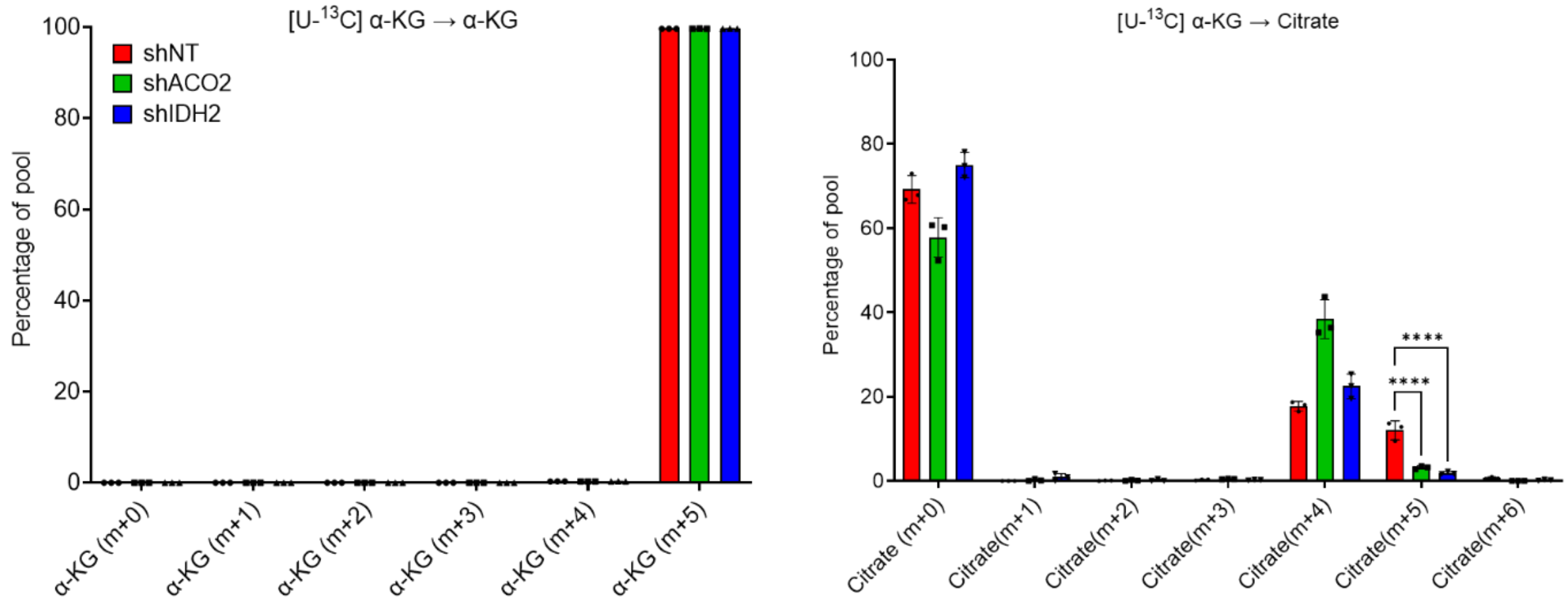

Fig R7. Left: Mass isotope distribution of α-KG obtained by LC/MS analysis of nuclei treated with U-13C5 labeled α-KG (2mM) for 1 hour, isolated from GL261 cells expressing shNT, shACO2, or shIDH2. m is nominal mass. Right: Mass isotope distribution of citrate obtained by LC/MS analysis of nuclei treated with U-13C5 labeled α-KG (2mM) for 1 hour, isolated from GL261 cells expressing shNT, shACO2, or shIDH2. m is nominal mass. n = 3, **** P ≤ 0.0001, P-values were calculated by two-way ANOVA with Tukey's multiple comparisons test. Error bars are presented as mean ± SD.

♣ Regarding in-vitro HAT assay with biotinylated recombinant nucleosomes, how does the mass-spec profile compare to non-labelled histone? Also, does this acetyl (m+2) signal at H3K27decrease in shACO2/shIDH2 cells?

This is an extremely important point, and now we have performed a comprehensive analysis to address Reviewer 1's comment. For this we collaborated with Drs. Leah Gates (expert in epigenetics) and Peder Lund (expert in mass spectrometry) at Case Western University. Drs. Gates and Lund have previously established isotope tracing followed by proteomics methods to trace the source of elemental carbons of histone acetyl marks[7, 8].

*To determine the impact of endogenous nuclear ACO2 on acetylation of histones, we employed MDA-MB-468 cells expressing shNT (control), shACO2, and shACO2 cells with dox-inducible expression of ∆MTS-NLS-ACO2 and cultured them in presence of U-C13-glutamine for 2hours. The cells were harvested, washed, and histones were extracted by acid extraction followed by LC/MS proteomics to measure the enrichment of C13-acetyl (m+2) on histone marks. Our data revealed that approximately **30-40% of H3Ac and H4Ac marks were enriched with C13 from glutamine compared to non-labelled histones**, and loss of ACO2 significantly reduced H3K9Ac, H3K14Ac, H3K23Ac, H4K8Ac, and H4K12Ac marks. Moreover, restoring the levels of ACO2 in the nucleus using dox-inducible expression of ∆MTS-NLS-ACO2 rescued most of the H3Ac and H4Ac marks that were significantly decreased due to loss of ACO2, confirming the functional role of nuclear ACO2 regulating histone acetylation. These experiments were performed with whole cells to study the endogenous ACO2 functions. Hence, we replaced the previous data obtained using the recombinant nucleosome and nuclear lysates, since we believe this new data decodes the endogenous nuclear functions of ACO2 in whole cells and the tracing studies is more comprehensive.*

Manuscript Fig. 4j

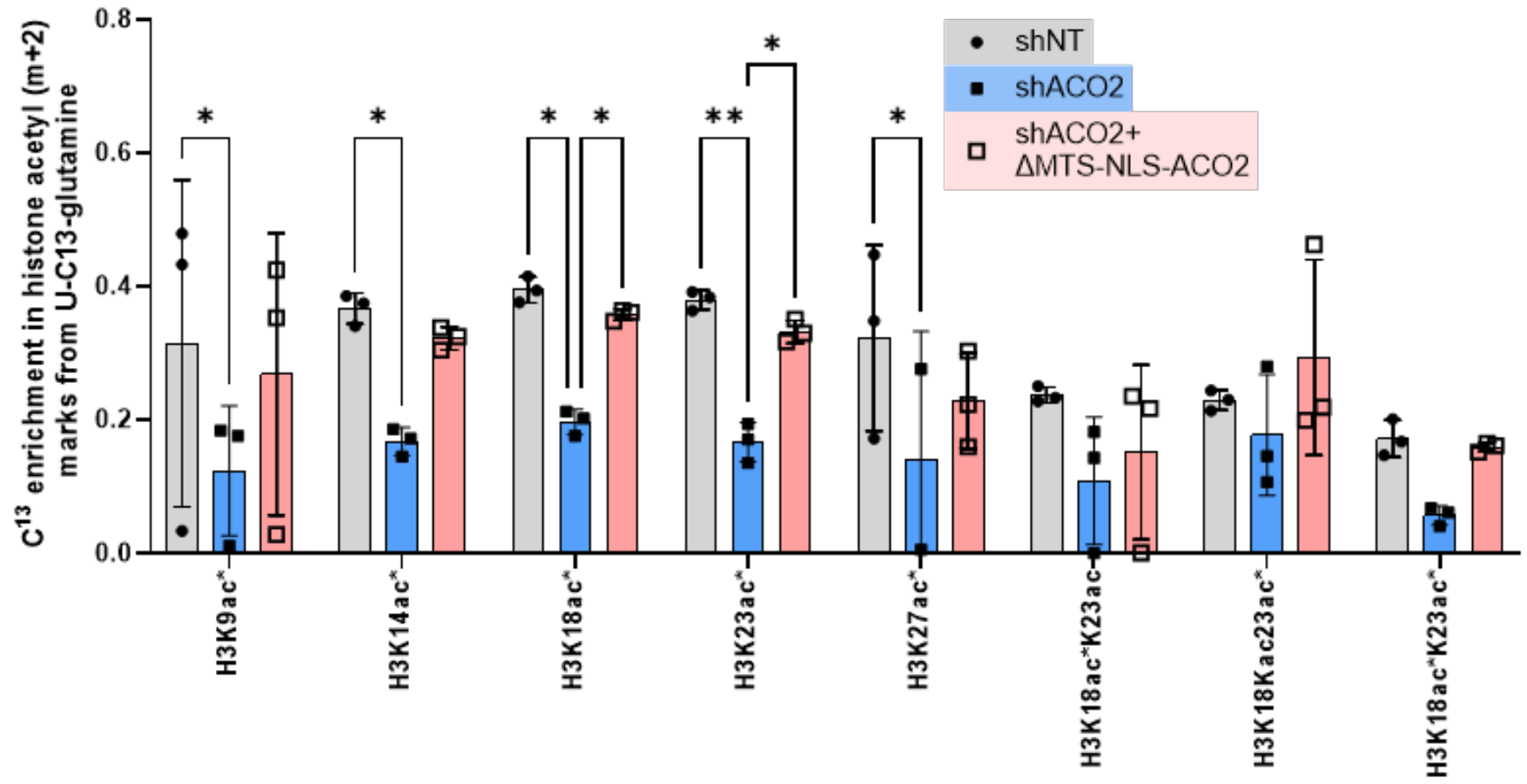

Fig R7. Fractional enrichment of acetyl C[13] (m+2) on H3Ac marks from U-$^{13}C_5$-Glutamine in MDA-MB cells stably expressing shNT, shACO2 and shACO2 cells with ACO2-ΔMTS-NLS. Error bars are presented as mean ± SD. * P ≤ 0.05, ** P ≤ 0.01. *n=3* biological replicates.

- The chromatin accessibility, transcription and in vivo growth conclusions are overstated based on the quantifiable and computational data shown:

♣ Regarding the MNase assay, the authors try to quantify the levels o mono-, di-, tri- and tetra-nucleosomes based on the migration on an agarose gel (Ext.data 6c). However, how many experiments are quantified to ensure changes are not due to experimental variability (not stated)? And can the authors really discern whether changes are due to more/less condensed chromatin and not due to MNase digestion efficiency based on initial chromatin amount or chromatin amount loaded after MNase digestion?

We thank the reviewer for their insightful questions regarding the MNase assay and the interpretation of chromatin condensation changes. Below, we provide additional details to address the concerns raised.

1. ***Reproducibility and Experimental Variability**:*
   The MNase digestion experiments were performed three times (n=3) in both GL261 and MDA-MB-468 cell lines to ensure reproducibility. A representative image from these experiments is included in the manuscript (Extended Data Fig. 6c). To minimize variability, we used an equal number of isolated nuclei under standardized conditions, and performed MNase digestion with increasing MNase enzyme concentrations (GL261: 0.25U, 0.5U and 1.0U and for MDA-MB-468: 0U, 0.25U, and 0.5U in 100uL MNase buffer). We found that 0.5U was optimal for MDA-MB-468, whereas GL261 required 1.0U of MNase to get mononucleosomes. After digestion, DNA was purified using phenol/chloroform extraction method and quantified using a Nanodrop, and equal concentration of DNA (1ug) was loaded onto agarose gels for analysis. The consistent results across replicates support the robustness of our findings (Fig. R8).

2. ***Chromatin Condensation vs. MNase Digestion Efficiency**:*
   To address the concern about whether the observed changes are due to chromatin condensation or differences in MNase digestion efficiency, we performed additional validation using the Agilent TapeStation system, which provides higher sensitivity and resolution compared to agarose gel electrophoresis. Digested DNA was quantified using Qubit to ensure equal loading, and equal amounts of DNA were analyzed on the TapeStation. As shown in the updated figure below (Fig. R8), the 0 U MNase control samples (undigested) show equal DNA amounts across conditions, confirming consistent starting material. In contrast, samples treated with 0.25 U and 0.5 U MNase show reduced intensities of mono-, di-, and tri-nucleosomes in shACO2, shIDH2, and shACLY samples compared to

the shNT control. These results suggest that the observed changes are due to differences in chromatin accessibility and condensation rather than variations in MNase digestion efficiency.
These additional experiments and controls strengthen our conclusions and address the reviewer's concerns regarding experimental variability and interpretation. These data only added here to address the reviewer's concerns.

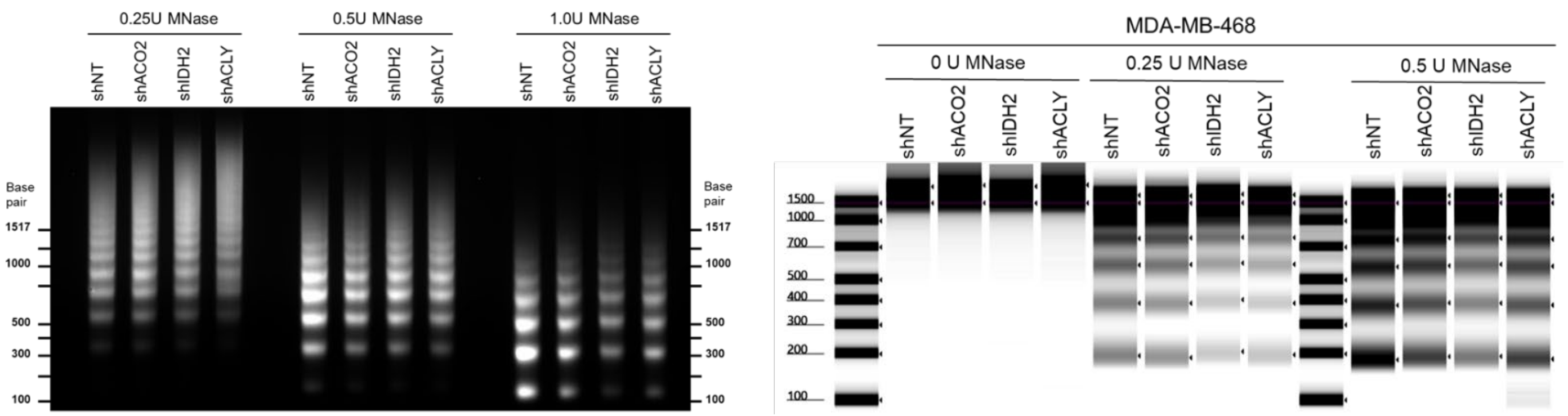

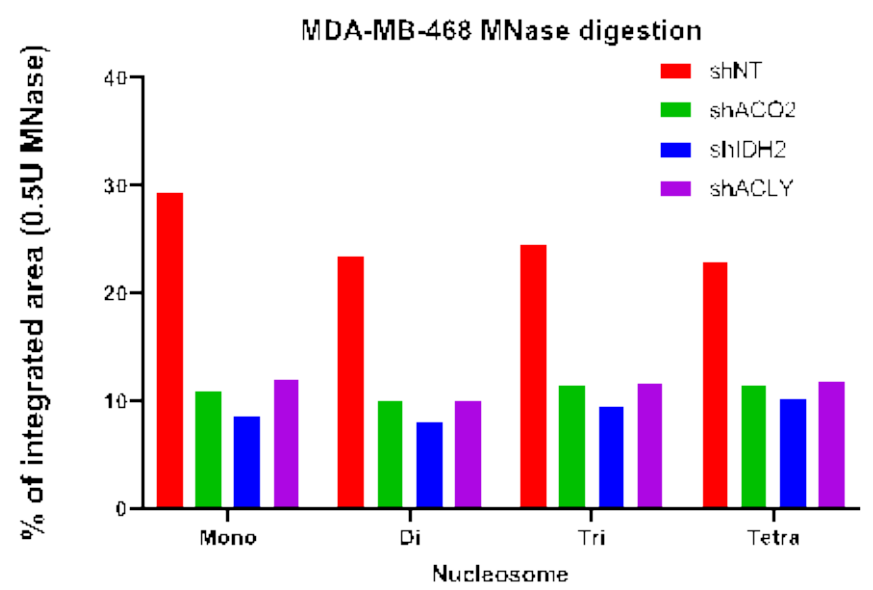

Fig R8. Left: Agarose gel analysis of MNase digested DNA (1ug) from GL261 nuclei treated with increasing conc of MNase (0.25U, 0.5U and 1.0U in 100uL MNase buffer). Right: Tap-station analysis of MNase digested DNA from MDA-MB-468 treated with increasing conc of MNase (0U, 0.25U and 0.5U in 100uL MNase buffer). Bottom: Quantification of MNase digestion efficacy.

♣ In relation to the nascent RNA experiment, how biologically relevant is the difference shown? The authors should represent it as a dot-plot or violin-plot, showing different replicas, average values and deviation.

Here is the raw data associated with the nascent RNA experiment and the graph shown in the manuscript was directly obtained from the flow software.

Histogram #	Filename	Parameter	Low bound	High bound	# of Events	% of gated cells	Median	Geometric Mean	CV
1	GL261 shNT + AKG.fcs	FITC-A	-658.88	262144	26797	100	4876.28	4821.62	34.88
2	GL261 shACO2 + AKG.fcs	FITC-A	-1145.23	262144	26672	100	3978.13	3963.16	36.13
3	GL261 shIDH2 + AKG.fcs	FITC-A	-2148.46	262144	24758	100	3613.9	3779.14	35.96
4	GL261 shACLY +AKG.fcs	FITC-A	-703.61	262144	26039	100	4070.43	4094.78	36.2

If we stack all the geometric mean or median from three different runs, and plot it as a violin or dot plot (Fig. R9), we don't feel that the data represents the altered nascent RNA synthesis observed. So, we would like to **keep the original graph in the manuscript (Fig. 5c)** and upload all the associated raw data and replicates.

Manuscript Fig. 5c

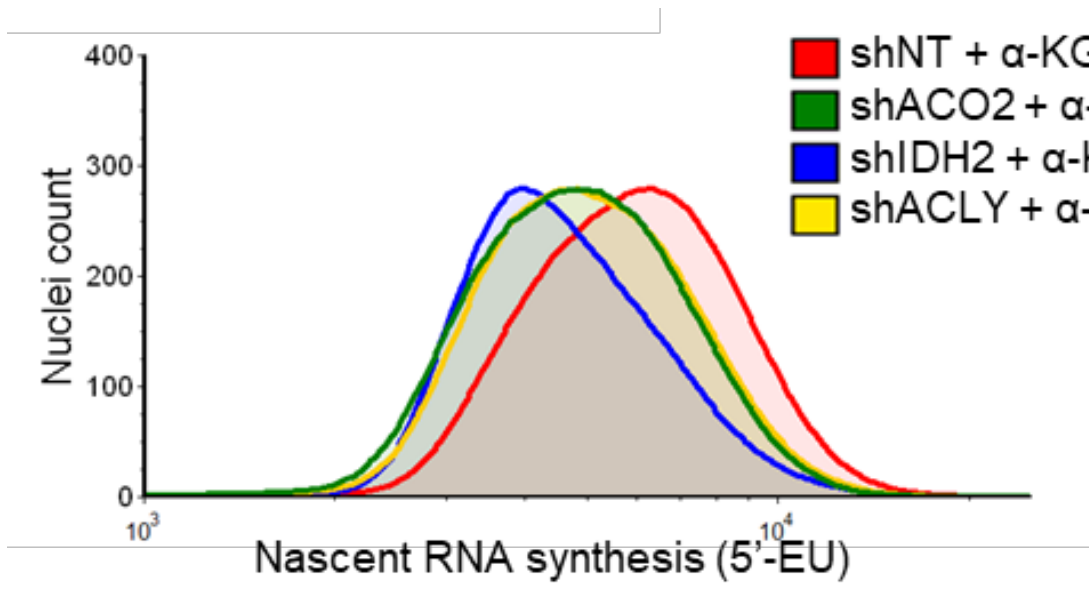

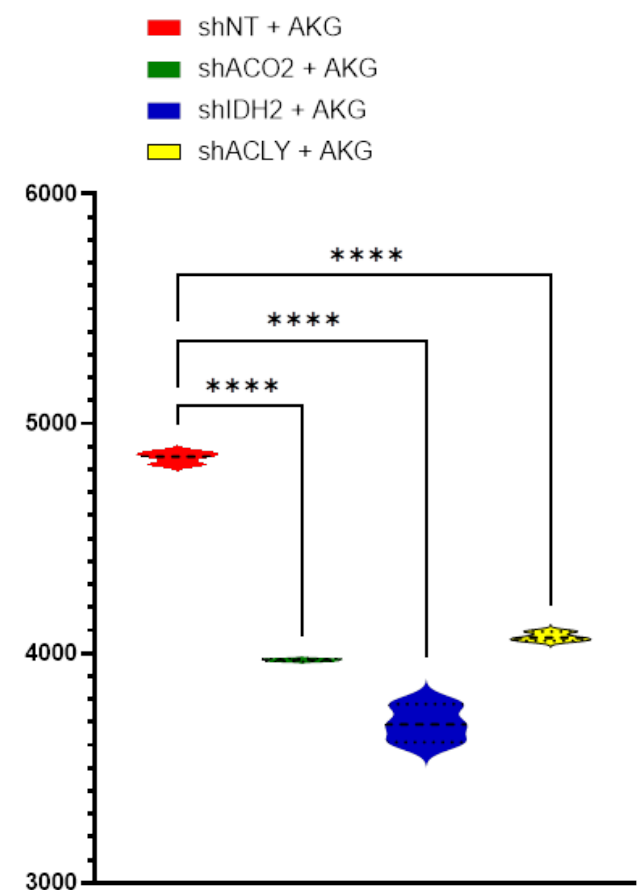

Fig R9. Left: Original plot as obtained from the flow data analysis. Violin plot showing the differences in nascent RNA synthesis.

♣ ATAC-seq analysis and integration are unclear. Why do authors suddenly change to C4-2 cellular model to study this part? From 5804 lost loci mentioned in Fig.5d why are there only 333 taken into consideration in Fig5f for the integration? What is the fold-change established in Fig.5d? If real DARs are 333 (also changes in tornado plots do not mention number of peaks and represent very small change according to scale) and from the integration only the expression of 40 genes is altered, they seem very small numbers for such a general regulatory mechanism described. In this line, GSEAs performed in ATAC-seq and RNA-seq datasets do not seem to show much accordance and the rationale for terms highlighted does not seem evident, authors should try to explain better or analyze more in depth. Also, expression changes shown for MKI67 and IL1RN represent tiny changes according to the scale to show such a high statistical significance (what statistical test has been performed?).

The rationale for selecting C4-2 line to study impact of ACO2, was based on our previous publication where we found that ACO2 loss reduces prostate tumor growth (Cancer research, 2021). So, the impact on chromatin accessibility could be linked to a phenotype.
In Fig. 5d, we identified 5804 loci that were lost in ACO2-KO compared to WT using an FDR ≤ 0.05 cutoff without applying a fold-change threshold. However, to focus on the most biologically significant changes and reduce noise, we applied a more stringent cutoff for downstream analysis. This included an FDR ≤ 0.05 and a fold-change > 1.5 within 5 kb of promoter regions, which narrowed the list to 333 genes. This stringent approach allowed us to identify specific and high-confidence regulatory mechanisms.
While the integration analysis revealed changes in the expression of 40 genes, it is important to note that these genes represent a subset of the broader regulatory impact of ACO2 on chromatin. As highlighted by the MNase digestion assay, histone acetylation mark analysis, and ATAC-seq data, the loss of ACO2 has a widespread effect on chromatin accessibility and structure. The 40 genes identified are those with the most pronounced changes in expression linked to chromatin accessibility, but they do not fully capture the extent of ACO2's regulatory role.
Expression of MKI67 and IL1RN were chosen as representative genes regulated at both the levels of chromatin and gene expression. Plots shown in Figure 5h and Extended Figure 7e are meant to show relative expression differences between groups, with axes representing regularized log transformed expression counts, and fold change cannot be readily estimated from these plots. Using DESeq2 method of fold change estimation and the Wald test for hypothesis testing, MKI67 shows a relative fold change of -1.72 (adjusted p-value = 0.000227), and IL1RN a relative fold change of -5.05 (adjusted p-value = 6.284E-06) in ACO2-KO vs WT. Thus, these gene changes we believe are meaningful and correlate with loss of accessibility with ACO2 loss of function.

♣ ChIP-QPCR data should be represented as percentage input.

As requested by reviewer#1, the ChIP data has now been represented as percentage of input (Figs. 6f, g).

Manuscript Fig. 6f-g

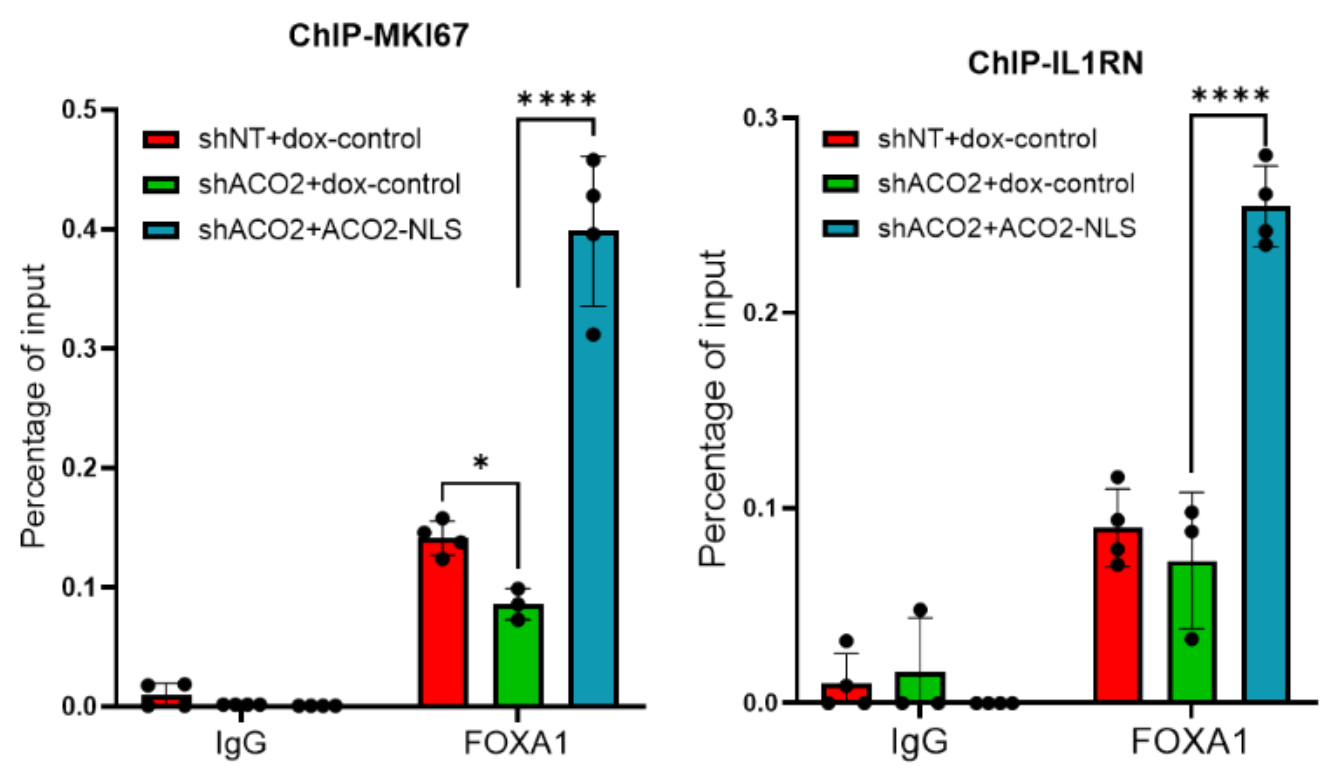

Fig. R10. Promoter occupancy of FOXA1 on Ki67 and IL1-RN.

♣ In vivo experiments lack several controls: for instance, ACO2, IDH2 and mutant construct expression level controls in tumors and tumor volume data for ACO2 are missing. The former is particularly important considering that doxycycline induced expression in MDA-MB-468 cells (Ext-data Fig4f), showed higher expression of mutant ACO2 as compared to endogenous (same data should also be shown for IDH2), which could explain clonogenic data differences and potentially IDH2 in vivo data, if that were the case. For statistical analysis of mice data, non-parametric analysis (Mann Whitney) is recommended, as normal distribution cannot be assumed. In summary, data shown demonstrates expression of both enzymes in the nucleus is required and sufficient to ensure proliferative capacity of the cells in vivo, but data is lacking to conclude tumorigenic potential as compared to shNT control cells.

Expression Level Controls in Tumors:

We would like to point out here that prior to injecting in mice, we generated stable cell lines expressing ACO2-ΔMTS-NLS and IDH2-ΔMTS-NLS using lentiviral transduction followed by blasticidin antibiotic selection. To avoid potential biases associated with single-clone phenotypes, we used pooled cell populations for all downstream experiments, including cell fractionation, cell proliferation assays, and tumor growth studies. As shown in Figure 3c, ACO2 levels in ACO2-ΔMTS-NLS are comparable with shNT in the nucleus, as are nuclear IDH2 levels in shNT and IDH2-ΔMTS-NLS expressing cells. In contrast, nuclear levels of ACO2 and IDH2 in shACO2 and shIDH2 are significantly lower respectively. These results confirm that the rescue constructs restore nuclear ACO2 and IDH2 levels to those observed in control cells.

To further address reviewers comments we performed immunoblotting using the some of the mouse tumors to confirm the relative levels of ACO2 and IDH2 in all the three groups (Extended Fig. 8d-e). The new data confirmed that the expression levels of IDH2 and ACO2 are comparable in shNT and ΔMTS-NLS expressing cells, whereas cells with knockdown had lower expression. Since bulk tumor samples were used, residual levels of ACO2 or IDH2 were seen in knockdown tumors most likely from tumor infiltrating cells.

Extended Fig. 8d-e

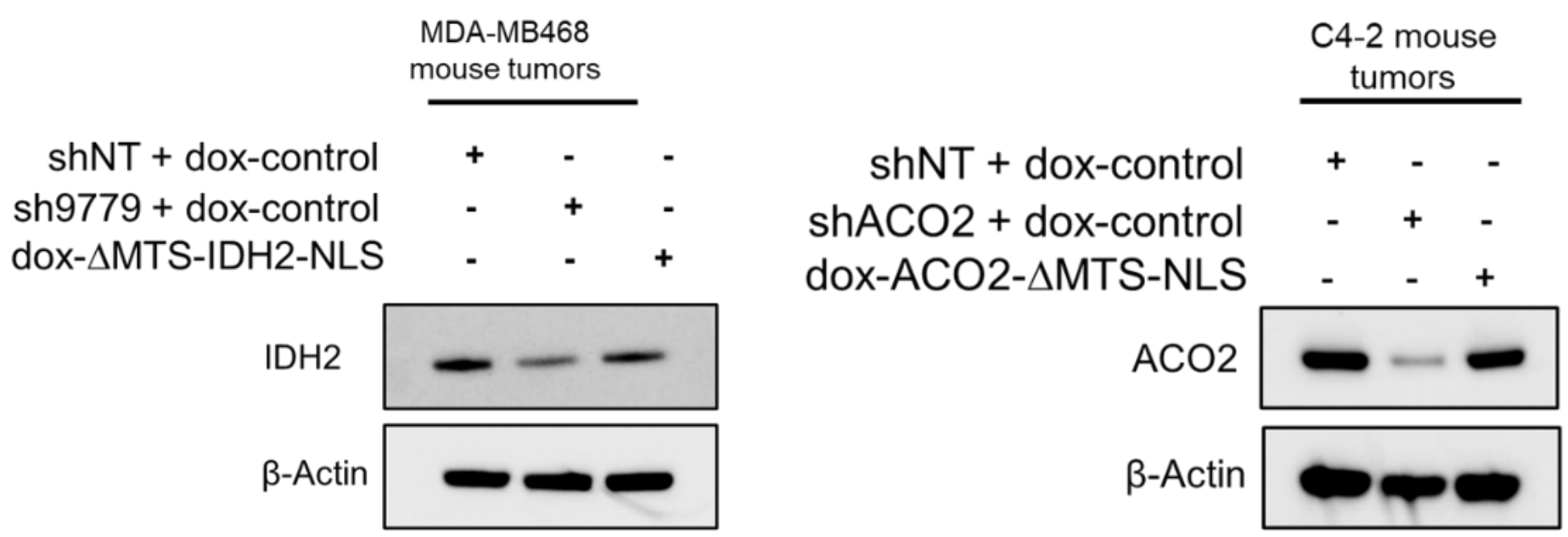

Fig. R11. Immunoblotting showing levels of ACO2 and IDH2 from mouse tumors.

Tumor Volume:

We have now included tumor volume data for both ACO2 and IDH2 groups across the entire period of the study (Extended Data Fig. 8f-g)This supports the conclusion that nuclear ACO2 and IDH2 are required for robust tumor growth. While some variation between replicates is observed, the overall trend is consistent and statistically significant.

Extended Fig. 8f-g

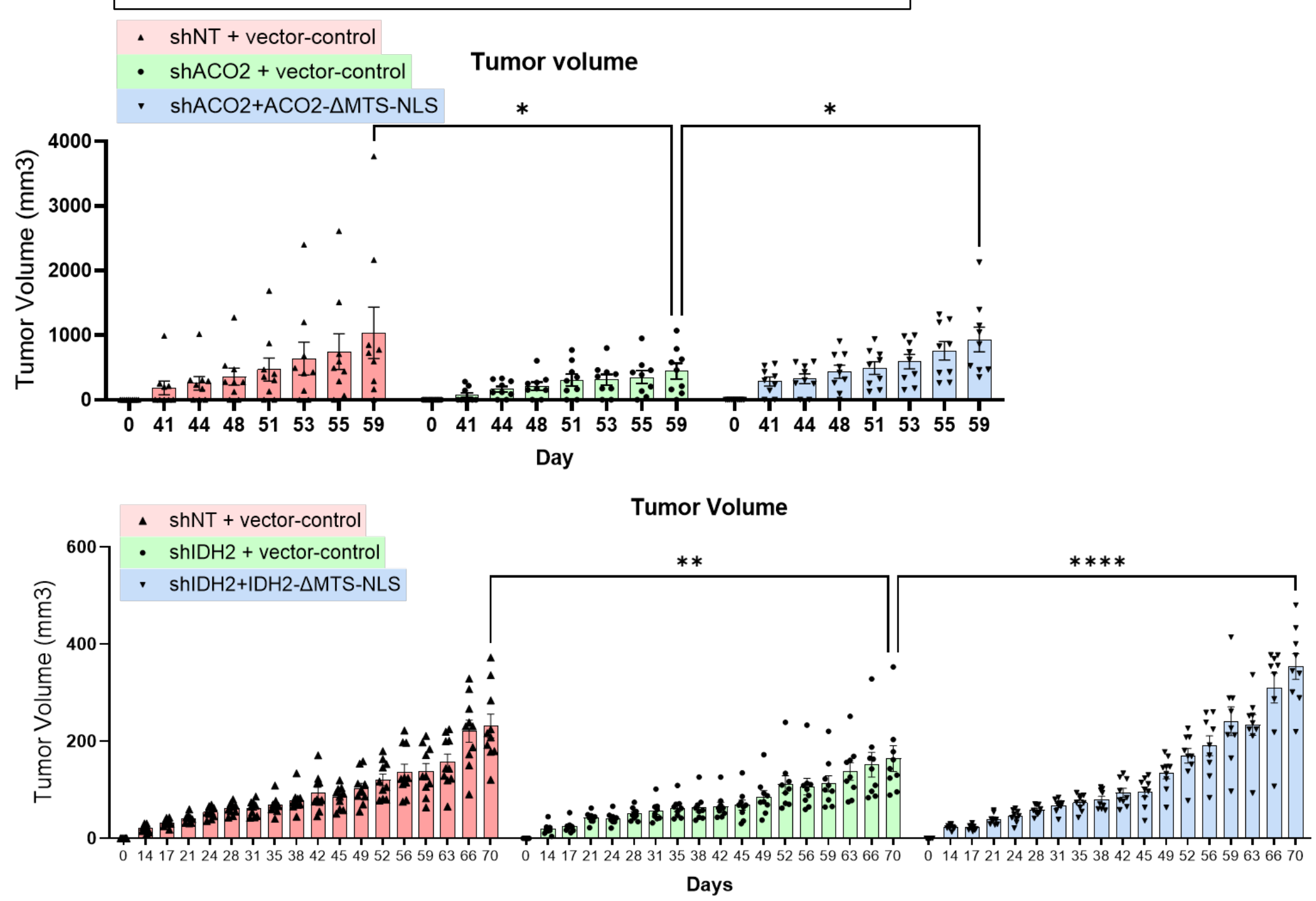

Fig. R12. Tumor growth curves obtained after implantation of the engineered lines.

Additionally, we have now performed IHC for Ki67 using the harvested tumors and included the H&E images.

Fig. 7f, g, h, i)

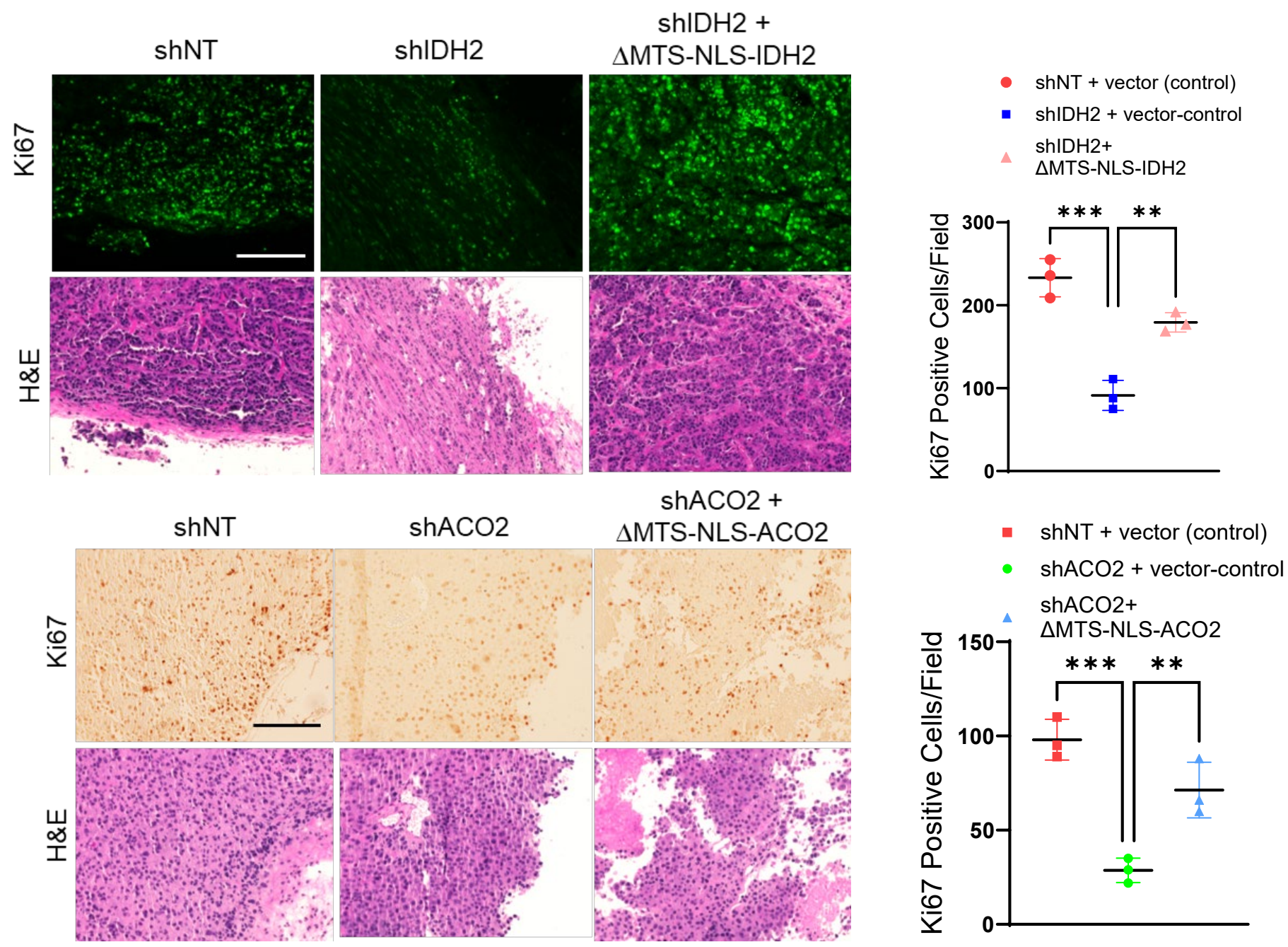

Fig. R13. IHC images showing Ki67 staining to demonstrate the growth potential.

Minor issues:

- The authors show that ACO2 may localize at the nucleus by KPNA2-driven translocation. However, no putative mechanism is posed to explain the presence of IDH2 in the nucleus. The authors should at least discuss potential mechanisms in the discussion.

-Added in the discussion.

- The IP-MS heatmap showing relative enrichment of histone subunits in Fig. 2h has the same color code in the max and min values of the scale, which makes its interpretation complicated.

-We have changed the color to represent it better.

- There is a mistake in Ext. data Fig6c, as it refers to quantification of Fig. 4a, but should be Fig. 5a.

Thank you for pointing this out. This has been corrected

- For in vivo experiments tumor growth rate should not be in mm3 (check axis label).

This has been corrected as well.

Reviewer #2 (Remarks to the Author):

Comments on Dessai et al.
In this manuscript, the authors propose a novel role for known enzymes of the tricarboxylic acid (TCA) cycle in generating acetyl-CoA at sites of FoxA transcription. Their claims are based on findings of the nuclear localization of citrate synthase (CS), aconitase 2 (ACO2), and isocitrate dehydrogenase 2 (IDH2), which are shown to associate with a known lysine acetyltransferase and all core histone molecules. To substantiate their findings, they demonstrate that these metabolic enzymes are active in isolated nuclei and can synthesize nuclear acetyl-CoA from 2OG, which can then be used for histone acetylation. A knockdown of ACO2 and IDH2 results in increased resistance to MNase digestion and decreased acetylation, suggesting higher chromatin condensation under these conditions. This is corroborated by the observation of many genomic regions with differential accessibility. Furthermore, they identify enriched FOXA1 binding sites within these regions. They also show that a nuclear-specific rescue of a global knockdown of ACO2 and IDH2 stimulates tumor growth. This manuscript describes a series of elegant experiments demonstrating a connection between metabolism and chromatin structure through the localization of TCA enzymes to the nucleus. However, some additional experiments are required to strengthen their conclusions.

We like to thank the Reviewer for appreciating our work and finding our manuscript elegant. Reviewer#2 provided us with excellent suggestions and constructive feedback to strengthen our work by performing some additional experiments. We performed all the suggested experiments, and the new data is provided here. We strongly believe these new set of data further substantiated our findings.

Detailed Criticisms:
1. The authors use western blots for quantifying histone acetylation. Given their ability to perform mass spectrometry (MS)-based assays, as indicated in figure 4h, they should use MS-based quantification for all measurements, as it is more reliable.

As suggested by the reviewer, we performed MS-based quantification to further validate our observations. In fig 4h, we previously showed by MS-based quantification that recombinant nucleosomes enrich acetyl marks originating from alpha-ketoglutarate. Now, we have performed MS-based quantification of endogenous histone acetyl marks and traced the source of acetyl carbons from glutamine. For this, we established a collaboration with Drs. Leah Gates (expert in epigenetics) and Peder Lund (expert in mass spectrometry) at Case Western University. Drs. Gates and Lund have previously established isotope tracing followed by proteomics methods to trace the source of elemental carbons of histone acetyl marks[7, 8].

Experimental details: To determine the impact of endogenous nuclear ACO2 on acetylation of histones, we employed MDA-MB-468 cells expressing shNT (control), shACO2, and shACO2 cells with dox-inducible expression of ∆MTS-NLS-ACO2 and cultured them in presence of U-C13-glutamine for 2hours. The cells were harvested, washed, and histones were extracted by acid extraction followed by LC/MS proteomics to measure the enrichment of C13-acetyl (m+2) on histone marks. Our data revealed that H3Ac marks were enriched with C13 from glutamine, and loss of ACO2 significantly reduced H3K9Ac, H3K14Ac, H3K23Ac and H3K27 Ac marks. Moreover, restoring the levels of ACO2 in the nucleus using dox-inducible expression of ∆MTS-NLS-ACO2 rescued H3K14, K18 and K23Ac marks that were significantly decreased due to loss of ACO2, confirming the functional role of nuclear ACO2 regulating histone acetylation. These experiments were performed with whole cells to study the endogenous ACO2 functions in addition to the experiments previously demonstrated with isolated nucleus. Hence, the MS-based quantifications recapitulated the changes in H3Ac marks observed with immunoblotting.

Manuscript Fig. 4j

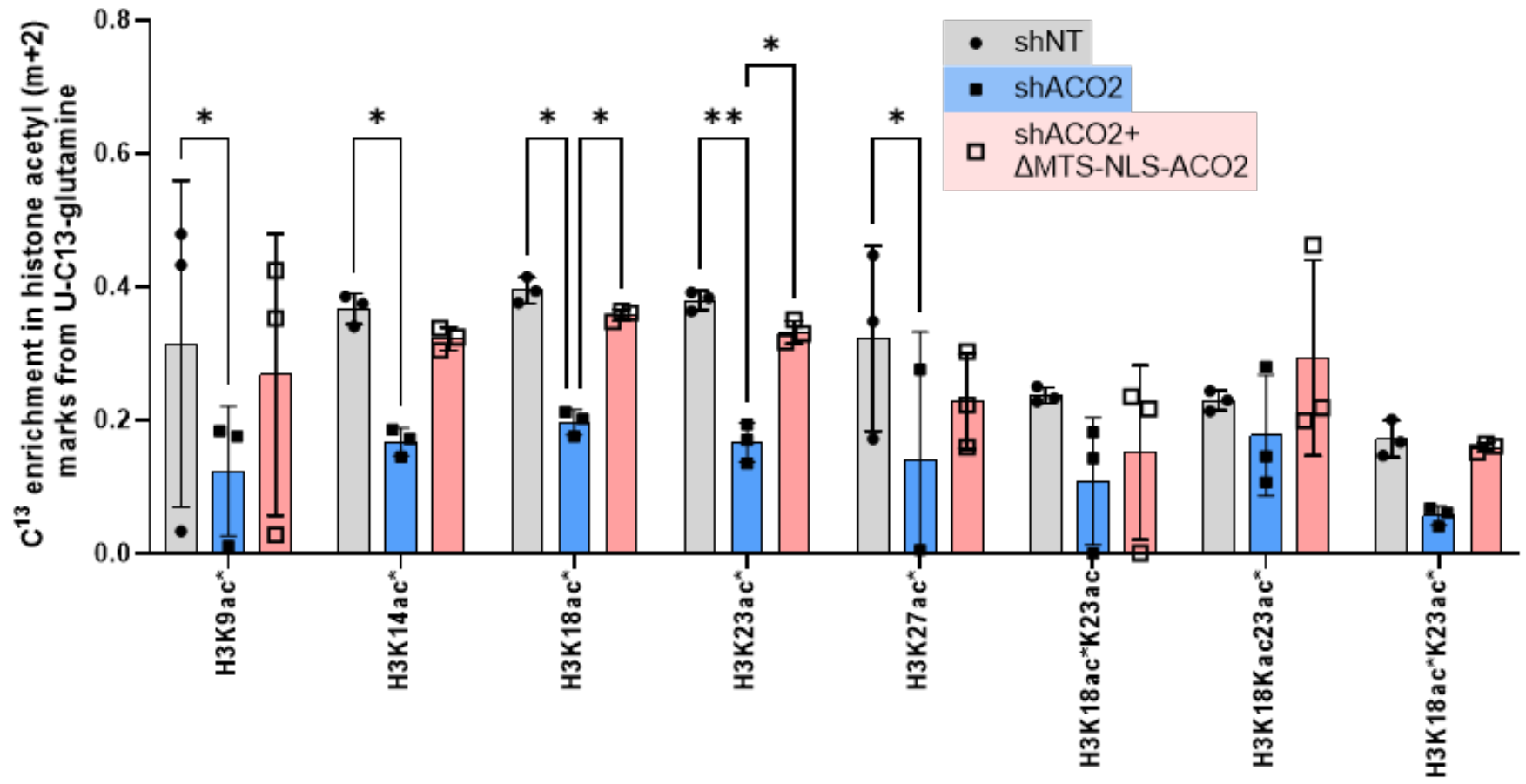

Fig R7/14. Fractional enrichment of acetyl C^{13} (m+2) on H3Ac marks from U-$^{13}C_5$-Glutamine in MDA-MB cells stably expressing shNT, shACO2 and shACO2 cells with ACO2-ΔMTS-NLS. Error bars are presented as mean ± SD. * $P \leq 0.05$, ** $P \leq 0.01$. *n=3* biological replicates.

2. While the authors provide convincing evidence that the nuclear localization of the enzymes can support acetyl-CoA synthesis in vitro, the claim that this occurs in a localized manner in the genome is less convincing. To address this, they should either elucidate the recruitment mechanism (though this may be out of scope) or provide evidence through methods such as interfering with potential interactors or mapping the recruitment of the complex and the acetylation sites affected.

Thanks for appreciating the convincing demonstration of the nuclear localization of metabolic enzymes, which was the primary objective of this study. We do agree with the reviewer that additional studies are needed to support the site-specific recruitment of these enzymes at the genomic loci as well as upstream signaling that stimulates this complex on chromatin. While a detailed elucidation of the recruitment mechanism is beyond the current scope of this study, our IP/IB data from nuclear lysates indicated that the ACO2-IDH2-ACLY complex specifically interacts with KAT2A, but not with other major histone acetyltransferases such as p300 or CBP (Figure 3f–g). This specificity suggests that these metabolic enzymes may be preferentially incorporated into a KAT2A-containing chromatin regulatory complex, potentially contributing to more spatially restricted acetyl-CoA synthesis and acetylation events along with FOXA1. To provide further evidence in this line (and to address your #minor point 2), we performed IP/IB of ACO2, IDH2 and KAT2A with FOXA1 from the nuclear lysates to determine

Manuscript Fig. 6h-k

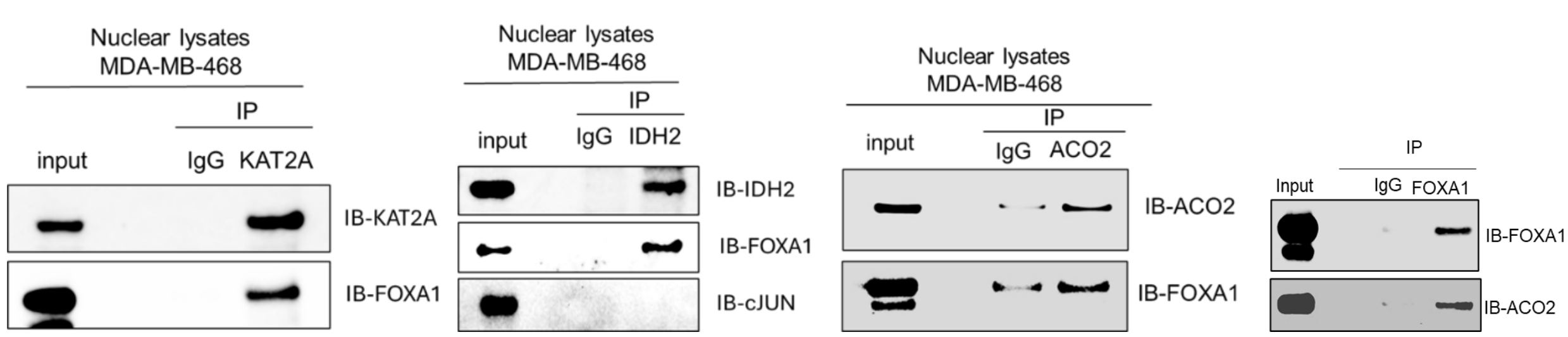

Fig R15. Western blot analysis of immunoprecipitated IDH2, ACO2 and KAT2A from nuclear lysates from MDA-MB-468, showing their interaction with FOXA1. IgG was used as antibody control.

whether KAT2A-containing complex of ACO2/IDH2 interacts with FOXA1. Our data revealed that KAT2A-chromatin regulatory complex containing ACO2-IDH2 both interacts with FOXA1 but not cJUN, suggesting FOXA1-specific genomic loci may be the primary targets in the cell lines that we have investigated. We are further investigating this in the context of prostate cancer, where FOXA1 is associated with aggressive disease. We have revised the manuscript text to clarify this point and temper our conclusions accordingly.

3. A major criticism is the lack of function/rescue experiments using inactive enzyme mutants or inhibitors to unambiguously demonstrate that the observed effects are due to enzyme activity. Control experiments with enzymatically inactive enzymes or the use of inhibitors would address this.

*We thank the reviewer for bringing up this important point. We agree that including catalytic-dead mutants or pharmacological inhibitors would have further strengthened that ACO2 or IDH2 are important for regulating the observed metabolic functions or histone acetylation. However, it may not be enough to distinguish nuclear functions from mitochondrial metabolic roles of these enzymes. So, to address these critical points raised and also as suggested by reviewer#3, we generated mutations in the endogenous NLS domain. First we identified and confirmed that the nuclear localization signal (NLS) of ACO2 is located at the C-terminus (position 29) "**RAKVAMSHFEPNEYIHYDLLEKNINIVR**". To confirm that this predicted bipartite NLS is critical for translocating the enzyme to the nucleus, we mutated the 'K' and 'R' residues (indicated in red) to alanine 'A' and expressed the mutant NLS (mNLS-ACO2) in ACO2-deficient (shACO2) cells followed by lentiviral transduction and stable selection of polyclonal pooled population. The mNLS-ACO2 expressing stably selected cells were subjected to cellular fractionation to determine subcellular distribution of ACO2 compared to control cells expressing WT-ACO2. Our data revealed that mNLS-ACO2 expressing cells failed to enter the nucleus compared to the control (WT) confirming the bipartite NLS is important for nuclear localization of ACO2.*

Manuscript Extended Fig. 4h-j

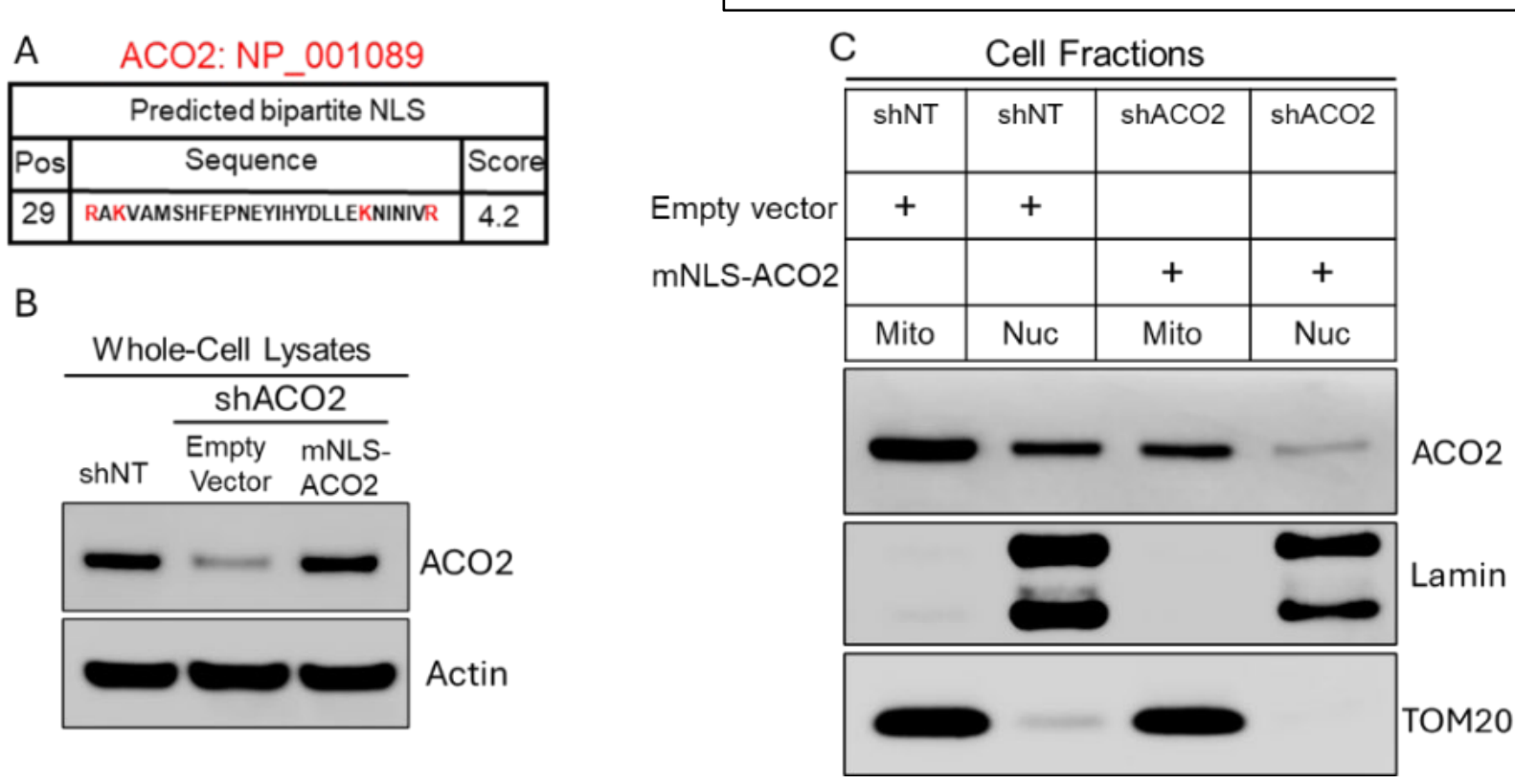

Fig. R16. A: Predicted NLS domain of ACO2. B: Levels of ACO2 shown by immunoblotting. C. Immunoblotting with cellular fractions showing the distribution of endogenous ACO2 (wild type) and ACO2 with mutated NLS (mNLS) between mitochondria and nucleus. Lamin A/C and Tom20 were used as nuclear, and mitochondrial marker proteins.

4. A more physiological approach could involve manipulating nuclear NAD+/NADH levels, which should significantly impact IDH2 activity.

We agree that redox balance could influence IDH2 activity and could control epigenetic marks. In the reductive carboxylation process, IDH2 uses NADPH and converts it to NADP during the catalysis of alpha-ketoglutarate to isocitrate. Manipulating NADPH/ NADP or NAD kinases by pharmacological approaches could lead to broad cellular effects, hence we performed nuclear-specific rescue experiments while simultaneously blocking mitochondrial functions, to more directly assess the nuclear contributions of IDH2. But we agree with reviewer that cellular stress response and redox state could physiologically regulate the nuclear metabolic functions, and we are now investigating the upstream signals or cellular stress signals that could trigger nuclear metabolic functions.

5. It is important to quantify how much this pathway contributes to total histone acetylation, given that such a carbon fixation reaction is rare in organisms outside the plant kingdom. This should be measured and discussed in a revised manuscript.

Thank you for raising this important point (also your minor point#1). As discussed earlier, in collaboration with Drs. Gates and Lund, we traced the source of elemental carbons of acetyl marks on histones from glutamine using isotope tracers followed by MS-based quantification. Our data revealed that compared to total H3 Ac marks (labeled + unlabeled), approximately 30-40% of the acetyl marks enrich C^{13} from glutamine (U-glutamine-C^{13}). Upon ACO2 depletion, several H3Ac such as K9Ac, K14Ac, K18Ac, K23Ac and K27Ac showed significantly reduced C^{13} enrichment around 10-20%. Moreover, these marks were significantly rescued by the expression of ACO2-∆MTS-NLS confirming the nuclear functions of ACO2 are essential for histone acetylation. Based on these data from isotope tracing experiments followed by proteomics, we can conclude that approximately 40% of the histone acetyl marks rely on nuclear ACO2 mediated metabolic functions to fix carbons on the histone acetyl marks from glutamine (Fig. R7/R14).

6. Additional data on the effects of ACO2 or IDH2 knockdown on the cell cycle and general metabolism would help determine whether the observed effects are specific to in situ acetyl-CoA synthesis or are influenced by other factors.

We appreciate the reviewer's suggestion to examine potential indirect effects through cell cycle or metabolic changes. To address this, we performed detailed cell cycle analyses in ACO2/IDH2 knockdown cells and nuclear-specific rescue lines, which revealed no significant differences compared to control cells. Most notably, while α-KG serves as an essential cofactor for multiple chromatin-modifying enzymes (including histone/DNA demethylases), supplementation with α-KG alone in ACO2/IDH2-deficient nuclei failed to rescue the chromatin accessibility defects (Figure 5a). This key observation demonstrates that nuclear ACO2 and IDH2 are specifically required to utilize α-KG for histone acetylation. Together, these results strongly indicate that: (1) the observed acetylation defects are not secondary to cell cycle alterations, and (2) the nuclear metabolic pathway we identified functions distinctly from other α-KG-dependent processes. These data have been added here to address the reviewers' concerns.

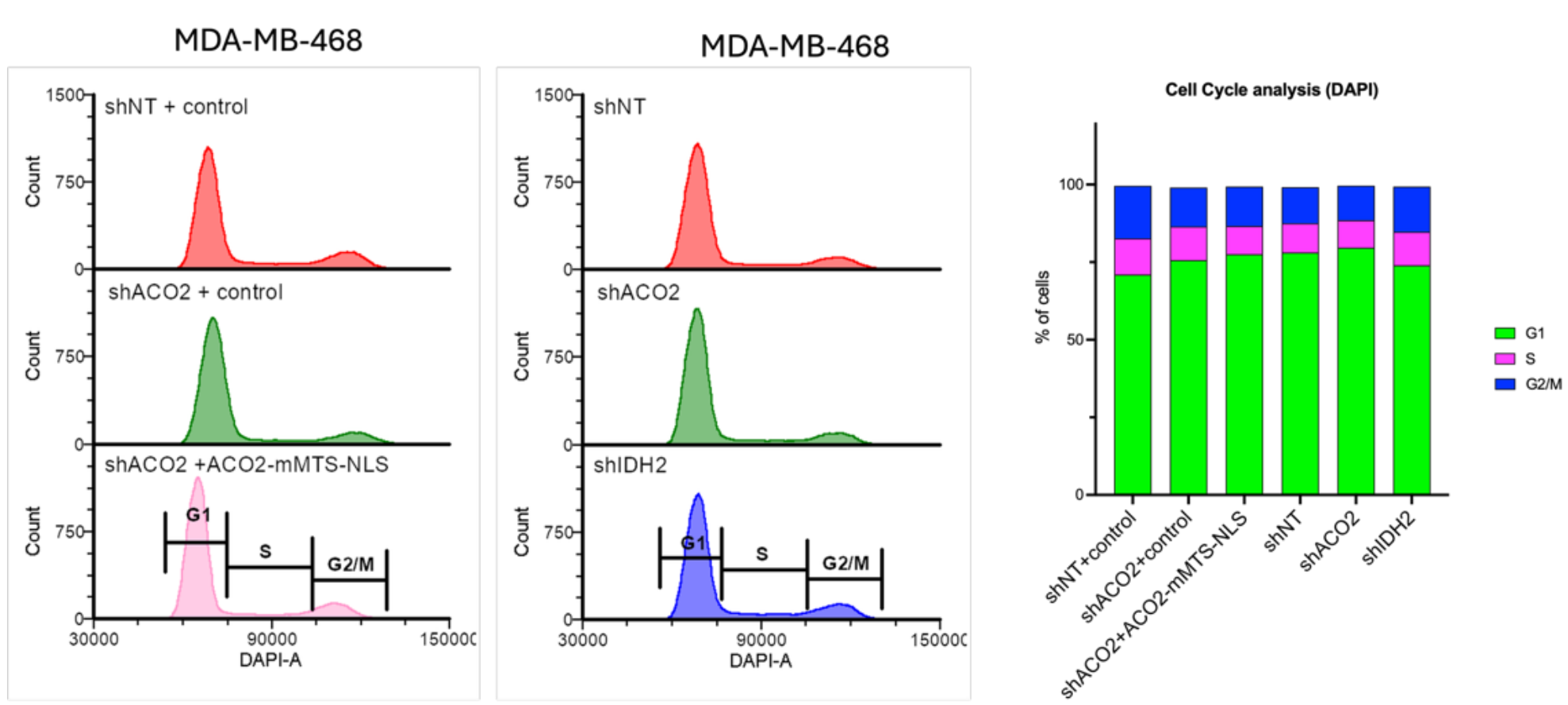

Fig. Graph showing DNA content of MDA-MB-468 cells based on DAPI intensity analyzed by flow cytometry. Cells were gated into G1, S and G2/M phase based on DAPI intensity. Percentage of cells in each phase are shown in bar graph.

Minor Criticisms:

1. The extended figure 5c shows ample nuclear citrate, which should fuel acetylation without the need for reductive carboxylation. Quantification of this pathway's contribution to total histone acetylation needs to be performed.

-Thanks for bringing this point out. As discussed earlier (in major point #5), the pathway contributes to 30-40% of histone acetylation from glutamine as the carbon source, in the given timepoint. This has been discussed in the manuscript.

2. In Figure 6, it is unclear whether the specific effect on FoxA binding depends on the loss of nuclear ACO2 or IDH2 or if it is also observed with other methods of decreasing nuclear acetyl-CoA, such as ACLY inhibition. The authors suggest that increased acetylation improves FoxA, ESR1, and AR binding. What makes these sites special? Can FoxA co-purify with ACO2, IDH2, or KAT2A? If not, how are these enzymes targeted to specific genomic loci, and what happens if this interaction is blocked?

As discussed in major point#2, we performed IP/IB of ACO2, IDH2 and KAT2A from the nuclear lysates to determine whether KAT2A-containing complex of ACO2/IDH2 interacts with FOXA1 or cJUN, both were predicted TFs from the ATACseq motif analysis. Our data revealed that KAT2A-chromatin regulatory complex containing ACO2-IDH2 both interacts with FOXA1 but not cJUN, suggesting FOXA1-specific genomic loci may be the primary targets in the cell lines that we have investigated. We are further investigating this in the context of prostate cancer, where FOXA1 is associated with aggressive disease. We have revised the manuscript text to clarify this point and temper our conclusions accordingly.

Manuscript Fig. 6h-k

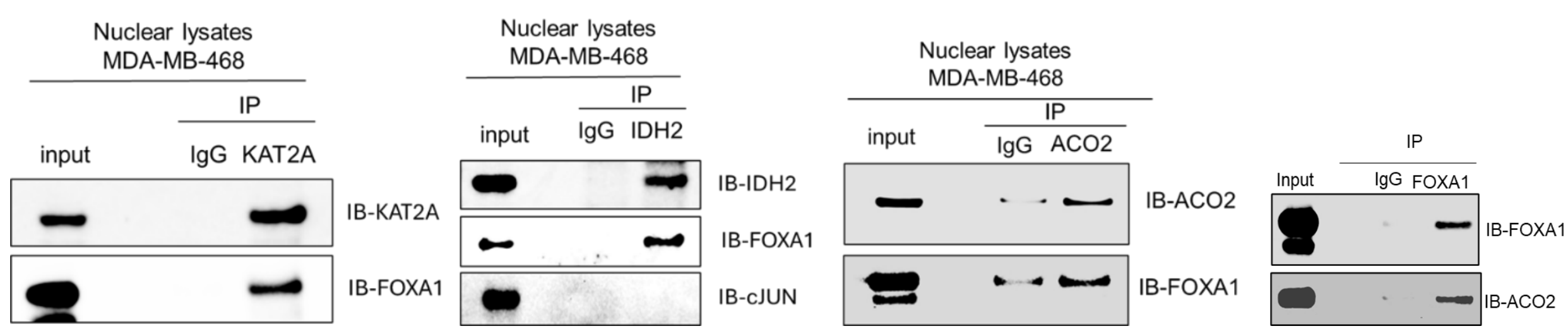

Fig R15/18. Western blot analysis of immunoprecipitated IDH2, ACO2 and KAT2A from nuclear lysates from MDA-MB-468, showing their interaction with FOXA1. IgG was used as antibody control.

Reviewer #3 (Remarks to the Author):

The authors explore a 'moonlighting' mechanism that directs the local abundance of acetyl-CoA pools by nuclear localization of mitochondrial enzymes ACO2 and IDH2. It is proposed that in the nucleus these enzymes generate citrate which can then be used as a substrate for acetyl-CoA production. Additional data suggests that subsequent histone acetylation and FOXA1 recruitment drives transcriptional responses. Increased nuclear expression of ACO2 and IDH2 was also sufficent to drive tumorigenesis. This is an extensive and interesting study that is in general carried out well. However, there remain several points of attention concerning interpretation of data and the inclusion of experiments to support conclusions that are sometimes based too much on correlations.

General Points

Unfortunaltey there is not sufficient data to support that nuclear localization of metabolic enzymes is critical for epigentic and transcriptional respones. A core conclusion of the study. Most experiments to address this issues are performed by whole cell Sh-mediated knockdowns, followed by adding back nuclear metabolic enzymes. They are often performed in isolated nuclei. This does not demonstrate that nuclear metabolic enzymes are required (under homeostatic conditions) in intact cells. The authors have identified an NLS in ACO2 that they propose is necessary for nuclear translocation. This NLS should be mutated (deltaNLS-ACO2) and shown that there is no translocaton of ACO2 to the nucleus. Under these conditions the effect of deltaNLS-ACO2 on histone acetylation and transcription should be evaluated. This would strongly support the conclusions of the study.

*We appreciate the reviewer's thoughtful feedback and agree that directly testing the requirement of ACO2's nuclear localization using a deltaNLS-ACO2 mutant would provide important mechanistic insight. ACO2 has a bipartite NLS at C-terminus (position 29) "**RAKVAMSHFEPNEYIHYDLLEKN INIVR"**. To confirm that this predicted bipartite NLS is critical for translocating the enzyme to the nucleus, we mutated the 'K' and 'R' residues (indicated in red) to alanine 'A', then sequence verified, and lentivirus expressing mutant NLS (mNLS-ACO2) were transduced into ACO2-deficient (shACO2) cells followed by stable selection of polyclonal pooled population. First, we performed immunoblotting of whole cell lysates to confirm the levels of mNLS-ACO2 and endogenous ACO2 in control (shNT) cells were similar. Next, we fractionated the control (shNT) and mNLS-ACO2 expressing MDA-MB-468 cells and found that mNLS-ACO2 failed to localize to the nucleus whereas control (shNT) cells showed robust nuclear localized ACO2. Mitochondrial localization of ACO2 was not impacted by mutating the 'ACO2-NLS' domain. Finally, we performed confocal z-section microscopy to image the distribution of ACO2 between nucleus and mitochondria. For this, we used shNT (control), shACO2, and shACO2 cells stably expressing ∆MTS-NLS-ACO2 or mNLS-ACO2 constructs. Our data revealed that mNLS-ACO2 failed to enter nucleus whereas ∆MTS-NLS-ACO2 was predominantly nuclear. These observations robustly confirm that endogenous bipartite NLS of ACO2 is essential for nuclear localization. Next, we acid extracted histones from mNLS-ACO2 cells and confirmed that the most robustly altered H3Ac marks by nuclear ACO2 as identified by isotope tracing-LC/MS (Fig. 4j) were not rescued by mNLS-ACO2. A detailed histone acetylation analysis in cells with nuclear excluding ACO2 and IDH2 is planned for future.*

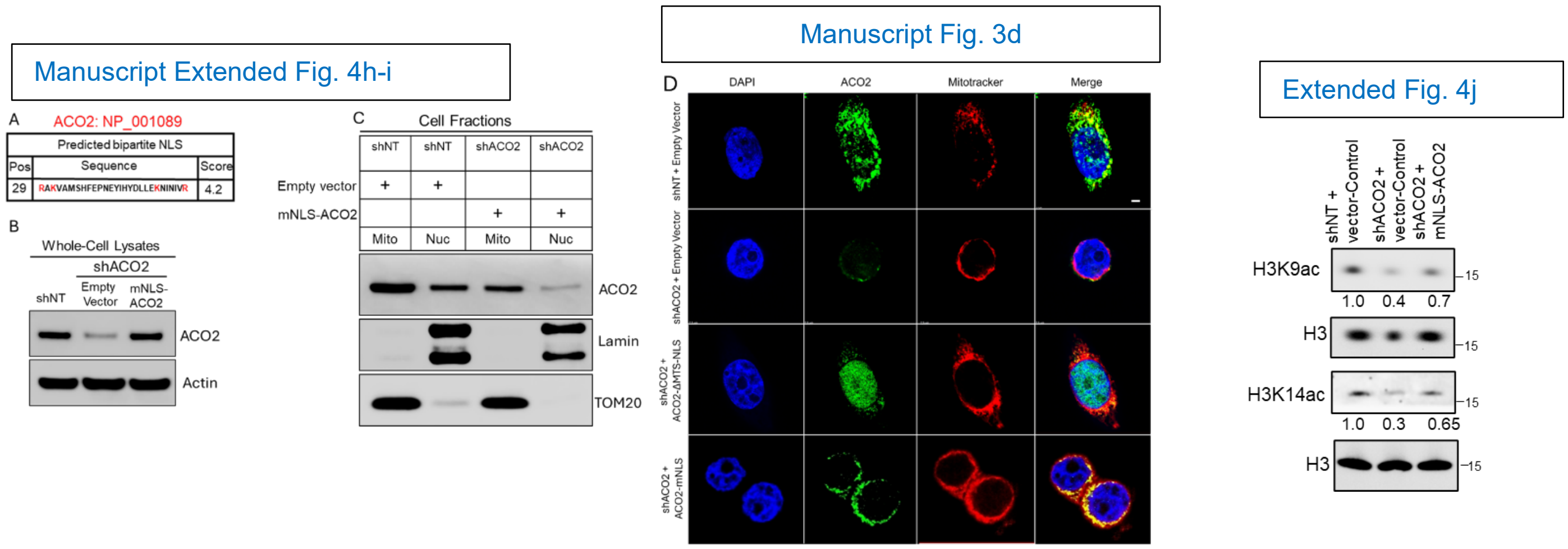

Fig. R19. A: Predicted NLS domain of ACO2. B: Levels of ACO2 shown by immunoblotting. C. Immunoblotting with cellular fractions showing the distribution of endogenous ACO2 (wild type) and ACO2 with mutated NLS (mNLS) between mitochondria and nucleus. Lamin A/C and Tom20 were used as nuclear, and mitochondrial marker proteins. D. Confocal microscopy showing the localization of endogenous ACO2 (shNT), nuclear ACO2 (∆MTS-NLS-ACO2) and mNLS-ACO2 (excluded from nucleus).

In line with this, there are no meaurements of nuclear metabolites in intact cells. To demonstrate the importance of their findings the authors should show that loss of ACO2/IDH2/ACLY modulates nuclear citrate and acetyl-CoA levels in intat cells. To do this they can emply widely available metabolic biosensors for citrate and acetyl-CoA. By generating nuclear and cytoplasmic reporters, they can directly show the impact of modulating ACO/IDH2 levels on nuclear acetyl-CoA.

To address the reviewer's comments on nuclear citrate/acetyl CoA levels, we performed additional experiments. Isolated intact nuclei from glutamine starved cells were treated with vehicle control (H2O),

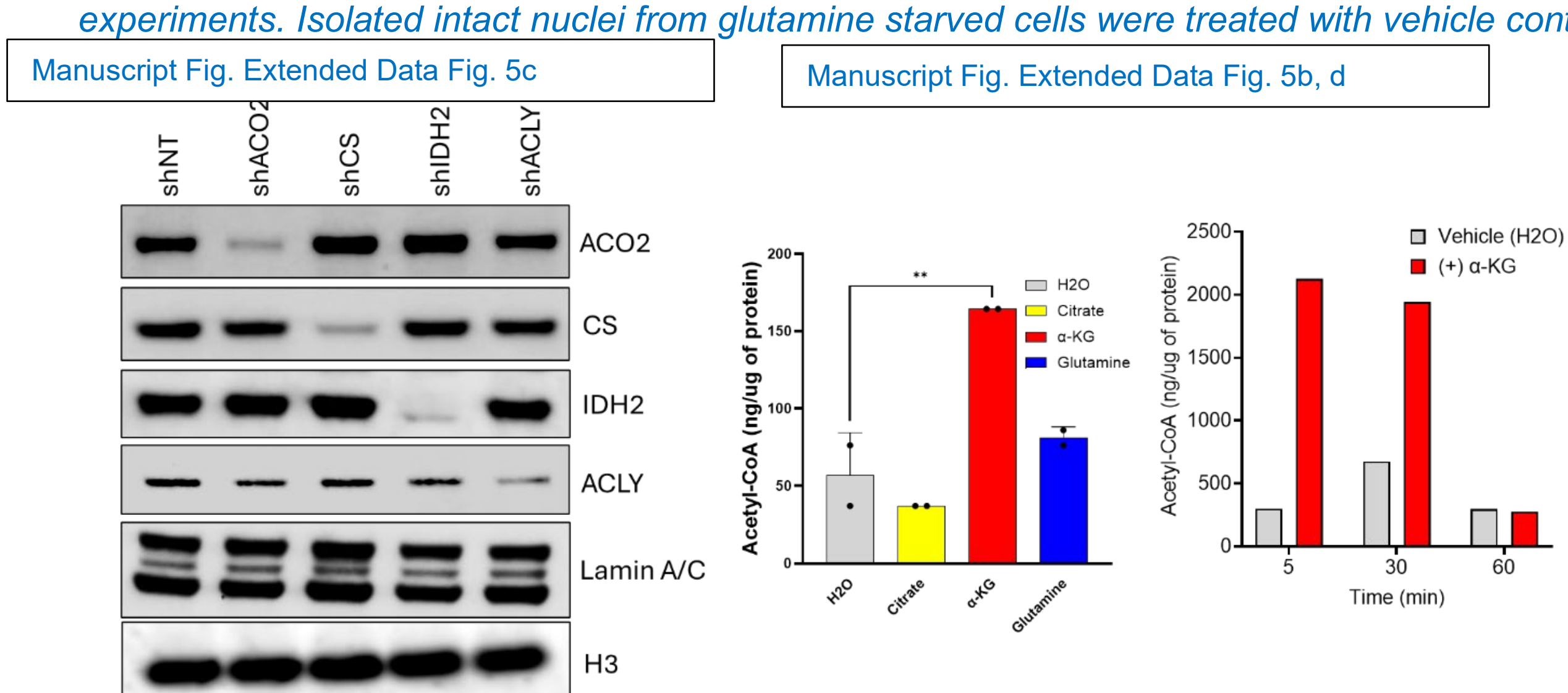

Fig R4/20. Left) Immunoblotting of nuclear lysates showing knockdown levels of ACO2, CS, IDH2 and ACLY used in Fig. 4c. Lamin A/C and histone H3 are used as a loading control. Middle) Isolated nuclei from glutamine starved GL261 cells were treated with H2O (vehicle control) citrate (2mM), alpha-ketoglutarate (2mM) and glutamine (2mM) for 1hr, followed by acetyl-CoA measurement (normalized to nuclear protein conc). Right) Isolated nuclei from glutamine starved GL261 cells were treated with vehicle control (H2O) or alpha-ketoglutarate (2mM) for 5-60min, followed by acetyl-CoA measurement (normalized to nuclear protein conc).

citrate (2mM), α-KG (2mM), and glutamine(2mM). Addition of α-KG- showed increased levels of acetyl-CoA in the nucleus compared to vehicle control (H2O), citrate and glutamine. We also conducted a time-course experiment with α-KG, which showed a rapid increase in acetyl-CoA levels until 30 minutes, followed by a decline after 60 minutes, likely due to its utilization by HAT enzymes. Both new data are now added to the manuscript (Extended Data Fig. 5b and d).

We also measured steady state levels of nuclear acetyl CoA in stable cells expressing shACO2 and shIDH2 compared to shNT (control). Interestingly, we did not observe a significant change in the steady state levels of acetyl CoA levels between these groups, although there is a trend of reduced acetyl-CoA (Fig. R5). However, we observed significant increase in acetyl CoA levels after addition of α-KG in the isolated nucleus, although the levels of free acetyl CoA levels decreased with time (Fig. R4-right panel). This could be attributed to the rapid utilization of acetyl-CoA by HAT enzymes or the inherent unstable nature of acetyl-CoA, which fails to capture the nascent acetyl CoA levels synthesized.

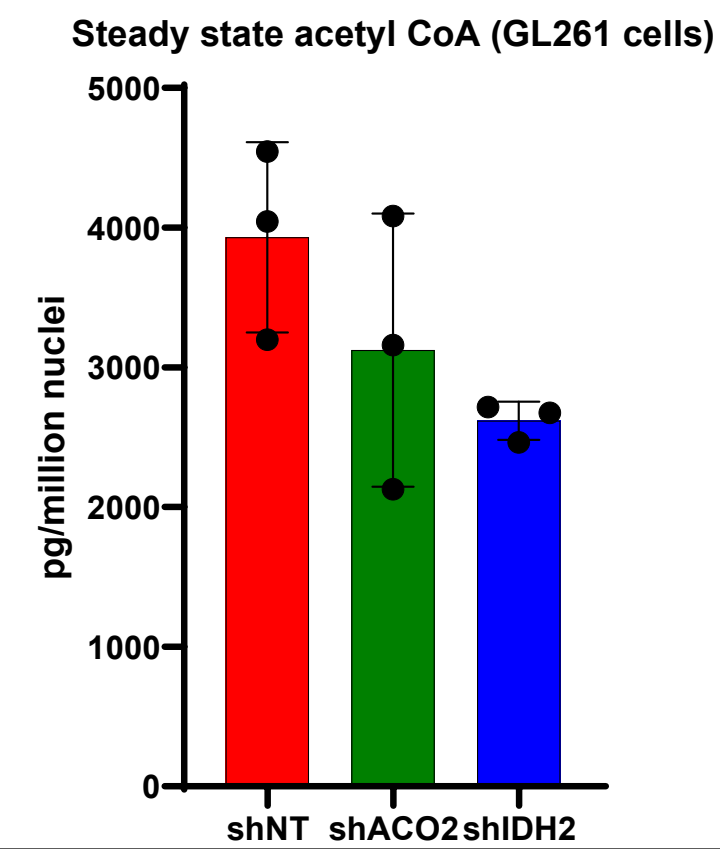

Fig R5/21. Acetyl-CoA measurement in isolated nucleus from GL261 cells stably expressing shNT, shACO2 and shIDH2.

To demonstrate that nuclear ACO2 can modulate histone acetylation in intact cells, we employed MDA-MB-468 cells expressing shNT (control), shACO2, and shACO2 cells with dox-inducible expression of ΔMTS-NLS-ACO2 and cultured them in presence of U-C13-glutamine for 2hours. The cells were harvested, washed, and histones were extracted by acid extraction followed by LC/MS proteomics to measure the enrichment of C13-acetyl (m+2) on histone marks. Our data revealed that H3Ac marks were enriched with C13 from glutamine, and loss of ACO2 significantly reduced H3K9Ac, H3K14Ac, H3K23Ac and H3K27 Ac marks. Moreover, restoring the levels of ACO2 in the nucleus using dox-inducible expression of ΔMTS-NLS-ACO2 rescued H3K14, K18 and K23Ac marks that were significantly decreased due to loss of ACO2, confirming the functional role of nuclear ACO2 regulating

Manuscript Fig. 4j

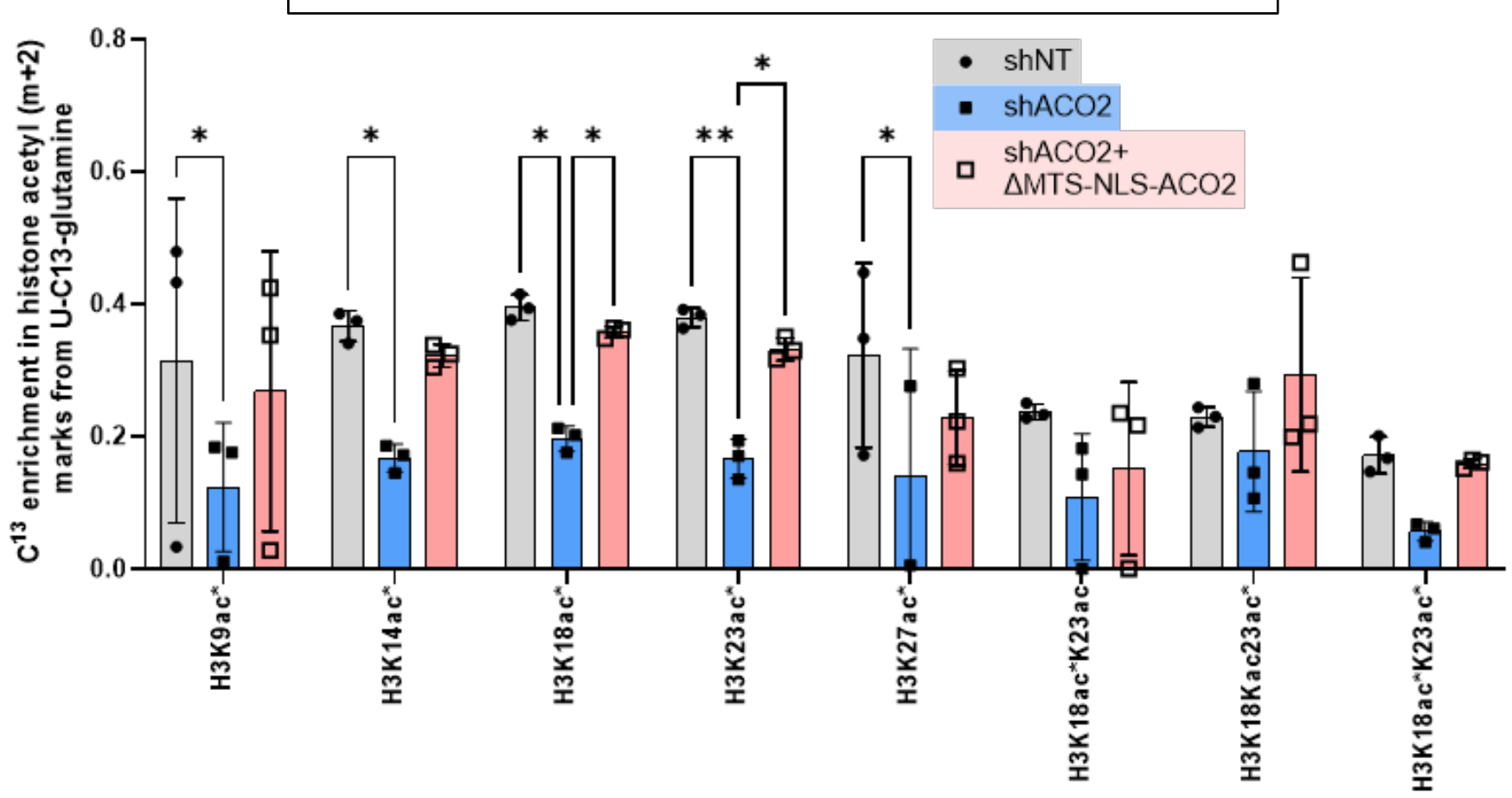

Fig R7/14/22. Fractional enrichment of acetyl C^{13} (m+2) on H3Ac marks from U-$^{13}C_5$-Glutamine in MDA-MB cells stably expressing shNT, shACO2 and shACO2 cells with ACO2-ΔMTS-NLS. Error bars are presented as mean ± SD. * P ≤ 0.05, ** P ≤ 0.01. *n=3* biological replicates.

histone acetylation. These experiments were performed with whole cells to study the endogenous ACO2 functions in addition to the experiments previously demonstrated with isolated nucleus.

We appreciate the reviewer's suggestion to assess nuclear metabolite pools and fully agree that evaluating compartmentalized levels of citrate and acetyl-CoA in intact cells using biosensors is an innovative idea but was beyond the scope of this study.

The model proposes that ACO2 generates citrate for ACLY that subsequently results in histone acetylation. However, the authors perform almost no experiments to demonstrate this critical role of ACLY. ACLY should be included in the analysis in Figure 1 as it is essential for production of acetyl-CoA, and central to the model proposed. Does driving ACLY to the nucleus, or preventing ACLY nuclear translocaion phenocopy the data presented for ACO2 and IDH2?

We thank the reviewer for highlighting the importance of including ACLY more explicitly in the model and analysis. We agree that ACLY plays a critical role in converting citrate to acetyl-CoA and is a central enzymatic node in the proposed pathway. Indeed, the nuclear function of ACLY has been rigorously characterized by multiple groups, particularly the work from Dr. Kathryn Wellen's lab, which identified both nuclear translocation and regulatory phosphorylation of ACLY [2-6].

While we excluded ACLY from Figure 1, which focuses on mitochondrial enzymes found in the nucleus, we included ACLY in later experiments where functional readouts were investigated. Specifically:

- ***Figure 2b and Extended Data Figure 3b**: We show nuclear ACLY localization in both GL261 and MDA-MB-468 cells via subcellular fractionation.*
- ***Figure 2f, Fig. 3j and Extended Data 4l**: Showing association with ACO2, IDH2 and histones, and also KAT2A.*
- ***Figures 3a–b and Extended Data 4a-d**: Demonstrating ACLY knockdown reduces histone acetylation to levels comparable with ACO2 and IDH2 depletion.*
- ***Figures 4c**: ACLY is essential for glutamine dependent histone acetylation, and its loss impacts ACO2/IDH2 nuclear functions.*
- ***Extended Data Figures 6a-b**: Loss of ACLY reduces chromatin accessibility in a manner consistent with ACO2/IDH2 knockdown.*
- ***Extended Data Figure 5k**: Nuclear ACLY levels are not altered by ACO2 or IDH2 knockdown, supporting a model where upstream nuclear citrate availability—not ACLY localization—is the regulated step.*

Together, these data demonstrate that ACLY phenocopies the effects of ACO2 and IDH2 on histone acetylation and chromatin accessibility, and functions downstream in the same nuclear complex.

Given the depth of prior literature on ACLY's nuclear metabolic function, our study was designed to focus specifically on identifying the upstream sources of citrate—namely ACO2 and IDH2—that feed this nuclear pool. Nonetheless, ACLY is functionally integrated into our study and included in multiple key datasets.

All figure legends should include number of independent experiments and statistics where appropriate. This is particulary relevant for Western Blot data, but also many other anayses. Quantification of multiple independent experiments should be performed. This is missing in many if not most experiments. For much of the work it appears that experiments were only performed once.

We have now updated the figure legends.

Proteomics and RNAseq data should be available in open access online repository. At the moment it is impossible to check results or quality while reviewing the manuscript.

The data has been submitted and accession number provided.

Specific Points

Figure 5 and Extended Figure 6-7. It is unclear how these ATAC-seq experiments are performed or how they should be interpreted. Are these whole cell ACO2-KO, or nuclear-specific ACO2-KO. What does 'KO' actually mean in this context? From the text it appears to be 'depletion' which suggests 'knock-down' (KD), but not knockout. This experimental approach needs to be better described and interpreted on basis of either whole cell loss of ACO2 or nuclear-specific loss. Why not use the shACO2+ACO2-dMTS-NLS cell context? This would seem necessary to define a role for nuclear ACO2 in chromatin accessibility and transcription.

The "ACO2-KO" (ACO2-knock out) referred to in Figures 5 and Extended Figures 6–7 was generated using a CRISPR-Cas9–based gene editing strategy in C4-2 cells and thus represents a whole-cell knockout. The clones were sequence verified with both alleles deleted. We regret the confusion caused by inconsistent terminology and have now clarified this in the revised figure legends and Methods section, explicitly distinguishing between CRISPR-Cas9 based "knockout" and shRNA mediated "knockdown" contexts.

To directly address the reviewer's suggestion, we have now performed a new ATAC-seq experiment in MDA-MB-468 cells expressing shNT (control- nontargeting), shACO2 (shRNA dependent knockdown) and shACO2 cells expressing ACO2-ΔMTS-NLS. This allowed us to assess whether nuclear-localized ACO2 are sufficient to restore accessibility of chromatin regions that were lost due to ACO2 knockdown.

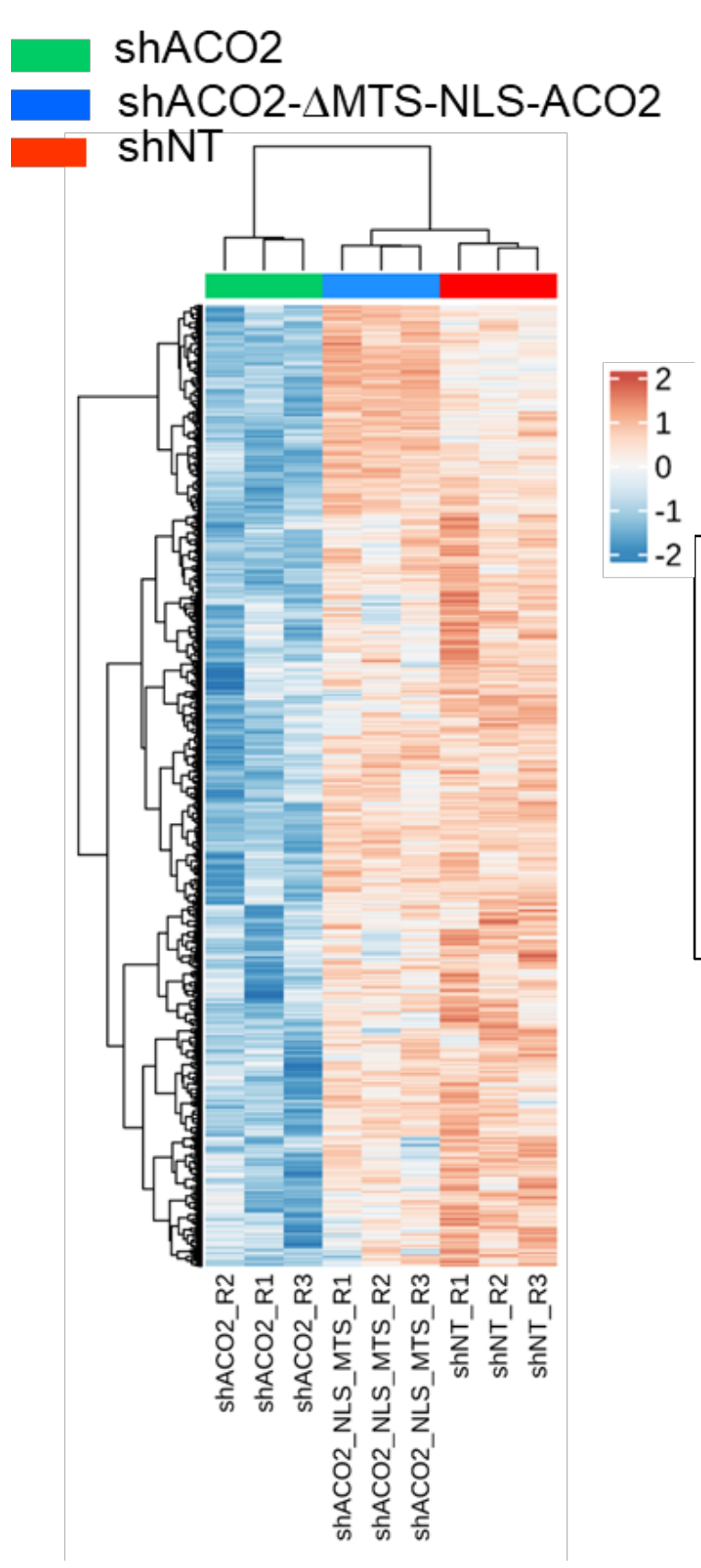

Manuscript Extended Data Fig. 7f

Fig. R23. ATAC-seq was performed using MDA-MB-468 cells expressing shNT, shACO2, shACO2 + ACO2-ΔMTS-NLS (n=3, biological replicates). Heatmap showing functional role of nuclear ACO2 on chromatin accessibility. Chromatin regions (n = 1687; accessibility loss > 1.5FC) that were lost due to ACO2 depletion (shACO2) compared to control (shNT) were restored by ACO2-ΔMTS-NLS expression.

It shows: heatmap of nuclear ACO2 function activated regions (n = 1687; accessibility loss > 1.5FC) that were lost due to ACO2 depletion (shACO2) compared to control (shNT) were restored by ACO2-∆MTS-NLS expression. There were also regions that were gained due to ACO2 knockdown but were not considered and only DARs that were restored by ACO2-∆MTS-NLS were shown here. Overall, the new ATACseq data confirms our findings that nuclear ACO2 controls chromatin accessibility. These new data is now included in the revised manuscript, and we thank the reviewer for prompting us to strengthen this conclusion.

Figure 6. The FOXA1 data should be removed from the paper as it doesn't strengthen the story. To include this data it is necessary to perform a series of additional experiments with FOXA1 KO in vitro and in vivo to demonstrate that it is indeed responsible for mediating the effects of nuclear ACO2. Without this, the data presented is correlative and is not required.

We thank the reviewer for this thoughtful comment and agree that the FOXA1 linking to ACO2 and IDH2 nuclear functions are correlative in nature. To address this comment (and also suggested by reviewer#2), we have done additional experiments. *We performed IP/IB of ACO2, IDH2 and KAT2A from the nuclear lysates to determine whether KAT2A-containing complex of ACO2/IDH2 interacts with FOXA1 or cJUN, both were predicted TFs from the ATACseq motif analysis. Our data revealed that KAT2A-chromatin regulatory complex containing ACO2-IDH2 both interacts with FOXA1 but not cJUN, suggesting FOXA1-specific genomic loci may be the primary targets in the cell lines that we have investigated. We are further investigating this in the context of prostate cancer, where FOXA1 is associated with aggressive disease. We have revised the manuscript text to clarify this point and mentioned additional studies are required to investigate the mechanistic role of FOXA1 in metabolic-epigenetic regulation.*

Manuscript Fig. 6h-k

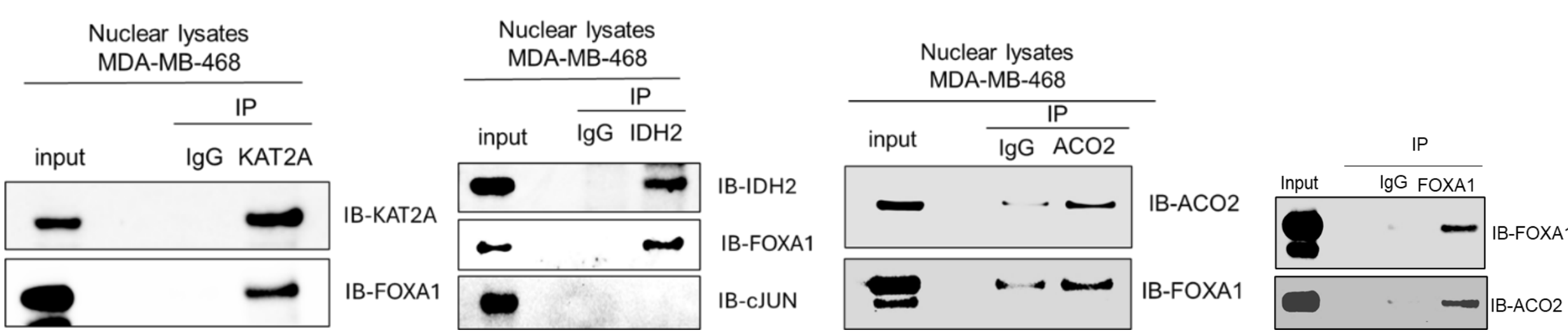

Fig R15/18/24. Western blot analysis of immunoprecipitated IDH2, ACO2 and KAT2A from nuclear lysates from MDA-MB-468, showing their interaction with FOXA1. IgG was used as antibody control.

Figure 6d. A significant reduction if IL1RN might be the case but this is so minor that it is hard to understand if this would have a functional effect. I would remove this rather unconvincing data from the paper. Are these the only two genes that show any significant decrease in mRNA expression under these conditions? What about the other 40 genes presented in Figure 6c?

We appreciate the reviewer's concern regarding the modest change in IL1RN expression. While the reduction in mRNA levels is relatively small, we have retained this data because it is supported by ChIP-qPCR data—FOXA1 occupancy at the IL1RN locus is significantly decreased upon ACO2 knockdown and fully restored with nuclear-specific ACO2 rescue. Among the other 40 genes that were

found to be significantly altered, we could only validate FoxA1 occupancy on the promoters of IL1RN and MKI67. Multiple primers were used for ChIP-qPCR but the data were inconsistent, hence we presented the data showing FoxA1 dependent transcriptional regulation of IL1RN and MKI67 modulated by ACO2.

Minor Points

Figure 1b. The authors describe considerable heterogeneity of ACO2 and IDH2 nuclear localization. Have they correlated this with cell division/proliferation? Or do they perhaps have an alternative explanation?

We thank the reviewer for highlighting the observed heterogeneity in ACO2 and IDH2 nuclear localization. This variability is indeed prominent across the cancer cell lines we tested and likely reflects underlying biological differences among tumor types. We performed cell cycle analysis, but did not find any significant changes due to ACO2 or IDH2 knockdown.

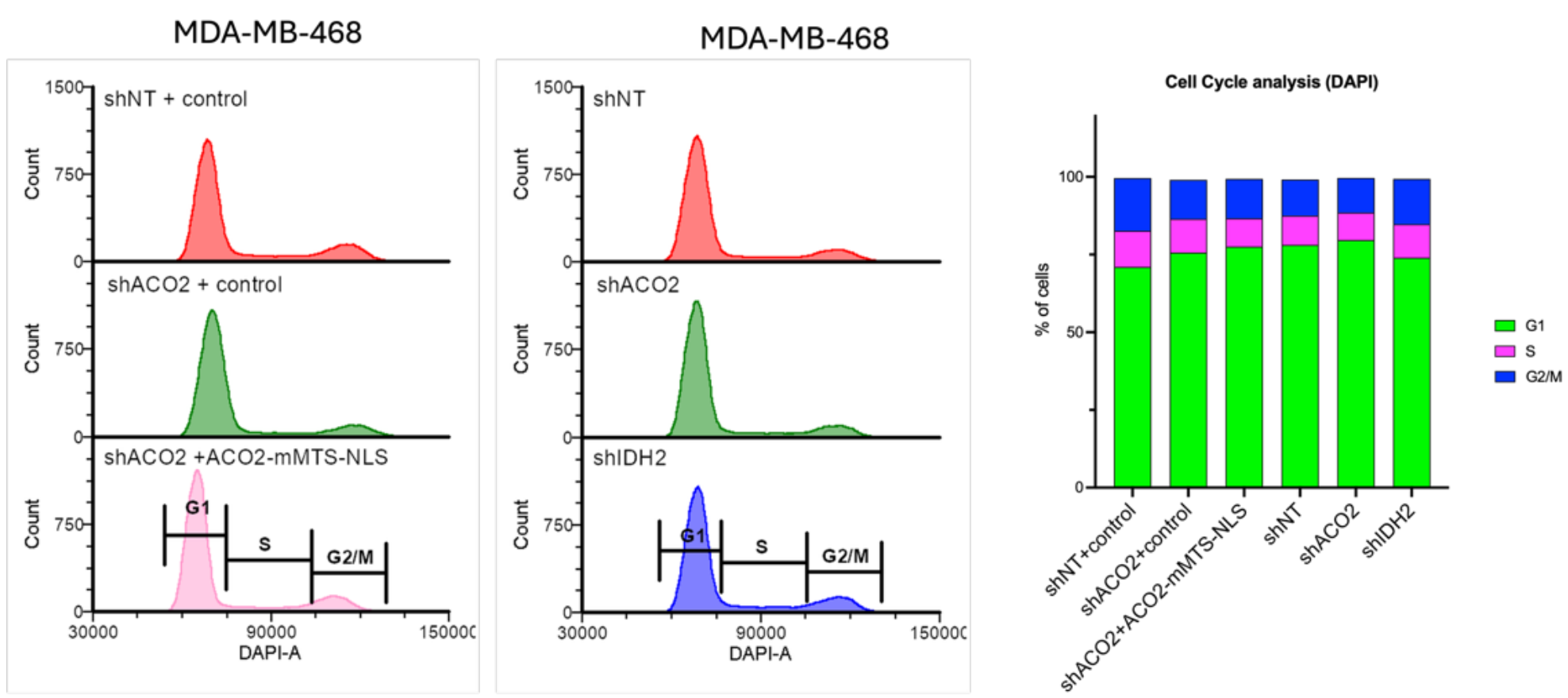

Fig. R17/25: Graph showing DNA content of MDA-MB-468 cells based on DAPI intensity analyzed by flow cytometry. Cells were gated into G1, S and G2/M phase based on DAPI intensity. Percentage of cells in each phase are shown in bar graph.

One plausible explanation is that the heterogeneity stems from intrinsic differences in the metabolic wiring of various cancer types. Tumors with reduced reliance on mitochondrial oxidative metabolism—whether due to oncogenic rewiring or microenvironmental constraints—may allow for a greater pool of mitochondrial enzymes to relocalize to the nucleus. In this context, diminished mitochondrial activity could free ACO2 and IDH2 from their canonical roles, enabling their repurpose in the nucleus for chromatin regulation and transcriptional control. This model aligns with the broader concept of cancer cell plasticity and metabolic adaptability, which we are planning to test out soon.

Page 7. The authors write 'ACO2 was identified to have a strong affinity towards ACLY'. There data does not support this statement, they show a non-quantitaive co-IP. The text should be modified to : 'can interact with ACLY'.

We have revised the text to state that “ACO2 can interact with ACLY” to more accurately reflect the data presented.

Figure 2i. The authors show that ACO2 and IDH2 interact. They further show that ACLY only inreracts with ACO2. How do they explain this? Are there independent ACO2-IDH2 and ACO2-ACLY complexes? In the model in Figure 3h they show that all three proteins are in one complex which doesn’t correlate with the data.

We appreciate the reviewer’s close attention to the interaction data and the proposed model. Our co-immunoprecipitation experiments consistently show robust and reproducible interactions between ACO2-IDH2 and ACO2-ACLY across multiple cell lines and biological replicates. In contrast, direct IDH2-ACLY interaction is not consistently observed in our IP/IB experiments—it is detectable in some cell lines but not all.

This variability suggests that the interactions between ACO2-IDH2 and ACO2-ACLY may form more stable or predominant sub-complexes, while a full ternary complex involving all three proteins may be transient, or formed at low abundance. Given the sequential nature of their enzymatic activities—where IDH2-derived isocitrate is converted to citrate by ACO2, and citrate is then used by ACLY to generate acetyl-CoA—it is plausible that IDH2 need not physically interact with ACLY but instead relies on ACO2 as a metabolic and physical bridge.

We have revised the text and updated the model in Figure 3k (previously 3h) to reflect this possibility, now depicting the ACO2-IDH2 and ACO2-ACLY interactions as more central, with potential for transient ternary complex formation.

The term ‘moonlighting’ is misleading. It suggests that these metabolic enzymes have an additional function. I would re-phrase this.

We have updated the writeup.

References:

1. Demicco, M., et al., *Metabolic heterogeneity in cancer.* Nat Metab, 2024. **6**(1): p. 18-38.
2. Carrer, A., et al., *Acetyl-CoA Metabolism Supports Multistep Pancreatic Tumorigenesis.* Cancer Discov, 2019. **9**(3): p. 416-435.
3. Santarsiero, A., et al., *ACLY Nuclear Translocation in Human Macrophages Drives Proinflammatory Gene Expression by NF-kappaB Acetylation.* Cells, 2021. **10**(11).
4. Zhao, S., et al., *ATP-Citrate Lyase Controls a Glucose-to-Acetate Metabolic Switch.* Cell Rep, 2016. **17**(4): p. 1037-1052.
5. Sivanand, S., et al., *Nuclear Acetyl-CoA Production by ACLY Promotes Homologous Recombination.* Mol Cell, 2017. **67**(2): p. 252-265.e6.
6. Ma, Y., et al., *The Nuclear Localization of ACLY Guards Early Embryo Development Through Recruiting P300 and HAT1 to Promote Histone Acetylation and Transcription.* Adv Sci (Weinh), 2025: p. e14367.
7. Gates, L.A., et al., *Histone butyrylation in the mouse intestine is mediated by the microbiota and associated with regulation of gene expression.* Nat Metab, 2024. **6**(4): p. 697-707.
8. Lund, P.J., et al., *Stable isotope tracing in vivo reveals a metabolic bridge linking the microbiota to host histone acetylation.* Cell Rep, 2022. **41**(11): p. 111809.

Reviewer #1 (Remarks to the Author):

Sawant et al report that “Nuclear-specific reductive carboxylation of alpha-ketoglutarate fuels histone acetylation to induce FOXA1-dependent gene activation”. The authors perform a comprehensive study of the presence of TCA cycle enzymes in the nucleus. Specifically, they describe that ACO2, IDH2 and ACLY localize at the chromatin forming a metabolic complex that includes HAT KAT2A.

I thank the authors for addressing most of my previous concerns and I believe the new data and added clarifications strengthen the work.

> *We thank the reviewer#1 for appreciating our work. We appreciate helpful suggestions and have addressed all the remaining minor comments.*

Some minor concerns remain from the arguments posed in the rebuttal:
- Metabolic analysis: The fact that metabolite measurement is performed in cells after HAT assay should also be mentioned in the manuscript text (apart from the methods section).

> *We added the information in manuscript text (page 10, line 19-20).*

- MNase digestion: The added controls increase the quality of the data and strengthen the conclusion. The quantification should be represented as the mean of all replicas performed and added in the manuscript.

> *We have added the means of all the replicas performed and updated the Extended Data Fig. 6c.*

-Nascent RNA synthesis: Regarding the representation as a violin plot (which is missing the label of axis Y), unless the quantification is just of one experiment (it would not make sense to have stats there then), I do not understand the authors feeling that it does not represent correctly the result. According to the statistical test, it goes in line with their conclusion and I think, together with the original plot, makes the data more robust.

> *As suggested, we have added both figures to the manuscript (Fig. 5c and Extended Data Fig. 6e).*

-ATAC-seq: I understand the rationale of the authors to change the cell line in this case. However, they should at least show the expression changes observed in MKI67 and IL1RN in the cell lines used throughout the paper the paper. Moreover, the information of altered peaks obtained in the ATAC-seq etc, should be mentioned in the ATAC-seq tornado plots (fig. 5e), together with numbers on the axes of the upper plots showing total binding.

> *Expression of MKI67 and IL1RN in MDA-MB468 cell line shown are shown in <u>Fig. 6d, e</u>. MKI67 expression also measured by IHC in xenograft tumors <u>(Figs. 7f, g, h, i)</u>. We have also added Mki67 expression data for GL-261 (Extended Data Fig. 7e).*
>
> *Differential accessibility, i.e. number of peaks lost in ACO2-KO (n = 16,626) now mentioned in the figure legends.*
>
> *Thank you for pointing out the missing numeric y-axis labels on the density plots in Fig. 5e which were erroneously cropped when compiling this figure - we have retrieved and reinserted the values into the upper panel.*

In vivo data: The authors disregarded the fact that in vivo statistical analysis should be performed using non-parametric tests. The stats shown (would be better to represent exact p values) do not represent the tumor variability observed. Thus, I insist on repeating the analysis with non-parametric tests. In the case statistical significance is not reached due to the variability, it could also be represented as tumor growth rate, based on calculation of the slope.

As suggested we repeated the statistical analysis using non-parametric tests (Friedman test for more than two groups) and the corrected analysis now shown in Fig. 7d and 7e.

Reviewer #2 (Remarks to the Author):

I am impressed by the number of additional data provided by the authors to address my concerns and the one's raised by the other reviewers. They now convincingly show that a substantial amount of acetyl groups on the histones is indeed derived from glutamine, suggesting that this might be an important source of nuclear acetyl-CoA. The NLS mutation further supports the hypothesis of the importance of a nuclear localisation of these enzymes (but not necessarily their activity). I am still not fully convinced that the synthesis happens in situ rather than generally increasing nuclear acetyl-CoA but it is certainly a valid hypothesis. The co-IP shows an interaction, but it does not necessarily prove that the synthesis happens only at these sites. The authors have tempered their statements along these lines.

Thank you for your kind comments, and helpful advice. We are exploring this further.

Reviewer #3 (Remarks to the Author):

The authors have done an excellent job in extensively addressing the reviewers comments.

We thank you for your helpful critiques and suggestions to strengthen the manuscript.

Reviewer #1 (Remarks to the Author):

I thank the reviewers for addressing all the remaining minor concerns.

We like to thank Reviewer#1 for constructive feedback on our work, and suggestions provided was instrumental for increasing the merit of the work.

Just two notes on my side:
- The axis of the violin plot added in Extended Data Fig. 6e, should go from 0-5000.

We made the change in the axis and now the range is 0-5000 as suggested.

- I believe lines 16-17 of page 83 are out of place.

Yes, thank you. We have now made formatting corrections.
